# Amphioxus muscle transcriptomes reveal vertebrate-like myoblast fusion genes and a highly conserved role of insulin signalling in the metabolism of muscle

**DOI:** 10.1186/s12864-021-08222-9

**Published:** 2022-02-01

**Authors:** Madeleine E. Aase-Remedios, Clara Coll-Lladó, David E. K. Ferrier

**Affiliations:** The Scottish Oceans Institute, Gatty Marine Laboratory, School of Biology, University of St Andrews, St Andrews, Fife, KY16 8LB UK

**Keywords:** Cephalochordate, Lancelet, Muscle development, Insulin Growth Factor, FOXO, Gene duplication, Genome duplication

## Abstract

**Background:**

The formation and functioning of muscles are fundamental aspects of animal biology, and the evolution of ‘muscle genes’ is central to our understanding of this tissue. Feeding-fasting-refeeding experiments have been widely used to assess muscle cellular and metabolic responses to nutrition. Though these studies have focused on vertebrate models and only a few invertebrate systems, they have found similar processes are involved in muscle degradation and maintenance. Motivation for these studies stems from interest in diseases whose pathologies involve muscle atrophy, a symptom also triggered by fasting, as well as commercial interest in the muscle mass of animals kept for consumption. Experimentally modelling atrophy by manipulating nutritional state causes muscle mass to be depleted during starvation and replenished with refeeding so that the genetic mechanisms controlling muscle growth and degradation can be understood.

**Results:**

Using amphioxus, the earliest branching chordate lineage, we address the gap in previous work stemming from comparisons between distantly related vertebrate and invertebrate models. Our amphioxus feeding-fasting-refeeding muscle transcriptomes reveal a highly conserved myogenic program and that the pro-orthologues of many vertebrate myoblast fusion genes were present in the ancestral chordate, despite these invertebrate chordates having unfused mononucleate myocytes. We found that genes differentially expressed between fed and fasted amphioxus were orthologous to the genes that respond to nutritional state in vertebrates. This response is driven in a large part by the highly conserved IGF/Akt/FOXO pathway, where depleted nutrient levels result in activation of FOXO, a transcription factor with many autophagy-related gene targets.

**Conclusion:**

Reconstruction of these gene networks and pathways in amphioxus muscle provides a key point of comparison between the distantly related groups assessed thus far, significantly refining the reconstruction of the ancestral state for chordate myoblast fusion genes and identifying the extensive role of duplicated genes in the IGF/Akt/FOXO pathway across animals. Our study elucidates the evolutionary trajectory of muscle genes as they relate to the increased complexity of vertebrate muscles and muscle development.

**Supplementary Information:**

The online version contains supplementary material available at 10.1186/s12864-021-08222-9.

## Introduction

Regulating metabolic rate in response to energy availability is a complex and essential aspect of survival. In many animals, a major source of energy is muscle, the degradation, maintenance, or growth of which is determined by the balance of cellular anabolism and catabolism. A period of low nutrient availability, i.e., fasting, causes muscles to be broken down by autophagy, where structural proteins are disassembled for their components. This process can also occur in several diseases [9, 137], and as a symptom of ageing [39]. Onset of this process involves nutrition-sensitive signalling pathways and results in changes in expression of autophagy or muscle growth genes [5]. While this general mechanism appears to be shared amongst most animals, there are important distinctions between the muscle physiology of vertebrates and certain invertebrates.

Vertebrate skeletal muscles consist of multiple fibres of multinucleate striated myotubes, full of actin-myosin cytoskeletal fibrils that constitute the primary protein reservoir in the body. While it has been found that some arthropods also form multinucleate cells [1, 25, 122], many other invertebrates have been found with only mononucleate muscle cells, including cnidarians [109], nematodes [82], annelids [100, 130], molluscs [44, 59, 89], and the invertebrate chordates amphioxus [31]. Interestingly, members of the other invertebrate chordate lineage, tunicates, have been found to have multinucleate muscle cells in the adult body wall musculature, which arise via myoblast fusion [102].

Myoblast fusion is a process integral to the formation of multinucleate muscle fibres, but has only thoroughly been investigated in a few species, primarily flies, mice, and zebrafish [1, 103]. This process requires cells to migrate, recognise, and adhere to one another before they can fuse, and is therefore a complex multi-step molecular mechanism. In vertebrates, myoblasts fuse into nascent myotubes in the first phase of fusion, then myotubes fuse together in the second phase [47], resulting in myofibres containing hundreds or thousands of nuclei. In flies, muscles are composed of single myotubes that grow from the initial asymmetric fusion of a fusion-competent myocyte (FCM) expressing membrane signals with a founder cell expressing the corresponding membrane receptors [122].

Sns (*Sticks and stones*) [11] and Hbs (*Hibris*) [4, 28, 110] in FCMs bind to Duf (*Dumfounded* a.k.a. *Kirre* (*Kin of Irregular-chiasm-C*)) [105] and Rst (*Roughest* a.k.a. *IrreC* (*Irregular-chiasm-C*)) [119] in the founder cell. This process is remarkably conserved between flies and vertebrates [116], where the Sns/Hbs orthologue Nephrin is required for myoblast fusion [113], and this binds to the Duf/Rst vertebrate orthologues Kirrel1, Kirrel2, and Kirrel3 (a.k.a. Neph1, Neph2, Neph3) [27]. At the site of fusion, the Arf-GEF Schizo (a.k.a. loner) removes N-cadherin from the membrane to allow for fusion between the growing myotube and FCMs in *Drosophila* [26], and the vertebrate orthologues Brag2 and Cadherin-15 (M-Cadherin) play similar roles [19, 21]. These interactions trigger the intracellular response, mediated by Dock1/Dock5 and its adaptor proteins Crk and Crkl in vertebrates, and in *Drosophila*, where its Dock orthologue Mbc also binds Crk [29, 83, 103]. Then, Dock/Crk activates Arf6 and Rac [19], which use the scar/WAVE complex to activate the Arp2/3 complex that can induce the actin remodelling required for fusion [6, 73]. This process is again orthologous in vertebrates (WASP/WAVE; ARP2/3), though the components of these complexes have not been characterised as fully as in flies [40].

Because the genetics of myoblast fusion were originally characterised in fly models, there are several genes identified in flies for which vertebrate orthologues do not exist, whose functions have not been identified, or have been identified with functions unrelated to myoblast fusion. The gene *blown fuse*, the protein of which regulates the stability of the scar/Wave complex and its role in actin regulation, has only been found in flies [50, 108]. The protein of the fly gene *rolling pebbles* (a.k.a. *Anti-social*) links the signal from Duf/Rst to Mbc, but its vertebrate orthologues TANC1 and TANC2 have only been found in neurons [120]. The above similarities, and these differences described here reflect an established approach to understand myoblast fusion by identifying whether vertebrate orthologues of fly fusion genes have a role in vertebrate myoblasts.

Vertebrate-centred approaches to understanding the molecules involved in myoblast fusion have searched for genes expressed along with known muscle genes like the Myogenic Regulatory Factors (MRFs), which identified the transmembrane protein Myomaker (MymK, a.k.a. Tmem8c) in mice [79] and zebrafish [62]. MymK is shared between invertebrate urochordates and vertebrates (known together as Olfactores), is expressed in myoblasts, and is essential for fusion [144]. Its partner, Myomixer (MymX) is specific to the vertebrates, and the combinatorial action of these two proteins may underpin the higher rate of myoblast fusion in vertebrates [8, 145]. Vertebrate cell culture experiments found Neogenin and Netrin to be expressed in myoblasts, and determined their essential role in regulating myotube formation [54], though these genes were first identified in the vertebrate nervous system, and the function of their fly orthologues (Frazzled and NetA) has only been reported for the nervous system and eye development [2, 81]. Other studies have found candidates through querying extracellular protein interaction databases for muscle genes, which identified Jam-B and Jam-C [97]. Neither of these have been found to have orthologues in flies.

Studies in vertebrates and flies have shown that similar mechanisms work to regulate the cellular response to nutrition in muscles [15]. Insulin Growth Factor (IGF) signalling is a major controller of skeletal muscle size in vertebrates [37, 92], and represents the direct relationship of nutrient availability to muscle maintenance. IGF mediates the signal from Growth hormone (Gh) to enact the changes observed in muscle cells. Muscle hypertrophy can be induced by Gh, resulting in increased myoblast fusion and myofibre size [115], and IGF-1, which results in increased protein synthesis and a downregulation of muscle-specific ubiquitin ligases [37]. The relationship of IGF and its downstream pathway in muscle growth or degradation has been determined in mice [78, 84, 106], fish [12, 20, 34], chicken [86], and the invertebrate models fruit fly [36, 124], nematode [94], oyster [51] and hydroid [72] responding to nutritional state.

In muscle, this pathway carries the signal of nutrient availability through a series of kinases resulting in the suppression of FOXO transcription factors, which regulate genes for the autophagic process. In times of low nutrient availability, i.e., fasting, FOXO transcription factors are not phosphorylated, and can enter the nucleus to activate their target genes. In starved mice, the ratio of phosphorylated to unphosphorylated FOXO decreases, as it is disinhibited by the upstream INS/Akt signalling [106]. FOXO transcription factors regulate proteolysis in a variety of ways in muscle cells, stimulating both lysosomal and proteasomal proteolysis [69, 114, 118, 146]. The primary muscle autophagy-related targets of FOXO transcription factors are the E3 ubiquitin ligases *MAFbx* and *MuRF1*, which are upregulated by FOXO in starved muscle and their proteins target muscle genes for degradation [32, 106]. Other FOXO targets are varied [131], and include *LC3b*, *Gabarapl1*, *Vps34*, *Ulk2*, *Atg12l*, *Atg4b*, and *Beclin1* [136, 146]. In mice, FOXO transcription factors regulate a group of E3-ubiquitin ligases including *Fbxo30* (*MUSA1*), *Fbxo31*, *Fbxo21*, and *Itch*, as well as proteasome subunits, ubiquitin and related genes, and markers of the autophagosome, e.g. *LC3* [78]. FOXO orthologues activate orthologous targets in vertebrates, flies, and nematodes [24, 75, 77, 85, 98, 99, 121, 131], suggesting a conserved repertoire of autophagy genes regulated by FOXO transcription factors. Across these animals, key players in the nutrition-response pathways are repeatedly triggered, illustrating the highly conserved nature of this pathway.

Other signalling pathways are also triggered in feeding-fasting experiments, including the Ras/Raf/MAPK pathway and mTOR downstream of Akt [74]. Both of these pathways are highly conserved. In the case of MAPK, EGF levels result in activation of the bHLHZ transcription factor MYC [70], while mTOR is involved in growth and transcription downstream of insulin/IGF signalling [17]. Both of these pathways show a similar pattern of increasing complexity in vertebrates (MAPK [7]; mTOR [90]). This is not unexpected, as the ancestor to vertebrates underwent two rounds of whole genome duplication (2R WGD), which has been credited with the evolution of more complex body plans and the many vertebrate novelties that arose at this time [16, 46].

From an evolutionary standpoint, the limited number of invertebrate models studied in the IGF/Akt/FOXO context means that we are only able to tentatively infer a pattern of increasing complexity moving from invertebrate to vertebrate animals. It has been noted that in such a highly conserved pathway in cnidarian, fly, nematode, and vertebrate models [72, 93], that the vertebrates have more genes for many of the proteins in the pathway [123]. For instance, vertebrates have four FOXO transcription factors, corresponding to only single genes in the urchin [129], tunicate [139], amphioxus [134, 141], fly [52], and nematode [91]. FOXO targets also show redundancy among the vertebrates, including three paralogues of *MuRF1* (a.k.a. *TRIM63*): *TRIM54*, *TRIM55*, and *TRIM101* [10, 68, 132], and three paralogues of *MAFbx* (a.k.a. *FBXO32*): *FBXO25*, *FBXO30*, and *FBXO40* [22, 49, 107, 140]. The one-to-four ratio of invertebrate to vertebrate FOXO genes illustrates how 2R may have played a major role in the increasing complexity at the invertebrate to vertebrate transition, though a more thorough analysis is required.

The current understanding of the relationships amongst the insulin/IGF parts of the pathway and the role of gene duplications at the origin of vertebrates is even less clear. Non-teleost vertebrates have up to two insulin and two IGF genes, compared to one or two ILPs (insulin like peptides) in some invertebrates [63, 76], though insects have undergone their own lineage-specific expansion of ILPs [3, 38, 87] as has the nematode [96] and the oyster [65]. In this case, the increased number of *INS/IGF* genes in vertebrates relative to the single presumed ancestral invertebrate gene is obscured. Including other invertebrate lineages without unique expansions in this gene family may show the pattern between ancestral invertebrate orthologues and possible vertebrate paralogues more clearly. A similar study determined the complement of MAPK genes in amphioxus [7], noting that while the pathway was highly conserved between amphioxus and vertebrates, the 2R WGD resulted in several paralogues of many of the kinases in vertebrates corresponding to a single amphioxus gene. This points to the role of 2R in increasing complexity at the invertebrate to vertebrate transition, and therefore a similar pattern may be expected for the amphioxus IGF/Akt/FOXO pathway.

Little is known about the IGF/Akt/FOXO pathway in amphioxus. Some studies have identified the amphioxus insulin and insulin-like peptides, as genes in this family have been widely studied, and orthologues have been found from across metazoans, as discussed above [18]. Amphioxus has one insulin-like peptide (ILP) and one IGF gene [41, 48, 63]. Forkhead box genes have also been identified in *B. floridae*, which has one FOXO orthologue [141]. While these studies suggest that a few steps in the IGF/Akt/FOXO pathway are intact and conserved in function in amphioxus, orthologues of genes for proteins making up the rest of the pathway, as well as the FOXO-controlled atrogenes and their regulation in response to nutrition, are yet to be identified.

Here we focus on the invertebrate chordate amphioxus (*Branchiostoma lanceolatum*) as it provides an excellent point of comparison to bridge the gap between the invertebrates and vertebrates for which key components of myogenesis have been established, including myoblast fusion genes and the IGF/Akt/FOXO pathway. Our amphioxus muscle transcriptomes reveal that many myoblast fusion genes are present and expressed in amphioxus muscles, and that amphioxus has orthologues of the myoblast fusion genes that were previously thought to be vertebrate-specific. We also found that nutritionally challenged amphioxus muscles respond in similar ways to commonly studied vertebrate and fly model organisms, in agreement with the high level of conservation of the IGF/Akt/FOXO pathway controlling this response. We also reconstructed the complete amphioxus IGF/Akt/FOXO pathway, many of the genes of which illustrate the effect of the 2R WGD on the increased complexity of the vertebrates. This work provides many candidate genes for further studies in amphioxus and other key lineages to fully understand the evolution of muscle development.

## Results and discussion

### Clarification of the ancestral chordate myoblast fusion gene complement

We first generated a transcriptome of *B. lanceolatum* muscle, stimulating gene expression with nutritional challenge, which resulted in 355,725 reads assembled into 14,854 isotigs, 7556 of which were annotated, and were joined into 7352 isogroups, finally representing 4022 annotated genes (see Materials and methods; Additional file 1). We then searched this transcriptome to identify amphioxus orthologues of genes known to be expressed in muscles of other species. Within our transcriptome, we found orthologues of many genes characteristic of vertebrate muscles and myogenesis. In particular, with respect to genes involved in myoblast fusion, we found that orthologues of myogenic genes in vertebrates are present in amphioxus and many are expressed in the muscle transcriptome (Table 1). This includes what were previously thought to be the vertebrate-specific muscle genes, Jam-B and Jam-C (amphioxus orthologue BL01782). Also expressed in muscle are Netrin (amphioxus orthologue BL15668) and its receptor Neogenin (amphioxus orthologue BL15756), which are required for vertebrate myoblast fusion, but in *Drosophila*, their functions have until now only been reported in neurons. A similar pattern is observed for the transcription factor NFAT (BL23062), expressed in amphioxus muscles, the vertebrate orthologue of which, NFATC2, regulates myoblast fusion, but where the fly gene has not been characterised with a function in myoblast fusion. Like vertebrates, no orthologue of the fly gene *Blown fuse* was found in the amphioxus genome or transcriptome. Besides these lineage-specific genes, amphioxus has orthologues of all the components of myoblast fusion genes that are shared between flies and vertebrates (Table 1). While amphioxus lacks multinucleate myofibres, its muscles still express many of the genes that control fusion in the muscles of other species.

**Table 1:** Human genes involved in myoblast fusion and their fly and amphioxus orthologues, many of which were detected in the amphioxus muscle transcriptome, and one of which was differentially expressed. P in parentheses denotes paralogues not involved directly in myoblast fusion in humans. A larger table including descriptions of their relevant functions can be found in Additional file 1

Process		Human gene	Fly gene	Amphioxus orthologues	Expressed in muscle	Differentially expressed
Myoblast fusion	Cell recognition	Nephrin (NPHS1) (P: NPHS2)	sns (Sticks and stones) & hbs (Hibris)	NPHS	–	
		Kirrel1, Kirrel2, Kirrel3	duf (Dumfounded) & rst (Roughest)	Kirrel	–	
		Myomaker (TMEM8C) (P: TMEM8A & TMEM8B)	CG13654	Tmem8a/b/c	yes	–
		Myomixer	–	–	–	
		TANC1, TANC2	rols (Rolling pebbles, a.k.a. Anti-social)	TANC	–	
		Junctional adhesional molecule 2 (JamB) & 3 (JamC) (P: JamA)	–	JAM	–	
		Netrin	netA (Netrin-A)	NTN1	yes	–
		Neogenin	fra (Frazzled)	Neo	yes	–
	Cell adhesion	Cadherin-2 (M-cadherin)	–	Cdh15	yes	–
		Cadherin-15 (N-cadherin)	CadN (Cadherin_N)			
		Caveolin1, Caveolin2, Caveolin3	Caveolin-1	Cav1/3	yes	–
		Myoferlin (a.k.a. Fer1L3) (P: Dysferlin Fer1L1, Fer1L5)	mfr (Misfire)	Myof	yes	–
		Integrin b1 (P: Integrin b3)	mys (Myospheriod)	ItgB1/3	yes	–
		Integrin a1, a3, a4, a5, a6, a7, a9, and av	if (Inflated)	ITGA4, ITGA5, ITGA6	yes	–
		Protein tyrosine kinase 2 (PTK2)	fak (Focal adhesion kinase)	Fak		
	Cell signalling	Brag2 (IQSEC)	siz (Schizo, a.k.a. loner)	IQSEC	yes	–
		ADP-ribosylation factor 6 (P: ARF1, 3, 4, & 5)	Arf51F (ADP ribosylation factor at 51F; Arf6)	Arf6	yes	–
		Dock1, Dock5	mbc (Myoblast city)	Dock1/5	–	
		Crk, Crkl	Crk (Crk oncogene)	Crk	yes	–
		Elmod1, Elmod2, Elmod3	Ced-12 (ELMO)	ELMOD	–	
		Rac1 (P: Rac2, Rac3)	Rac1, Rac2	Rac	yes	–
	Actin dynamics	–	blow (Blown fuse)	–	–	
		N-WASP (P:WASP)	WASp	WASP	yes	–
		WIPF	Wip (Vrp/Sltr)	WIPF	–	
		CYFIP1, CYFIP2	Sra-1	CYFIP	–	
		Nck-associated protein1 Nckap1 (P: Nckap1L)	hem (a.k.a. kette)	NCKAP	–	
		WASF1 (WASP family member 1) (P: WASF2, WASF3)	scar (Suppressor of cAMP Receptor)	WASF	yes	–
		ABI2	abi	ABI	–	
		Brk1	HSPC300	–	–	
		Arpc1a, Arpc1b	ArpC	Arp1	yes	–
		Arp2, Apr3, Arp4, Arp5	Arp66B	Arp2, Arp3, Arp4, Arp5	yes, all but Arp4	–
Regulation		Nuclear factor of activated T-cells cytoplasmic 2 (P: NFATC1, 3, 4, & NFAT5)	NFAT (NFAT nuclear factor)	Nfatc	yes	–
		Myocyte-specific enhancer factor 2A (P: MEF2B, C, & D)	Mef2	Mef2	yes	yes
		Myogenic Differentiation 1, Myogenin, Myogenic factor 5, & Myf 6	Nau (Nautilus)	MRF1, MRF2a, MRF2b, MRF3, & MRF4	yes, all but MRF4	–
		Paired box 3 & 7 (P: 2, 4, 5, 6, 8, 9)	prd (paired)	Pax3/7a, Pax3/7b	yes	–
		SIX homeobox 1 & 4 (P: 2, 3, 5, & 6)	sine oculis, Optix, Six4	Six1/2, Six3/6, & Six4/5	yes, Six1/2 & Six4/5	–

Because of the several lineages which diverged between flies and vertebrates in which only mononucleate, presumably unfused myocytes have been detected, including amphioxus, it is likely the two mechanisms of myoblast fusion arose convergently (Supplementary Fig. 1, Additional file 1). Initially the considerable conservation of the genetics of myoblast fusion between fly and vertebrate models was of interest to many and some suggested it could reflect two states derived from a shared ancestral mechanism [116]. With further work, the differences between fly myoblast fusion, occurring asymmetrically between a founder cell and FCMs resulting in myotubes with as many as a dozen nuclei, compared to fusion in vertebrates where similar myoblasts fuse to form, then fuse with, nascent myotubes resulting in myofibres with hundreds of nuclei, as well as the dependence on proteins thought to be novel to urochordates or vertebrates (e.g., MymK, MymX, JamB/C) suggest convergence [1]. Indeed, initial hypotheses of a single origin may have been misled by the fly-centred approach. While the origins of MymK and MymX have been traced to the base of the urochordates, the invertebrate chordate amphioxus is a more suitable outgroup to address the origin of vertebrate myoblast fusion, as it does not have multinucleate muscle cells, but is more closely related to vertebrates than flies, especially as vertebrate myoblast fusion is triggered not only in development, but also in muscle growth and regeneration. We now show that the Jam-B/C genes were in fact already present in the last common ancestor of chordates and expressed in muscles, as were the Netrin and Neogenin, and NFAT genes. Thus, the presence of a more vertebrate-like myogenic gene profile in the amphioxus muscles provides a more accurate proxy for the ancestral state of chordate muscles and the invertebrate precursor to the vertebrates than has thus far been obtained from study of more conventional (but phylogenetically more distant) model species, like *D. melanogaster*.

### Expected functions for genes differentially expressed in feeding-fasting experiment

**Fig. 1:**
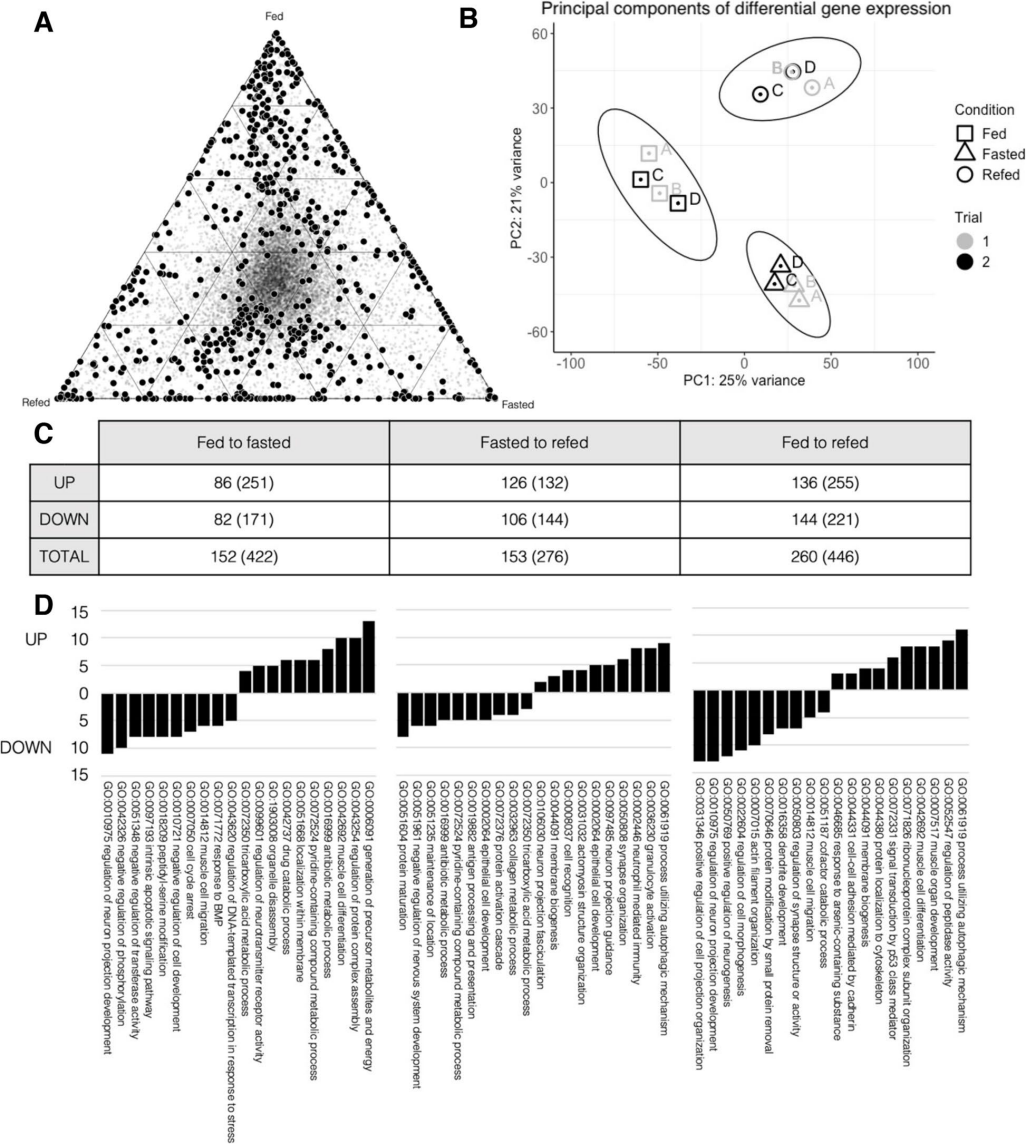
**A** Triangle plot of significantly (DESeq2: *p* < 0.1) DEGs (black circles) and non-DEGs (grey dots). Position denotes relative proportion of total expression between the three conditions so that genes upregulated in fed amphioxus are located towards the ‘Fed’ point of the triangle. **B** PCA plot with first and second primary components of variance of significantly DEGs (*n* = 795) separates the three treatment groups (shapes), without separating the two trials (colour). Each of the four individuals in a treatment group is labelled A, B, C, or D. **C** Number of genes and number of isotigs corresponding to that gene (in parentheses) significantly up- or down- regulated in three comparisons. Up-regulated from fed to fasted means significantly more reads were detected in the fasted amphioxus than in fed for that isotig. For some genes, some isotigs were up-regulated while others were down-regulated, hence a lower total number of genes than the sum of each direction. **D** Number of the significantly up- or down-regulated genes assigned to GO biological processes by WebGestalt analysis in the three comparisons

We then undertook a more targeted feeding-fasting-refeeding experiment of *B. lanceolatum* (see Materials and methods) and mapped the reads from amphioxus muscles sampled in each condition against our initial transcriptome for assembly and annotation. From our differential gene expression analysis, we found that 795 RNA isotigs were significantly differentially expressed between the three different treatment conditions (Fig. 1). There is a greater difference in gene expression between the fed state and fasted or refed states, suggesting that amphioxus may not have fully recovered after the final week of refeeding, especially as the fasting treatment took six weeks (see Materials and methods). The first principal component of the differential gene expression comprises 25% of the variance between the subjects, and clearly separates the fed treatment group from the fasted and refed groups along the x-axis of the PCA (Fig. 1B). This is also apparent from the triangle plot of the isotigs, where there appears to be a cluster of differentially expressed isotigs towards the ‘Fed’ vertex opposing a spread-out line distributed between ‘Fasted’ and ‘Refed’ at the bottom of the triangle (Fig. 1A). Despite the lengthy time it took for amphioxus to be deemed to be fasted due to prolonged retention of food in their gastrointestinal tract, as well as the potential weak recovery in gene expression, we still observe differential expression of many informative amphioxus genes.

The 795 differentially expressed isotigs correspond to a total of 401 human orthologues used for Gene Ontology (GO) annotation (Fig. 1C; 1D). These isotigs were aligned to the *B. lanceolatum* genome and annotated by comparison to known gene models. Human orthologues of these genes were used for functional annotation. Many of the isotigs mapped to intronic regions of the amphioxus genome, suggesting these could represent unspliced mRNAs or unannotated isoforms of gene models. In vertebrates, as quiescent muscle stem cells quickly differentiate during regeneration, this process is driven in part by the splicing of unprocessed mRNAs built up in the nucleus [142]. We detected that Dek (BL16094), the regulator of intron processing in this context, was expressed in our transcriptome, as well as some other genes involved in splicing (e.g., NCBP2: BL14925 and SNRPG: BL56599), but no orthologues of the markers of quiescence or proliferation were expressed. While this is potentially indicative of a splicing process involved in amphioxus muscle rebuilding and differentiation, it could also be an artefact of the incomplete gene models created by automatic annotation (e.g., FOXO is annotated as two separate genes, BL30356 and BL15802). The comparison of BLAT mapping coordinates against incomplete gene model locations, or gene models that do not represent all isoforms of a gene, would incorrectly yield intronic matches.

The GO gene set overrepresentation analysis shows that many of these genes are involved in autophagy (e.g. GO:0061919 process utilizing autophagic mechanism, GO:0052547 regulation of peptidase activity) as well as processes specific to muscles (e.g. GO:0031032 actomyosin structure organization, GO:0007517 muscle organ development, GO:0042692 muscle cell differentiation, GO:0014812 muscle cell migration). Other processes include metabolic responses (e.g. GO0006091 generation of precursor metabolites and energy, GO:0072350 tricarboxylic acid metabolic process), and signalling (GO:0043254 regulation of protein complex assembly, GO:0042326 negative regulation of phosphorylation, GO:0072331 signal transduction by p53 class mediator). These correspond to cellular components including the autophagosome (GO:0005776), endolysosome (GO:0036019, and GO:0031904 endolysosome lumen) as well as the structural component of muscle cells (e.g. GO0015629 actin cytoskeleton, GO0043292 contractile fiber). From our transcriptomic data, we see that the differentially expressed amphioxus genes are involved in similar processes observed in starved and refed vertebrate models, as expected.

Unexpectedly, several processes involved with neurogenesis were also overrepresented in the differentially expressed genes downregulated in fasted amphioxus (GO:0016358 dendrite development, GO:0050769 positive regulation of neurogenesis, GO:0010975 regulation of neuron projection development). In the fasted to refed comparison, neurogenic processes were upregulated (GO:0097485 neuron projection guidance, GO:0106030 neuron projection fasciculation) and from fed to refed, negative regulation of nervous system development is downregulated (GO:0051961) while neuron projection guidance is upregulated (GO:0097485). This indicates either that the neurogenic process is involved in amphioxus muscle growth, or that the two processes are regulated or conducted by similar mechanisms. This could also be artefactual, as the neurogenesis and neuron projection gene sets contain nearly 500 human genes (GO:0050769 has 447 genes; GO:0010975 has 475 genes), many of which overlap between the two sets. Our gene set contained twelve genes from GO:0050769, ten of which were also assigned to GO:0010975, and ten of the genes in either GO:0050769 or GO:0010975 were in GO:0031346 (positive regulation of cell projection organisation), indicating they may not have a function specific to neurogenesis. There is some signal, however, of a few neuron-specific genes being differentially expressed. Three genes involved in the cellular component GO:0031594 (neuromuscular junction), one of which is not found in any other GO category here (DLGAP4), while the other two are present in other categories including GO:0051668 (localisation within membrane; STX1B & LRP4). Amphioxus myogenesis undergoes a unique form of innervation during muscle growth, whereby nerve attachments form from the myotomal muscle fibres and grow to connect with the neural tube [ 147. , 148]. While the physiology of this process is somewhat understood, its genetic basis is still unknown. Perhaps a neurogenic program homologous to that of vertebrates is triggered in amphioxus myogenesis, despite differences in the process and physiology of muscle rebuilding between vertebrates and amphioxus. Also, some of the genes involved in myoblast fusion in only flies or vertebrates are found expressed in neurons, e.g., netA and fra in flies [81], and TANC1 and TANC2 in vertebrates [120], indicating there may be some overlap between neuron membrane proteins and the source of proteins co-opted into myoblast fusion.

Future work could determine if and how neurogenic protrusions in amphioxus occur during muscle development and growth.

**Fig. 2:**
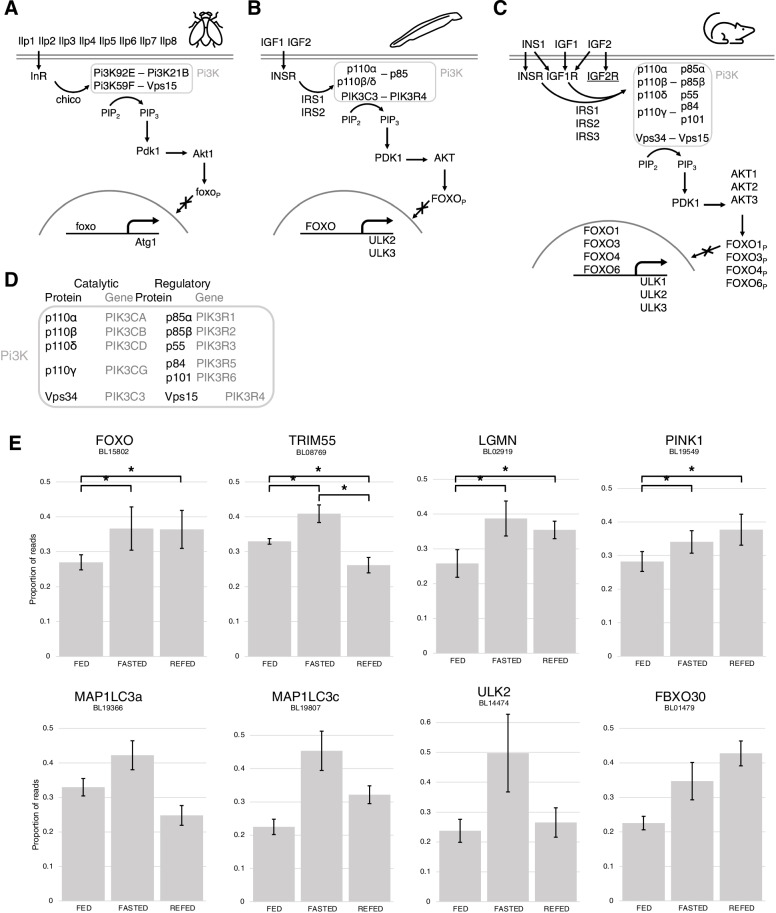
Schematic of the IGF/AKT/FOXO pathway in fruitfly (**A**), amphioxus (**B**), and mammals (**C**), and expression of FOXO-regulated genes in amphioxus (**E**). Insulin/IGF bind to their receptors, and IRS recruits Pi3K class I or III complexes to the membrane. Pi3K converts PIP_2_ to PIP_3_, which activates PDK, which phosphorylates AKT, which phosphorylates FOXO, inactivating it by preventing its entry to the nucleus. Without insulin, FOXO is not phosphorylated, and it can activate its target genes, including for example, Atrogin-1 (ULK family in chordates). **D** Key to gene names for protein subunits of Pi3K complex in mammals. **E** Proportion of overall normalised (by variance stabilisation in DESeq2) number of reads detected in each experimental condition for genes regulated by FOXO involved in autophagy [136]. Statistically significant differences in expression (*p* < 0.05, DESeq2 DGE analysis) are denoted by asterisks and brackets. Total number of reads are FOXO: 17,117; TRIM55: 297,158; LGMN: 71,123; PINK1: 226,008; MAP1LC3a: 5612; MAP1LC3c: 1049; ULK2: 25,482; FBXO30: 3212. Gene names reflect BLAST annotation and may not represent direct orthology. BL– numbers are B. lanceolatum gene model IDs. Error bars are the standard deviation of the mean across the four samples in each condition

KEGG pathways [55–57] which were overrepresented in our gene set included multiple signalling pathways, particularly the FOXO and Insulin signalling pathways (hsa04068 and hsa04910). FOXO itself was significantly differentially expressed between our treatments, as well as several of its known gene targets (Fig. 2E). BL08769 (TRIM54/55/63) is the amphioxus orthologue of the E3-ubiquitin ligases TRIM55, Murf1, and Murf2, which are upregulated in muscle atrophy and target myosin light chain components and troponin I [132, 149–151]. Legumain is an endopeptidase associated with the lysosome, regulated by FOXO [ 152. ]. The amphioxus PINK1 orthologue, BL19549, regulates the cellular response to oxidative stress when activated by FOXO (Mei et al., 2009). Other FOXO targets appear to be differentially expressed, though this difference was not statistically significant (Fig. 2E). Though there seem to be large differences in expression between the experimental states, these differences are not significant because of the lower overall read counts, fewer contigs for each gene, and less even expression between contigs (Fig. 2E, legend; Additional file 2). MAP1LC3a and MAP1LC3c (orthologous to yeast Atg8) have a role in the formation of the autophagosome [ 153. , 136]. ULK2 is involved in the initiation of autophagy [136], and FBXO30 is a paralogue of FBXO32, FBXO25 and FBXO40, all of which are E3 ubiquitin ligases related to atrogin-1. All of these genes play important roles in the regulation of autophagy and show changing levels of expression in line with elevated levels of FOXO expression and inferred activation of its target genes. This suggests that the homologous FOXO targets shared between vertebrates and flies may also be FOXO-regulated in amphioxus. This also illustrates the larger difference between the fed state, with lower FOXO expression, and both the fasted and refed states, with higher FOXO expression. In our refed animals, FOXO has nearly as high expression as in fasted animals, though this pattern could be further investigated with more robust quantitative methods. Still, this, coupled with the processes, components, and functions that were overrepresented in our gene set, shows the expected cellular response to starvation and indicates that amphioxus is responding similarly to the previously studied animal models, just at much slower rates, which is presumably due to a lower rate of metabolism and less active lifestyle than species such as flies and vertebrates. The observed response included the inactivation of the FOXO pathway, presumably allowing unphosphorylated FOXO to initiate the expression of its target genes for autophagy and muscle degradation, some of which we detected to be differentially expressed in line with FOXO’s detected expression. Further experiments could confirm the conserved role of FOXO in amphioxus but our findings here at least suggest that amphioxus would make an informative comparative model to understand the evolution of the genetics of chordate muscle metabolism.

### The Ins/Akt/FOXO pathway is highly conserved amid gene duplications in several lineages

**Fig. 3:**
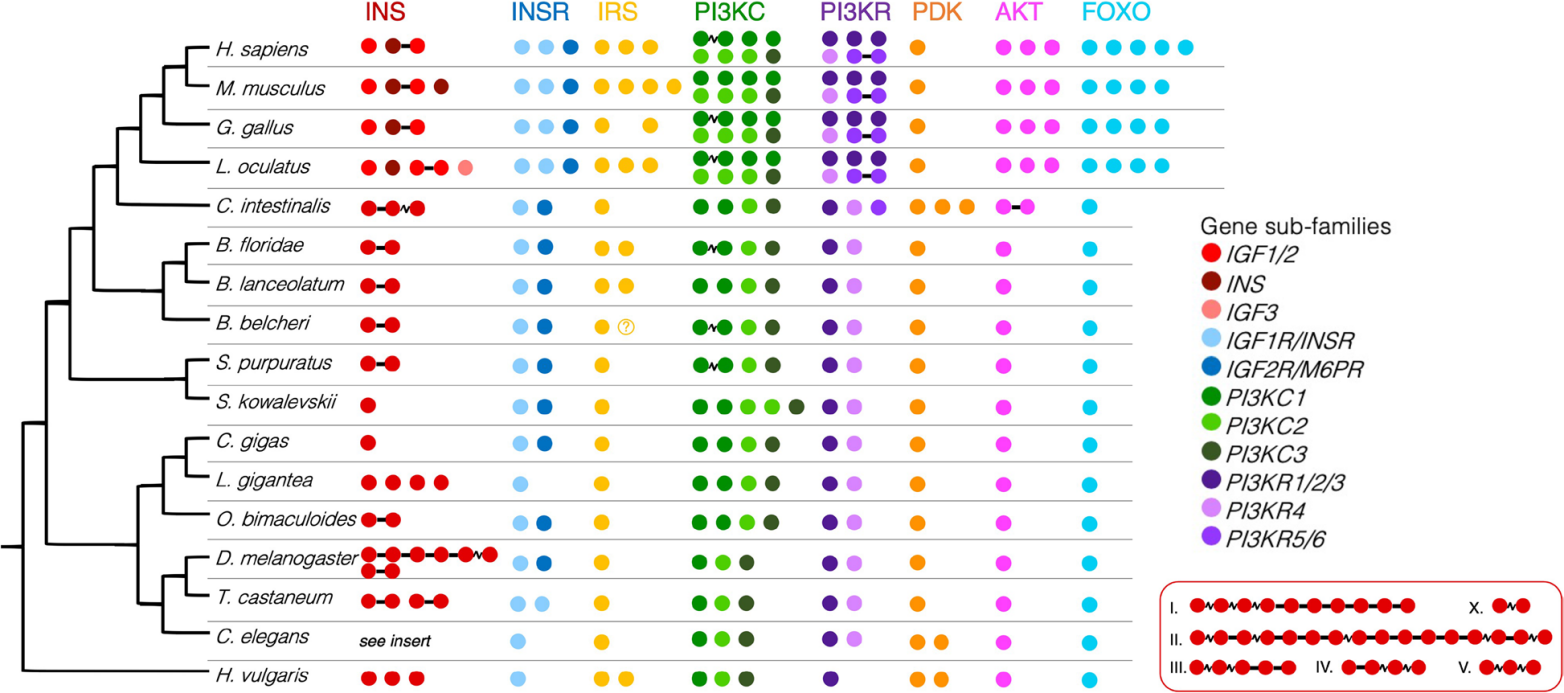
Schematic of genes for proteins in the IGF/AKT/ FOXO pathway in the 17 species included in this study. Clustered adjacent genes are joined with black lines, while genes linked but over longer distances are joined with a jagged line. Question mark denotes uncertainty if the gene is lost/gained. The B. belcheri IRS2 could not be located, but may not have been sequenced. The 40 C. elegans ILPs are shown in the red box to the right

Our analysis of the FOXO pathway in amphioxus and selected metazoans shows that the genes for components of the pathway are highly conserved (Fig. 2). Genes for proteins in each step of the core pathway (based on KEGG map04068) were identified in all of our study species, including the hydroid, two insects, a nematode, three molluscs, an echinoderm and a hemichordate, as well as a urochordate, three amphioxus species, and four vertebrates: gar, chicken, mouse, and human (Fig. 3). This shows the remarkable conservation of this pathway in animals and its likely origin in the metazoan ancestor.

We also validated amphioxus pathway components and detected transcripts for amphioxus orthologues of IGF, IGFR, IRS, PDPK, Akt, and FOXO in our muscle transcriptome (Additional file 3), though only FOXO was differentially expressed. Though expression of amphioxus Pi3K complex component genes were not detected, this could be due to their permanent presence in muscle cells that does not change in response to nutrient availability. Furthermore, the lack of any significant differential expression of genes for most components of the pathway is expected, since their response to nutrient availability is primarily via activation or inactivation by processes such as phosphorylation, not levels of expression (see Introduction). Though the functions of these amphioxus proteins have yet to be determined, our results suggest this pathway is conserved in amphioxus and our identifications provide a foundation for future work addressing the evolution of these genes, particularly in comparison to vertebrate models.

It is evident from this reconstruction that for certain genes, the 2R WGD had a large impact on the number of paralogues in vertebrates, while for other genes, vertebrates have no extra paralogues, and some genes expanded independently in different lineages. The most marked example of expansions in invertebrates occurs amongst the insulin and insulin-like growth factors, where several lineages have multiple clusters of ILPs that likely originated via serial tandem duplications. These genes may have been retained in duplicate because of subfunctionalisation, as the eight dILPs are expressed in different contexts [ 154. ], but for a gene like insulin, it may instead have been advantageous to have multiple paralogues to increase gene production. If multiple paralogues have subfunctionalised in invertebrates, for instance to be expressed in different tissues or at different stages of growth, these signals are all received by a single receptor (except *Drosophila* ILPs7 and 8, which activate a separate leucine-rich repeat G-protein coupled receptor, similarly to vertebrate relaxins, in the larger insulin-like gene superfamily [ 155. ]). In contrast, the diversification of the vertebrate insulin/IGF family corresponds to an equal increase in the number of receptors, presumably because IGF1 and IGF2, as well as INSR and IGF1R arose simultaneously due to the 2R WGD. The vertebrate IGF2R is a cation-independent mannose-6-phosphate receptor, paralogous to the cation-dependent M6PR, and orthologous to CI-M6PR in invertebrates, which does not bind invertebrate ILPs or vertebrate insulin, and is not homologous to the INSR/IGF1R genes [13, 35]. IGF2R (CI-M6PR) contains 15 repeats of a domain that CD-M6PR contains only one of, and it is these additional domains that confer binding to IGF2 [35]. Still unclear is the relationship of the two amphioxus and tunicate IGFs and the two receptors, as it seems that while invertebrate IGF2R orthologues, CI-M6PRs (called lerp in fruit fly) are present, they do not bind any of the invertebrate ILPs [42].

Also notable is the genomic location of the insulin family genes. As already noted, the invertebrate insulin paralogues tend to sit in clusters; five of the eight fruit fly ILPs are found clustered on chromosome 3 L, and two of the remaining three lie adjacent on the X chromosome (Fig. 3). The 40 nematode ILPs are likewise found in several clusters (Fig. 3 insert). Also, the two INS/IGF genes in the octopus, urchin, and in amphioxus are situated in tandem, potentially orthologous to the cluster of INS and IGF2 in vertebrates (Fig. 3). The phylogeny, however, suggests that the two-gene clusters in octopus, amphioxus, urchin, the three linked genes in the tunicate, and the IGF2-INS cluster in vertebrates all arose separately, as these genes are separated by speciation nodes and the vertebrate clade is monophyletic (Additional file 1). Alternatively, this topology could also have arisen via divergent selection on these genes within each species, resulting in sequences that are more similar between paralogues than orthologues, despite the duplication predating the speciation of these groups.

### Codiversification of the Pi3K complex subunits

**Fig. 4:**
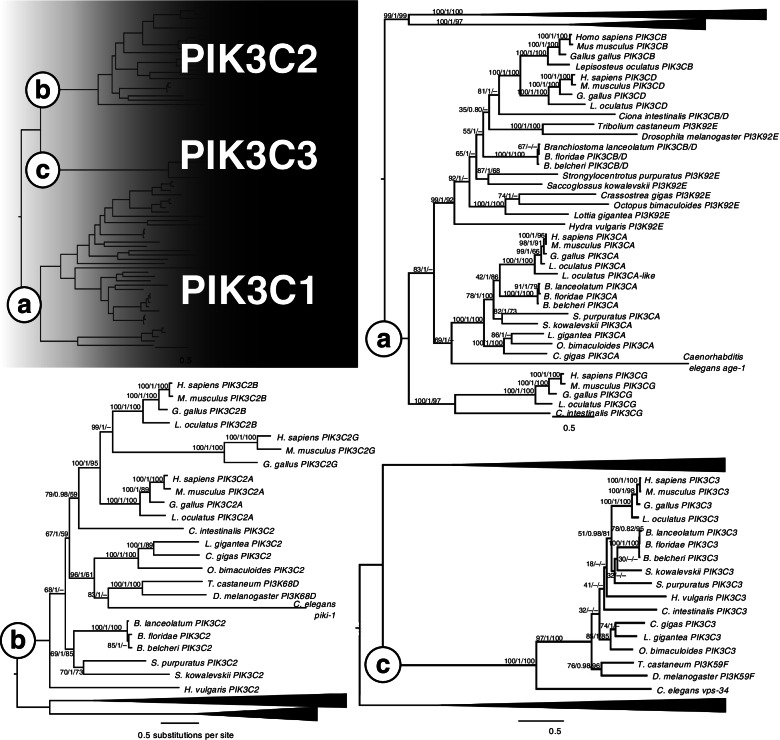
Maximum likelihood phylogeny of Pi3K catalytic subunits. **a** Subtree of Class I subunits, with Class II and III subtrees collapsed. **b** Class II subunits and **c**) Class III subunits. Support values are IQ-TREE bootstrap support (% of 1000 replicates), MrBayes posterior probability, and MEGA Neighbor-Joining bootstrap support (% of 1000 replicates), separated by slashes. Dashes denote missing support values for branches not present in that tree-building method

How function relates to paralogue retention is exemplified by the Pi3K complex. This kinase has roles in numerous cell processes, and with its different binding partners determined by the makeup of its subunits, it has an immensely complex set of roles [ 156. – 158. ]. There are three classes of Pi3K (Fig. 2D), which phosphorylate different phosphatidylinositols, distinguishing their roles in different signalling pathways [ 159. ]. The catalytic domains share homology within and between classes, and show that animals have three gene types, corresponding to the three classes of kinase (Fig. 4). While the class III catalytic domain remained in the single copy in vertebrates (Fig. 4c), there are three vertebrate paralogues of the class II catalytic domain corresponding to one invertebrate gene (Fig. 4b), and four vertebrate paralogues of the class I catalytic domain orthologous to two invertebrate genes (Fig. 4a). The 2R WGD likely generated the class II paralogues, as the vertebrate clade is monophyletic (Fig. 4c; vertebrate clade support 100/1/95). For the class I vertebrate paralogues, one invertebrate gene (lost in insects) is orthologous to the PIK3CA gene in vertebrates, while the other (lost in hydroid and nematode) is orthologous to the vertebrate PIK3CB and PIK3CD genes (Fig. 4a; PIK3CA clade support 99/1/−; PIK3CB/D clade support 100/1/92).

**Fig. 5:**
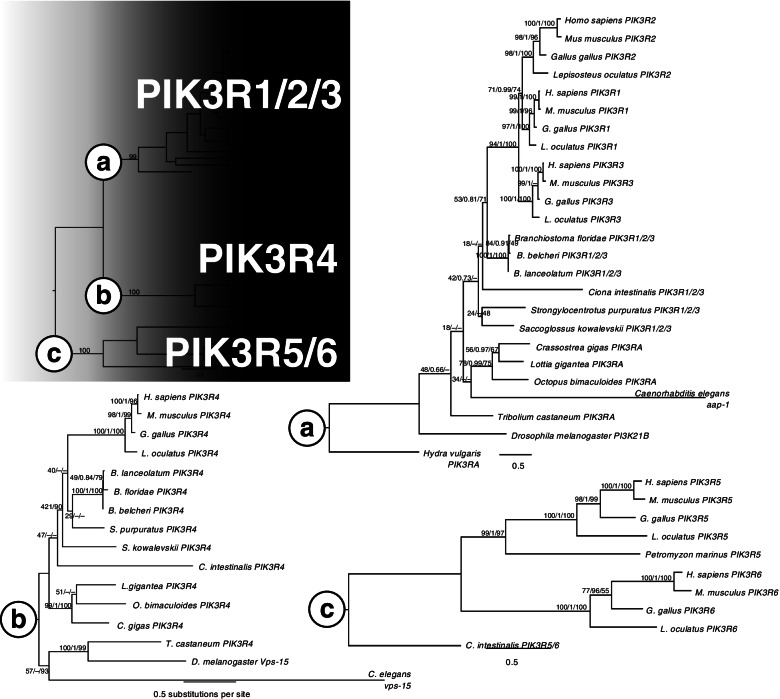
Maximum likelihood phylogeny of Pi3K regulatory subunits. **a** Phylogeny of Class I subunits, **b** Class III subunits, and **c** Olfactores-specific Class I type b subunits. Support values are IQ-TREE bootstrap support (% of 1000 replicates), MrBayes posterior probability, and MEGA Neighbor-Joining bootstrap support (% of 1000 replicates), separated by slashes. Dashes denote missing support values for branches not present in that tree-building method. Support values for phylogeny of all subunit types (top left) is IQ-TREE consensus ML values. Phylogenies for each class were created separately to determine finer-scale topology due to low levels of similarity between the different classes

There is little similarity between the three classes of regulatory subunits. The class IA regulatory subunit has three vertebrate paralogues, corresponding to a single invertebrate gene present in all species in the study (Fig. 5a; vertebrate clade support 94/1/100). There is also an Olfactores-specific gain of a gene type, the class IB subunit, PIK3R5/6, with a single orthologue in the tunicate and two linked genes in vertebrates (Fig. 5c; Fig. 3). This corresponds to the Olfactores-specific gain of its partner, PIK3CG (Fig. 4a; PIK3CG clade support 100/1/97; Fig. 3), suggesting these two proteins arose together, and the PIK3R5/6 precursor duplicated in tandem after the tunicates diverged. Class II does not have a regulatory subunit, while class III has a single gene in both invertebrates and vertebrates, PIK3R4 (Fig. 5b), similar to the single copy class III catalytic subunit (Fig. 4b).

The consistent patterns of gene numbers between the catalytic and regulatory subunits within each class suggests the codiversification of these proteins and is presumably linked to their conjoined function (Fig. 2D). For these genes, the mode of subfunctionalisation appears to have been sequence-based rather than in the regulatory regions, especially as these genes are generally ubiquitously expressed. While some subunits were not retained in duplicate following 2R, e.g. the class III partner genes PIK3C3 and PIK3R4, the class I complex diversified, resulting in multiple vertebrate paralogues of the catalytic (PIK3CB and PIK3CD) and regulatory (PIK3R1, PIK3R2, and PIK3R3) subunits with an origin in the 2R WGD. The 2R origin of these paralogues is supported by synteny as well, as PIK3CB and PIK3CD are in distinct regions of the vertebrate genomes corresponding to the same ancestral chordate linkage group, P [111]. There is a similar pattern with PIK3R1, PIK3R2, and PIK3R3 in group L, as well as the three class II catalytic domains PIK3C2A, PIK3C2B, PIK3C2G and group O (Additional file 3). Further strengthening the evidence for the role of 2R in the origin of these paralogues is that the amphioxus pro-orthologues of these genes reside in the same linkage groups, with PIK3CB/D in P, PIK3R1/2/3 in L, and PIK3C2 in O (Additional file 3).

The codiversification of these paralogues after the 2R WGD, where genes for interacting proteins were retained together, is consistent with retention by dosage-compensation, which then allows time for functional divergence to occur between paralogues [ 160. ,  161. ]. Furthermore, the highly conserved nature of the catalytic subunits compared to the divergence of the regulatory subunits may be consistent with the lynchpin hypothesis, where pathways gain complexity as small changes to redundant protein sequences can change paralogous proteins’ interactors or substrates, and quickly increase complexity [ 162. ]. In this case, the catalytic domain is constrained to an enzymatic function (PIP phosphorylation), while the regulatory domain is relatively free to diverge and evolve different domains, allowing complexing with different binding partners in different contexts. It would be of interest to determine the reasons certain Pi3Ks returned to the single copy while others were retained in duplicate, especially as these kinases constitute central positions in myriad signalling pathways in nearly every cell. To address this question, we suggest future studies use a suitable preduplicate outgroup as a proxy for the ancestral state such as amphioxus, and our identification of the amphioxus orthologues may provide a good starting point for this future work.

### Evolution of FOXO transcription factors: the impact of 2R

**Fig. 6:**
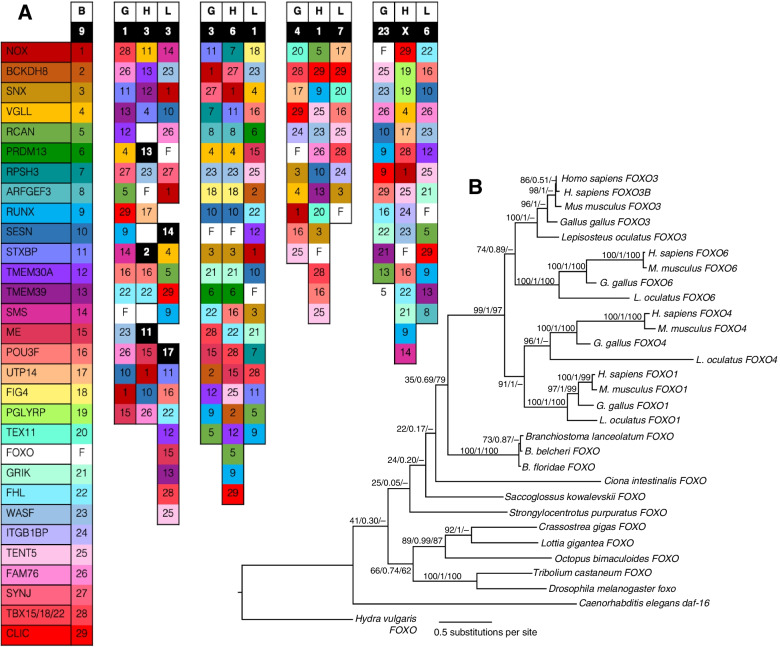
**A** Synteny of the FOXO locus in amphioxus (B), chicken (G), human (H) and spotted gar (L). Genes are represented by boxes coloured and numbered by each 2R WGD family on chromosomes labelled with white text in black boxes. Each gene family has a single gene on amphioxus chromosome 9, and up to four paralogues in vertebrate genomes, e.g., the FOXO ohnologues on chicken chromosome 3 and human chromosome 6 are both adjacent to an ohnologue from the SESN (10) family. Distances are not to scale, and gene order does not exclude intervening genes. Detailed locations are listed in the supplementary information. **B** Maximum likelihood phylogeny of metazoan FOXO genes. Support values are IQ-TREE bootstrap support (% of 1000 replicates), MrBayes posterior probability, and MEGA Neighbor-Joining bootstrap support (% of 1000 replicates). Dashes denote missing support values for branches not present in that tree-building method. Alignment was made using MAFFT (supplementary information)

For the FOXO family, 2R is the primary duplication event detected. The phylogeny of these genes shows that each invertebrate gene is orthologous to the four vertebrate paralogues (Fig. 6B, vertebrate clade support: 99/1/97), and these four vertebrate paralogues sit in four distinct paralogous regions in the vertebrate genomes (Fig. 6A). The expected “one-to-four” orthology between a single amphioxus FOXO locus, located on chromosome 9 in the new *B. floridae* genome assembly [111], and four vertebrate loci in distinct chromosomal locations is consistent with the origin of the four FOXO paralogues in the 2R WGD. While many paralogues were lost across these loci, the resultant pattern illustrates the four-fold paralogy of vertebrate genomes [23]. The human FOXO3B is not shared with any other vertebrates here, and groups with the human FOXO3 in the phylogeny, suggesting it is a recent, human-specific duplication (N.B. its name does not represent orthology to teleost fish FOXO3b genes that arose in the teleost-specific 3R WGD). As such, it represents an intriguing human-specific elaboration of this widely conserved pathway.

Regulatory genes, especially transcription factors with complex regulatory regions themselves tend to be overrepresented in paralogues retained in duplicate following WGDs. This is because of their propensity to subfunctionalise via the Duplication-Degeneration-Complementation process, where duplicated genes accrue degenerate mutations in their regulatory regions, partitioning their expression so that each is expressed at a subset of the ancestral genes’ expression, and thus both are required to fulfil that ancestral role [33]. For the FOXO genes, there is some evidence of subfunctionalisation amongst the vertebrate paralogues, as they are expressed differentially [ 163. ,  164. ]. While FOXO1 knockout mutants are lethal, FOXO4 mutants have no phenotype and FOXO3 mutants show reproductive abnormalities [ 165. ], while FOXO6 is expressed in the developing brain and liver [ 166. ]. FOXO1 is required for myoblast fusion in development [ 167. ], but FOXO3 is involved in muscle nutritional response and mitophagy [136]. Further work on FOXO in amphioxus could begin to characterise the ancestral function and provide a point of comparison to understand the subfunctionalisation of vertebrate FOXO paralogues. Understanding how these genes evolved will also help understand the impact of 2R WGD on the evolution of vertebrate complexity.

## Conclusions

All along this highly conserved pathway, it is clear that duplication has led to increasing complexity, especially in the case of the vertebrates. The 2R WGD created redundant genes duplicated in the context of their entire regulatory region, important for genes with complex regulation like FOXO transcription factors, as well as simultaneous duplication of entire networks, important for protein complexes with multiple subunits like Pi3K. Comparisons to amphioxus show that orthologues of many of the vertebrate genes involved in the more complex muscle building processes, including myoblast fusion, were present in the chordate ancestor. We suggest that perhaps it was the 2R WGD that allowed this process to fully develop into vertebrate myogenesis, possibly alongside key genetic innovations such as Myomaker and Myomixer, which are the only Olfactores-specific myogenic genes that are now distinguishable following our analysis of myoblast fusion candidate genes in the amphioxus muscle transcriptome data. Our study suggests a convergent rather than orthologous origin of multinucleate myocytes in flies and vertebrates, since amphioxus possesses a vertebrate-like set of myoblast fusion genes, yet myoblast fusion has not been detected in amphioxus. We also detected a complete INS/Akt/FOXO pathway in amphioxus and show that it is involved in the response to nutritional limitation similar to other well-studied invertebrate and vertebrate models. Thus, amphioxus may be a useful model organism to understand the evolution of vertebrate muscle physiology and disease. Here, our use of amphioxus as a more appropriate and phylogenetically better-placed pre-duplicate comparison to the vertebrates is key to assessing the impact of 2R WGD and detecting vertebrate-specific novelties of muscle evolution and development.

## Materials and methods

### Amphioxus transcriptome

**Fig. 7:**
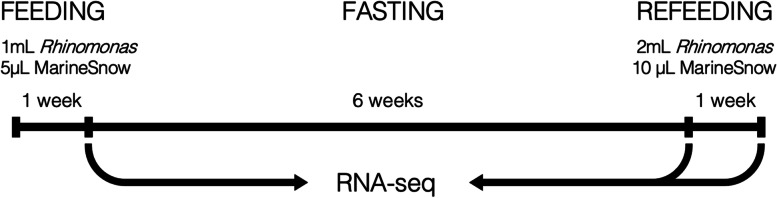
Schematic of the eight-week trial of the amphioxus feeding-fasting-refeeding experiment with three sample timepoints

European amphioxus (*B. lanceolatum*) were obtained from the Plymouth Marine Laboratory (Plymouth, UK) and maintained in the aquarium facilities of the Scottish Oceans Institute (SOI) in St Andrews (Scotland, UK). Amphioxus were fed with a diet of the red algae *Rhinomonas reticulata*, visible in the amphioxus gut, and supplemented with MarineSnow (Two Little Fishies, Inc), fasted for six weeks, then refed before sampling at the eighth week (Fig. 7). To generate the *de novo* transcriptome, 21 amphioxus muscle RNA samples were pooled, from 7 fed, 8 fasted, and 6 refed animals. Total RNA was extracted from dissected amphioxus muscle and immersed in TRIsure, then homogenised. After centrifugation, the aqueous phase was isolated, and RNA was precipitated with isopropanol, recentrifuged, and isolated in the pellet. Samples were sent for Roche 454 pyrosequencing at TGAC (The Genome Analysis Centre, which has now been renamed to the Earlham Institute). Reads were assembled with Newbler v.2.6 and annotated with BLASTx searches against the nr NCBI database in BLAST2GO with an e-value cutoff of 10^−3^ to create the *de novo* amphioxus muscle transcriptome. Statistics of the assembly are presented in Additional file 1.

### Feeding-fasting-refeeding experiment

For the differential gene expression experiment, twelve amphioxus were kept at ambient temperature over the course of two eight-week trials. Each trial consisted of a feeding period of a week, followed by a 43-day fasting period allowing amphioxus to clear their guts completely, and a one-week refeeding period (Fig. 7). At each time point in each trial, two amphioxus were sampled, their muscles dissected, and RNA extracted. These samples were sent for paired-end Illumina Hiseq2000 flow cell sequencing at TGAC. Illumina reads were mapped against the 454 *de novo* transcriptome with SOAP [138] and reads were quantified with RSEM [64]. Differential gene expression between the three experimental conditions was compared with DESeq2 [67] with a p-value alpha of 0.1.

### Differential gene expression and functional annotation

Differentially expressed isotigs (*n* = 795) were functionally annotated by mapping against the *B. lanceolatum* genome assembly [71]. Mapping was compared to *B. lanceolatum* gene models with GFFCompare [95] for a list of 476 *B. lanceolatum* genes from 664 isotigs with good mapping hits corresponding to a *B. lanceolatum* gene model. These were BLAST searched against the human proteome (UniProt UP000005640) to find human orthologues. A list of human protein IDs for genes whose amphioxus orthologues were differentially expressed in each treatment comparison was submitted to WebGestalt [66] for functional annotation and overrepresentation analysis. For each comparison, up- and down- regulated genes were compared to the GO biological process, GO molecular function, GO cellular component, and KEGG pathway databases against the human genome reference set. The top 10 gene sets were retrieved, and can be found in Additional file 1.

### INS/Akt/FOXO pathway bioinformatics

The canonical INS/Akt/FOXO pathway was determined from the KEGG pathway (map04068; FoxO signalling pathway) and a survey of the literature (e.g., [3, 72, 123]). Vertebrate protein sequences were taken from Ensembl [143], and most invertebrate sequences were taken from Ensembl Metazoa or UniProt [125], and checked against specific databases including EchinoBase [60], ANISEED [14], BeetleBase [133], FlyBase [126], and WormBase [43]. All sequences were checked via BLAST against the genome assemblies available, and their CDS locations recorded. Amphioxus sequences were taken from NCBI. Sequences and respective genomic locations can be found in Additional file 3. Sequences were aligned in Jalview [135] so that gene models for some species could be manually curated. Final alignments were made using MAFFT [58] with the preset ENS-I and were not manually curated. Alignments can be found in Additional file 4.

### Phylogenetics

Alignments were first submitted to the IQ-TREE web server [88, 128] for model testing [53] and Maximum Likelihood phylogeny building with 1000 ultrafast bootstrap replicates [45]. Consensus support values on the consensus ML tree were used to make the figures, and branches were annotated with support values from Bayesian Inference and Neighbor Joining. Maximum Likelihood phylogenies for PIK3C, PIK3R, and FOXO alignments were subsequently made with 1000 real bootstrap replicates in IQ-TREE which are presented in the figures. This model or the closest equivalent was used in MEGAX [61] to create the NJ phylogeny. Bayesian phylogenies were made using MrBayes v3.2.7 [104] on the CIPRES Science Gateway [80]. The model was set to mixed, so as to allow switching between models during the analysis. It was run for a maximum of 5x10^8^ generations, printing trees every 5x10^5^ generations and sampling every 5x10^4^ generations, but with a stop rule for convergence of the average standard deviation of split frequencies = 0.01. MCMC output was analysed in Tracer v1.7.1 [101] to determine appropriate burn-in between runs, and consensus trees were made with TreeAnnotator (TreeAnnotator) 127. Trees were visualised in FigTree v1.4.4 (FigTree) 30. Phylogenies can be found in Additional file 1.

### Synteny

Synteny of the FOXO locus began with the paralogy groups created by Simakov et al. [111]. Based on the genomic location in gar and chicken genomes, the ancestral chordate FOXO locus was determined to be in chordate linkage group K. This corresponds to previous chordate linkage group 17 from Srivastava et al. [117]. Locations of human and gar orthologues of chicken genes in this group were retrieved with BioMart [112], and this list was reduced to include only gene families with multiple but no more than five paralogues across four FOXO loci and with orthologues on the FOXO-bearing chromosome in amphioxus, using *B. lanceolatum* gene models mapped to chromosome 9 in the new *B. floridae* chromosome-level assembly [111]. Details of synteny can be found in Additional file 3.

## Supplementary Information

**Additional file 1.**

**Additional file 2.**

**Additional file 3.**

**Additional file 4.**

## Data Availability

The datasets supporting the conclusions of this article are included within the article and its additional files. This Transcriptome Shotgun Assembly project has been deposited at DDBJ/ENA/GenBank under the accession GJID00000000 (https://www.ncbi.nlm.nih.gov/nuccore/GJID00000000), which is publicly open. The version described in this paper is the first version, GJID01000000.
