# Supplementary material for: Amphioxus muscle transcriptomes reveal vertebrate-like myoblast fusion genes and a highly conserved role of insulin signalling in the metabolism of muscle
Source: BMC Genomics. 2022 Feb 1;23:93. doi: 10.1186/s12864-021-08222-9 (PMC8805411; doi:10.1186/s12864-021-08222-9)
Supplement: Supplementary file 1 — Additional file 1. [file 12864_2021_8222_MOESM1_ESM.docx]

**This file’s contents:**

Additional file 1

1. *B. lanceolatum* transcriptome
   1. Statistics
2. Supplementary figure 1: Diagram of multinucleate muscles across metazoan cladogram
3. Supplementary table 1: Differential gene expression annotation
   1. GO cellular components
   2. GO molecular functions
   3. KEGG pathways
4. INS/IGF phylogenies
   1. ML
      1. Supplementary figure 2: Trimmed
      2. Supplementary figure 3: Full length, more vertebrates
   2. BI
      1. Supplementary figure 4: Trimmed
   3. NJ
      1. Supplementary figure 5: Trimmed
5. PI3KC phylogenies
   1. Supplementary figure 6: ML
   2. Supplementary figure 7: BI
   3. Supplementary figure 8: NJ
6. PI3KR phylogenies
   1. ML
      1. Supplementary figure 9: PIK3R1/2/3
      2. Supplementary figure 10: PIK3R4
      3. Supplementary figure 11: PIK3R5/6
   2. BI
      1. Supplementary figure 12: PIK3R1/2/3
      2. Supplementary figure 13: PIK3R4
      3. Supplementary figure 14: PIK3R5/6
   3. NJ
      1. Supplementary figure 15: PIK3R1/2/3
      2. Supplementary figure 16: PIK3R4
      3. Supplementary figure 17: PIK3R5/6
7. FOXO phylogenies
   1. Supplementary figure 18: ML
   2. Supplementary figure 19: BI
      Supplementary figure 20: NJ
8. Supplementary table 2: Myoblast fusion genes
   1. Expansion of main text Table 1
   2. References

**Other supplementary files**

Additional file 2

Supplementary table 3: FOXO target gene expression

1. FOXO
2. TRIM55
3. LGMN
4. PINK1
5. MAP1LC3a
6. MAP1LC3c
7. ULK2
8. FBXO30

Additional file 3

Supplementary table 4: FOXO pathway gene accessions and locations

1. FOXO pathway gene accessions and locations
2. Amphioxus transcripts of FOXO pathway genes

Supplementary table 5: Synteny: ancestral chordate linkage groups for Pi3Ks

Supplementary table 6: Synteny of FOXO neighbourhoods in vertebrate and amphioxus genomes

Additional file 4: Alignments (PFAM format)

1. INS/IGF
   1. Trimmed
   2. Full length, more vertebrates
2. INSR/IGF1R
3. IGF2R
   1. IGF2R
   2. Additional M6PR sequences
4. IRS
5. PIK3C
6. PIK3R
   1. All
   2. PIK3R1/2/3
   3. PIK3R4
   4. PIK3R5/6
7. PDK
8. AKT
9. FOXO
10. *B. lanceolatum* transcriptome
    1. Muscle transcriptome sequenced on Roche 454. Reads were assembled with Newbler v2.6 (20110517_1502). Date of Assembly: 2012/06/27 14:06:19
    2. Transcriptome sequences were submitted to GenBank under the BioProject identifier PRJNA753289 with accession GJID00000000.
    3. Statistics
       1. Reads
          1. Total number 561905
          2. Aligned 411155
          3. Assembled 355725
          4. Partial 55528
          5. Singleton 81008
          6. Repeat 49506
          7. Outlier 17877
          8. Too short 2480
       2. Isotigs
          1. Total number 14854
          2. Average contig count 3.9
          3. Largest contig count 18
          4. Number with 1 contig 5328
          5. Average isotig size 1643
          6. N50 isotig size 2292
          7. Largest isotig size 10480
       3. Isogroups
          1. Total number 7352
          2. Average contig count 2.9
          3. Largest contig count 18
          4. Number with 1 contig 5328
          5. Average isotig count 2.0
          6. Largest isotig count 99
          7. Number with 1 isotig 5366
11. Supplementary figure 1: Diagram of multinucleate muscles across a metazoan phylogeny. Species and lower classifications are joined by a cladogram representing their relationships. The presence (star) or absence (dash) of multinucleate myofibres is indicated for those species and, if present, the muscle type where these have been reported is described. Further references (to complement main text references) are included.
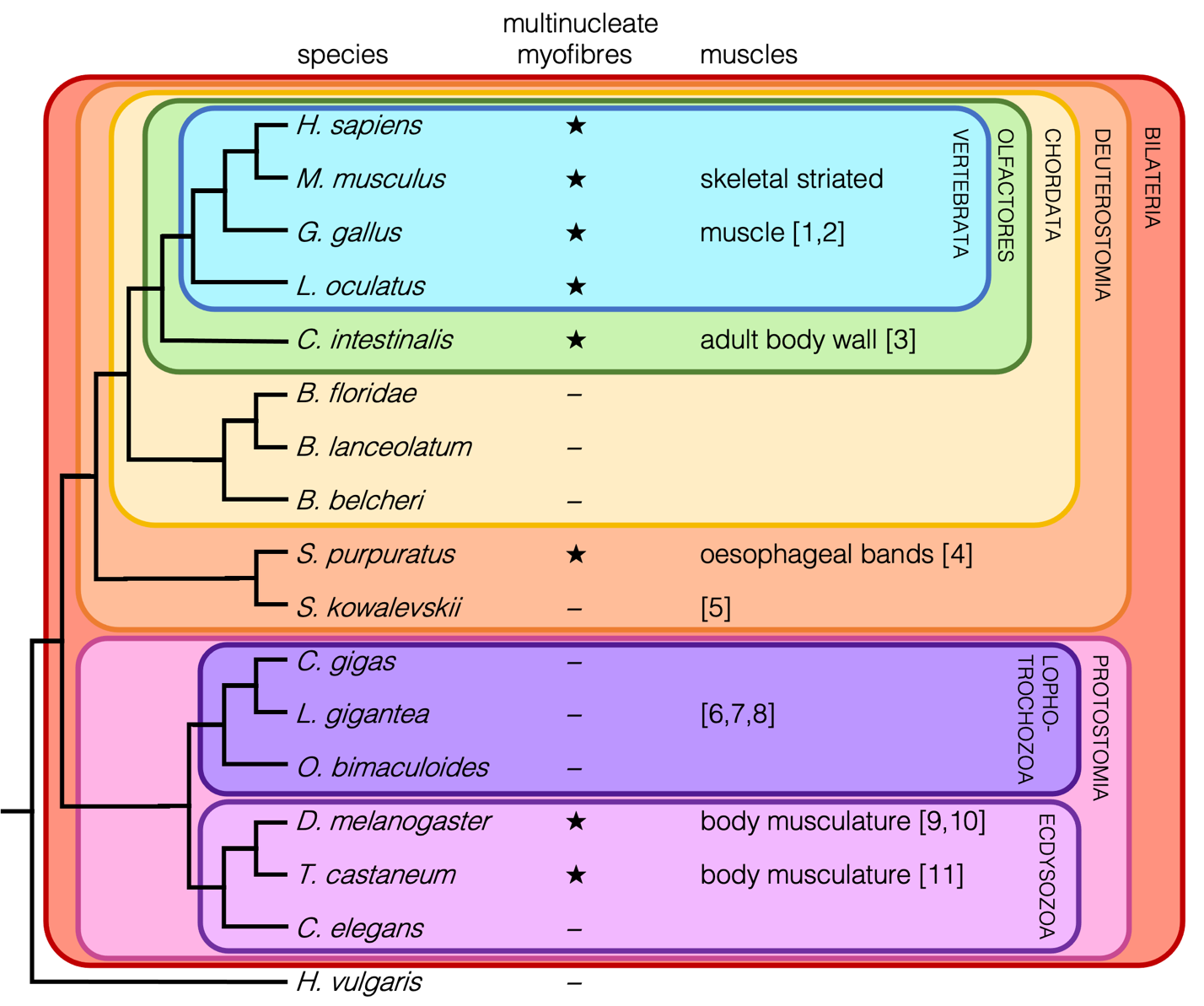


1. Hilfer SR, Searls RL, Fonte VG. An ultrastructural study of early myogenesis in the chick wing bud. *Dev Biol*. 1973;30:374–91.

2. Buckingham M. Myogenic progenitor cells and skeletal myogenesis in vertebrates. *Curr Opin Genet Dev*. 2006;16:525–32.

3. ﻿Razy-Krajka F, Stolfi A. Regulation and evolution of muscle development in tunicates. *Evodevo*. 2019;10:1–34.

4. ﻿Andrikou C, Iovene E, Rizzo F, Oliveri P, Arnone MI. Myogenesis in the sea urchin embryo: the molecular fingerprint of the myoblast precursors. *Evodevo*. 2013;4:33.

5. Ruppert EE. Key characters uniting hemichordates and chordates: homologies or homoplasies? *Can J Zool*. 2005;83:8–23.

6. ﻿Kurita Y, Hashimoto N, Wada H. Evolution of the molluscan body plan: the case of the anterior adductor muscle of bivalves. *Biol J Linn Soc*. 2016;119:420–9.

7. ﻿Dyachuk V, Odintsova N. Development of the larval muscle system in the mussel *Mytilus trossulus* (Mollusca, Bivalvia). *Dev Growth Differ*. 2009;51:69–79.

8. ﻿Zullo L, Fossati SM, Imperadore P, Nödl MT. Molecular determinants of cephalopod muscles and their implication in muscle regeneration. *Front Cell Dev Biol*. 2017;5:53.

9. ﻿Dworak HA, Sink H. Myoblast fusion in Drosophila. *BioEssays*. 2002;24:591–601.

10. ﻿Gunage RD, Dhanyasi N, Reichert H, VijayRaghavan K. Drosophila adult muscle development and regeneration. *Semin Cell Dev Biol*. 2017;72:56–66.

11. ﻿Schultheis D, Weißkopf M, Schaub C, Ansari S, Dao VA, Grossmann D, et al. A large scale systemic RNAi screen in the red flour beetle Tribolium castaneum identifies novel genes involved in insect muscle development. *G3 Genes, Genomes, Genet*. 2019;9:1009–26.

1. Supplementary table 1: Differential gene expression annotation. The top ten GO terms for (a) cellular components and (b) molecular functions, as well as (c) KEGG pathways that we found were overrepresented in our gene sets from WebGestalt. Each set represents down- or upregulated genes from our differential gene expression analysis for the three different comparisons between the three treatments, fed, fasted, and refed. The number of genes from a gene set belonging to that term is listed, as well as the overall number of genes associated with that term.
   1. GO cellular components

| Comparison | Directionality | GO terms | Genes | GeneSet Size |
| --- | --- | --- | --- | --- |
| Fed-Fasted | Down | GO:0015629 actin cytoskeleton  GO:0031252 cell leading edge  GO:0030055 cell-substrate junction  GO:0043292 contractile fiber  GO:0005667 transcription factor complex  GO:0031253 cell projection membrane  GO:0005770 late endosome  GO:0016605 PML body  GO:0005905 clathrin-coated pit  GO:0031904 endosome lumen | 12  9  6  6  5  5  4  3  2  2 | 482  393  411  225  355  335  242  99  67  36 |
|  | Up | GO:0015629 actin cytoskeleton  GO:0043292 contractile fiber  GO:0101002 ficolin-1-rich granule  GO:1904949 ATPase complex  GO:0016528 sarcoplasm  GO:0031594 neuromuscular junction  GO:0005844 polysome  GO:0000118 histone deacetylase complex  GO:0005790smooth endoplasmic reticulum  GO:0000791 euchromatin | 11  9  6  4  3  3  3  3  2  2 | 482  225  183  102  77  76  73  60  34  33 |
| Fasted-Refed | Down | GO:0031012 extracellular matrix  GO:0030055 cell-substrate junction  GO:0015629 actin cytoskeleton  GO:0101002 ficolin-1-rich granule  GO:0043292 contractile fiber  GO:0098862 cluster of actin-based cell projections  GO:0005775 vacuolar lumen  GO:0036019 endolysosome  GO:0031904 endosome lumen  GO:0032994 protein-lipid complex | 8  7  7  5  5  4  4  3  3  2 | 496  411  482  183  225  143  170  20  36  38 |
|  | Up | GO:0015629 actin cytoskeleton  GO:0005775 vacuolar lumen  GO:0043292 contractile fiber  GO:0031252 cell leading edge  GO:0030055 cell-substrate junction  GO:0031012 extracellular matrix  GO:0101002 ficolin-1-rich granule  GO:1904949 ATPase complex  GO:0005776 autophagosome  GO:0036019 endolysosome | 9  8  8  8  8  7  5  4  3  2 | 482  170  225  393  411  496  183  102  88  20 |
| Fed-Refed | Down | GO:0015629 actin cytoskeleton  GO:0030055 cell-substrate junction  GO:0031252 cell leading edge  GO:0099568 cytoplasmic region  GO:0031983 vesicle lumen  GO:0043292 contractile fiber  GO:0098858 actin-based cell projection  GO:0101002 ficolin-1-rich granule  GO:0043209 myelin sheath  GO:0005581 collagen trimer | 20  12  12  11  9  9  8  6  6  4 | 482  411  393  479  337  225  197  183  158  87 |
|  | Up | GO:0005874 microtubule  GO:0015629 actin cytoskeleton  GO:0043292 contractile fiber  GO:0005774 vacuolar membrane  GO:0005775 vacuolar lumen  GO:1904949 ATPase complex  GO:0016605 PML body  GO:0005776 autophagosome  GO:0031904 endosome lumen  GO:0036019 endolysosome | 10  9  9  7  7  7  6  4  2  2 | 402  482  225  397  170  102  99  88  36  20 |

- 1. Molecular function

| Fed-Fasted | Down | GO:0004674 protein serine/threonine kinase activity  GO:0001228 DNA-binding transcription activator activity, RNA polymerase II-specific  GO:0003779 actin binding  GO:0017124 SH3 domain binding  GO:0008307 structural constituent of muscle  GO:0047485 protein N-terminus binding  GO:0008565 protein transporter activity  GO:0008013 beta-catenin binding  GO:0050699 WW domain binding  GO:0016645 oxidoreductase activity, acting on the CH-NH group of donors | 9  7  7  4  4  3  3  3  2  2 | 449  444  419  126  44  104  89  83  31  26 |
| --- | --- | --- | --- | --- |
|  | Up | GO:0004674 protein serine/threonine kinase activity  GO:0003779 actin binding  GO:0044389 ubiquitin-like protein ligase binding  GO:0005516 calmodulin binding  GO:0002020 protease binding  GO:0008307 structural constituent of muscle  GO:0008013 beta-catenin binding  GO:0030547 receptor inhibitor activity  GO:0042805 actinin binding  GO:0030506 ankyrin binding | 11  8  6  6  4  4  3  2  2  2 | 449  419  298  192  124  44  83  39  39  20 |
| Fasted-Refed | Down | GO:0004175 endopeptidase activity  GO:0048037 cofactor binding  GO:0017171 serine hydrolase activity  GO:0003779 actin binding  GO:0016614 oxidoreductase activity, acting on CH-OH group of donors  GO:0017124 SH3 domain binding  GO:0017022 myosin binding  GO:0098631 cell adhesion mediator activity  GO:0008307 structural constituent of muscle  GO:0042805 actinin binding | 8  7  7  6  3  3  3  2  2  2 | 436  492  208  419  130  126  68  59  44  39 |
|  | Up | GO:0003779 actin binding  GO:0004674 protein serine/threonine kinase activity  GO:0008307 structural constituent of muscle  GO:0061134 peptidase regulator activity  GO:0002020 protease binding  GO:0098631 cell adhesion mediator activity  GO:0017022 myosin binding  GO:0042805 actinin binding  GO:0070063 RNA polymerase binding  GO:0001098 basal transcription machinery binding | 10  6  5  4  4  4  3  3  2  2 | 419  449  44  211  124  59  68  39  58  53 |
| Fed-Refed | Down | GO:0003779 actin binding  GO:0050839 cell adhesion molecule binding  GO:0016741 transferase activity, transferring one-carbon groups  GO:0008022 protein C-terminus binding  GO:0051082 unfolded protein binding  GO:0005200 structural constituent of cytoskeleton  GO:0008135 translation factor activity, RNA binding  GO:0005518 collagen binding  GO:0031369 translation initiation factor binding  GO:0016645 oxidoreductase activity, acting on the CH-NH group of donors | 14  10  6  6  5  4  4  3  3  2 | 419  478  221  187  118  101  87  67  31  26 |
|  | Up | GO:0004674 protein serine/threonine kinase activity  GO:0003779 actin binding  GO:0019787 ubiquitin-like protein transferase activity  GO:0044389 ubiquitin-like protein ligase binding  GO:0061134 peptidase regulator activity  GO:0002020 protease binding  GO:0008307 structural constituent of muscle  GO:0098631 cell adhesion mediator activity  GO:0005518 collagen binding  GO:0017091 AU-rich element binding | 13  8  8  7  6  5  5  4  3  2 | 449  419  416  298  211  124  44  59  67  28 |

- 1. KEGG pathways

| Fed-Fasted | Down | hsa04510 Focal adhesion  hsa04015 Rap1 signaling pathway  hsa04722 Neurotrophin signaling pathway  hsa04912 GnRH signaling pathway  hsa05211 Renal cell carcinoma  hsa05031 Amphetamine addiction  hsa04720 Long-term potentiation  hsa04137 Mitophagy  hsa05416 Viral myocarditis  hsa04744 Phototransduction | 6  5  5  4  3  3  3  3  3  2 | 199  206  119  93  69  68  67  65  59  28 |
| --- | --- | --- | --- | --- |
|  | Up | hsa04120 Ubiquitin mediated proteolysis  hsa04371 Apelin signaling pathway  hsa04152 AMPK signaling pathway  hsa04910 Insulin signaling pathway  hsa04068 FoxO signaling pathway  hsa04922 Glucagon signaling pathway  hsa04920 Adipocytokine signaling pathway  hsa04137 Mitophagy  hsa00020 Citrate cycle (TCA cycle)  hsa04710 Circadian rhythm | 7  6  6  5  5  5  4  4  4  3 | 136  137  120  137  132  103  69  65  30  31 |
| Fasted-Refed | Down | hsa04510 Focal adhesion  hsa04140 Autophagy  hsa04152 AMPK signaling pathway  hsa01200 Carbon metabolism  hsa04919 Thyroid hormone signaling pathway  hsa04612 Antigen processing and presentation  hsa05211 Renal cell carcinoma  hsa00020 Citrate cycle (TCA cycle)  hsa04961 Endocrine and other factor-regulated calcium reabsorption  hsa04964 Proximal tubule bicarbonate reclamation | 4  4  3  3  3  3  3  3  2  2 | 199  128  120  116  116  77  69  30  47  23 |
|  | Up | hsa04142 Lysosome  hsa04371 Apelin signaling pathway  hsa04910 Insulin signaling pathway  hsa04140 Autophagy  hsa04722 Neurotrophin signaling pathway  hsa03018 RNA degradation  hsa00310 Lysine degradation  hsa05134 Legionellosis  hsa00600 Sphingolipid metabolism  hsa04710 Circadian rhythm | 6  4  4  4  3  3  2  2  2  2 | 123  137  137  128  119  79  59  55  47  31 |
| Fed-Refed | Down | hsa04810 Regulation of actin cytoskeleton  hsa04510 Focal adhesion  hsa04360 Axon guidance  hsa04015 Rap1 signaling pathway  hsa04919 Thyroid hormone signaling pathway  hsa05211 Renal cell carcinoma  hsa04260 Cardiac muscle contraction  hsa04520 Adherens junction  hsa05416 Viral myocarditis  hsa05134 Legionellosis | 9  9  7  6  5  5  4  3  3  3 | 213  199  175  206  116  69  78  72  59  55 |
|  | Up | hsa04910 Insulin signaling pathway  hsa04142 Lysosome  hsa04932 Non-alcoholic fatty liver disease (NAFLD)  hsa04371 Apelin signaling pathway  hsa04120 Ubiquitin mediated proteolysis  hsa04068 FoxO signaling pathway  hsa04137 Mitophagy  hsa00270 Cysteine and methionine metabolism  hsa04710 Circadian rhythm  hsa04744 Phototransduction | 6  6  5  5  5  4  4  3  3  2 | 137  123  149  137  136  132  65  47  31  28 |

Note on phylogenies:

ML phylogenies were made in IQ-TREE with 1000 bootstrap replicates, except for insulin phylogenies which were made with 1000 ultra-fast bootstrap replicates. NJ trees were made with MEGA X with 1000 bootstrap replicates. BI trees were made with MrBayes and trees from both runs were pooled (after removing burn-in) to create the maximum clade credibility tree.

Models for phylogeny building were chosen by ModelFinder (as part of IQ-TREE), but not set in MrBayes.

Taxon labels here are unchanged from original taxon labels reflecting the sequence names in the alignments. Species abbreviations: Hsap, *Homo sapiens*; Mmus, *Mus musculus*; Ggal, *Gallus gallus*; Locu, *Lepisosteus oculatus*; Cint, *Ciona robusta* (formerly *Ciona intestinalis* A); Blan, *Branchiostoma lanceolatum*; Bbel, *B. belcheri*; Bflo, *B. florida*e; Skow, *Saccoglossus kowalevskii*; Spur, *Strongylocentroides purpuratus*; Obim, *Octopus bimaculoides*; Cgig, *Crassostrea gigas*; Lgig, *Lottia gigantea*; Tcas, *Tribolium castaneum*; Dmel, *Drosophila melanogaster*; Cele, *Caenorhabdits elegans*; Hvul, *Hydra vulgaris*.

1. INS/IGF phylogenies
   1. ML
      1. Supplementary figure 2: Maximum Likelihood phylogeny of the trimmed alignment of Insulin/IGF genes. ModelFinder chose the model VT+I + G4. Branch labels are support values out of 1000 bootstrap replicates. See Note on phylogenies for methods. Alignments can be found in Additional file 4.


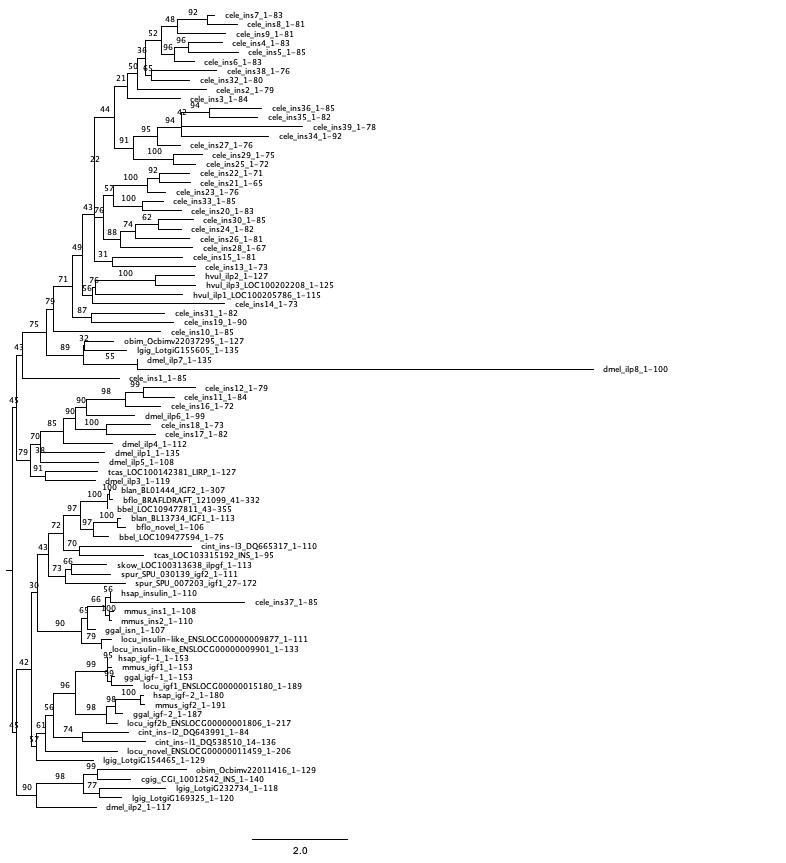


- - 1. Supplementary figure 3: Maximum Likelihood phylogeny of the full length alignment of Insulin/IGF genes with more sequences from vertebrates. ModelFinder chose the model VT+R4. Branch labels are support values out of 1000 bootstrap replicates. See Note on phylogenies for methods. Alignments can be found in Additional file 4.


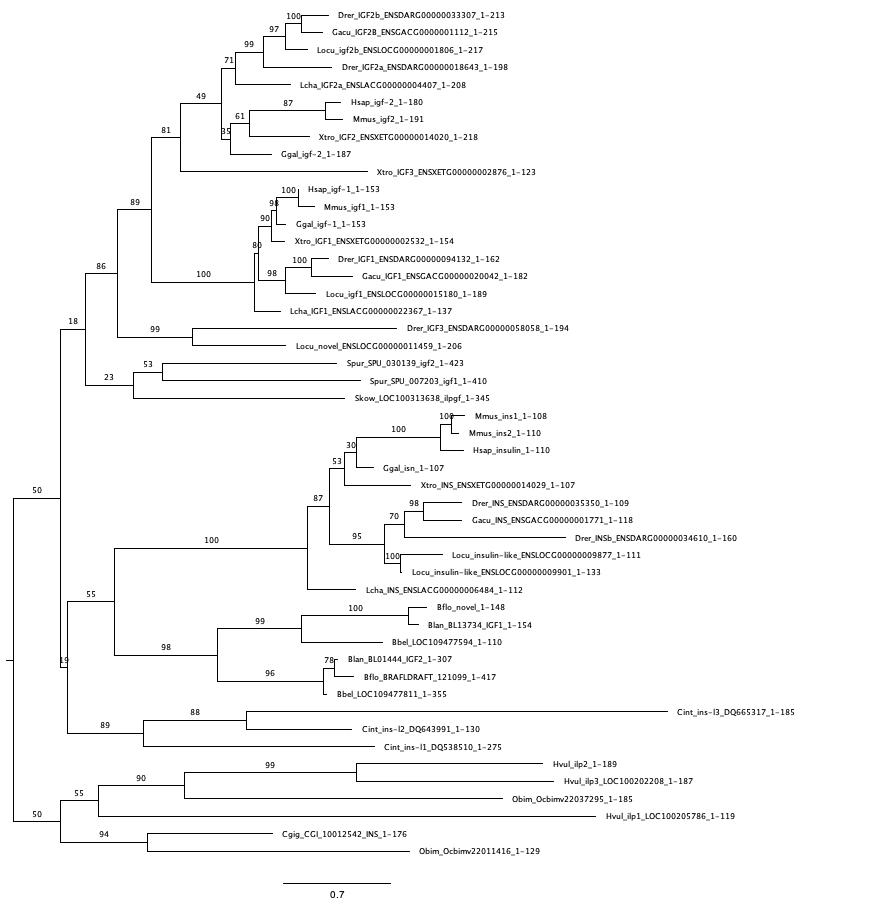


- 1. BI
     1. Supplementary figure 4: Bayesian inference phylogeny of the trimmed alignment of Insulin/IGF genes. MrBayes settled on model 1 with an alpha parameter a=1.3415. Branch labels are posterior probabilities. See Note on phylogenies for methods. Alignments can be found in Additional file 4.


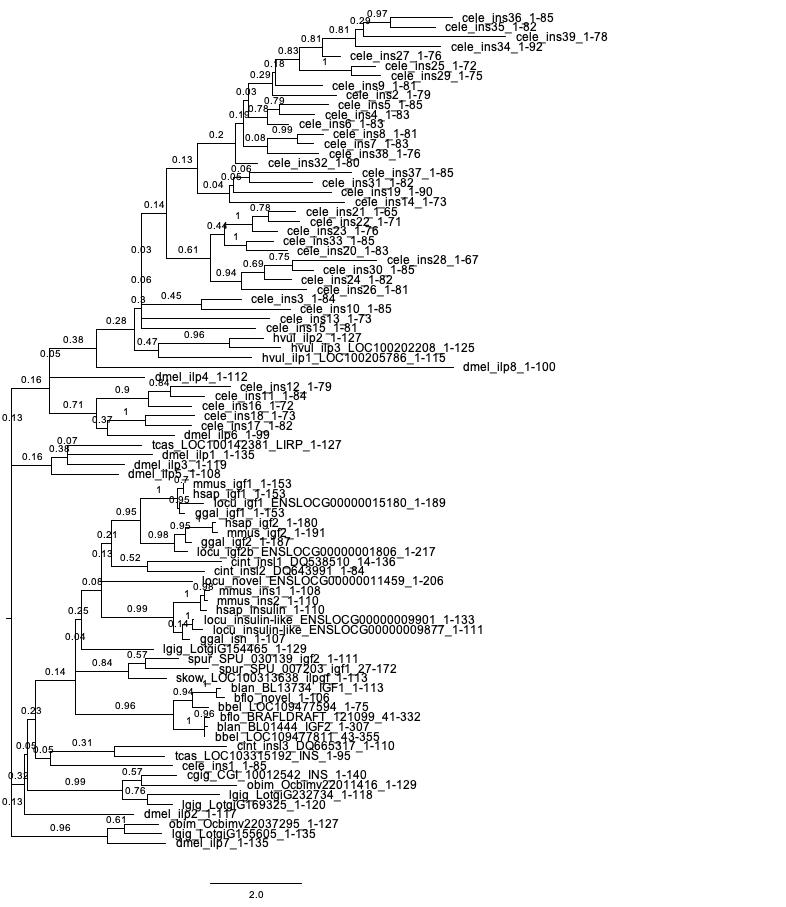


- 1. NJ
     1. Supplementary figure 5: Neighbor-joining phylogeny of the trimmed alignment of Insulin/IGF genes. The closest available model to the one chosen by ModelFinder was JTT+G4; a=1.55. Branch labels are support values out of 1000 bootstrap replicates. See Note on phylogenies for methods. Alignments can be found in Additional file 4.


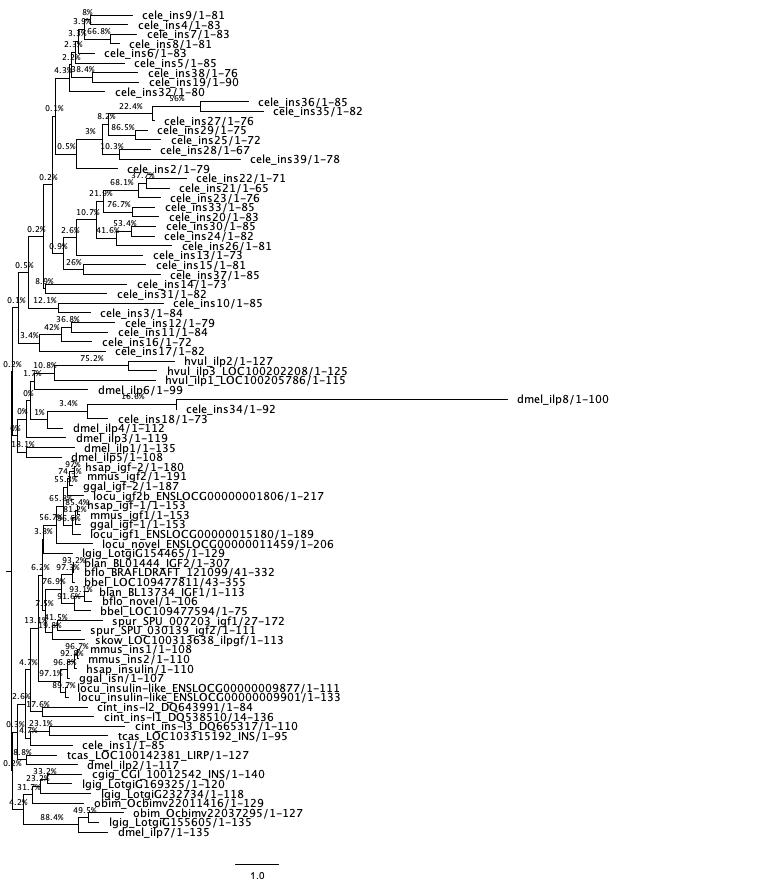


1. PI3KC phylogenies
   1. Supplementary figure 6: Maximum likelihood phylogeny of Pi3K catalytic subunits. ModelFinder choose the model LG+F+R6. Branch labels are support values out of 1000 bootstrap replicates. See Note on phylogenies for methods. Alignments can be found in Additional file 4.


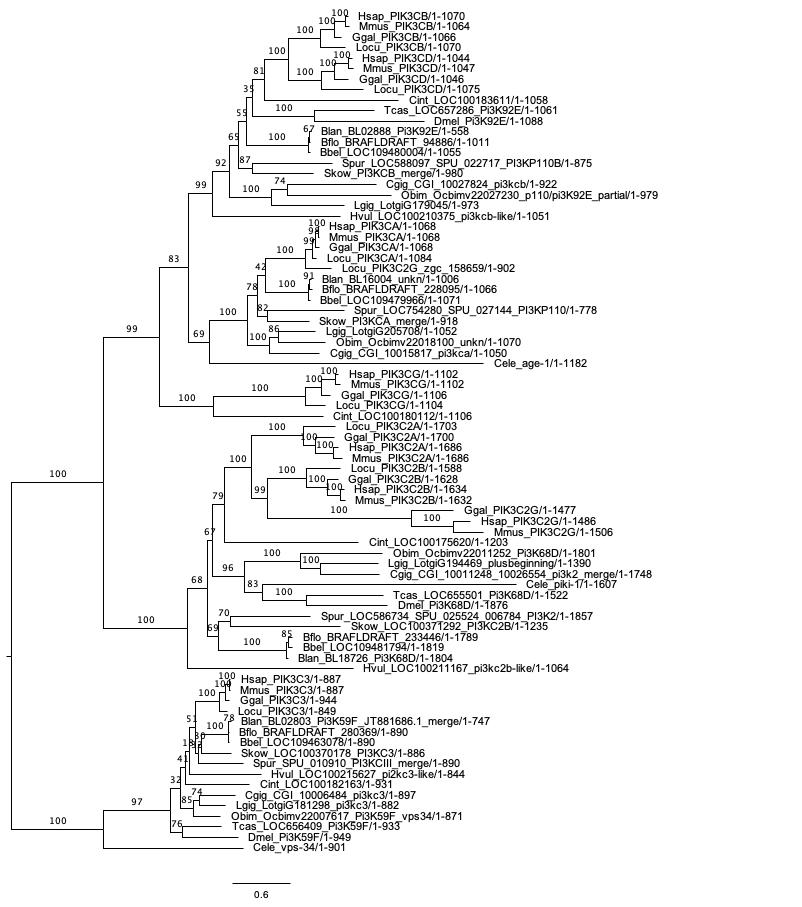


- 1. Supplementary figure 7: Bayesian inference phylogeny of Pi3K catalytic subunits. MrBayes settled on the model 1 with an alpha parameter of a=1.204. Branch labels are posterior probabilities. See Note on phylogenies for methods. Alignments can be found in Additional file 4.


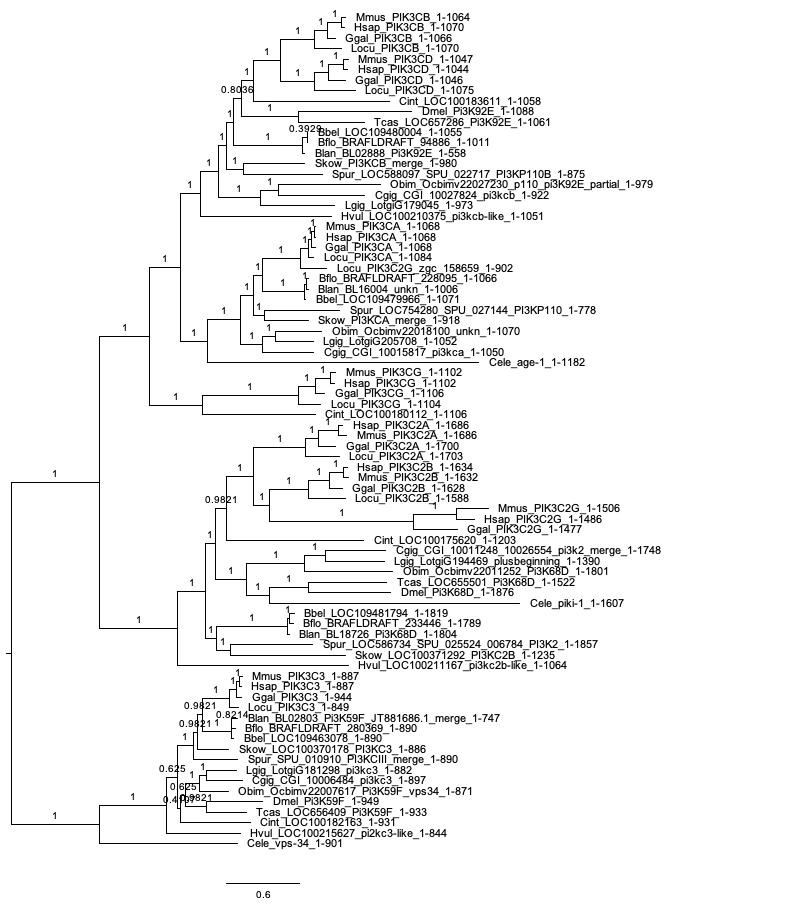


- 1. Supplementary figure 8: Neighbor-joining phylogeny of Pi3K catalytic subunits. The closest available model to the one chosen by ModelFinder was JTT. Branch labels are support values out of 1000 bootstrap replicates. See Note on phylogenies for methods. Alignments can be found in Additional file 4.


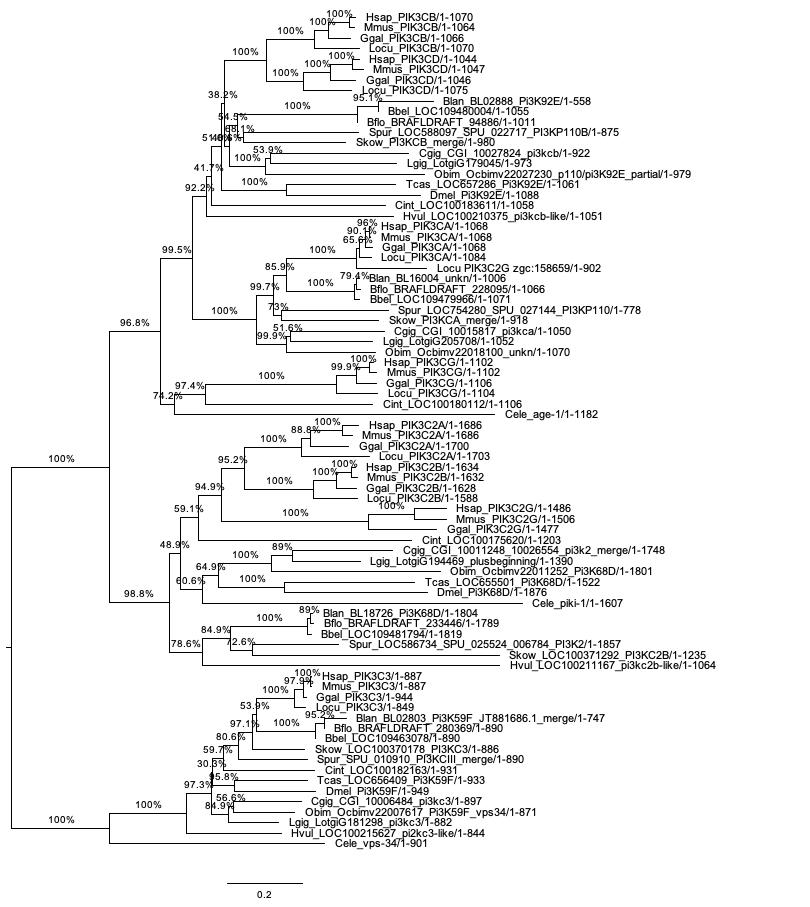


1. PI3KR phylogenies
   1. ML
      1. Supplementary figure 9: Maximum likelihood phylogeny of class I Pi3K regulatory subunits 1, 2, and 3 (PI3KR1/2/3). ModelFinder chose the model JTTDCMut+I+G4. Branch labels are support values out of 1000 bootstrap replicates. See Note on phylogenies for methods. Alignments can be found in Additional file 4.


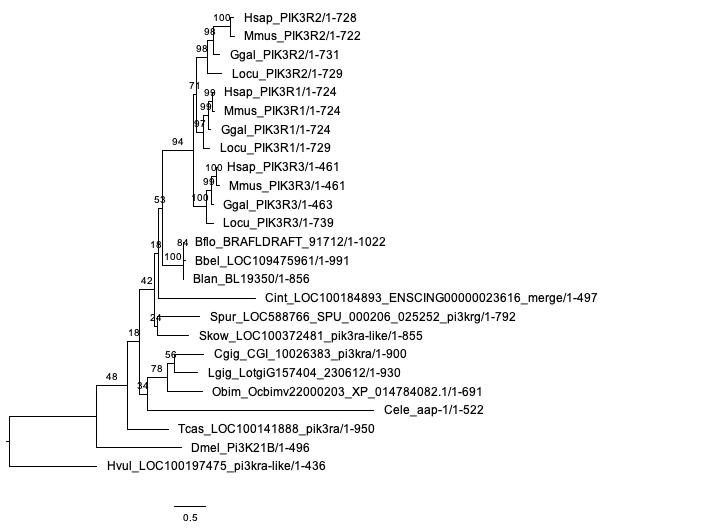


- - 1. Supplementary figure 10: Maximum likelihood phylogeny of class III Pi3K regulatory subunit (PI3KR4). ModelFinder chose the model LG+F+R4. Branch labels are support values out of 1000 bootstrap replicates. See Note on phylogenies for methods. Alignments can be found in Additional file 4.


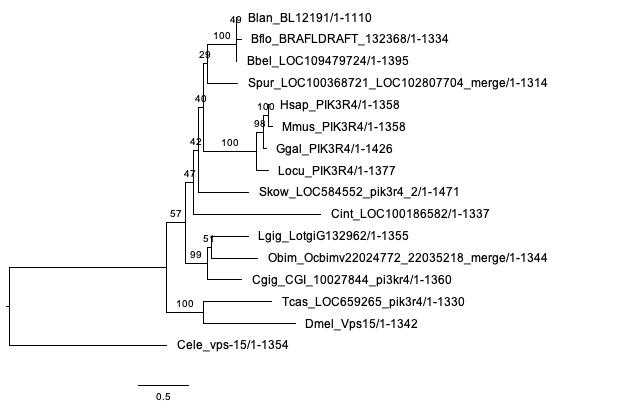


- - 1. Supplementary figure 11: Maximum likelihood phylogeny of class I Pi3K regulatory subunits 5 and 6 (PI3KR5/6). ModelFinder chose the model JTT+F+G4. Branch labels are support values out of 1000 bootstrap replicates. See Note on phylogenies for methods. Alignments can be found in Additional file 4.


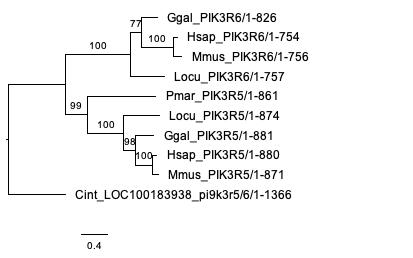


- 1. BI
     1. Supplementary figure 12: Bayesian inference phylogeny of Pi3K class I regulatory subunits 1, 2, and 3 (PI3KR1/2/3). MrBayes settled on model 1 with an alpha parameter of a=1.5469. Branch labels are posterior probabilities. See Note on phylogenies for methods. Alignments can be found in Additional file 4.


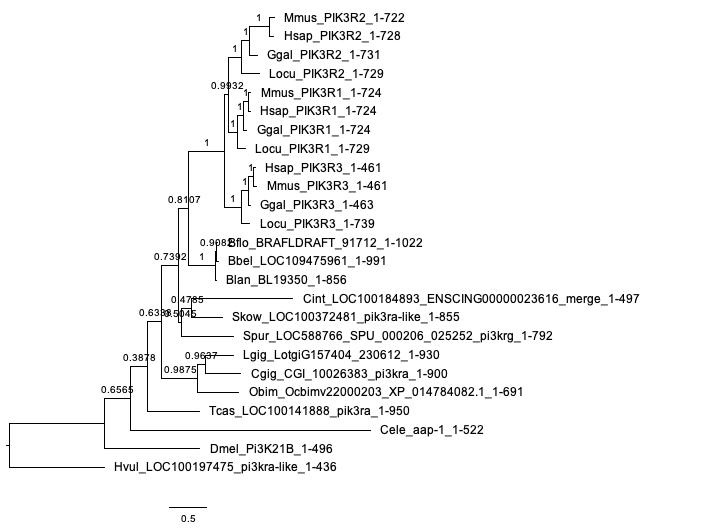


- - 1. Supplementary figure 13: Bayesian inference phylogeny of Pi3K class III regulatory subunit (PI3KR4). MrBayes settled on model 1 with an alpha parameter of a=1.169. Branch labels are posterior probabilities. See Note on phylogenies for methods. Alignments can be found in Additional file 4.


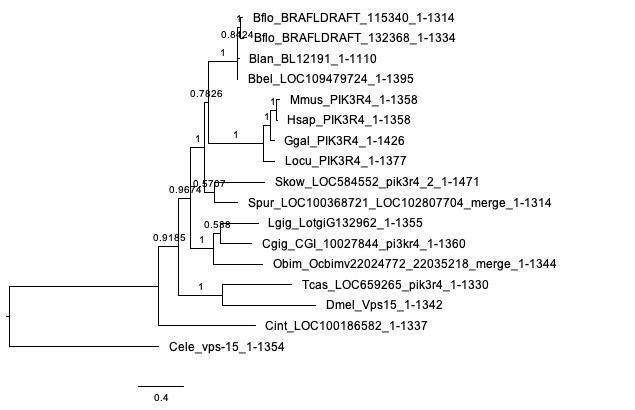


- - 1. Supplementary figure 14: Bayesian inference phylogeny of Pi3K class I regulatory subunits 5 and 6 (PI3KR5/6). MrBayes settled on model 1 with an alpha parameter of a=2.903. Branch labels are posterior probabilities. See Note on phylogenies for methods. Alignments can be found in Additional file 4.


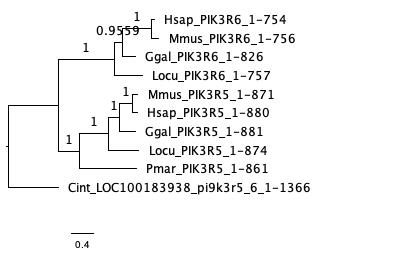


- 1. NJ
     1. Supplementary figure 15: Neighbor-joining phylogeny of P13K class II regulatory subunits 1, 2, and 3 (PI3KR1/2/3). The closest available model to the one chosen by ModelFinder was JTT+G; a=1.5. Branch labels are support values out of 1000 bootstrap replicates. See Note on phylogenies for methods. Alignments can be found in Additional file 4.


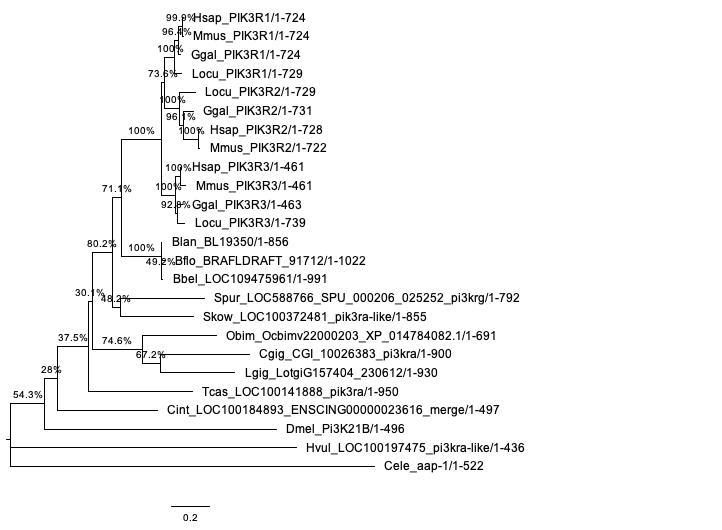


- - 1. Supplementary figure 16: Neighbor-joining phylogeny of Pi3K class III regulatory subunit (PI3KR4). The closest available model to the one chosen by ModelFinder was JTT. Branch labels are support values out of 1000 bootstrap replicates. See Note on phylogenies for methods. Alignments can be found in Additional file 4.


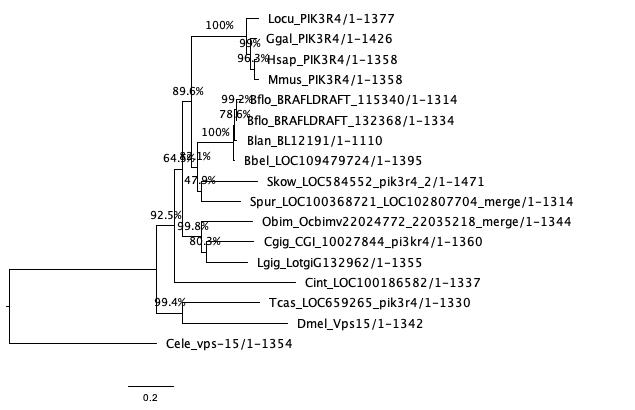


- - 1. Supplementary figure 17: Neighbor-joining phylogeny of Pi3K class I subunits 5 and 6 (PI3KR5/6). The closest available model to the one chosen by ModelFinder was JTT+G,;a=2.53. Branch labels are support values out of 1000 bootstrap replicates. See Note on phylogenies for methods. Alignments can be found in Additional file 4.


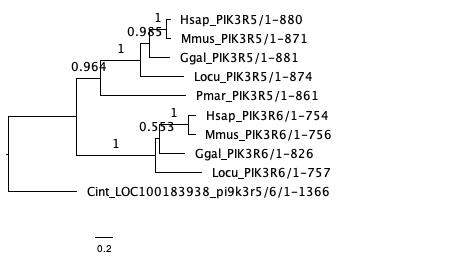


1. FOXO phylogenies
   1. Supplementary figure 18: Maximum likelihood phylogeny of FOXO genes. ModelFinder chose the model JTT+F+R3. Branch labels are support values out of 1000 bootstrap replicates. Colours represent species relationships (vertebrates, green; cephalochordates, pink; urochordate, red; ambulacrarians, orange; lophotrochozoans, purple; ecdysozoans, blue; cnidarian, black). See Note on phylogenies for methods. Alignments can be found in Additional file 4.


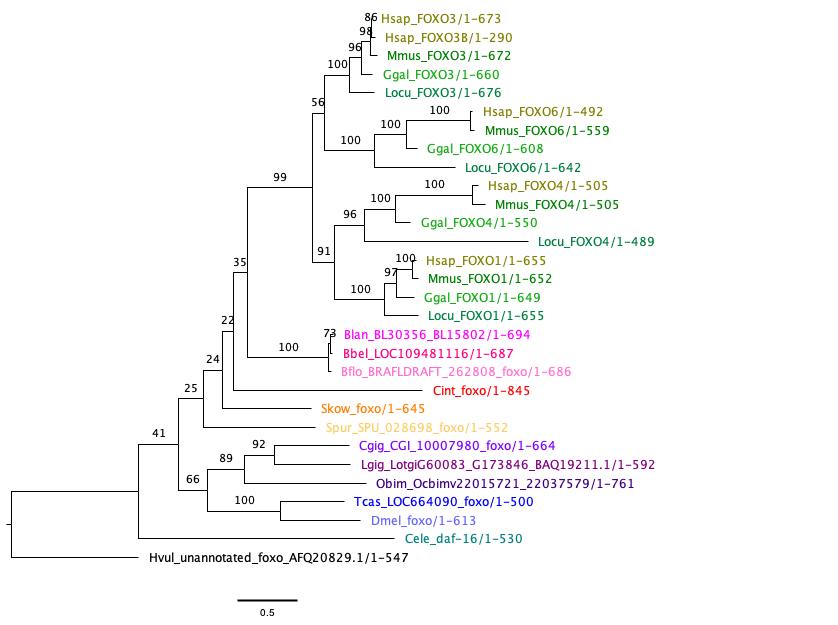


- 1. Supplementary figure 19: Bayesian inference phylogeny of FOXO genes. MrBayes settled on model 1 with an alpha parameter of a=1.238. Branch labels are posterior probabilities. Colours represent species relationships (vertebrates, green; cephalochordates, pink; urochordate, red; ambulacrarians, orange; lophotrochozoans, purple; ecdysozoans, blue; cnidarian, black). See Note on phylogenies for methods. Alignments can be found in Additional file 4.


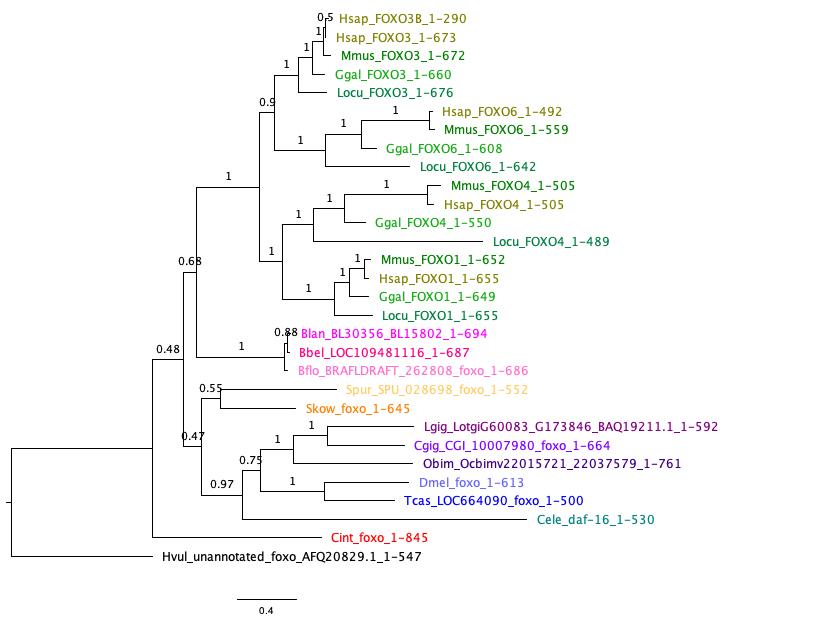


- 1. Supplementary figure 20: Neighbor-joining phylogeny of FOXO genes. The closest available model to the one chosen by ModelFinder was JTT. Branch labels are support values out of 1000 bootstrap replicates. Colours represent species relationships (vertebrates, green; cephalochordates, pink; urochordate, red; ambulacrarians, orange; lophotrochozoans, purple; ecdysozoans, blue; cnidarian, black). See Note on phylogenies for methods. Alignments can be found in Additional file 4.


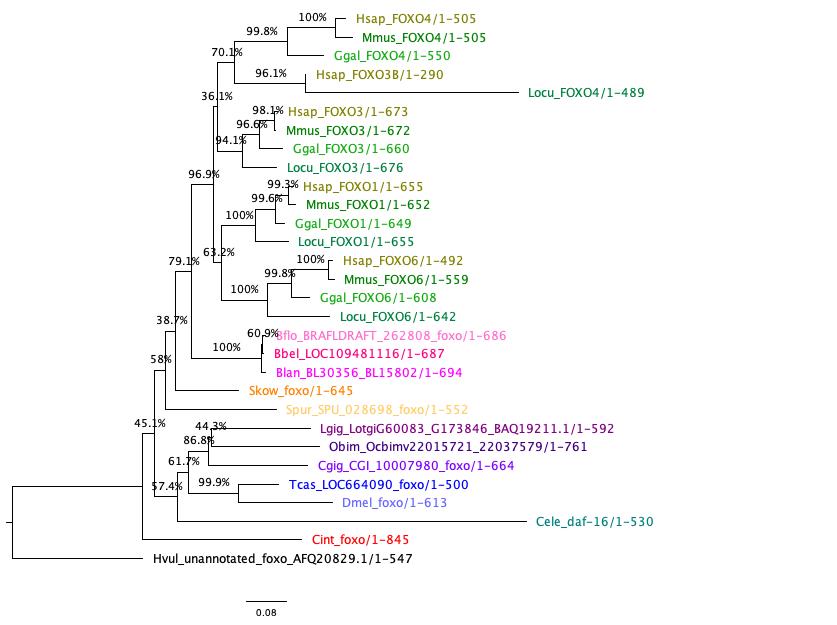


1. Supplementary table 2: Myoblast fusion genes
   1. Expansion of main text Table 1: Human genes involved in myoblast fusion, their functions, and their fly and amphioxus orthologues, many of which were detected in the amphioxus muscle transcriptome, and one of which was differentially expressed. P in parentheses denotes paralogues not involved directly in myoblast fusion in humans.

| Process | | Human genes | Function in vertebrates | Fly gene | Function in flies | Amphioxus orthologues | Expressed in muscle | Differentially expressed |
| --- | --- | --- | --- | --- | --- | --- | --- | --- |
| Myoblast fusion | Cell recognition | Nephrin (NPHS1)  (P: NPHS2) | Immunoglobulin domain containing membrane bound marker for myoblast fusion [1–3] | sns (Sticks and stones) | Expressed in FCM, allows for myoblast recognition, binds Duf/Rst [4, 5] | NPHS |  |  |
|  |  |  |  | hbs (Hibris) |  |  |  |  |
|  |  | Kirrel1, Kirrel2, Kirrel3 | Immunoglobulin domain containing membrane bound receptor proteins needed for myoblast recognition [6] | duf (Dumfounded) | Expressed in founder cells, allows for myoblast recognition, binds Sns/Rst [7, 8] | Kirrel |  |  |
|  |  |  |  | rst (Roughest) |  |  |  |  |
|  |  | Myomaker (TMEM8C) (P: TMEM8A & TMEM8B) | Cell surface adhesion (a), regulator in EGFR pathway (b), and myoblast specific fusion mediator (c) [9, 10] | CG13654 (Tmem8a/b) | Function not characterised, localised to membrane (automatic annotation) | Tmem8a/b | yes | – |
|  |  | Myomixer | Partner of Myomaker; increases efficiency of fusion [11] | – | – | – | – | – |
|  |  | TANC1, TANC2 | No function in myoblast fusion recorded [3, 12] | rols (Rolling pebbles, a.k.a. Anti-social) | Intracellular adaptor protein expressed in founder cells; links Duf/Rst to Mbc [12] | TANC | – |  |
|  |  | Junctional adhesional molecule 2 (JamB) & 3 (JamC) (P: JamA) | Cellular receptors essential for myoblast fusion [10, 13] | – | – | JAM | – |  |
|  |  | Netrin | Guidance of CNS axons, secreted by myoblasts, receptor is neogenin [3, 14] | netA (Netrin-A) | No apparent role in myoblast fusion [15] | NTN1 | yes | – |
|  |  | Neogenin | Cell surface receptor mediating adhesion in myogenesis, bound by Netrin [14] | fra (Frazzled) | No apparent role in myoblast fusion [15] | Neo | yes | – |
|  | Cell adhesion | Cadherin-2 (M-cad.) | Transmembrane Ca-dependent cell adhesion glycoproteins, adhesion of which activates Rac1 [16] | – | – | Cdh15 | yes | – |
|  |  | Cadherin-15 (N-cad.) |  | CadN (Cadherin_N) | No apparent role in myoblast fusion [17] |  |  |  |
|  |  | Caveolin1, Cav2, Cav3 | Scaffolding protein within caveolar membranes, required for myoblast fusion [18, 19] | Caveolin-1 | Function not clear, potential expression in muscles [20] | Cav1/3 | yes | – |
|  |  | Myoferlin (a.k.a. Fer1L3) (P: Dysferlin Fer1L1, Fer1L5) | Calcium sensitive membrane binding, role in myotube maturation [21] | mfr (Misfire) | No apparent role in myoblast fusion [22] | Myof | yes | – |
|  |  | Integrin b1 (P: Integrin b3) | Cell surface receptor, required for fusion, regulators of muscle fiber cytoskeleton [23] | mys (Myospheriod) | Integrin beta subunit, heterodimerises with if to form ECM receptor [24] | ItgB1/3 | yes | – |
|  |  | Integrin a1, a3, a4, a5, a6, a7, a9, and av | Binding partners of B1 integrin, expressed in muscle, but myoblast fusion function not determined. [19, 23, 25] | if (Inflated) | Integrin alpha subunit, heterodimerises with mys to form ECM receptor [24] | ITGA4, ITGA5, ITGA6 | yes | – |
|  |  | Protein tyrosine kinase 2 (PTK2) | Tyrosine kinase involved in focal adhesion, integrin signaling, regulates caveolin 3 and B1D [19] | fak (Focal adhesion kinase) | nRTK involved in signaling from integrins to regulate cytoskeletal dynamics [26] | Fak | – |  |
|  | Cell signalling | Brag2 (IQSEC) | Guanine nucleotide exchange factor interacts with Cadherin-2, activates Arf6 [27, 28] | siz (Schizo, a.k.a. loner) | GEF activated by Duf or Rst in founder cell, activates Arf6 [29, 30] | IQSEC | yes | – |
|  |  | ADP-ribosylation factor 6 (P: ARF1, 3, 4, & 5) | Protein trafficking GTP-binding regulating endocytic recycling and cytoskeleton remodelling [2, 28] | Arf51F (ADP ribosylation factor at 51F; Arf6) | Effector of loner, activates Rac in founder cell (Chen et al, 2003; Kim et al., 2007) | Arf6 | yes | – |
|  |  | Dock1, Dock5 | Critical regulator of myoblast fusion, activates Rac GTPase signalling [2, 31] | mbc (Myoblast city) | Guanine nucleotide exchange factor for Rac [32] | Dock1/5 | – |  |
|  |  | Crk, Crkl | Dock protein adaptors, required for myoblast fusion [33] | Crk (Crk oncogene) | Dock protein adaptor [34, 35] | Crk | yes | – |
|  |  | Elmod1, Elmod2, Elmod3 | GTPase-activating protein, acts on ARF6 with Dock [36] | Ced-12 (ELMO) | Dimeric guanine nucleotide exchange factor that regulates Rac [36, 37] | ELMOD | – |  |
|  |  | Rac1 (P: Rac2, Rac3) | Membrane-associated small GTPase, related to myoblast fusion through M-cadherin, also activated by Dock/Elmo and Arf6, regulates actin dynamics [16, 31, 38] | Rac1, Rac2 | GTPases regulating the actin cytoskeleton, act redundantly in myoblast fusion [39] | Rac | yes | – |
|  | Actin dynamics | – | – | blow (Blown fuse) | Cytoplasmic protein, regulates actin cytoskeletal dynamics via modulating the stability of the scar/WAVE complex [40] | – | – | – |
|  |  | N-WASP (P:WASP) | Components of the N-WASP complex [41, 42] | WASp | Arp2/3 activator, recruited to sites of fusion by sns, mediated by crk, activates wsp [32, 43] | WASP | yes | – |
|  |  | WIPF |  | Wip (Vrp/Sltr) |  | WIPF | – |  |
|  |  | CYFIP1, CYFIP2 |  | Sra-1 |  | CYFIP | – |  |
|  |  | Nck-associated protein1 Nckap1 (P: Nckap1L) |  | hem (a.k.a. kette) |  | NCKAP | – |  |
|  |  | WASP family member 1 (WASF1) (P: WASF2 & 3) | Components of the WAVE complex, binds actin and Arp2/3 complex, activated by Rac [41, 42] | scar (Suppressor of cAMP Receptor) | Components of scar/WAVE complex; interacts with blow, recieves Rac signal, activates Arp2/3, regulates actin polymerisation [2, 44] | WASF | yes | – |
|  |  | ABI2 |  | abi |  | ABI | – |  |
|  |  | Brk1 |  | HSPC300 |  | – |  |  |
|  |  | Arpc1a, Arpc1b | Probable component of Arp2/3 complex involved in reculation of actin polymerisation [45] | ArpC | Component of Arp2/3 complex, actin polymerisation [2] | Arp1 | yes | – |
|  |  | Arp2, Apr3, Arp4, Arp5 |  | Arp66B |  | Arp2, Arp3, Arp4, Arp5 | yes (all but Arp4) | – |
| Regulation | | Nuclear factor of activated T-cells cytoplasmic 2 (P: NFATC1, 3, 4, & NFAT5) | TF regulating myogenesis and myoblast fusion; regulates myotube fusion [46, 47] | NFAT (NFAT nuclear factor) | TF in many processes, not specific to myoblasts [48]; role in neurons [49] | Nfatc | yes | – |
|  |  | Myocyte-specific enhancer factor 2A (P: MEF2B, C, & D) | TF that binds MEF2 element on muscle genes for cell growth, survival, and apoptosis downstream of MAPK [2, 50] | Mef2 | TF regulating many muscle genes, controls formation and patterning of body wall muscle [51] | Mef2 | yes | yes |
|  |  | Myogenic Differentiation 1, Myogenin, Myogenic factor 5, & Myogenic factor 6 | TFs driving myogenesis [52–54] | Nau (Nautilus) | TE marking founder cells, activates muscle genes, required for myogenesis [55] | MRF1, MRF2a, MRF2b, MRF3, & MRF4 | yes (all but MRF4) | – |
|  |  | Paired box 3 & 7 (P: 2, 4, 5, 6, 8, 9) | TFs with a paired domain and homeodomain involved in neural development and myogenesis [53, 56] | prd (paired) (P: sv, ey, eyg, gsb, Poxn, Poxn) | Paired-rule gene with a paired domain and a homeodomain, TF in segmentation and development [57, 58] | Pax3/7a, Pax3/7b | yes | – |
|  |  | SIX homeobox 1 & 4 (P: Six2, 3, 5, & 6) | TFs with homeodomain involved in development, including muscle [53] | sine oculis, Optix, Six4 | TF with homeodomain, regulating eye development, interacts with eyeless (Pax6 orthologue) [59] | Six1/2, Six3/6, & Six4/5 | yes Six1/2 & Six4/5 | – |

- 1. References

1. Sohn RL, Huang P, Kawahara G, Mitchell M, Guyon J, Kalluri R, et al. A role for nephrin, a renal protein, in vertebrate skeletal muscle cell fusion. Proc Natl Acad Sci. 2009;106:9274–9.

2. Abmayr SM, Pavlath GK. Myoblast fusion: lessons from flies and mice. Development. 2012;139:641–56.

3. Rochlin K, Yu S, Roy S, Baylies MK. Myoblast fusion: When it takes more to make one. Dev Biol. 2010;341:66–83.

4. Bour BA, Chakravarti M, West JM, Abmayr SM. Drosophila SNS, a member of the immunoglobulin superfamily that is essential for myoblast fusion. Genes Dev. 2000;14:1498–511.

5. Artero RD, Castanon I, Baylies MK. The immunoglobulin-like protein Hibris functions as a dose-dependent regulator of myoblast fusion and is differentially controlled by Ras and Notch signaling. Development. 2001;128:4251–64.

6. Srinivas BP, Woo J, Leong WY, Roy S. A conserved molecular pathway mediates myoblast fusion in insects and vertebrates. Nat Genet. 2007;39:781–6.

7. Ruiz-Gómez M, Coutts N, Price A, Taylor M V., Bate M. Drosophila Dumbfounded. Cell. 2000;102:189–98.

8. Strünkelnberg M, Bonengel B, Moda LM, Hertenstein A, Gert de Couet H, Ramos RGP, et al. Rst and its paralogue kirre act redundantly during embryonic muscle development in Drosophila. Development. 2001;128:4229–39.

9. Landemaine A, Rescan P-Y, Gabillard J-C. Myomaker mediates fusion of fast myocytes in zebrafish embryos. Biochem Biophys Res Commun. 2014;451:480–4.

10. Kim JH, Jin P, Duan R, Chen EH. Mechanisms of myoblast fusion during muscle development. Curr Opin Genet Dev. 2015;32:162–70.

11. Bi P, Ramirez-Martinez A, Li H, Cannavino J, McAnally JR, Shelton JM, et al. Control of muscle formation by the fusogenic micropeptide myomixer. Science (80- ). 2017;356:323–7.

12. Suzuki T, Li W, Zhang J-PP, Tian Q-BB, Sakagami H, Usada N, et al. A novel scaffold protein, TANC, possibly a rat homolog of Drosophila rolling pebbles (rols), forms a multiprotein complex with various postsynaptic density proteins. Eur J Neurosci. 2005;21:339–50.

13. Powell GT, Wright GJ. Jamb and Jamc Are Essential for Vertebrate Myocyte Fusion. PLoS Biol. 2011;9:e1001216.

14. Kang J-S, Yi M-J, Zhang W, Feinleib JL, Cole F, Krauss RS. Netrins and neogenin promote myotube formation. J Cell Biol. 2004;167:493–504.

15. Akin O, Zipursky SL. Frazzled promotes growth cone attachment at the source of a Netrin gradient in the Drosophila visual system. Elife. 2016;5:e20762.

16. Charrasse S, Comunale F, Fortier M, Portales-Casamar E, Debant A, Gauthier-Rouvière C. M-Cadherin Activates Rac1 GTPase through the Rho-GEF Trio during Myoblast Fusion. Mol Biol Cell. 2007;18:1734–43.

17. Schafer G, Narasimha M, Vogelsang E, Leptin M. Cadherin switching during the formation and differentiation of the Drosophila mesoderm - implications for epithelial-to-mesenchymal transitions. J Cell Sci. 2014;127:1511–22.

18. Galbiati F, Volonté D, Engelman JA, Scherer PE, Lisanti MP. Targeted down-regulation of caveolin-3 is sufficient to inhibit myotube formation in differentiating C2C12 myoblasts. Transient activation of p38 mitogen-activated protein kinase is required for induction of caveolin-3 expression and subsequent myotube fo. J Biol Chem. 1999;274:30315–21.

19. Quach NL, Biressi S, Reichardt LF, Keller C, Rando TA. Focal adhesion kinase signaling regulates the expression of caveolin 3 and β1 integrin, genes essential for normal myoblast fusion. Mol Biol Cell. 2009;20:3422–35.

20. Zhang Y, Wang Y, Qu C, Huang H, Li H, Li Y, et al. The Expression and Role of Caveolin-1 in Different Development Periods of Drosophila melanogaster. 2016.

21. Doherty KR. Normal myoblast fusion requires myoferlin. Development. 2005;132:5565–75.

22. Smith MK, Wakimoto BT. Complex regulation and multiple developmental functions of misfire, the Drosophila melanogaster ferlin gene. BMC Dev Biol. 2007;7:21.

23. Schwander M, Leu M, Stumm M, Dorchies OM, Ruegg UT, Schittny J, et al. β1 Integrins Regulate Myoblast Fusion and Sarcomere Assembly. Dev Cell. 2003;4:673–85.

24. Perkins AD, Ellis SJ, Asghari P, Shamsian A, Moore EDW, Tanentzapf G. Integrin-mediated adhesion maintains sarcomeric integrity. Dev Biol. 2010;338:15–27.

25. Madaro L, Marrocco V, Fiore P, Aulino P, Smeriglio P, Adamo S, et al. PKCθ signaling is required for myoblast fusion by regulating the expression of caveolin-3 and β1D integrin upstream focal adhesion kinase. Mol Biol Cell. 2011;22:1409–19.

26. Fujimoto J, Sawamoto K, Okabe M, Takagi Y, Tezuka T, Yoshikawa S, et al. Cloning and Characterization of Dfak56, a Homolog of Focal Adhesion Kinase, in Drosophila melanogaster. J Biol Chem. 1999;274:29196–201.

27. Pajcini K V., Pomerantz JH, Alkan O, Doyonnas R, Blau HM. Myoblasts and macrophages share molecular components that contribute to cell–cell fusion. J Cell Biol. 2008;180:1005–19.

28. Bach A-SS, Enjalbert S, Comunale F, Bodin S, Vitale N, Charrasse S, et al. ADP-Ribosylation Factor 6 Regulates Mammalian Myoblast Fusion through Phospholipase D1 and Phosphatidylinositol 4,5-Bisphosphate Signaling Pathways. Mol Biol Cell. 2010;21:2412–24.

29. Chen EH, Pryce BA, Tzeng JA, Gonzalez GA, Olson EN. Control of Myoblast Fusion by a Guanine Nucleotide Exchange Factor, Loner, and Its Effector ARF6. Cell. 2003;114:751–62.

30. Dottermusch-Heidel C, Groth V, Beck L, Önel S-FF. The Arf-GEF Schizo/Loner regulates N-cadherin to induce fusion competence of Drosophila myoblasts. Dev Biol. 2012;368:18–27.

31. Laurin M, Fradet N, Blangy A, Hall A, Vuori K, Côté J-F. The atypical Rac activator Dock180 (Dock1) regulates myoblast fusion in vivo. Proc Natl Acad Sci. 2008;105:15446–51.

32. Kim S, Shilagardi K, Zhang S, Hong SN, Sens KL, Bo J, et al. A Critical Function for the Actin Cytoskeleton in Targeted Exocytosis of Prefusion Vesicles during Myoblast Fusion. Dev Cell. 2007;12:571–86.

33. Moore CA, Parkin CA, Bidet Y, Ingham PW. A role for the Myoblast city homologues Dock1 and Dock5 and the adaptor proteins Crk and Crk-like in zebrafish myoblast fusion. Development. 2007;134:3145–53.

34. Galletta BJ, Niu X-PP, Erickson MRS, Abmayr SM. Identification of a Drosophila homologue to vertebrate Crk by interaction with MBC. Gene. 1999;228:243–52.

35. Dworak HA, Sink H. Myoblast fusion in Drosophila. BioEssays. 2002;24:591–601.

36. Brugnera E, Haney L, Grimsley C, Lu M, Walk SF, Tosello-Trampont A-C, et al. Unconventional Rac-GEF activity is mediated through the Dock180–ELMO complex. Nat Cell Biol. 2002;4:574–82.

37. Geisbrecht ER, Haralalka S, Swanson SK, Florens L, Washburn MP, Abmayr SM. Drosophila ELMO/CED-12 interacts with Myoblast city to direct myoblast fusion and ommatidial organization. Dev Biol. 2008;314:137–49.

38. Vasyutina E, Martarelli B, Brakebusch C, Wende H, Birchmeier C. The small G-proteins Rac1 and Cdc42 are essential for myoblast fusion in the mouse. Proc Natl Acad Sci U S A. 2009;106:8935–40.

39. Hakeda-Suzuki S, Ng J, Tzu J, Dietzl G, Sun Y, Harms M, et al. Rac function and regulation during Drosophila development. Nature. 2002;416:438–42.

40. Schröter RH, Lier S, Holz A, Bogdan S, Klämbt C, Beck L, et al. kette and blown fuse interact genetically during the second fusion step of myogenesis in Drosophila. Development. 2004;131:4501–9.

41. Derivery E, Gautreau A. Generation of branched actin networks: Assembly and regulation of the N-WASP and WAVE molecular machines. BioEssays. 2010;32:119–31.

42. Gruenbaum-Cohen Y, Harel I, Umansky KB, Tzahor E, Snapper SB, Shilo BZ, et al. The actin regulator N-WASp is required for muscle-cell fusion in mice. Proc Natl Acad Sci U S A. 2012;109:11211–6.

43. Massarwa R, Carmon S, Shilo B-Z, Schejter ED. WIP/WASp-Based Actin-Polymerization Machinery Is Essential for Myoblast Fusion in Drosophila. Dev Cell. 2007;12:557–69.

44. Berger S, Schafer G, Kesper DA, Holz A, Eriksson T, Palmer RH, et al. WASP and SCAR have distinct roles in activating the Arp2/3 complex during myoblast fusion. J Cell Sci. 2008;121:1303–13.

45. Richardson BE, Nowak SJ, Baylies MK. Myoblast fusion in fly and vertebrates: New genes, new processes and new perspectives. Traffic. 2008;9:1050–9.

46. Horsley V, Friday BB, Matteson S, Kegley KM, Gephart J, Pavlath GK. Regulation of the Growth of Multinucleated Muscle Cells by an Nfatc2-Dependent Pathway. J Cell Biol. 2001;153:329–38.

47. Horsley V, Pavlath GK. Forming a Multinucleated Cell: Molecules That Regulate Myoblast Fusion. Cells Tissues Organs. 2004;176:67–78.

48. Rao A, Luo C, Hogan PG. Transcription factors of the NFAT family: Regulation and function. Annual Review of Immunology. 1997;15:707–47.

49. Freeman A, Franciscovich A, Bowers M, Sandstrom DJ, Sanyal S. NFAT regulates pre-synaptic development and activity-dependent plasticity in Drosophila. Mol Cell Neurosci. 2011;46:535–47.

50. Wu W, Folter S de, Shen X, Zhang W, Tao S. Vertebrate Paralogous MEF2 Genes: Origin, Conservation, and Evolution. PLoS One. 2011;6:e17334.

51. Ranganayakulu G, Zhao B, Dokidis A, Molkentin JD, Olson EN, Schulz RA. A Series of Mutations in the D-MEF2 Transcription Factor Reveal Multiple Functions in Larval and Adult Myogenesis in Drosophila. Dev Biol. 1995;171:169–81.

52. Abmayr SM, Balagopalan L, Galletta BJ, Hong S-J. Cell and molecular biology of myoblast fusion. In: International Review of Cytology. Academic Press Inc.; 2003. p. 33–89.

53. Hindi SM, Tajrishi MM, Kumar A. Signaling Mechanisms in Mammalian Myoblast Fusion. Sci Signal. 2013;6:re2–re2.

54. Davis RL, Weintraub H, Lassar AB. Expression of a single transfected cDNA converts fibroblasts to myoblasts. Cell. 1987;51:987–1000.

55. Wei Q, Rong Y, Paterson BM. Stereotypic founder cell patterning and embryonic muscle formation in Drosophila require nautilus (MyoD) gene function. Proc Natl Acad Sci. 2007;104:5461–6.

56. Hammond CL, Hinits Y, Osborn DPS, Minchin JEN, Tettamanti G, Hughes SM. Signals and myogenic regulatory factors restrict pax3 and pax7 expression to dermomyotome-like tissue in zebrafish. Dev Biol. 2007;302:504–21.

57. Frigerio G, Burri M, Bopp D, Baumgartner S, Noll M. Structure of the segmentation gene paired and the Drosophila PRD gene set as part of a gene network. Cell. 1986;47:735–46.

58. Dohrmann C, Azpiazu N, Frasch M. A new Drosophila homeo box gene is expressed in mesodermal precursor cells of distinct muscles during embryogenesis. Genes Dev. 1990;4 12 A:2098–111.

59. Kumar JP. The sine oculis homeobox (SIX) family of transcription factors as regulators of development and disease. Cell Mol Life Sci. 2009;66:565–83.
