# Supplementary material for: Amphioxus muscle transcriptomes reveal vertebrate-like myoblast fusion genes and a highly conserved role of insulin signalling in the metabolism of muscle
Source: BMC Genomics. 2022 Feb 1;23:93. doi: 10.1186/s12864-021-08222-9 (PMC8805411; doi:10.1186/s12864-021-08222-9)
Supplement: Supplementary file 4 — Additional file 4. [file 12864_2021_8222_MOESM4_ESM.rtf]

Protein sequence alignments of INS/Akt/FOXO pathway genes.Sequences were aligned with MAFFT and alignments were not curated except for trimming the ins/IGF alignment.Proteins are identified by name if well characterised, e.g. for human or fly, and with the accession number if not.Four letter species identifiers were used, e.g. Homo sapiens becomes Hsap.1. Ins/IGF	a. Trimmedggal_igf-2/1-187                          ------------------------------MCAARQILLLLLAFLAYA-------LDSAAAYGTAETLCGGELVDTL---QFVCGDR------GFYFSRPV-----------------------------G---R-NNRRINRGIVE-ECCFR-SCDLA-L-LET-YCAKSVKSERDLSATSLAGLPAL--NKESFQKPSHAKYSKYNVWQKKSSQRLQREVPGILRARRYRWQAEGLQAAEEARAMHRPL--------------------------------------------------------------------------------------------------------------ISLPSQRPPAPRASPEATGPQEhsap_igf-2/1-180                          -----------------------MGIPM------GKSMLVLLTFLAFA-------SCCIAAYRPSETLCGGELVDTL---QFVCGDR------GFYFSRPA-----------------------------S---R--VSRRSRGIVE-ECCFR-SCDLA-L-LET-YCATPAKSERDVSTPPTV-------LPDNFPRYPVGKFFQYDTWK-QSTQRLRRGLPALLRARRGHVLAKELEAFREAK-RHRPL--------------------------------------------------------------------------------------------------------------IALPTQDPAHGGAPPEMASNRKmmus_igf2/1-191                           -----------MGGSVAGFQVP-MGIPV------GKSMLVLLISLAFA-------LCCIAAYGPGETLCGGELVDTL---QFVCSDR------GFYFSRPS-----------------------------S---R--ANRRSRGIVE-ECCFR-SCDLA-L-LET-YCATPAKSERDVSTSQAV-------LPDDFPRYPVGKFFQYDTWK-QSAGRLRRGLPALLRARRGRMLAKELKEFREAK-RHRPL--------------------------------------------------------------------------------------------------------------IVLPPKDPAHGGASSEMSSNHQhsap_igf-1/1-153                          ---MGKISSLPTQLFKCCFCDF-LKVKMHTMSSSHLFYLALCLLTF--------TS---SATAGPETLCGAELVDAL---QFVCGDR------GFYFNKPTGY------------------------------GSSSRRAPQTGIVD-ECCFR-SCDLR-R-LEM-YCAPLKPAKSARSVRAQRHTDMPKTQKEVHLKNASRG--------------------------------------SAGNKNYRM-------------------------------------------------------------------------------------------------------------------------------------mmus_igf1/1-153                           ---MGKISSLPTQLFKICLCDF-LKIKIHIMSSSHLFYLALCLLTF--------TS---STTAGPETLCGAELVDAL---QFVCGPR------GFYFNKPTGY------------------------------GSSIRRAPQTGIVD-ECCFR-SCDLR-R-LEM-YCAPLKPTKAARSIRAQRHTDMPKTQKEVHLKNTSRG--------------------------------------SAGNKTYRM-------------------------------------------------------------------------------------------------------------------------------------ggal_igf-1/1-153                          ---MEKINSLSTQLVKCCFCDF-LKVKMHTVSYIHFFYLGLCLLTL--------TS---SAAAGPETLCGAELVDAL---QFVCGDR------GFYFSKPTGY------------------------------GSSSRRLHHKGIVD-ECCFQ-SCDLR-R-LEM-YCAPIKPPKSARSVRAQRHTDMPKAQKEVHLKNTSRG--------------------------------------NTGNRNYRM-------------------------------------------------------------------------------------------------------------------------------------locu_igf1_ENSLOCG00000015180/1-189        -----MSNRHTTEFFQWQLCDV-FKFEMYTLSCAHILYFILCVLCL--------TR---TAAAVPETLCGAELVDTL---QFVCGER------GFYFNKPTGY------------------------------GSNTRRPHNRGIVD-ECCFQ-SCELW-R-LEM-YCAPVKPGKSARSVRAQRHTDVPKTQKVPGVIPNMPVLVTLGTTHSPQGLQSISLQKNVSGNSHPSCKEVHLKNSSRGNRNYRM-------------------------------------------------------------------------------------------------------------------------------------locu_igf2b_ENSLOCG00000001806/1-217       -MEDQQKYSYQAFCHTCLGTEN-RRMKMRKMSTSRQMLVFTIALTFYI-------MDVAKPFSYAETLCGGELVDAL---QFVCEDR------GFYFSRPT-----------------------------S---RSNSRRAQKGIVE-ECCFR-SCDLN-L-LEM-YCAKPAKSERDVSSTSLQGIPALPALKEAPRKPLTVKYSKYDVWQRKAAQRLRRGIPAILRARKFRHQAEKVKAQEQLL-FHKPL--------------------------------------------------------------------------------------------------------------ITLPSKLPPAVQSSTEKSVSHKlocu_novel_ENSLOCG00000011459/1-206       ------MKSSGHVSKPVCMASS-STGQRALKVSWIRRLWVTYAVLC--------FLALPQRGTATKSRCGRELLADL---EFVCGDR------GYYRGSKPLD-----------------RQVLSLPVIAGNAQGYGRRLRGKGIVE-QCCLR-GCDLQ-H-LES-YCAKPQRSRRLAPSVEPHSQEGKLREIFRKHVLAPSRNLESIRLNKVVALGNRNLPEPGAATSVPRISRHSFLLKEISRKDVERQP-----------------------------------------------------------------------------------------------------------------------------------locu_insulin-like_ENSLOCG00000009877/1-111 ------------------------------MARRFQVFSLLALLVL--------TSPPVTDAAANQHLCGSHLAEAL---YLVCGER------GFTYDPDKM-SGTEPGLG--LLT---GKAGEENVVDEYPFKEQGEMKVKRGIIE-QCCHK-PCTIY-E-LES-YCN----------------------------------------------------------------------------------------------------------------------------------------------------------------------------------------------------------------------locu_insulin-like_ENSLOCG00000009901/1-133 ---------MTRTGAGSYLCTTNLSGSTGIMALWFQVFSLLALLVL--------SSPPVTNAAANQHLCGSHLVEAL---YLVCGEK------GFFYNPNKAKRDMEPVLG--FLT---GKSGQENEVDEYQFKQQGEMKVKRGIVE-QCCHK-PCTIY-E-LEN-YCN----------------------------------------------------------------------------------------------------------------------------------------------------------------------------------------------------------------------ggal_isn/1-107                            ------------------------------MALWIRSLPLLALLVF--------SGPGTSYAAANQHLCGSHLVEAL---YLVCGER------GFFYSPKARRDVEQPLVS--S--------PLRGEAGVLPFQQEEYEKVKRGIVE-QCCHN-TCSLY-Q-LEN-YCN----------------------------------------------------------------------------------------------------------------------------------------------------------------------------------------------------------------------hsap_insulin/1-110                        ------------------------------MALWMRLLPLLALLAL--------WGPDPAAAFVNQHLCGSHLVEAL---YLVCGER------GFFYTPKTRREAEDLQVG--QVE-----LGGGPGAGSLQPLALEGSLQKRGIVE-QCCTS-ICSLY-Q-LEN-YCN----------------------------------------------------------------------------------------------------------------------------------------------------------------------------------------------------------------------mmus_ins1/1-108                           ------------------------------MALLVHFLPLLALLAL--------WEPKPTQAFVKQHLCGPHLVEAL---YLVCGER------GFFYTPKSRREVEDPQVE--QLE-----LGGS--PGDLQTLALEVARQKRGIVD-QCCTS-ICSLY-Q-LEN-YCN----------------------------------------------------------------------------------------------------------------------------------------------------------------------------------------------------------------------mmus_ins2/1-110                           ------------------------------MALWMRFLPLLALLFL--------WESHPTQAFVKQHLCGSHLVEAL---YLVCGER------GFFYTPMSRREVEDPQVA--QLE-----LGGGPGAGDLQTLALEVAQQKRGIVD-QCCTS-ICSLY-Q-LEN-YCN----------------------------------------------------------------------------------------------------------------------------------------------------------------------------------------------------------------------blan_BL01444_IGF2/1-307                   ------------------------------MNLSSVYVLASLAVVC--------LL---VKETQAEYLCGSTLADVL---SFVCGNR------GYNSQPRRSLGKRAID-----F--------ISEQEAKDYMGAMPHIRRRRGLVE-ECCYN-VCDYS-Q-LES-YCNPYTTAPATATPVRT--APPEEQEEEQQDEDAAPLDGMVGDQAPLGSIENIENLVYHYDRDDITVDAAKKEPK-KLKEILGSFEDKKANPVFPFIRQS---KNVKPNKFPDSSAHQYPTDLVEEGPTNEIPESPSQKPTLERLGYKDNQTDKKEPAENNNNNNRARDNRTKSSTVEPHTVPDYISKQYTHKPLITLPRGTPRRIESPGQLSLN--bbel_LOC109477811/43-355                  --------------------------QATGMNLSSVYVLASLAVVC--------LL---VKETRAEYLCGSTLADVL---SFVCGNR------GYNSQPRRSLGKRAID-----F--------ISEQQAKDYMGSMPPVRRRRGLVE-ECCLN-VCDYS-H-LES-YCNPHPTAPATASPVRT--TESQP-EEQERDEELAPLDGMVGDQAPLGSIENIENLVYHYDRDDITVDAAKKEPKMKLKEILGSFEDKKANPVFPFIRQSTKNQNVKPNKFPDSSAHQYPTDLVEEGPTNEIPETPSQKPTLERLGYKDNQTDKKEPAENN-NNNRARDNRTKSSTVEPHTVPDYISKQYTHKPLITLPRGTPRRIDAPGQLSLN--bflo_BRAFLDRAFT_121099/41-332             --------------------------QATGMNLSSVYVLASLAVVC--------LL---VKETQAEYLCGSTLADVL---SFVCGNR------GYNSQPRRSA---QVD-----F--------ISEQQAKDYMGAMPHIRRRRGLVE-ECCYN-VCDYS-Q-LES-YCNPYTTAPATATPVRT--TEPQPE-----EAEDDPLDGMVGDQAPLGSIENIENLVYHYDSDDITIDAAKKEPK-KLKEILGSFEDKKANPVFPFIRQS---KNIKPNKFPDNFAHQYPTDLVEEGPTNEIPESPSQKPTLERLGYKHNQTDKKQPTENNNNNNRARDNRTKSSTVEPHTVPEYISKQYTHKPLITLPRVNVR-------------cint_ins-l3_DQ665317/1-110                -------------MSETSRVVS-ATRPSSGGTYRRLCLVTCIVVTCM---AIFPGSSIAHGRKRFVLSCGSRLVNSM---RYMCLQE------VWTPKL--------------------------------KKLSKHMRRTKRFIAK-RCCSE-ICSVI-V-LRQ-FCLGDET------------------------------------------------------------------------------------------------------------------------------------------------------------------------------------------------------------------cint_ins-l2_DQ643991/1-84                 ------------------------------------MKIVIVVLIVLF-------IITPSPVESWSGACGARLINRL---RFICGER------GVFNPKHL-----------------------------HNNRRRLARRVRRDLVT-ECCRG-SCNRKWI-LRT-YCG----------------------------------------------------------------------------------------------------------------------------------------------------------------------------------------------------------------------obim_Ocbimv22011416/1-129                 -----------MEKIIKMFTWN-IVFLLFVTTLSRYTVKAGLEHRC--------NDETISRTSVSSSYCAAKMPNFL---RLVCNRE------EYGKMDEILFYTTRCPKP-----KRSKLNGVVISKKKAKSHLTRKSNFWCGIVC-ECCHH-TCSLD-E-LLA-YC-----------------------------------------------------------------------------------------------------------------------------------------------------------------------------------------------------------------------cgig_CGI_10012542_INS/1-140               -----MSCRNWCNSWEIMPVNR-KHQIFILLCLHFTSVQSDFERVC--------NSQTDLRGPDPQGICGRLIPEML---HLVCGGQ------YYVPSKRDNVDFPRYSPL-----EGLILGKREASMYLTSQHSRTKRNAYQGIVC-ECCYH-GCNWF-E-LQQ-YCGFRKK------------------------------------------------------------------------------------------------------------------------------------------------------------------------------------------------------------------lgig_LotgiG232734/1-118                   ----------------MAESVM-WKLLLSLCILVGTPVFAGYERSC--------NLERQ--QEDPRGLCGSQLESIL---HNVCFYL------NKRASPKERVKKRMD--------QSIKLDKFSALS---FLVKRGDGFGNTGITC-ECCLN-RCNLI-E-LSS-YCIL---------------------------------------------------------------------------------------------------------------------------------------------------------------------------------------------------------------------blan_BL13734_IGF1/1-113                   ---------------MNDRSLY-TSVLQTTMSPSGVLLMTCLLMI---------GA---ATPARSAYLCGSTLFDVL---SWVCEER------GYTAPEKNPD-----V-----D--------NVENEARIHPRSPQFSRRVQKLID-DCCFN-VCDFD-T-LES-YCNPWAE------------------------------------------------------------------------------------------------------------------------------------------------------------------------------------------------------------------bflo_novel/1-106                          ---------------------H-TSVLQTTMSPSGVLLMTCLSLI---------GC---AAPASSAYLCGSTLFDVL---SWVCEGR------GEPGVNK-ND-----V-----P--------NVDNEARLHPRSPQFSRRVRELID-DCCFN-VCTFD-T-LES-YCTPWAE------------------------------------------------------------------------------------------------------------------------------------------------------------------------------------------------------------------bbel_LOC109477594/1-75                    ------------------------------MSPSRVLLMTCLSLI---------GL---T-LANGEYLCGSTLKDVM---SFVCGRR------GHRARL-----------------------------------------NDHKLIE-DCCLN-VCDYE-Y-LES-YCNPSPR------------------------------------------------------------------------------------------------------------------------------------------------------------------------------------------------------------------lgig_LotgiG169325/1-120                   ---------------MEVTCKC-PLVLLGVLFLNFGTVLTHLEWTC--------TLETK--RESPRGVCGQRLPEVL---SMVCKRY------GGYRDTWGTN--SRL--------GNIILGKRDAFS---YLGKRGQSYGEQGITC-ECCYH-SCSFR-E-LRQ-YCRNSQQ------------------------------------------------------------------------------------------------------------------------------------------------------------------------------------------------------------------tcas_LOC103315192_INS/1-95                -----MKRHLTPPGWL-------------TMNVPKLWLKVCFTLLL--------AGQIHANIDRKEFFCGKKLVKTL---TELCAIY------NYPTLPRRR--------------------------------------FRRQIVD-ECCRS-QCSRR-YLVQY-YCMEAH-------------------------------------------------------------------------------------------------------------------------------------------------------------------------------------------------------------------spur_SPU_007203_igf1/27-172               QRFRLKYLRFVYDNTKELRDNV-KRKLEPSNRVIVKDFRAYGKMVCFRY-----PVAVRHVTASFPLLCGQELVKAV---AAVCNDR------GYYGQPSKRSAGIFELE-------TRAKTFLKSGMSRGETRRSKRGARTGLIVT-ECCLN-RCSVS-H-LES-YCNPLPP------------------------------------------------------------------------------------------------------------------------------------------------------------------------------------------------------------------skow_LOC100313638_ilpgf/1-113             ------------------------------MVRCMECLCVCLAVAV--------FT---TDVMAWDKLCGRTLVDVL---ALICNGR------GYNSGSPKKKVKRESP-----FRSGMEANDFFGNISSKEKRRQRRRSGSGKIVD-ECCHQ-ACDYT-T-LES-YCAPLPE------------------------------------------------------------------------------------------------------------------------------------------------------------------------------------------------------------------dmel_ilp6/1-99                            -------------------------MVLKVPTSKVL--LVLATLFAVAA--MIASPLAPTEYEQRRMMCSTGLSDVI---QKICVSG------TVALGDVF-----------------------------PNSFGKRRKRDLQNVTD-LCCKSGGCTYR-E-LLQ-YCKG---------------------------------------------------------------------------------------------------------------------------------------------------------------------------------------------------------------------tcas_LOC100142381_LIRP/1-127              ------------------------------MDLQYVLVVVATVLAGIH-TCRTDEMANFRGTKSKAVYCGRRLSETL---STVCKGN------YNTLNKKEMGASRRPG-YPSLSQHSLDYPYQSKANAASHHMSGFRRRKRRGVFN-ECCEK-PCSLE-E-LSQ-YCGGPSR------------------------------------------------------------------------------------------------------------------------------------------------------------------------------------------------------------------obim_Ocbimv22037295/1-127                 ------------------MKRI-RWQEASIKAVFYISVLFNLLIDC-------FQHWRNVWNQECFRRCRAELGPHI---SIACQND------IYKIINKRRRHRRSLRFPFLLQDSEAFSYLKRE----H---TMVKRGQAGGIME-ECCYMKGCTWE-E-YAE-FCHSHSR------------------------------------------------------------------------------------------------------------------------------------------------------------------------------------------------------------------lgig_LotgiG155605/1-135                   ------------------MERN-GIFTFKLQQINVILSTLLIIILTLTKLVQTPEDLLALWHTDCHRRCRSQLIEHV---NIACIFD------PYKIYRKNDSNKTRN---LFIDKSRATTFLATRDTGRK---RKGHSIKKRGVME-ECCYNKSCSWE-E-YAE-YCHSHNR------------------------------------------------------------------------------------------------------------------------------------------------------------------------------------------------------------------dmel_ilp7/1-135                           ------------------MTRM-IIQNSGSWTLCGAVLLFVLPLIP------TQSDWENVWHQETHSRCRDKLVRQL---YWACEKD------IYRLTRRNKRTGNDEAWIKKTTTEPDGSTWLHVNYANMFLRSRRSDGNTPSISN-ECCTKAGCTWE-E-YAE-YCPSNKR------------------------------------------------------------------------------------------------------------------------------------------------------------------------------------------------------------------dmel_ilp8/1-100                           -------------------------------------------------------------MSSKLHMCRWMLLVIG---VCCLMGS------SSGSFCSLEFAHHHLNRLGSGKTHNKHHYISRSSYPMGGYLKVTREHFNRLSEL-DIFPR-------------YKPIKPHHEKKHRFKRDH-------------------------------------------------------------------------------------------------------------------------------------------------------------------------------------------------------cint_ins-l1_DQ538510/14-136               NNFQRDVLFNEELLEKNEPSYI-VPSLRHCCRRRITVSVITYAFVL--------LY--VSSHCEAEYLCGSRLVDAL---RFICGSR------GINGPGRGSY-------------------------MHARRGSHLRKRPEADITV-LCCER-GCTMK-Q-MER-FCGEPGR------------------------------------------------------------------------------------------------------------------------------------------------------------------------------------------------------------------spur_SPU_030139_igf2/1-111                ------------------------------MDPFRVLLYMV-------T-----FLLYVVGPISSFRLCGRELADAL---AVVCKGR------GYYIDDSEIAQKDSPIVP------HHVASSFLGSSSASAHSRQRRRVRTGQIVN-ECCDK-ECSNN-I-MES-YCNRRTP------------------------------------------------------------------------------------------------------------------------------------------------------------------------------------------------------------------lgig_LotgiG154465/1-129                   ------MDVEDETIWLDCTPGI-QSIRRQKMKTYYQLVALAIFLSL--------GT--ITEQAEPERLCGRNLANAL---DLVCYDR------GFHWEVSKRSDINHP-------------AKRRNRRMIDVFIDKRNYHGRRGVVE-ECCYI-GCTYE-T-LES-YCAEPGD------------------------------------------------------------------------------------------------------------------------------------------------------------------------------------------------------------------dmel_ilp2/1-117                           ----------------------------MSKPLSFISMVAVIL------------LASSTVKLAQGTLCSEKLNEVL---SMVCEEY------NPVIPHKRFEEEDNSISE--PLRSALFPGSYLGGVLNSLAEVRRRTRQRQGIVE-RCCKK-SCDMK-A-LRE-YCSVVRN------------------------------------------------------------------------------------------------------------------------------------------------------------------------------------------------------------------dmel_ilp4/1-112                           ------------------------------MSLIRLGLAL----LLL----LATVSQLLQPVQGRRKMCGEALIQAL---DVICVNG------FTRRVRRIEQET-ETGRLKQKHTDADTEKGVPPA----VGSGRKLRRHRRRIAH-ECCKE-GCTYD-D-ILD-YCA----------------------------------------------------------------------------------------------------------------------------------------------------------------------------------------------------------------------dmel_ilp1/1-135                           -----------------MFSQH-NGAAVHGLRLQSLLIAAMLTAAMA----MVTGSGHQLLPPGNHKLCGPALSDAM---DVVCPHG------FNTLPRKRQDDSSMWQTLDGAGYSFSPLLTNLYGSEVLIKMRRHRRHLTGGVYD-ECCVK-TCSYL-E-LAI-YCLPK--------------------------------------------------------------------------------------------------------------------------------------------------------------------------------------------------------------------dmel_ilp3/1-119                           -----------------------MGIEMRCQDRRILLPSLLLL------------ILMIGGVQATMKLCGRKLPETL---SKLCVYG------FNAMTKRTLDPV-----NFNQIDGFEDRSLLERLLSDSSVQMLKTRRLRDGVFD-ECCLK-SCTMD-E-VLR-YCAAKPR------------------------------------------------------------------------------------------------------------------------------------------------------------------------------------------------------------------dmel_ilp5/1-108                           -----------------------------MMFRSVIPVLLFLI-----------PLLLSAQAANSLRACGPALMDML---RVACPNG------FNSMFAKRGTLG--------LFDYEDHLADLDSSESHHMNSLSSIRRDFRGVVD-SCCRK-SCSFS-T-LRA-YCDS---------------------------------------------------------------------------------------------------------------------------------------------------------------------------------------------------------------------cele_ins15/1-81                           ------------------------------MKLLPLIVVFALLAVISE----SYDFQPRDNKHHSYRSCGESLSRRV---AFLCNGG------AIQT--------------------------------------------EILRAL-DCCST-GCTDK-Q-IFS-WCDFRKL------------------------------------------------------------------------------------------------------------------------------------------------------------------------------------------------------------------cele_ins38/1-76                           ------------------------------MNLFLLVCIAFAII----TVTSFDEKSQRSHVFSYKKHCGRRIVSLV---QA-CD------------------------------------------------------RIDHDLSI-DCCTQ-NCSSE-F-VKK-IMCPSKL------------------------------------------------------------------------------------------------------------------------------------------------------------------------------------------------------------------cele_ins22/1-71                           ------------------------------MHTTTILICFFIFLV-------QVSTMDAHTDKYVRTLCGKTAIRNI---ANLCPPKPEMKGICSTG--------------------------------------------EYPSIT-EYCSM-GFS----------------------------------------------------------------------------------------------------------------------------------------------------------------------------------------------------------------------------------cele_ins21/1-65                           ------------------------------MKTYSFFVLFIVFIF-------FISSSKSHSKKHVRFLCATKAVKHI---RKVCPDM------CLTG--------------------------------------------EEVEVN-EYCKM-GYS----------------------------------------------------------------------------------------------------------------------------------------------------------------------------------------------------------------------------------cele_ins16/1-72                           ------------------------------MQS----LPILACLL-------TLSVFAPEIHGRELKRCSVKLFDIL---SVICGTE------SDAE-------------------------------------------ILQKVAV-KCCQE-QCGFE-E-M----CQHANL------------------------------------------------------------------------------------------------------------------------------------------------------------------------------------------------------------------cele_ins12/1-79                           ------------------------------MQSNITASLFIALLI-------FGVISAAPSHEKTHKKCSDKLYLAM---KSLCSYR------GYSE-------------------------------------------FLRNSAT-KCCQD-NCEIS-E-MMA-LCVVAPN------------------------------------------------------------------------------------------------------------------------------------------------------------------------------------------------------------------cele_ins13/1-73                           ------------------------------MKLLHIFIIFLLFQSCSNK--------MCQYSKKKYKICGVRALKHM---KVYCTRG------MTR--------------------------------------------DYGKLLV-TCCSK-GCNAI-D-IQR-ICL----------------------------------------------------------------------------------------------------------------------------------------------------------------------------------------------------------------------cele_ins14/1-73                           ------------------------------MLTHLKFLLLVSLF-------------INFAVSSEDIKCDAKFISRI---TKLCIHG------ITED-------------------------------------------KLVRLLT-RCCTS-HCSKA-H-LKM-FCTLKPH------------------------------------------------------------------------------------------------------------------------------------------------------------------------------------------------------------------cele_ins31/1-82                           ------------------------------MKMPLILLLLVAAA------SAFVHHFDHSMFARPEKTCGGLLIRRV---DRICPNL------NYTY------------------------------------------KIEWELMD-NCCEV-VCEDQ-W-IKETFCRAPRF------------------------------------------------------------------------------------------------------------------------------------------------------------------------------------------------------------------cele_ins37/1-85                           ------------------------------MAAFLPIALSIAMLTVLTN--ANPIHPVPNAAFLPYRSCGSHLVHRA---FEACSGK------KDRS-------------------------------------------SDVDLWK-MCCKD-ECTDL-DIKES-LCKYASQ------------------------------------------------------------------------------------------------------------------------------------------------------------------------------------------------------------------cele_ins18/1-73                           ------------------------------MVHRLFIVLIAIIL---------VAKSTAISLDGRMKMCPPGGSTFTMAWSMSCS------------------------------------------------------MRRRQLQT-ICCQV-GCNVE-D-LLA-YCAPI--------------------------------------------------------------------------------------------------------------------------------------------------------------------------------------------------------------------cele_ins1/1-85                            ------------------------------MYWFRQVYRPSFFFGFL----AILLLSSPTPSDASIRLCGSRLTTTL---LAVCRNQ------LCT----------------------------------------GLTAKRGGIAT-ECCEK-RCSFA-Y-LKT-FCCNQDD------------------------------------------------------------------------------------------------------------------------------------------------------------------------------------------------------------------cele_ins11/1-84                           ------------------------------MSSYRQTLFILIILIVIIL--FVNEGQGAPHHDKRHTACVLKIFKAL---NVMCNHE------GDAD-------------------------------------------VLRRTAS-DCCRE-SCSLT-E-MLA-SCTLTSS------------------------------------------------------------------------------------------------------------------------------------------------------------------------------------------------------------------cele_ins29/1-75                           ------------------------------MFCKFVFLIFL-----------LISLSVATADFGAQRRCGRHLVNFL---EGLCGGP------CSEA--------------------------------------------PTVELASWACSS-AVSIQ-D-LEK-LCCPSNL------------------------------------------------------------------------------------------------------------------------------------------------------------------------------------------------------------------cele_ins25/1-72                           ------------------------------MLFKIIILFFL-----------L--LQLSEAKPEAQRRCGRYLIRFL---GELCNGP------CSGV--------------------------------------------SSVDIATIACAT-AVPIE-D-LKN-MCCPNL-------------------------------------------------------------------------------------------------------------------------------------------------------------------------------------------------------------------cele_ins17/1-82                           ------------------------------MFSTRGVLLLLSLMAAVAAFGLFDTIRPPRAKHGSLKLCPPGGASFLDAFNLICP------------------------------------------------------M-RRRTMN-MCCET-GCEFT-D-IFA-ICNPFG-------------------------------------------------------------------------------------------------------------------------------------------------------------------------------------------------------------------cele_ins28/1-67                           ------------------------------MMRSFFVLLALLA----------------IVTSTASPTCGRALLHRI---QSVCGLC------TIDA--------------------------------------------HHELIA-IACSR-GLGDK-E-IIE-MCCPI--------------------------------------------------------------------------------------------------------------------------------------------------------------------------------------------------------------------cele_ins27/1-76                           ------------------------------MKFFRLILLCA--LV-------LTTMAFLAPSTAAKRRCGRRLIPYV---YSICGGP------CENG---------------------------------------------DIIIEHCFSGT-TPTIA-E-VQK-ACCPELS------------------------------------------------------------------------------------------------------------------------------------------------------------------------------------------------------------------cele_ins2/1-79                            ------------------------------MNAIIFCLLFTTVTAT------YSTPTPNRASRVQKRLCGRRLILFM---LATCGEC-------DT-------------------------------------------DSSEDLSH-ICCIK-QCDVQ-D-IIR-VCCPNSF------------------------------------------------------------------------------------------------------------------------------------------------------------------------------------------------------------------cele_ins10/1-85                           ------------------------------MSLHFSTIQKTILLISFLL---LVTLAPRTSAAFPFQICVKKMEKMC---RIINPEQ------CAQV----------------------------------------NKITEIGALT-DCCTG-LCSWE-E-IRI-SCCSVL-------------------------------------------------------------------------------------------------------------------------------------------------------------------------------------------------------------------cele_ins36/1-85                           ------------------------------MNIGKCSIIFLLFCV--FGSILSAIRKRHPEGKLVIRDCKRYLIMYS---RTICKEK------CEKF-------------------------------------------DEDLLVEGCHSNQ-TLSNE-R-TRE-LCCPNAG------------------------------------------------------------------------------------------------------------------------------------------------------------------------------------------------------------------cele_ins35/1-82                           ------------------------------MKQIFLVILAA--CL--LAIILAHHKMDENAFGINNRHCQRALKVYS---FAICGAI------CQNY--------------------------------------------EKILMEGCGSTV-MLTMQ-R-TKL-ICCPEPV------------------------------------------------------------------------------------------------------------------------------------------------------------------------------------------------------------------cele_ins9/1-81                            ------------------------------MIVTLIVFLV-----IGLQMAHLGNRARRTLETEKIYRCGRKLYTDV---LSACNGP------CEP-------------------------------------------GTEQDLSK-LCCGN-QCTFV-E-IRK-ACCADKL------------------------------------------------------------------------------------------------------------------------------------------------------------------------------------------------------------------cele_ins33/1-85                           ------------------------------MANTCLILLLLLVIFVTVGFSMPEQRRHRRHRHHGQKHCGTKIVRKL---QMLCPKM------CTIS--------------------------------------------DDTLLT-EMCSH-SLFDD-E-IQL-RCCPKED------------------------------------------------------------------------------------------------------------------------------------------------------------------------------------------------------------------cele_ins20/1-83                           ------------------------------MRNSFQLILLVLTISYSNSMLFNKEPKHHHHHHRHKGYCGVKAVKKL---KQICPDL------CSNV--------------------------------------------DDNLLM-EMCSK-NLTDD-D-ILQ-RCCPE--------------------------------------------------------------------------------------------------------------------------------------------------------------------------------------------------------------------cele_ins4/1-83                            ------------------------------MFSFFTYFLLSALLLSASCR--QQLSRARRVPAGEVRACGRRLLLFV---WSTCGEP------CTP-------------------------------------------QEDMDIAT-VCCTT-QCTPS-Y-IKQ-ACCPEK-------------------------------------------------------------------------------------------------------------------------------------------------------------------------------------------------------------------cele_ins7/1-83                            ------------------------------MPPIILVFFLVLIPASQQYPFSLSSRTRRVPDEKKIYRCGRRIHSYV---FAVCGKA------CES-------------------------------------------NTEVNIAS-KCCRE-ECTDD-F-IRK-QCCP---------------------------------------------------------------------------------------------------------------------------------------------------------------------------------------------------------------------cele_ins8/1-81                            ------------------------------MSPIILIFFLVFIPFSQQHT--SLPSRVRRVPEQKNKLCGKQVLSYV---MALCEKA------CDS-------------------------------------------NTKVDIAT-KCCRD-ACSDE-F-IRH-QCCP---------------------------------------------------------------------------------------------------------------------------------------------------------------------------------------------------------------------cele_ins5/1-85                            ------------------------------MHSIVALMLIGTILPIAALHQKHMDAISRADRHTNYRSCALRLIPHV---WSVCGDA------CQP-------------------------------------------QNGIDVAQ-KCCST-DCSSD-Y-IKE-ICCPFD-------------------------------------------------------------------------------------------------------------------------------------------------------------------------------------------------------------------cele_ins3/1-84                            ------------------------------MKLSVVLALFIIFQLGAASLMRNHSLMARSRRGDKVKICGTKVLKMV---MVMCGGE------CSS--------------------------------------------TNENIAT-ECCEK-MCTME-D-ITT-KCCPSR-------------------------------------------------------------------------------------------------------------------------------------------------------------------------------------------------------------------cele_ins34/1-92                           ------------------------------MLHHKTLIIALLLTLFISGIDSLTVKTTAAPLAQVNPQCLRRLTLLA---RGVCRQP------CQPSDKPK-------------------------------------TSAQQLLQL-ACSAR-RPTNE-Q-IIS-YCCPEKS------------------------------------------------------------------------------------------------------------------------------------------------------------------------------------------------------------------cele_ins30/1-85                           ------------------------------MSSHALVLFLLLFLLPVALGHFLAPREPVVAAQGAKKTCGRSLLIKI---QQLCHGI------CTVH--------------------------------------------ADDLHE-TACMK-GLTDS-Q-LIN-SCCPPIP------------------------------------------------------------------------------------------------------------------------------------------------------------------------------------------------------------------cele_ins24/1-82                           ------------------------------MRSPTLF--LLLLLVPLALCHVFKEMGLIRANQGPQKACGRSMMMKV---QKLCAGG------CTIQ--------------------------------------------NDDLTI-KSCST-GYTDA-G-FIS-ACCPSG-------------------------------------------------------------------------------------------------------------------------------------------------------------------------------------------------------------------cele_ins6/1-83                            ------------------------------MNSVFTIIFVLCALQVAASFRQSMPRARRVPAPGETRACGRKLISLV---MAVCGDL------CNP-------------------------------------------QEGKDIAT-ECCGN-QCSDD-Y-IRS-ACCP---------------------------------------------------------------------------------------------------------------------------------------------------------------------------------------------------------------------cele_ins26/1-81                           ------------------------------MRALVAILCLMAL----CHAAMLGLIGNHHHGTKAGLTCGMNIIERV---DKLCNGQ------CTRN--------------------------------------------YDALVI-KSCHR-GVSDM-E-FMV-ACCPTMK------------------------------------------------------------------------------------------------------------------------------------------------------------------------------------------------------------------cele_ins39/1-78                           ------------------------------MNTFFFLAVLLVFCS-------ATQPLAIYLNISTPQDCIHKIFRMT---ISFCSQV------ECQN---------------------------------------------MEAMQKICNTT-TPTIK-H-VGE-LCCPEFF------------------------------------------------------------------------------------------------------------------------------------------------------------------------------------------------------------------cele_ins23/1-76                           ----------------------------------MFVLLIILSII-------LAQVTDAHSELHVRRVCGTAIIKNI---MRLCPGVPA----CENG--------------------------------------------EVPSPT-EYCSM-GYSDS-Q-VKY-LCCPTSQ------------------------------------------------------------------------------------------------------------------------------------------------------------------------------------------------------------------cele_ins19/1-90                           ------------------------------MIFYLTTYLVTMSPLFLILLLLVSESYEVLMLFGYKRTCGRRLMNRI---NRVCVKD------IDPAD----------------------------------------IDPKIKLSE-HCCIK-GCTDG-W-IKK-HICSEEVL-----------------------------------------------------------------------------------------------------------------------------------------------------------------------------------------------------------------cele_ins32/1-80                           ------------------------------MTSILLILLLVITVTG-----MFSSSLAVKSRSRRELICGRRLSKTV---TNLCVEM-------NP-------------------------------------------QKEEDIAT-KCCKNKGCSRE-Y-IKS-IMCPDE-------------------------------------------------------------------------------------------------------------------------------------------------------------------------------------------------------------------hvul_ilp1_LOC100205786/1-115              ------------------------------MLIEMQSSIIFLIIII------LGVLGGEEKPAHAQALCGNQFLLRW---KVLCQIE------SQKKANNLLKSMMKEQKHFLLKANTAKKFLNPL----KRKRRSIFSGKLDNADE-ECCKE-KCVTL-E-ILE-YPC----------------------------------------------------------------------------------------------------------------------------------------------------------------------------------------------------------------------hvul_ilp2/1-127                           ---------------------M-NFNNFTFMLFVWMCLTFDLYNLLL-------NKKENKIIQRELRVCTQAFLQSL---LKHLCVF------PNPVAKLIKKDTPITKKDFTLSNDVASNFLNKRDNTWFRHQYPDVYPTDINVHD-ECCYNKGCVVD-E-IME-YCN----------------------------------------------------------------------------------------------------------------------------------------------------------------------------------------------------------------------hvul_ilp3_LOC100202208/1-125              --------------------------MYDKMTVVCLCLLLHISERIAYGETIENDFIPKKKEANEWRVCSHRSFLFI---LKYVCNI------NNKPLSWSKKERQFTKKELVIGGNQATSFLNT-NYHWLNAG----FSTDVDFID-ECCNLKGCNSN-E-ISE-YCN----------------------------------------------------------------------------------------------------------------------------------------------------------------------------------------------------------------------	b. Full length, more vertebratesDrer_INSb_ENSDARG00000034610/1-160        MGTFVFPLMPSRAMGQAAGRYISSTIQAQSWPLFTDSAHSGKGCACPQSV-------------------------------------------------------------STMVLLLQASVLILLLASLPG-SQ----------------------------------------------------------------------------SSPSQHLCG-SSLVDALYLVCGPRGFFYT-NR----------------------------------------------------------------------------------------GRR------------------------DLETLLALLSNL------------AGYEA--ADADPLKE----KVMKMKRG-------IVEQCC-HRPCTIY-HLEDYCS---------------------------------------------------------------------------------------------------------------------------------------------------------------------------------------------------------------------------------------------------------------------------------------------------------------------------------------------------------------------------------------------------------------------------Drer_INS_ENSDARG00000035350/1-109         ----------------------------------------------------------------------------------------------------------------MAVWLQAGALLVLLVVSSVS-TN----------------------------------------------------------------------------PGTPQHLCG-SHLVDALYLVCGPTGFFYN-PK------------------------------------------------------------------------------------------R------------------------DVEPLLGFLPPK------------SAQETEVADFAFKDH----AELIRKRG-------IVEQCC-HKPCSIF-ELQNYCN---------------------------------------------------------------------------------------------------------------------------------------------------------------------------------------------------------------------------------------------------------------------------------------------------------------------------------------------------------------------------------------------------------------------------Gacu_INS_ENSGACG00000001771/1-118         ---------------------------------------------------------------------------------------------------------------MASLWLQSVSLLVLLVWSCPGSQA----------------------------------------------------------------------------AAGPQHLCG-SHLVDALYLVCGERGFFYN-PK------------------------------------------------------------------------------------------R------------------------DVDPLMGFLPPK-----VGGASAAAGGENEVAEFAFKDQ----MEMMVKRG-------IVEQCC-HRPCNIF-DLQNYCN---------------------------------------------------------------------------------------------------------------------------------------------------------------------------------------------------------------------------------------------------------------------------------------------------------------------------------------------------------------------------------------------------------------------------Lcha_INS_ENSLACG00000006484/1-112         ----------------------------------------------------------------------------------------------------------------MALWVRVLPLFLLIALSAPSTTQ----------------------------------------------------------------------------AIANQHLCG-SHLVEALYLVCGEKGFFYS-PR----------------------------------------------------------------------------------------GRR------------------------EIEQSLTGALCF------------TGVCSAGYIYILMQQ----GTMKEKRG-------IVEQCC-HNTCSLY-QLENYCN---------------------------------------------------------------------------------------------------------------------------------------------------------------------------------------------------------------------------------------------------------------------------------------------------------------------------------------------------------------------------------------------------------------------------Hsap_insulin/1-110                        ----------------------------------------------------------------------------------------------------------------MALWMRLLPLLALLALWGPDPAA----------------------------------------------------------------------------AFVNQHLCG-SHLVEALYLVCGERGFFYT-PK----------------------------------------------------------------------------------------TRR------------------------EAEDLQVGQVEL------------GGGPG-AGSLQPLAL----EGSLQKRG-------IVEQCC-TSICSLY-QLENYCN---------------------------------------------------------------------------------------------------------------------------------------------------------------------------------------------------------------------------------------------------------------------------------------------------------------------------------------------------------------------------------------------------------------------------Mmus_ins1/1-108                           ----------------------------------------------------------------------------------------------------------------MALLVHFLPLLALLALWEPKPTQ----------------------------------------------------------------------------AFVKQHLCG-PHLVEALYLVCGERGFFYT-PK----------------------------------------------------------------------------------------SRR------------------------EVEDPQVEQLEL------------GGSP---GDLQTLAL----EVARQKRG-------IVDQCC-TSICSLY-QLENYCN---------------------------------------------------------------------------------------------------------------------------------------------------------------------------------------------------------------------------------------------------------------------------------------------------------------------------------------------------------------------------------------------------------------------------Mmus_ins2/1-110                           ----------------------------------------------------------------------------------------------------------------MALWMRFLPLLALLFLWESHPTQ----------------------------------------------------------------------------AFVKQHLCG-SHLVEALYLVCGERGFFYT-PM----------------------------------------------------------------------------------------SRR------------------------EVEDPQVAQLEL------------GGGPG-AGDLQTLAL----EVAQQKRG-------IVDQCC-TSICSLY-QLENYCN---------------------------------------------------------------------------------------------------------------------------------------------------------------------------------------------------------------------------------------------------------------------------------------------------------------------------------------------------------------------------------------------------------------------------Ggal_isn/1-107                            ----------------------------------------------------------------------------------------------------------------MALWIRSLPLLALLVFSGPGTSY----------------------------------------------------------------------------AAANQHLCG-SHLVEALYLVCGERGFFYS-PK----------------------------------------------------------------------------------------ARR------------------------DVEQPLVS----------------SPLRGEAGVLPFQQE----EYEKVKRG-------IVEQCC-HNTCSLY-QLENYCN---------------------------------------------------------------------------------------------------------------------------------------------------------------------------------------------------------------------------------------------------------------------------------------------------------------------------------------------------------------------------------------------------------------------------Locu_insulin-like_ENSLOCG00000009877/1-111 ----------------------------------------------------------------------------------------------------------------MARRFQVFSLLALLVLTSPPVTD----------------------------------------------------------------------------AAANQHLCG-SHLAEALYLVCGERGFTYD-PD----------------------------------------------------------------------------------------KMS------------------------GTEPGLGLLTGK------------AGEENVVDEYPFKEQ----GEMKVKRG-------IIEQCC-HKPCTIY-ELESYCN---------------------------------------------------------------------------------------------------------------------------------------------------------------------------------------------------------------------------------------------------------------------------------------------------------------------------------------------------------------------------------------------------------------------------Locu_insulin-like_ENSLOCG00000009901/1-133 MTRT------------GAGSY-----------------------LCTTNLSGS-------------------------------------------TG-------------IMALWFQVFSLLALLVLSSPPVTN----------------------------------------------------------------------------AAANQHLCG-SHLVEALYLVCGEKGFFYN-PN---------------------------------------------------------------------------------K------AKR------------------------DMEPVLGFLTGK------------SGQENEVDEYQFKQQ----GEMKVKRG-------IVEQCC-HKPCTIY-ELENYCN---------------------------------------------------------------------------------------------------------------------------------------------------------------------------------------------------------------------------------------------------------------------------------------------------------------------------------------------------------------------------------------------------------------------------Xtro_INS_ENSXETG00000014029/1-107         ----------------------------------------------------------------------------------------------------------------MALWMQCLPLVLVLLFSTPN-TE----------------------------------------------------------------------------ALANQHLCG-SHLVEALYLVCGDRGFFYY-PK----------------------------------------------------------------------------------------IKR------------------------DIEQAMVN----------------GPQDNELDGMQLQPQ----EYQKMKRG-------IVEQCC-HSTCSLF-QLESYCN---------------------------------------------------------------------------------------------------------------------------------------------------------------------------------------------------------------------------------------------------------------------------------------------------------------------------------------------------------------------------------------------------------------------------Drer_IGF1_ENSDARG00000094132/1-162        MSSG----------------------------------HFFQGHWC------------------------------------------DVFK----CT-------------MRCLPSTHTLSLVLCVLALTPATL----------------------------------------------------------------------------EAGPETLCG-AELVDTLQFVCGDRGFYFS-KP----------------------------------------------------------------------------------------TGY------------------------------------------------------------GPS----SRRSHNRG-------IVDECC-FQSCELR-RLEMYCAP------VKT-----------------GKS--PRSLRAQRH-----------------------------------------------------------------------------TDIPRTPKKP--------------------------------------------------ISGHSHSS---CKEVHQKNSSRGNTG------------------GRNYRM---------------------------------------------------------------------------------------------------------------------------------------------------------------------------------------Gacu_IGF1_ENSGACG00000020042/1-182        MSS--------------------------------------QWHLC------------------------------------------DVFK----SA-------------MCCISCGRTLSLLLCVLSLTPTPT----------------------------------------------------------------------------GAGPETLCG-AELVDTLQFVCGERGFYFS-KP-----------------------------------------------------------------------------------------GY------------------------------------------------------------GPN----ARR--SRG-------IVDECC-FQSCELR-RLEMYCAP------PKT-----------------GQP--TRFVRAQRH-----------------------------------------------------------------------------TDLPRTPMVSTA-------------------VQKVDRSTERRTAQQ--PD--KTKNKKRPLPGHSHSS---FKEVHQKNSSRGNAG------------------GRNYRM---------------------------------------------------------------------------------------------------------------------------------------------------------------------------------------Lcha_IGF1_ENSLACG00000022367/1-137        M-----------------------------------------------------------------------------------------------KK-------------MCTRSYIHLFCLILCVLNLTHSTA----------------------------------------------------------------------------AAGSETLCG-AELVDTLQFVCGDRGFYFH-KA----------------------------------------------------------------------------------------TGY------------------------------------------------------------GSSG--RGRGQGHKG-------IVNECC-FQSCDLR-RLEMYCAP------PKT-----------------SKS--TRSVRAQRH-----------------------------------------------------------------------------TDMPKAQ-----------------------------------------------------------------KEVHLKNSSRGNTG------------------NRNYRM---------------------------------------------------------------------------------------------------------------------------------------------------------------------------------------Xtro_IGF1_ENSXETG00000002532/1-154        MEKNN-----------------------------SLSTQLFKCYFC------------------------------------------DFLK----LK-------------MHKMSYIHLLYLALCFLTLT-HSA----------------------------------------------------------------------------AAGPETLCG-AELVDTLQFVCGDRGFYFS-KP----------------------------------------------------------------------------------------TGY------------------------------------------------------------GSS----NRRSHHRG-------IVDECC-FQSCDFR-RLEMYCAP------AKP-----------------AKS--ARSVRAQRH-----------------------------------------------------------------------------TDMPKAQ-----------------------------------------------------------------KEVHLKNASRGNTG------------------SRGFRM---------------------------------------------------------------------------------------------------------------------------------------------------------------------------------------Hsap_igf-1/1-153                          MGKIS-----------------------------SLPTQLFKCCFC------------------------------------------DFLK----VK-------------MHTMSSSHLFYLALCLLTFT-SSA----------------------------------------------------------------------------TAGPETLCG-AELVDALQFVCGDRGFYFN-KP----------------------------------------------------------------------------------------TGY------------------------------------------------------------GSS----SRRAPQTG-------IVDECC-FRSCDLR-RLEMYCAP------LKP-----------------AKS--ARSVRAQRH-----------------------------------------------------------------------------TDMPKTQ-----------------------------------------------------------------KEVHLKNASRGSAG------------------NKNYRM---------------------------------------------------------------------------------------------------------------------------------------------------------------------------------------Mmus_igf1/1-153                           MGKIS-----------------------------SLPTQLFKICLC------------------------------------------DFLK----IK-------------IHIMSSSHLFYLALCLLTFT-SST----------------------------------------------------------------------------TAGPETLCG-AELVDALQFVCGPRGFYFN-KP----------------------------------------------------------------------------------------TGY------------------------------------------------------------GSS----IRRAPQTG-------IVDECC-FRSCDLR-RLEMYCAP------LKP-----------------TKA--ARSIRAQRH-----------------------------------------------------------------------------TDMPKTQ-----------------------------------------------------------------KEVHLKNTSRGSAG------------------NKTYRM---------------------------------------------------------------------------------------------------------------------------------------------------------------------------------------Ggal_igf-1/1-153                          MEKIN-----------------------------SLSTQLVKCCFC------------------------------------------DFLK----VK-------------MHTVSYIHFFYLGLCLLTLT-SSA----------------------------------------------------------------------------AAGPETLCG-AELVDALQFVCGDRGFYFS-KP----------------------------------------------------------------------------------------TGY------------------------------------------------------------GSS----SRRLHHKG-------IVDECC-FQSCDLR-RLEMYCAP------IKP-----------------PKS--ARSVRAQRH-----------------------------------------------------------------------------TDMPKAQ-----------------------------------------------------------------KEVHLKNTSRGNTG------------------NRNYRM---------------------------------------------------------------------------------------------------------------------------------------------------------------------------------------Locu_igf1_ENSLOCG00000015180/1-189        MSNR-------------------------------HTTEFFQWQLC------------------------------------------DVFK----FE-------------MYTLSCAHILYFILCVLCLT-RTA----------------------------------------------------------------------------AAVPETLCG-AELVDTLQFVCGERGFYFN-KP----------------------------------------------------------------------------------------TGY------------------------------------------------------------GSN----TRRPHNRG-------IVDECC-FQSCELW-RLEMYCAP------VKP-----------------GKS--ARSVRAQRH-----------------------------------------------------------------------------TDVPKTQKVPGV-------------------IPNMPVLVTLGTTHS--PQGLQSISLQKNVSGNSHPS---CKEVHLKNSSRG---------------------NRNYRM---------------------------------------------------------------------------------------------------------------------------------------------------------------------------------------Drer_IGF2b_ENSDARG00000033307/1-213       MEDQ--------------------------------LKHHSVCHTC---------------------------------------SRTDSFVNKV-IK-------------MFWSIRMPICILFLTLSAFE--------------------------------------------------------------------------------VASAETLCG-GELVDALQFVCEDRGFYFS-RP----------------------------------------------------------------------------------------TSR-------------------------------------------------------------SN----SRRSQNRG-------IVEECC-FSSCNLA-LLEQYCA--------KP-----------------AKS--ERDVSATSL-----------------------------------------------------------------------------QVIPVMPALK-------------------------------------------QEVPRK-HVTVKYSK---YDVWQRKAAQRLRRGIPAI------------LRAKKFRRQAERIKAQEQL-----------------------------LHHRP---------------------------LITLPSKLPP-ILLPTENYVSHK----------------------------------------------------------------------------------------Drer_IGF2a_ENSDARG00000018643/1-198       MDDY-----------------------------------HVFCASC---------------------------------------RKTEET--------------------RTTMRSLIVFVLSLSMLISN--------------------------------------------------------------------------------VTAGETLCG-GELVDTLQFVCGEDGFYIS-RP----------------------------------------------------------------------------------------NRS-------------------------------------------------------------------NSRRPQRG-------IVEECC-FRSCELH-LLQQYCA--------KP-----------------VKS--ERDVSSSSL-----------------------------------------------------------------------------QVFPVSQALH-------------------------------------------KD-----TINVKYSK---YEVWQQKAAQRLRRGVPSI------------LLARKFRRQMEKIQDEEQT-----------------------------SFHRP---------------------------LMTLPNRQPA-IVPHVQISTSRK----------------------------------------------------------------------------------------Gacu_IGF2B_ENSGACG0000001112/1-215        MDTQ------------------------------KRPGHHSRCHTC---------------------------------------RRTDMSRMKM-KK-------------MSSSSRALLLALALALYVVE--------------------------------------------------------------------------------MASAETLCG-GELVDALQFVCEDRGFYFS-RP----------------------------------------------------------------------------------------TSR-------------------------------------------------------------GN----NRRNQNRG-------IVEECC-FRSCDLN-LLEQYCA--------KP-----------------AKS--ERDVSATSL-----------------------------------------------------------------------------QVIPMMPALK-------------------------------------------QEVPRKQHVTVKYSK---YEVWQRKAAQRLRRG-PAI------------LRARRFRRQEEKIKAQEQA-----------------------------VFHRP---------------------------LISLPSKLPP-VLLATDDSLNHK----------------------------------------------------------------------------------------Lcha_IGF2a_ENSLACG00000004407/1-208       MEEY--------------------------------CTHPVVCQIC-----------------------------------TKEPEDSNSSNFKV-SK-------------MSTSRHLLLLSMAIIVYIADVAKA----------------------------------------------------------------------------FGPPETLCG-GELVDTLQFVCGDRGFYFS-RP----------------------------------------------------------------------------------------TGR-------------------------------------------------------------------STKRPNRG-------IVEECC-FRSCDLA-LLETYCA--------KA-----------------VKT--ERDLTSSSL-----------------------------------------------------------------------------QVLP---AVN-------------------------------------------KDVFRK-TSIARYSK---YDWWQRKPAQRLRRGLPSI------------ARAGKLRRLARA--------------------------------------RRP---------------------------LIARPSRHPFTARARPQRYPRRE----------------------------------------------------------------------------------------Xtro_IGF2_ENSXETG00000014020/1-218        MEQLSCKH--------------------------RSSSVDAEGQLC---------------------------------------RQAESRSTQL-PR-------------MSVMRHLLLLSITFLVYTLDSAKA----------------------------------------------------------------------------YGATETLCG-GELVDTLQFVCGDRGFYFS-RN----------------------------------------------------------------------------------------NGR-------------------------------------------------------------------SNRRANRG-------IVEECC-FRSCDLE-LLETYCA--------KP-----------------AKN--ERDVSTAPS-----------------------------------------------------------------------------TAIP---PLN------------------------------------------KQDLYHK-HHHTKSSK---YDIWQRKSIHRLRRGVPAI------------VRARQYRLLMQQAEESEQA-----------------------------LSHRP---------------------------LTTLPITRPLHLQQTSEPSLN------------------------------------------------------------------------------------------Hsap_igf-2/1-180                          MG-------------------------------------------------------------------------------------------------------------IPMGKSMLVL-LTFLAFASCCIAA----------------------------------------------------------------------------YRPSETLCG-GELVDTLQFVCGDRGFYFS-RP----------------------------------------------------------------------------------------ASR-------------------------------------------------------------------VSRR-SRG-------IVEECC-FRSCDLA-LLETYCA--------TP-----------------AKS--ERDVSTPP------------------------------------------------------------------------------TVLP--------------------------------------------------DNFPR-YPVGKFFQ---YDTW-KQSTQRLRRGLPAL------------LRARRGHVLAKELEAFREA-----------------------------KRHRP---------------------------LIALPTQDPAHGGAPPEMASNRK----------------------------------------------------------------------------------------Ggal_igf-2/1-187                          ---------------------------------------------------------------------------------------------------------------MCAARQILLLLLAFLAYALDSAAA----------------------------------------------------------------------------YGTAETLCG-GELVDTLQFVCGDRGFYFS-RP----------------------------------------------------------------------------------------VGR-------------------------------------------------------------------NNRRINRG-------IVEECC-FRSCDLA-LLETYCA--------KS-----------------VKS--ERDLSATSL-----------------------------------------------------------------------------AGLP---ALN-------------------------------------------KESFQK-PSHAKYSK---YNVWQKKSSQRLQREVPGI------------LRARRYRWQAEGLQAAEEA----------------------------RAMHRP---------------------------LISLPSQRPPAPRASPEATGPQE----------------------------------------------------------------------------------------Mmus_igf2/1-191                           MGGS-------------------------------------------------------------------------------------VAGFQVPMG-------------IPVGKSMLVL-LISLAFALCCIAA----------------------------------------------------------------------------YGPGETLCG-GELVDTLQFVCSDRGFYFS-RP----------------------------------------------------------------------------------------SSR-------------------------------------------------------------------ANRR-SRG-------IVEECC-FRSCDLA-LLETYCA--------TP-----------------AKS--ERDVSTSQ------------------------------------------------------------------------------AVLP--------------------------------------------------DDFPR-YPVGKFFQ---YDTW-RQSAGRLRRGLPAL------------LRARRGRMLAKELKEFREA-----------------------------KRHRP---------------------------LIVLPPKDPAHGGASSEMSSNHQ----------------------------------------------------------------------------------------Locu_igf2b_ENSLOCG00000001806/1-217       MEDQ------------------------------QKYSYQAFCHTC---------------------------------------LGTENRRMKM-RK-------------MSTSRQMLVFTIALTFYIMDVAKP----------------------------------------------------------------------------FSYAETLCG-GELVDALQFVCEDRGFYFS-RP----------------------------------------------------------------------------------------TSR-------------------------------------------------------------S-----NSRRAQKG-------IVEECC-FRSCDLN-LLEMYCA--------KP-----------------AKS--ERDVSSTSL-----------------------------------------------------------------------------QGIPALPALK--------------------------------------------EAPRK-PLTVKYSK---YDVWQRKAAQRLRRGIPAI------------LRARKFRHQAEKVKAQEQL-----------------------------LFHKP---------------------------LITLPSKLPPAVQSSTEKSVSHK----------------------------------------------------------------------------------------Drer_IGF3_ENSDARG00000058058/1-194        MPSD-------------------------------------AMPTC-------------------------------------HAKRLQIMPGFMLKV-------------PSWRSVCVLYSLCCVLILPDN-------------------------------------------------------------------------------TEGARARCG-RELVDDLEFVCGDRGFYIG-KP----------------------------------------------------------------------------------------GAA------------------------------------------------------------RSG----GPRSRGKG-------IVDQCC-VRGCDLQ-HLELYCA--------KS-----------------KKV--RRDVPASVL------------------------------------------------------------------------------QTPEDQFWL--------------------------------------------------VFQRRYQKLADLQRDEESASQRIRER-----TL-----YQQNLLNTKTSTQTSSTHLSSTT-------------------------------ETP-------------------------------------------------------------------------------------------------------------------------TFISHIR----------Xtro_IGF3_ENSXETG00000002876/1-123        M--------------------------------------------CLQ--------------------------------DSKKLKKAKLTRKKE-TP-------------FPISRVVLCLSLVFTLYVEATNARC-------------------------------------------------------------------------LRPRSKELLCG-AELVDILQFICGPTGFYVS-KG----------------------------------------------------------------------------------------------------------------------PP--------------------------------------FRNRNRPG-------IVEECC-FCGCSVA-ILESYCA--------AP-----------------VTNITGREEQKS------------------------------------------------------------------------------------------------------------------------------------------------------------------------------------------------------------------------------------------------------------------------------------------------------------------------------------------------------------------------------------Locu_novel_ENSLOCG00000011459/1-206       MKSSG----------------------------------HVSKPVCMASSSTGQRALK-----------------------------------------------------VSWIRRLWVTYAVLCFLALPQR------------------------------------------------------------------------------GTATKSRCG-RELLADLEFVCGDRGYYRGSKP----------------------------------------------------------------------------------------LDRQ--------------------VL---------------------SLPVIAGNAQGY-----------GRRLRGKG-------IVEQCC-LRGCDLQ-HLESYCA--------KP-----------------QRS--RRLAPSVEPHS---------------------------------QEGKLREIFRKHVLAPSRNLE--------SIRLNKV-VALGNRNLPEPGAATSV------------------------------------------------PRISRHSFL--LKEISRKDVER-----------------------------------------------------------------------QP------------------------------------------------------------------------------------------------------------------------------------------Cint_ins-l1_DQ538510/1-275                MYDA-------------------------------------KGNSCLRKTNNFQRDVLF----------------------NEELLEKNEPSYIVPSL-------------RHCCRRRITVSVITYAFVLLYVSS----------------------------------------------------------------------------HCEAEYLCG-SRLVDALRFICGSRGINGP-GR------------------------------------------G----------------SYMH-------------------------ARR------------------------------------------------------------GSH----LRKRPEAD-------ITVLCC-ERGCTMK-QMERFCG--------EPGRR--------------HQS--RRRGYRNRHRN-------------------------------------------------------------------------PYSRYPRPPHPPTFNDPP------------------ATTSTELITTTAM-------------STTMRQNA---TTAVRSTLLDDLRHRYRSI------------LERERARSQSGNAADRKSI----------------------------------------------------------FHDIVKVASELFRESNTDPGGSGYEYEYHDR------------ILTASFHVNAESEDHH-------------------------------------------------------Cint_ins-l3_DQ665317/1-185                MSET---------------------------------------------------------------------------------SRVVSATR-PSSG-------------GTYRRLCLVTCIVVTCMAIFPGSS------------------------------------------------------------------------IAHGRKRFVLSCG-SRLVNSMRYMCLQEVWTPK----------LCSVFRT-------------------------KFCGTEINFHP-------ICRF---TC----------------ITESRISDRQEIAITTSASPQRVVSPYPHNNTNDVTTPSPNA-----------SHVFRSLRSSKKLSK--------HMRRTKRF-------IAKRCC-SEICSVI-VLRQFCLG--------------------------DET---------------------------------------------------------------------------------------------------------------------------------------------------------------------------------------------------------------------------------------------------------------------------------------------------------------------------------------------------------------------------------------------Cint_ins-l2_DQ643991/1-130                M------------------------------------------------------------------------------------------------------------------KIVIVVLIVLFIITPSP-------------------------------------------------------------------------------VESWSGACG-ARLINRLRFICGERGVFNP-KTLLLRMK----------------------------------YPG----------------RYRH-------------------------HRRTRSATTIK-------------EIIQETSPSV-------------SVLRGLVTVGNHLHNNRRR----LARRVRRD-------LVTECC-RGSCNRKWILRTYCG---------------------------------------------------------------------------------------------------------------------------------------------------------------------------------------------------------------------------------------------------------------------------------------------------------------------------------------------------------------------------------------------------------------------------Bflo_novel/1-148                          ------------------------------------------------------------------------------------------HTSVLQTT-------------MSPSGVLLMTCLSLIGCAA---------------------------------------------------------------------------------PASSAYLCG-STLFDVLSWVCEGRGEPGV-NK----------------------------------------------------------------------------------------------------------------------N------------------DVPNVDNEARLHP-RSP----QFSRRVRE-------LIDDCC-FNVCTFD-TLESYCTPW----AETP-----------------EPN--PNDAEDAA-------------------------------------------------------------------------------EIPEGE-----------------------------------------------------TTISKFGS-------------------------------DHVTRERDSRNGVLS-----------------------------------------------------------------------------------------------------------------------------------------------------------------------------------Blan_BL13734_IGF1/1-154                   MNDR------------------------------------------------------------------------------------SLYTSVLQTT-------------MSPSGVLLMTCLLMIGAAT---------------------------------------------------------------------------------PARSAYLCG-STLFDVLSWVCEERGYTAP-EK----------------------------------------------------------------------------------------------------------------------NP-----------------DVDNVENEARIHP-RSP----QFSRRVQK-------LIDDCC-FNVCDFD-TLESYCNPW----AETP-----------------EPN--PNDAEDAA-------------------------------------------------------------------------------EIPEE------------------------------------------------------TTISKFGS-------------------------------DHVTRERDSHNGVLS-----------------------------------------------------------------------------------------------------------------------------------------------------------------------------------Bbel_LOC109477594/1-110                   ---------------------------------------------------------------------------------------------------------------MSPSRVLLMTCLSLIGLT----------------------------------------------------------------------------------LANGEYLCG-STLKDVMSFVCGRRGH--------------------------------------------------------------------------------------------------------------------------------------------------------------------RARLNDHK-------LIEDCC-LNVCDYE-YLESYCNPSPRKHPRQP-----------------RKH--PRQFRTTPK---------------------------------------------------------------------------TPRKFPTEE-----------------------------------------------------TTMPKFVV---------------------------------------------------------------------------------------------------------------------------------------------------------------------------------------------------------------------------------Blan_BL01444_IGF2/1-307                   ---------------------------------------------------------------------------------------------------------------MNLSSVYVLASLAVVCLLVK--------------------------------------------------------------------------------ETQAEYLCG-STLADVLSFVCGNRGYNSQ-PRRSL-------------------------------------------------------------------------------------GKR------------------------AID-------------------FISEQEAKDYM---GAM----PHIRRRRG-------LVEECC-YNVCDYS-QLESYCNPY----TTAPATA--TPVRTAPPEE-QEEE--QQDEDAAPLDGM-VGDQAPLGSIENIENLVYHYDRDDITVDAAKKEPK-K---LKEILGSFEDKKANPVFP--FIRQS---KNVKPNKFPDSSAHQYP------------------------------------TDLVEEGPTNEI--------P--ESPSQKPTLERLGYKDNQTDKKEPAENNNNNNRARDNRTKSSTVEPHTVP---------------------DYISK--QYTHKP---------------------------LITLPRGTPRRIESPGQLSLN------------------------------------------------------------------------------------------Bflo_BRAFLDRAFT_121099/1-417              MPILTGQ----------------------------------VWITCGQRNGNYR--------------------IHPISGLPFQDKQAGISSDVQATG-------------MNLSSVYVLASLAVVCLLVK--------------------------------------------------------------------------------ETQAEYLCG-STLADVLSFVCGNRGYNSQ-PRRSTPLGVAYSVATRQLCYSNFPVLSEQYVFLFGPNLKDGTWRGV---FSPGCERRVLINRWKPRKGGAHEIPVQGRPEAGLQTLMRRGASA------------------------QVD-------------------FISEQQAKDYM---GAM----PHIRRRRG-------LVEECC-YNVCDYS-QLESYCNPY----TTAPATA--TPVRTTEPQ--PEEA--EDD----PLDGM-VGDQAPLGSIENIENLVYHYDSDDITIDAAKKEPK-K---LKEILGSFEDKKANPVFP--FIRQS---KNIKPNKFPDNFAHQYP------------------------------------TDLVEEGPTNEI--------P--ESPSQKPTLERLGYKHNQTDKKQPTENNNNNNRARDNRTKSSTVEPHTVP---------------------EYISK--QYTHKP---------------------------LITLPRVNVR-----------------------------------------------------------------------------------------------------Bbel_LOC109477811/1-355                   MFCQRGRL--------AA----------------FAPPRSLITPSCSTRRG----------------------------AAPTSAARGAARRYFQATG-------------MNLSSVYVLASLAVVCLLVK--------------------------------------------------------------------------------ETRAEYLCG-STLADVLSFVCGNRGYNSQ-PRRSL-------------------------------------------------------------------------------------GKR------------------------AID-------------------FISEQQAKDYM---GSM----PPVRRRRG-------LVEECC-LNVCDYS-HLESYCNPH----PTAPATA--SPVRTTESQ--PEEQ--ERDEELAPLDGM-VGDQAPLGSIENIENLVYHYDRDDITVDAAKKEPKMK---LKEILGSFEDKKANPVFP--FIRQSTKNQNVKPNKFPDSSAHQYP------------------------------------TDLVEEGPTNEI--------P--ETPSQKPTLERLGYKDNQTDKKEPAE-NNNNNRARDNRTKSSTVEPHTVP---------------------DYISK--QYTHKP---------------------------LITLPRGTPRRIDAPGQLSLN------------------------------------------------------------------------------------------Spur_SPU_030139_igf2/1-423                MDPF----------------------------------------------------------------------------------------------------------------RVLLYMVTFLLYVVG--------------------------------------------------------------------------------PISSFRLCG-RELADALAVVCKGRGYYID-DS----------------------------------------------------------------------------------------------------------------EIAQKDSP-----------------IVPHHVASSFLGSSSASAHSRQRRRVRTG------QIVNECC-DKECSNN-IMESYCNR------RTPEVPPESAISENPSEEITEDSTLRTDGESTEI---------------------------------------------RTDTNPATNLE----------------VPSPDANTPDATATSDVEQPRSDNTTAVEKPRKKDNGKGKNSSLESSTKKNRTSKGMSKEDRRRIASDERRASRERKKELSRERRKRLK------------------LQQRKDKKKKKRLESAERNRGTDHMGLSEDSTLLAREPLGIDVRK--RFHHTPRSSREQASTATHALDDDPATSRQERRRTQSRPSSRERKTHRTTTATAREEEMQRERRNVMQRLTVIEIDESSFTASDSDNDDVGEVEGCMELGSSVKQESWQEYRTDARVRVRLQLYGYFGAFFS-IRTLT-------Skow_LOC100313638_ilpgf/1-345             M---------------------------------------VRCMEC------------------------------------------------------------------------LCVCLAVAVFTTD--------------------------------------------------------------------------------VMAWDKLCG-RTLVDVLALICNGRGYNSG-SP----------------------------------------------------------------------------------------KKK-----------------------VKRESP-----------------FRSGMEANDFFGNISSKEKRRQRRRSGSG------KIVDECC-HQACDYT-TLESYCAP------LPEGVVADDSLKRF-----LSQSF-GNDFKDTANED------------------------------------KLE---IVTVVRPSHD-EMDGTET--RIEDN---EHVTPPTKPDVNTETSSL--------ILDDINVNKQIISSDTSVEVKSKAGN-TKRKREKKDRDNSSSKKRSHPKPKKSRKKQRLQRIRKKARGTR--------KTKLRHVKVKSKSTPIQQIETTTTMKPFFDVDTDIIFYR-----FVNKTWRVIEEPEI-------------------------MVGGKSESSRESDIDSSISHNSRESH----------------EPSREIDTTDCDSV-------------------------------------------------------Spur_SPU_007203_igf1/1-410                MTQ------------------------------------------CYLRRPTNRLLLEFWLQSAVTLSQRFRLKYLRFVYDNTKELRDNVKRKLEPSNRVIVKDFRAYGKMVCFRYPVAVMSVVLLALLRHV-------------------------------------------------------------------------------TASFPLLCG-QELVKAVAAVCNDRGYYGQ-PS----------------------------------------------------------------------------------------KRS-------------------------------------------AGIFELETRAKTFLKSGMSRG---ETRRSKRG-ARTGL-IVTECC-LNRCSVS-HLESYCNP------LPPDAVHDAEVHIR-----LEKSA-EEDADEGRPQDGPSQLDTATGTVP----------------------------------------ETDTSETRGRVRIDAVEKVISERLIPTSTTGS--------------------------------------SPSPSRKKPRKDKSERRNSS---REAKQARREERRRNRERGSG-----------GRSRSGRRKDKDNDRASRAK--------RHGLNLWRN---MFSDK--FFSDIP--------------------------GLENQPNLHPVNGRAPSSTTIDTFQMKSSI-----------PIQDSPEGNGKENFEQ----------SSINQEA------DKKMR--------FSALMTKLRTMVLKFPKDRCgig_CGI_10012542_INS/1-176               MSCRNW---------------------------------------C----------------------------------NSWEIMPVNRKHQ----------------------------IFILLCLHFTSVQSDFERV---------------------------------------------------------------CNSQTDLRGPDPQGICG-RLIPEMLHLVCGGQYYVPS-KRDVSSLS--------------------------------------------------------HHK-----------------------QDR------------------------NVDFPRYSPLE---------GLILGKREASMYLTSQHSR----TKRNAYQG-------IVCECC-YHGCNWF-ELQQYCGFR-------------------------KKRNTEPDSISASSQNS----------------------------------GKLI---D------------------------------------------------------------------------------------------------------------------------------------------------------------------------------SVLNK--------------------------------------------------------------------------------------------------------------------------------------------------Obim_Ocbimv22037295/1-185                 MKRIRWQ---------------------------------------------------------------------------------------EASI-------------KAVFYISVLFNLLIDCRVKANIQEDVIEI-IQGRN-------------------------FQHW--------------------------------RNVWNQECFRRCR-AELGPHISIACQNDIYKIINKR---------------------------------------------------------------------------------------PPRRLRRSTNIS------------VDTNDVRRPLSQHQLRRHRRSLRFPFLLQDSEAFSYLKREHTM----VKRGQAGG-------IMEECCYMKGCTWE-EYAEFCHSH-------------------------SRY--QTSGENGNC---------------------------------------------------------------------------------------------------------------------------------------------------------------------------------------------------------------------------------------PTF----------------------------------------------------------------------------------------------------------------------------------------Obim_Ocbimv22011416/1-129                 MEKI-----------------------------------------------------------------------------------------------------------IKMFTWNIVFLLFVTTLSRYTVKAGLEHR---------------------------------------------------------------CNDETISRTSVSSSYCA-AKMPNFLRLVCNREEYGKM-DEILF-------------------------------------Y-----------------------------------------------TTR---------------CP----------KPKRSKLN---------GVVISKKKAKSHLT---------RKSNFWCG-------IVCECC-HHTCSLD-ELLAYC----------------------------------------------------------------------------------------------------------------------------------------------------------------------------------------------------------------------------------------------------------------------------------------------------------------------------------------------------------------------------------------------------------------------------Hvul_ilp2/1-189                           MNFN-----------------------------------------------------------------------------------------------------------NFTFMLFVWMCLTFDLYNLLDSKSADDYDNAKIQN---KNEDLKNEESFTSRINEILKAYYNRW-------KTENALEYKTNFDVVEYEDDSLNKKENKIIQRELRVCTQAFLQSLLKHLC--------------------------------------------------------------------------------------------------------------------VFPNPVAKLIKKDTPITKK-----------DFTLSNDVASNFLNKRDNT----WFRHQYPDVYPTDINVHDECCYNKGCVVD-EIMEYCN---------------------------------------------------------------------------------------------------------------------------------------------------------------------------------------------------------------------------------------------------------------------------------------------------------------------------------------------------------------------------------------------------------------------------Hvul_ilp1_LOC100205786/1-119              M--------------------------------------------------------------------------------------------L-----------------IEMQSSIIFLIIIILPGVLGGEEK----------------------------------------------------------------------------PAHAQALCG-NQFLLRWKVLCQIESQKKA-NNLHIELKS-----------------------------------------------------------------------------MMK-EQK--------------------------------------------HFLLKANTAKKFLNP-LKR----KRRSIFSG--KLD-NADEECC-KEKCVTLEILEYPC----------------------------------------------------------------------------------------------------------------------------------------------------------------------------------------------------------------------------------------------------------------------------------------------------------------------------------------------------------------------------------------------------------------------------Hvul_ilp3_LOC100202208/1-187              MYDK---------------------------------------------------------------------------------------------------------------MTVVCLCLLLHISERIAYGETIESSVLDVDSITSSKKEQEDSSVLTLRLNKILRRYYEEWSGTFTNDKLKKETDLKNKISKNLLVNDFIPKKK---EANEWRVCSHRSFLFILKYVCNINNKPLSWSK----------------------------------------------------------------------------------------KER---------------------------------QFT------KKELVIGGNQATSFLNT--------NYHWLNAG-FSTDVDFIDECCNLKGCNSN-EISEYCN---------------------------------------------------------------------------------------------------------------------------------------------------------------------------------------------------------------------------------------------------------------------------------------------------------------------------------------------------------------------------------------------------------------------------2. InsR/IGF1RHsap_INSR/1-1382                         M--------------------------------ATGGRRGAAAAPL-------------------------------------------------------------------------------------------------------------------------------------------------------------------------------------LVAVAALLLGAAG---HLYPG---------------------------------------------------------------------------------------------------EVC-PGMDIRNNLTRL-----------------HELENCSVIEGHLQILLMFKT----RPEDFRDL----------SFPKLIMITDYLLLFRVYGLESLK--DLFPNLTVIRGSRLFF-NYALVIFEMVHLKELGLYNLMNITRGSVRIEKNNELCYLATIDWSRILDS-----VEDNYIVLNKDDN--EEC-GDICPGTAKGK--------TNCPAT-------VINGQ-----FVERCW--------THSHCQK--VC------PTICKSHG--C-TA-----EGLC-CHSECLGNCSQ-PDDPTKCVACRNFY----LDGRCVETCPPPYYH--FQDWRCVNFSFCQDLHHKCKNSRR-QGCHQYVI--------HNNKCIPECPSGYTMN--SSN--LLCTPCL-G-PCPKVC--HLL----------EGEKTIDSVTSAQELRGCTVINGS--LIINIR--GGNNLAAELEANLGLIEEISGYLKIRRSYALVSLSFFRKLRLIRGET-----LEIGNYSFYALDNQNLRQLWDWS----KHNLTITQGKLFFHYNPKLCLSEIHKMEEVSGTKGRQ-ERNDIALKTNGDQASCENE--LLKFSYIRTSFDK-----------------ILLRWEPYW-PP-------------DFRDLLGFMLFYKEAPY--QNVTEFDGQDAC-GSNSWTVVDIDPPLRSN-DPKSQ--NHPGWLMRGLKPWTQYAIFVKTLVTF-SDERRTYGAKSDIIYVQTDATNPSVPLDPISVSNSSSQIILKWKPPSDPNGNITHYLVFWERQAED-SELFE-LDYCLKGLKLPSRTWSPP----------FESEDSQ------------KHNQSEYED---SAGECCSC---PKTDSQI-LKELEE------SSFRKTFEDYLHNVVFVPRKTSSGTG-----AEDP-----------------------------------RPSRKRRSLGDVGN--------------------------------VTVAVP--TVAAF-------PNTSSTSVPTSPEEHRPF---------EKV-----VNKESLVISGLRHFTGYRIELQACNQ----------DTPEERCS--------VAAYVSARTMPEAKADDIVGPVTHEI-----FENNVVHLMWQEPKEPNGLIVLYEVSYRRYG----------------------D-EELHLCVSRKH-F-ALERGCRLRGLSPGNYSVRIRATSLAGNGSWTEPTYFYVTD---YLDVPSNIAK----------------------------------------------------------------------------------------------------------------------------------------------------------------------------------------------------------------------------------------------------------------------------IIIGPL-IF-VFLFSVVI----GSIYLFLRKRQPDG--PLGPLYASSNPEYLSASDVFPCSVYVPDEWEVSREKITLLRELGQGSFGMVYEGNARDIIK--GEAE-TRVAVKTVNESASLRERIEFLNEASVMKGF---TCHHVVRLLGVVSKGQPTLVVMELMAHGDLKSY---LRSLRPEAE------------------NNP--------------GRPPPTLQEMIQMAAEIADGMAYLNAKKFVHRDLAARNCMVAHDFTVKIGDFGMTRDIYETDYYRKGGKGLLPVRWMAPESLKDGVFTTSSDMWSFGVVLWEITSLAEQPYQGLSNEQVLKFV-MDGGYLDQPDNCPERVTDLMRMCWQFNPKMRPTFLEIVNLLKD--DL-HPSFPEVSFFHSEE-NKAPESEELE----MEFEDM-ENV-----------------------------------PLDRSSHC------------------------------------------QREEAG-GRDGGS-----------------------------SLGFKRSYEEHIPYT----------------------------------------------------------------------------------------------------------------------------------------------------------------------------HM-NGGKKN----------------------------------------------------------------------------------------------------------------------------------------------------------------------------------------GRILTL------------------------------PRSNPS---------------------------------------Hsap_IGF1R/1-1367                        M------------------------------KSGSGGGSPTSLWGL-------------------------------------------------------------------------------------------------------------------------------------------------------------------------------------LFLSAALSLWPTS-------G---------------------------------------------------------------------------------------------------EICGPGIDIRNDYQQL-----------------KRLENCTVIEGYLHILLIS------KAEDYRSY----------RFPKLTVITEYLLLFRVAGLESLG--DLFPNLTVIRGWKLFY-NYALVIFEMTNLKDIGLYNLRNITRGAIRIEKNADLCYLSTVDWSLILDA-----VSNNYIVGNKPPK---EC-GDLCPGTMEEK--------PMCEKT-------TINNE-----YNYRCW--------TTNRCQK--MC------PSTCGKRA--C-TE-----NNEC-CHPECLGSCSA-PDNDTACVACRHYY----YAGVCVPACPPNTYR--FEGWRCVDRDFCANILSAES-----SDSEGFVI--------HDGECMQECPSGFIRN-GSQS--MYCIPCE-G-PCPKVC-EEE-----------KKTKTIDSVTSAQMLQGCTIFKGN--LLINIR--RGNNIASELENFMGLIEVVTGYVKIRHSHALVSLSFLKNLRLILGEE-----QLEGNYSFYVLDNQNLQQLWDWD----HRNLTIKAGKMYFAFNPKLCVSEIYRMEEVTGTKGRQ-SKGDINTRNNGERASCESD--VLHFTSTTTSKNR-----------------IIITWHRYR-PP-------------DYRDLISFTVYYKEAPF--KNVTEYDGQDAC-GSNSWNMVDVDLPPNKD--------VEPGILLHGLKPWTQYAVYVKAVTLTMVENDHIRGAKSEILYIRTNASVPSIPLDVLSASNSSSQLIVKWNPPSLPNGNLSYYIVRWQRQPQD-GYLYR-HNYCSKD-KIPIRKYADGT---------IDIEEVT------------ENPKTEVCGG--EKGPCCAC---PKTEAEK-QAEKEE------AEYRKVFENFLHNSIFVPR----------------------------------------------------PERKRRDVMQVAN--------------------------------TTMSSRSRNTTAA-------DTY-NITDPEELETEYPFFE-------SRV-----DNKERTVISNLRPFTLYRIDIHSCNH----------EAEKLGCS--------ASNFVFARTMPAEGADDIPGPVTWEP-----RPENSIFLKWPEPENPNGLILMYEIKYGSQV------------------------EDQRECVSRQE-Y-RKYGGAKLNRLNPGNYTARIQATSLSGNGSWTDPVFFYVQA---KTG-YENFIH---------------------------------------------------------------------------------------------------------------------------------------------------------------------------------------------------------------------------------------------------------------------------LIIALPV-AV-LLIVGGLV----IMLYVFHRKRNNSR-LGNGVLYASVNPEYFSAAD-----VYVPDEWEVAREKITMSRELGQGSFGMVYEGVAKGVVK--DEPE-TRVAIKTVNEAASMRERIEFLNEASVMKEF---NCHHVVRLLGVVSQGQPTLVIMELMTRGDLKSY---LRSLRPEME------------------NNP--------------VLAPPSLSKMIQMAGEIADGMAYLNANKFVHRDLAARNCMVAEDFTVKIGDFGMTRDIYETDYYRKGGKGLLPVRWMSPESLKDGVFTTYSDVWSFGVVLWEIATLAEQPYQGLSNEQVLRFV-MEGGLLDKPDNCPDMLFELMRMCWQYNPKMRPSFLEIISSIKE--EM-EPGFREVSFYYSEE-NKLPEPEELD----LEPENM-ESV-----------------------------------PLDPSASS------------------------SSLPLP------------DRHSGH-KAENGP--------------------------GPGVLVLRASFDERQPYA----------------------------------------------------------------------------------------------------------------------------------------------------------------------------HM-NGGRKN----------------------------------------------------------------------------------------------------------------------------------------------------------------------------------------ERALPL------------------------------PQSSTC---------------------------------------Mmus_INSR/1-1372                         M--------------------------------GFGRGCETTAVPL-------------------------------------------------------------------------------------------------------------------------------------------------------------------------------------LVAVAALLVGTAG---HLYPG---------------------------------------------------------------------------------------------------EVC-PGMDIRNNLTRL-----------------HELENCSVIEGHLQILLMFKT----RPEDFRDL----------SFPKLIMITDYLLLFRVYGLESLK--DLFPNLTVIRGSRLFF-NYALVIFEMVHLKELGLYNLMNITRGSVRIEKNNELCYLATIDWSRILDS-----VEDNYIVLNKDDN--EEC-GDVCPGTAKGK--------TNCPAT-------VINGQ-----FVERCW--------THSHCQK--VC------PTICKSHG--C-TA-----EGLC-CHKECLGNCSE-PDDPTKCVACRNFY----LDGQCVETCPPPYYH--FQDWRCVNFSFCQDLHFKCRNSRK-PGCHQYVI--------HNNKCIPECPSGYTMN--SSN--LMCTPCL-G-PCPKVC--QIL----------EGEKTIDSVTSAQELRGCTVINGS--LIINIR--GGNNLAAELEANLGLIEEISGFLKIRRSYALVSLSFFRKLHLIRGET-----LEIGNYSFYALDNQNLRQLWDWS----KHNLTITQGKLFFHYNPKLCLSEIHKMEEVSGTKGRQ-ERNDIALKTNGDQASCENE--LLKFSFIRTSFDK-----------------ILLRWEPYW-PP-------------DFRDLLGFMLFYKEAPY--QNVTEFDGQDAC-GSNSWTVVDIDPPQRSN-DPKSQTPSHPGWLMRGLKPWTQYAIFVKTLVTF-SDERRTYGAKSDIIYVQTDATNPSVPLDPISVSNSSSQIILKWKPPSDPNGNITHYLVYWERQAED-SELFE-LDYCLKGLKLPSRTWSPP----------FESDDSQ------------KHNQSEYDD---SASECCSC---PKTDSQI-LKELEE------SSFRKTFEDYLHNVVFV-----------------P-----------------------------------RPSRKRRSLEEVGN--------------------------------VTATTL--TLPDF-------PNVSSTIVPTSQEEHRPF---------EKV-----VNKESLVISGLRHFTGYRIELQACNQ----------DSPDERCS--------VAAYVSARTMPEAKADDIVGPVTHEI-----FENNVVHLMWQEPKEPNGLIVLYEVSYRRYG----------------------D-EELHLCVSRKH-F-ALERGCRLRGLSPGNYSVRVRATSLAGNGSWTEPTYFYVTD---YLDVPSNIAK----------------------------------------------------------------------------------------------------------------------------------------------------------------------------------------------------------------------------------------------------------------------------IIIGPL-IF-VFLFSVVI----GSIYLFLRKRQPDG--PMGPLYASSNPEYLSASDVFPSSVYVPDEWEVPREKITLLRELGQGSFGMVYEGNAKDIIK--GEAE-TRVAVKTVNESASLRERIEFLNEASVMKGF---TCHHVVRLLGVVSKGQPTLVVMELMAHGDLKSH---LRSLRPDAE------------------NNP--------------GRPPPTLQEMIQMTAEIADGMAYLNAKKFVHRDLAARNCMVAHDFTVKIGDFGMTRDIYETDYYRKGGKGLLPVRWMSPESLKDGVFTASSDMWSFGVVLWEITSLAEQPYQGLSNEQVLKFV-MDGGYLDPPDNCPERLTDLMRMCWQFNPKMRPTFLEIVNLLKD--DL-HPSFPEVSFFYSEE-NKAPESEELE----MEFEDM-ENV-----------------------------------PLDRSSHC------------------------------------------QREEAG-GREGGS-----------------------------SLSIKRTYDEHIPYT----------------------------------------------------------------------------------------------------------------------------------------------------------------------------HM-NGGKKN----------------------------------------------------------------------------------------------------------------------------------------------------------------------------------------GRVLTL------------------------------PRSNPS---------------------------------------Mmus_IGF1R/1-1369                        M------------------------------KSGSGGGSPTSLWGL-------------------------------------------------------------------------------------------------------------------------------------------------------------------------------------VFLSAALSLWPTS-------G---------------------------------------------------------------------------------------------------EICGPGIDIRNDYQQL-----------------KRLENCTVIEGFLHILLIS------KAEDYRSY----------RFPKLTVITEYLLLFRVAGLESLG--DLFPNLTVIRGWKLFY-NYALVIFEMTNLKDIGLYNLRNITRGAIRIEKNADLCYLSTIDWSLILDA-----VSNNYIVGNKPPK---EC-GDLCPGTLEEK--------PMCEKT-------TINNE-----YNYRCW--------TTNRCQK--MC------PSVCGKRA--C-TE-----NNEC-CHPECLGSCHT-PDDNTTCVACRHYY----YKGVCVPACPPGTYR--FEGWRCVDRDFCANIPNAES-----SDSDGFVI--------HDDECMQECPSGFIRN-STQS--MYCIPCE-G-PCPKVCGDEE-----------KKTKTIDSVTSAQMLQGCTILKGN--LLINIR--RGNNIASELENFMGLIEVVTGYVKIRHSHALVSLSFLKNLRLILGEE-----QLEGNYSFYVLDNQNLQQLWDWN----HRNLTVRSGKMYFAFNPKLCVSEIYRMEEVTGTKGRQ-SKGDINTRNNGERASCESD--VLRFTSTTTWKNR-----------------IIITWHRYR-PP-------------DYRDLISFTVYYKEAPF--KNVTEYDGQDAC-GSNSWNMVDVDLPPNKE--------GEPGILLHGLKPWTQYAVYVKAVTLTMVENDHIRGAKSEILYIRTNASVPSIPLDVLSASNSSSQLIVKWNPPTLPNGNLSYYIVRWQRQPQD-GYLYR-HNYCSKD-KIPIRKYADGT---------IDVEEVT------------ENPKTEVCGG--DKGPCCAC---PKTEAEK-QAEKEE------AEYRKVFENFLHNSIFVPR----------------------------------------------------PERRRRDVMQVAN--------------------------------TTMSSRSRNTTVA-------DTY-NITDPEEFETEYPFFE-------SRV-----DNKERTVISNLRPFTLYRIDIHSCNH----------EAEKLGCS--------ASNFVFARTMPAEGADDIPGPVTWEP-----RPENSIFLKWPEPENPNGLILMYEIKYGSQV------------------------EDQRECVSRQE-Y-RKYGGAKLNRLNPGNYTARIQATSLSGNGSWTDPVFFYVPA---KTT-YENFMH---------------------------------------------------------------------------------------------------------------------------------------------------------------------------------------------------------------------------------------------------------------------------LIIALPV-AI-LLIVGGLV----IMLYVFHRKRNNSR-LGNGVLYASVNPEYFSAAD-----VYVPDEWEVAREKITMNRELGQGSFGMVYEGVAKGVVK--DEPE-TRVAIKTVNEAASMRERIEFLNEASVMKEF---NCHHVVRLLGVVSQGQPTLVIMELMTRGDLKSY---LRSLRPEVE-----------------QNNL--------------VLIPPSLSKMIQMAGEIADGMAYLNANKFVHRDLAARNCMVAEDFTVKIGDFGMTRDIYETDYYRKGGKGLLPVRWMSPESLKDGVFTTHSDVWSFGVVLWEIATLAEQPYQGLSNEQVLRFV-MEGGLLDKPDNCPDMLFELMRMCWQYNPKMRPSFLEIIGSIKD--EM-EPSFQEVSFYYSEE-NKPPEPEELE----MEPENM-ESV-----------------------------------PLDPSASS------------------------ASLPLP------------ERHSGH-KAENGP--------------------------GPGVLVLRASFDERQPYA----------------------------------------------------------------------------------------------------------------------------------------------------------------------------HM-NGGRAN----------------------------------------------------------------------------------------------------------------------------------------------------------------------------------------ERALPL------------------------------PQSSTC---------------------------------------Ggal_INSR/1-1324                         -----------------------------------------------------------------------------------------------------------------------------------------------------------------------------------------------------------------------------------------------------------------------------------------------------------------------------------------------------------------MDIRNNLTRL-----------------SLLENCTVIEGHLQILLMFKT----KPEDFREL----------SFPKLTMITDYLLLFRVYGLESLK--GLFPNLTVIRGTHLFF-NYALVIFEMVHLKEIGLYNLMNITRGAVRIEKNNELCYLSTIDWSRILDS-----VEDNYIIANKDDK--EEC-GDVCPGTVKGK--------SNCPPT-------VINGI-----FIERCW--------THDRCQR--VC------PPACKSQG--C-TS-----DGQC-CHSECLGDCTE-PNNAERCVACRNFY----LDGTCVETCPPGHYR--FEGWRCVTFSFCQELHNKCKSARE-SGCH--VI--------HNNECVHECPSGYIMN--SSN--LHCTPCA-G-PCPKVC--DY-----------GKEKTIDSVTSAQELRGCTVVNGS--LVINIR--GGNNIAAELEANLGLIEEISGYLKIRRSYALVSLSFFRKLHLIRGET-----LEAGNYSFYALDNQNLRQLWDWS----KHNLTIARGKLFFHYNPKLCLSEIHKMEEISGTKGRQ-ERNDIALKTNGDQASCENE--LLKFSSIRTSHDK-----------------ILLKWEPYW-PP-------------DFRDLLGFMLFYKEAPY--QNVTEFDGQDAC-GSNSWTVVDVDPPPRSN-EPKAQ--AQPGWLLRGLKPWTQYAVFVKTLVTF-SDERRTYGAKSEIIYVQTNATVPSVPLDPISVSNSSSQIILKWKPPSEPNGNITHYLVYWQQQAED-SELYE-LDYCLKGLKLPSRTWSPP----------FESEDPQ------------KYNQSESED---VSGECCSC---PKTDSQI-QKELEE------SAFRKTFENYLHNEVFV-----------------P-----------------------------------RPSRKRRDLGSIAN--------------------------------ATVVIP--TIPSS-------PN-SSAAASESAEEQKPF---------EKV-----KFKESLVISGLRHFTGYRIELHACNH----------DAQESRCS--------VAAYVSARTMPEAKADDIVGPVTHEL-----VEKNTVHLKWQEPKEPNGLIVLYEVNYGRLG----------------------EAEEAHFCVSRKH-F-ASEQGCKLRGLQPGNYSVRIRATSLAGNGSWTEPTYFYVAD---YLNAQPNIA-----------------------------------------------------------------------------------------------------------------------------------------------------------------------------------------------------------------------------------------------------------------------------VIIVPI-IF-AIIIAGII----GAAYVLVKKRQTEG--PTGPLYASSNPEYLSASD-----VYVPDEWEVPREKITLLRELGQGSFGMVYEGIAKDIVK--GELE-TRVAVKTVNESASLRERIEFLNEASVMKGF---SCHHVVRLLGVVSKGQPTLVVMELMAHGDLKSY---LRSLRPDAE------------------NNP--------------GRPPPTLREMIQMAAEIADGMAYLNAKKFVHRDLAARNCMVAEDFTVKIGDFGMTRDIYETDYYRKGGKGLLPVRWMAPESLKDGVFTTYSDVWSFGVVLWEISSLAEQPYQGLSNEQVLKFV-MDGGYLDQPDNCPERLHNLMQMCWQYNPKMRPTFIEVIEMLKE--DL-HPSFQEVSFFYSEE-NKPLETEEYE----MDFENM-ESI-----------------------------------PLDPSSYT------------------------------------------QRDKAL-GRDNGP-----------------------------SMALKGNYEEHIPYT----------------------------------------------------------------------------------------------------------------------------------------------------------------------------HM-NGGKKN----------------------------------------------------------------------------------------------------------------------------------------------------------------------------------------GRILSM------------------------------PRSSPS---------------------------------------Ggal_IGF1R/1-1363                        M------------------------------KSGAGGGTLAVFCGL-------------------------------------------------------------------------------------------------------------------------------------------------------------------------------------LLAFAALCLCPTN-------G---------------------------------------------------------------------------------------------------EICGPNVDIRNDIHEL-----------------KRLENCTVVEGFLQILLIS------KAEDYRNF----------RFPKLTVITDYLLLFRVAGLESLS--DLFPNLTVIRGRNLFY-NYALVIFEMTNLKEIGLHNLRNITRGAIRIEKNSDLCYLSTVDWSLILDA-----VSNNYIVGNKPPK---EC-GDLCPGTMEEK--------PLCEKT-------SINNE-----YNYRCW--------TTNHCQK--MC------PSSCGKRA--C-TD-----QNEC-CHPECLGSCTA-PDNNTACVACRNYY----YEGVCMPTCPPNTYK--FEGWRCVTKEFCSKVPATET-----SDYERFVI--------HNDECMAECPSGFIRN-GSQS--MFCSPCE-G-PCPKIC--ED-----------GKTKTIDSVTSAQMLQGCTILKGN--LLINIR--RGNNIASELENFMGLIETVTGYVKIRHSHALVSLSFLKNLRYILGEE-----QVDGNYSFYVLDNHNLQQLWDWN----HHNLTIKEGKMYFAFNPKLCVSEIYRMEEVSGTKGRQ-SKGDINPRNNGERASCESH--ILRFVSNTTLKNR-----------------IKLTWERYR-PP-------------DYRDLISFTVYYKEAPF--KNVTEYDGQDAC-GSNSWNMVDVDLPPNKE--------NDPGILLQGLKPWTQYAIYVKAVTLTMMENHHIHGAKSEIVYIRTNAAVPSIPLDVISASNSSSQLIVKWNPPSLPNGNLSYYIVRWQQQPQD-SYLYR-HNYCSKD-KVPIRRYADGT---------IDTEEAT------------EPTKPEGCGG--EKGPCCAC---PKTEAEK-QAEKEE------AEYRKVFENFLHNSIFVPR----------------------------------------------------PDRKRRDVFRIAN--------------------------------ATLATRNRNITGA-------DHFTNASDAEESEVEYPFFE-------TKV-----DGKERTVISHLQPFTLYRIDIHSCNH----------EADTLGCS--------ASNFVFARTMPSEGADNIPGTVAWEA-----KEENTVYLKWLEPTNPNGLILMYEIKYGQHG------------------------EEKRECVSRQE-Y-KKLGGAKLTHLNPGNYSARVQATSLAGNGSWTEPVSFYVQP---KSANYDNFLH---------------------------------------------------------------------------------------------------------------------------------------------------------------------------------------------------------------------------------------------------------------------------LIIVLPI-AF-LLIIGGLL----IMLYVFNKKRNSDR-LGNGVLYASVNPEYFSASD-----VYVPDEWEVPREKITMCRELGQGSFGMVYEGIAKGVVK--DEPE-TRVAIKTVNESASMRERIEFLNEASVMKEF---NCHHVVRLLGVVSQGQPTLVIMELMTRGDLKSY---LRSLRPDTE------------------SNP--------------GQAPPTLKKMIQMAGEIADGMAYLNANKFVHRDLAARNCMVAEDFTVKIGDFGMTRDIYETDYYRKGGKGLLPVRWMSPESLKDGVFTTHSDVWSFGVVLWEIATLAEQPYQGMTNEQVLRFV-MEGGLLEKPDNCPDMLFELMRMCWQYNPKMRPSFLEIISSIKD--EL-DPAFKEVSFFYSEE-NKPPDTEELD----LETENM-ESI-----------------------------------PLDPSSTL---------------------------QPT------------DKHSGH-KAENGP----------------------------GVVVLRASFEERQPYA----------------------------------------------------------------------------------------------------------------------------------------------------------------------------HM-NGGRKN----------------------------------------------------------------------------------------------------------------------------------------------------------------------------------------ERALPL------------------------------PQSSAC---------------------------------------Locu_INSR/1-1365                         M---------------------------------------RLDTPV-------------------------------------------------------------------------------------------------------------------------------------------------------------------------------------VAFVTFICVLSNC---GISDG---------------------------------------------------------------------------------------------------EIC-KSMDIRNNITHL-----------------HAIENCTVIEGHLKILLMFKT----KPEDFRGL----------RFPKLTMITDYLLLFRVYGLESLR--DLFPNLTVVRGTNLFF-NYALVLFEMLQLKEIGLHSLMNITRGAVRIEKNPDLCYLSTLDWSKILDS-----VEDNYIVSNKDDR---EC-GDVCPGTAKGK--------TSCPQT-------IFNGH-----FSERCW--------TQDHCQR--TC------PSSCKHRA--C-TK-----EGQC-CHDECLGSCTA-PNDPRKCVACRRYL----HQGACVDNCPAGSYI--FEGWRCVSFKFCQDLHNKCKQG---TGCDEYVI--------HNGECIPECPSGYTTM-NSTM--LNCTPCA-G-LCPKVC---------------TGVKTVDSVTAAQALRGCTILNGS--LVINIR--GGNNIAAELEANLGQLEEITGYLKIRRSYALVSLSFLRKLKLIRGEV-----LESGNFSFYALDNQNLRQLWDWS----KHNMTILQGRMFFHFNSKLCMSEIRKMEEVTGTKGRD-IKNDISMKTNGDQASCETQ--VLKFTQIRTLSDK-----------------IIIKWEPFW-PP-------------DYRDLLGFMVLYKEAPY--RNVTEFDGQDAC-GSNSWVIADVDPPPRAS-DGRDQ--VEPGHLIHPLKPWTQYAIFVKTLLSA-SDEHQVHGAKSEIIYVRTNATKPSVPLDPISSSNSSSQIMLKWKPPTDANGNITHYLVFCQQQPEA-SDLYK-FDYCQKGMKLPSRAPTHQ-----------DTEEAQ------------KWNQTDEPG---QSGRCCSC---PKTETQL-KREAEE------SEYRKTFENYLHNEVFEIRTSSAGLE-----TNED-----------------------------------RPSRRRRSLGAVAN--------------------------------STIQRFFTTPSTL-------PNASTTRGPEEEEGAKVV---------LTV-----HAKESAVISSLRHFTSYQIEIHACNH----------PTDPSRCS--------MAAYVSARTMPEDKADDIVGQVTHEV------LTNTVHIRWQEPKSPNGMIILYEVNYKRLG----------------------DSEELHHCVSRKS-Y-SVDQGCKLRVLHPGNYSVRIRATSLAGNGSWTEPTYFYVQD---PRD-PSSVMK----------------------------------------------------------------------------------------------------------------------------------------------------------------------------------------------------------------------------------------------------------------------------IAIGSV-IC-IVLILVVA----GAVFMVVRKKQTEG--PTGPLYASSNPEYLSAND-----VYVPDEWEVPREKIDVLRELGQGSFGMVYEGIAKEIVK--GEPE-TRVAVKTVNESASLRERIEFLNEASVMKSF---SCHHVVRLLGVVSKGQPTLVVMELMTHGDLKSY---LRSLRPDAE------------------NNP--------------GRPPPTLKEMIQMAAEIADGMAYLNAKKFVHRDLAARNCMVAEDFTVKIGDFGMTRDIYETDYYRKGGKGLLPVRWMAPESLKDGVFTAHSDCWSFGVVLWEISTLAEQPYQGLSNEQVLKFV-MDGGYLDRPDNCAERLHNLMQMCWQYNPKMRPTFHEIIEMLKD--DL-HPSFQEVSFFYSEE-NKPPETEEFD----MDFENM-ESI-----------------------------------PLDPSSYS------------------------------------------QREESL-GRDNGP-----------------------------SMGLRGNYEEHVPYT----------------------------------------------------------------------------------------------------------------------------------------------------------------------------HM-NGGKKN----------------------------------------------------------------------------------------------------------------------------------------------------------------------------------------GRILSL------------------------------PRSSPS---------------------------------------Locu_IGF1R/1-1398                        M------------------------------RSGTERDILTLFWGL-------------------------------------------------------------------------------------------------------------------------------------------------------------------------------------LLSISVISLWPAN-------G---------------------------------------------------------------------------------------------------EICGPSIDIRNDISEF-----------------RRLENCTVVEGYLQILLIGDKSSSHNQEVFRSL----------SFPKLTLITDYLLLFRVSGLESLS--ALFPNLSVIRGRNLFY-NYALVIFEMTSLKDIGLYNLRNITRGAIRIEKNPDLCYLDSVDWSLIMDN-----EYNNFIAGNKQSK---EC-GDVCPGIMEDK---------QCIRT-------SFNEN-----YNLRCW--------TSNHCQK--VC-------TSCDKRA--C-TD-----SGQC-CHPECLGTCTL-PDNDTACAACLHYF----HEGRCVPDCPPDTYK--FEGWRCVTKEFCSKVPTSMP-----DAFDRFVI--------HSGECVQDCPSRFMRN-ESQS--MFCSACD-G-LCDKIC--EE--------------KVINSIDAAQSLKGCTVIKGN--LHINIR--RGNNIASELESFMGLIQTVTGYVKIHHSHTLVSLSFLKSLRYINGEE-----LIEDMYSFHVLDNQNLQHLWDWS----LHNLTIQSGRLLFALNPKLCVSEIRKMWEKTGITEKF--KEDNFRNNKSGKSQGESY--ILKFKSNSTMSNK-----------------IKLTWERYR-PA-------------DYRDLISFIVYYKEAPY--QNITEFDGQDGC-GSNSWNMVDVDLP-DKE--------GDPGVLLSPLKPWTQYAIFVKAITLA-AEDRHILGAKSEVVYIRTNASEPSMPLDVRSYSNSSSKLLVKWSPPTSPNGNLTYYLVRWQQQPED-RELYR-HNYCSKELKIPIRIPATGA---------VDLDEDT------------KPTKSDAGGG--EKGPCCVC---PKTVDEL-KAEAED------ASYRKVFENFLHNSIFTPRP---------------------------------------------------PDRRRRDIFAVAN--------------------------------ASLPRSTGGNATG-------PEG-NGTELEPSQREYPFYE-------AKV-----HGREWTEISHLQSFTVYRIDIHACNH----------EV--KHCS--------AAAFVFSRTKPADKADDIPGLVAWEK-----GEDDSVLLKWPEPVNPNGLVLMYEIKFRQAA-----------------------EMEKHECVSRQH-Y-KTHGWARLSNLGHGNYSARVRATSLAGNGSWTEAVNFYVQA---KPTKYENMFY---------------------------------------------------------------------------------------------------------------------------------------------------------------------------------------------------------------------------------------------------------------------------LMIIIPV-IV-LLGLAVLV----VVLIVFNKKRNSDR-LGNGVLYASVNPEYFSAAE-----MYVPDEWEVPREKITMCRELGQGSFGMVYEGIAKGVVK--DEPE-TRVAIKTVNESASMRERIEFLNEASVMKEF---NCHHVVRLLGVVSQGQPTLVIMELMTRGDLKSY---LRSLRSPEK-------------------SA--------------SQSLPPLKKMIQMAGEIADGMAYLNANKFVHRDLAARNCMVAEDFTVKIGDFGMTRDIYETDYYRKGGKGLLPVRWMSPESLKDGVFTTNSDVWSFGVVLWEIATLAEQPYQGMSNEQVLRFV-MEGGLLDKPDNCPDMLFELMRMCWQYNPKMRPSFLEIIGSIKD--EL-EPPFREMSFFYSDE-NKPPDTEELD----LEVENM-ENV-----------------------------------PLDPSSSLQPIAPVPLGQAPPLQPASPTAPGSAAPPGPPSAPPCSPGPALDKHSGL-KVANGP-----------------------------VVLLRPSFDETQPYA----------------------------------------------------------------------------------------------------------------------------------------------------------------------------HM-NGGRKN----------------------------------------------------------------------------------------------------------------------------------------------------------------------------------------ERALPL------------------------------PQSSAC---------------------------------------Cint_LOC778651_INSR/1-1439               MRKV---ILTCLVAL-LI----------SVPASSFSDELVRSLSKL---------------------RKSPTSS--------------------------THN---------------------------------------------------------------------------------------------------NNKQRLSR---------RVIRSVSNGITHAPSQINETADQQDNNND-------------------------------------------------------------------------------FDSPW---------SVYSDGPVC-DLMDIRNNPSKL-----------------TDLENCTIVDGFVRILFLNDNEQM-EPQDLSKY----------SFPKLRVISGYLFLYRVYGLVSLR--TLFPNLVMIGGSELFYKKYSLVIFENPQLREIGLESLKYIKNGAARIQNNGQLCYLDTIDWARLGKWFGNEAIERPVLLDNKDE----GC-LSQCGSE-------------KCVDV-------TEDGL-----QQSFCW--------GLNVCQQS-AC------PDQCSHA---C-NS-----KQCV-CHEHCLGGCTL-PNNASACHACRYSY----HNQTCVKTCPPGYLK--IYGWLCITRDTCADR--------------YWKQ--------HNGECLEDCPPMYTESIKGDY--HFCKRCQ-G-ECGKVC--QLP----------TLEHTISDLASVDALDQCTVIKGQ--LQISLT--GGSEVVEKMETALSELREVSS-IVIRRSFPLVSLTFLKKLKNITGDPQYLYRQGTDNYSVYVFDNKNLKELWDLKMVNGSTNFTIGGGKAFFQNNPYLCMDKIEKIENLTPGVEDT---NDISQISNGDLITCVVE--QLQIVNIEKFQDV-----------------VEIKWISPY-VK-------------DYRTLIGYRVFYKKSKY--ENATRYTEVDGC-GRSSWSVQHVPNQKGPE-NPTEV--TYTETSLLNLDPFTKYAFYVVSSNTV-GA---KKGFQSDILYFTTPASQSSPPQRLIAKAINSSAINVTWHTPQHPNGNVTHYKVRWMRQKEKSNMLNT-IDICKGGVVHSATVSKP-----------VVVPEEP------------EIINENTNN-------QCQM---TKPLDSSTKVVESQ-----------KFEDYILKNLVVAGY-------YPPIDEEE--------------------------------FFDSNFRKKRSIKENRS--------------------------------SNITGNTTTAAT--------VTLASFTAPSEASLNYKLFK-------TIVNTSEGQNVDSTILTGLHHFSEYYIEVLACNQ----------ASATVGCS--------SATLMTRRTKKLDDADKIDDSTLKYIP-SPEKTSKPSVITWQPPEDPNGFIVKYDLKFTKQD----------------------QTVDREVCCSIMS-FRKKNSCPVPRDLLPGKYQASILPTSLAAKGVWSQPISFIVED---VNTTSLS------------------------------------------------------------------------------------------------------------------------------------------------------------------------------------------------------------------------------------------------------------------------------SMVAIIVVGI-IVILIVIL-------IVYLRKRQIHN-SKKSQTYVSVNPEYSSIG------VYEPDEYEIPEENVELMDEIGHGHFGKVYEGLAKQVVK--GQPK-TKVAVKTLHGNESISKRMEFLKEASVMKAF---NSHHVVRLLGVVSMSKRPMVIMEFMAKGDLKTY---LRSTRPDAE------------------IRK---------------GDPPSLQQKLQMCGEIADGMSYLSETKYVHRDLAARNCLVHEDLTVKIGDFGLTRDVYETDYYRIDSRGILPVRWMAPESLKDGVFDSRSDVWSFGIVLWEIATLAEQPYQGQQHDQVTRFV-IDGGYMEQPKECPSKLYDMMLMCWHYSPSMRPTFLEIVASLSP--DL-SDRFKQDSFFHETK-QAEEISNDGDGF--VASGSS-ERV--------------------------------RFLPKQVAADV------------------------------------------HEDE-----------------------------------------SKQSDDESSAYC----------------------------------------------------------------------------------------------------------------------------------------------------------------------------RL-NGLHQN------------------------------------------------------------------------------------------------------------------------------------------DPSADYDDK-------------------------------------DGSIHLTL-------------------------YSRNNSTIC---------------------------------------Blan_BL00176_INSR/1-1586                 M-------------------------------RVVDKMAGLMWAAL-------------------------------------------------------------------------------------------------------------------------------------------------------------------------------------TLVIGLGLLVP-----SNGEE---------------------------------------------------------------------------------------------------HIC-GSMDIRNRVSNL-----------------RQLDNCTVIEGYLQILLIDFA----EEQEYRGL----------SFPDLVEITDYFLLYRVRGLTNLS--ELFPNLAVIRGTNLFF-NYALVVFEMLDMQKIGLYSLQNITRGSVRIEKNPNLCYLDTIDWSFIAES----GYSNNFIVDNRDEE---EC-VNYCPGS--------------CRITH---------PV-----LQDLCW--------AEEHCQK--VC------PESCLGN---CLHGCTTRSSCTC-CNEMCIGGCRG-GTSRRHCVACKYFV----HNGECLEQCPTDTYQ--YKDRRCITADECPNSTNS-----------IWKL--------HHGKCIPECPSGYTTD-PDNP--RLCTECE-G-QCPKSC--KG--------------GLVDSLAAAQRFRGCTIIEEE--LKISIR--GGDNIIDELEDNLGLIEEVGHYVAIVRSYALVTLDFLRSLKRIWGIE------KENGYAFYVLDNRNLEKLLDWD----RTNLTMDDGKLFFHFNPKLCMNVILTMVEKVGVSEDTVSDTDISTLTNGDQAQCSYS--KLDINEISTTKNM-----------------IILRWRAFT-PP-------------DFRDLLSYTVSYRETPE--QSIDEYEGQDAC-GNTEWKDFDVSP-------------DQTAHIITGLKPWTQYALLVKTYTKA-GA-REGSGAKSDIVYARTDADKPTHPLDVVVYSNSSNTLIITWNPPNRPNGNVTHYIVKYKRQQEDVSAMEN-RDYCQGGLKPHRPTQGL-----------EDIVNNE------------EETNNSTTG----DGTCCEC---PKSEDEI-RIEEEE------AAFQQEFENFLHNNVYHKRE------------NDT-----------------------------------RAGRRRRELLRRATIS------------------------------PFYSNQTVNVTL--------LS---TVPPTPTPNPNPQLE-------TTV-----WNEHMVVLTGLRHFSEYIIEVIACNA----------DE-AVGCS--------GSAVELARTQADDSADNIPGNITV-----VETKDNKAKLYWPEPYDPNSMVVLYNIEYKKLGVD--------GN----------YEDPQQICVENFR-I-LEQQGFTISSLAAGNYSVRVQATSFAGNGSWSNYVTFYVEEKAGQDTPPQVPLSLMIGMGVGFSLLVILAVIFAFWYCAKKKFGDKQMPNGVLYASVNPEYMSSADVYVPDEWEVPREKVTLIRELGQGSFGMVYEGEAKDVVKDEPKVSVAVKTVNESASIRERIEFLNEASVMKTFNCHHVVKLVGVVSKGQPTLVVMELMALGDLKNYLRRHRPEEDAGLSDSPASNEAKNSPFAENDHDLPPTFKDIIQMAGEIADGMSYLAAKKFVHRDLACRNCMVAQDRTVKIGDFGMTRDIYETDYYRKGGKGQDTPPQVPLSLMIGMGV-GFSLLVILAVI----FAFWYCAKKKFGDKQMPNGVLYASVNPEYMSSAD-----VYVPDEWEVPREKITLIRELGQGSFGMVYEGEAKDVVK--DEPK-VSVAVKTVNESASIRERIEFLNEASVMKTF---NCHHVVKLVGVVSKGQPTLVVMELMALGDLKNY---LRRHRPEEDAGLSDSPASNEAKNSPFAEND--------------HDLPPTFKDIIQMAGEIADGMSYLAAKKFVHRDLACRNCMVAQDRTVKIGDFGMTRDIYETDYYRKGGKGLLPVRWMSPESLKDGVFTSQSDVW----------------------------------------------YDLMKLCWQYRQSMRPTFLEIVEILSP--EL-QPHFPEVSFYHSLD-NHGREPLEMD------------EV-----------------------------------ALDSGADT------------------------------------------ETEM----YPSGS-----------------------------EFSSTPSPPSETPYS----------------------------------------------------------------------------------------------------------------------------------------------------------------------------HM-NGSHPQ----------------------------------------------------------------------------------------------------------------------------------------------------------------------------------------NGSMNLRI----------------------------PKSTLC---------------------------------------Bflo_BRAFLDRAFT_128184/1-1336            M-------------------------------RVVDKMAGLMWAAL-------------------------------------------------------------------------------------------------------------------------------------------------------------------------------------TLVIGLGLLVP-----SNGEE---------------------------------------------------------------------------------------------------YIC-DSMDIRNRVTNL-----------------RQLENCTVIEGYLQILLIDFA----EEQDYRGL----------SFPNLVEITDYFLLYRVRGLTNLS--ELFPNLAVIRGTNLFF-NYALVVFEMLDMQKIGLYSLQNITRGSVRIEKNPNLCYLDTIDWSFIAES----GYRNNFIVDNRDEE---EC--GYCPKR--------------CRNEN--------PVV-----LQDLCW--------AEEHCQK--VC------PESCLGN---CLDG-----SSGC-CHENCIGGCDG--PTERDCVACKYFV----HNGECLIQCPPDTYQ--YKDRRCITEDECPNTTNA-----------VWKL--------HHRKCIPECPSGYTTD-VNNP--RLCTECE-G-QCPKY-----------------------------------------------------NIITELEENLGLIEEVGHYVAIVRSYALVTLDFLRSLKRIRGIQ------KENGYAFYVLDNRNLEKLFEWD----RTDLTIDEGKLFFHFNPKLCRHVILTMVEKVGLPPDTITDTDISTLTNGDQAQCSFS--RLEIEEINTRKDM-----------------IILRWSEFT-PP-------------DPRDLLSYTVSYRETEE--QGIDEYDGQDAC-GNTEWKEFDVSP-------------DQRAHIITGLKPWTQYALLVKTYTKA-GA-REGSGAKSDIVYARTDADKPTHPQDVVVYSNSSNTLIITWKPPNRPNGNVTHYIVRYKRQLEEEDSMEH-REYCIGGLKPHRPTQGL-----------EDIVNNE------------EEPNNGTIG----DGTCCEC---PKSEDEI-RIEEEE------AAFQQEFENFLHNNVYHKRE------------NET-----------------------------------RAGRRRREL--PVTAR------------------------------PFYSNQTVNVTL--------PSTNTTAPPTPTPNPNPQLE-------TTV-----WNEHMVVLTGLRHFSEYIIEVIACNA----------DVINVGCS--------GSAVELARTQADDSADNIPGNITVDVGVDVETKVKMAKLYWPKPYQPNSMVVLYNVEYKKLGAD--------GN----------YEQPQQKCVESYK-L-MEQQGYTISNLVAGNYSVRVQATSFAGNGSWSNYVTFYVEE---EDTSP--------------------------------------------------------------------------------------------------------------------------------------------------------------------------------------------------------------------------------------------------------------------ANQDPQQQVPVSLMIGMGV-GFSLLVLLAVV----FAFWYCTKKRFGDKQMPNGVLYASVNPEYMSSAD-----VYVPDEWEVPREKITLIRELGQGSFGMVYEGEAKDLVK--DEPM-VSVAVKTVNESASIRERIEFLNEASVMKTF---NCHHVVKLMGVVSKGQPTLVVMELMALGDLKNY---LRRHRPEEDAGLSDSPASNEAKNSPFAEND--------------NDPPPTFKDIIQMAGEIADGMSYLAAKKFVHRDLACRNCMVAQDRTVKIGDFGMTRDIYETDYYRKGGKGLLPVRWMSPESLKDGVFTSQSDVWSYGVVLWEMATLASQPYQGKSNEEVLKFV-IDGGMLEKPEGCPNKLYDLMKLCWQYRQSMRPTFLEIVEILSP--EL-QAHFAEVSFYHSLD-NHGREPLEMD------------DV-----------------------------------ALDSGADT------------------------------------------ETEM----YPSGS-----------------------------EFSSTPSPPSETPYS----------------------------------------------------------------------------------------------------------------------------------------------------------------------------HM-NGSHPQ----------------------------------------------------------------------------------------------------------------------------------------------------------------------------------------NGSMNLRI----------------------------PKSTLC---------------------------------------Bbel_LOC109480721_ILPR_LOC109480720/1-998 M-------------------------------RVVDKMAGLMWAAL-------------------------------------------------------------------------------------------------------------------------------------------------------------------------------------TLVIGLGFLVP-----TNGEE---------------------------------------------------------------------------------------------------YVC-ESMDIRNRVSNL-----------------RRLDNCTVIEGYLQILLIDFA----EEQDYRGL----------SFPKLVEITDYFLLYRVRGLTNLS--ELFPNLAVIRGTNLFF-NYALVVFEMLDMQKIGLYRLQNITRGSVRIEKNPNLCYLNTIDWSFIAES----GYRNNFIVDNRDEE---EC-VDYCPER--------------CRIP-------QLAAV-----LSDLCW--------AEEYCQK--VC------DTSCLGN---CMYNCQSQACSAC-CHTNCIGGCHG--PNEEHCVARKYFV----QDGKCTEQCTGGTYEVFMKTKRKTYCPVAPKKKQC----------IIGKL--------HHGKCIPECPSGYTTD-SENP--RLCTECE-G-QCPKAC--KG--------------GLVDSLAAAQRFRGCTIIEEE--LKISIR--GGDNIITELQDNLGLIEEVGQYVAIVRSYALVTLDFLRSLKRIRGQ-------DENGYAFYVLDNRNLEKLFNWD----RMNMTIDVGRLFFHFNPKLCQQVIHTMWDKVGLTGQP-SDVDVSTLTNGDQAQCEYSFHKLVISDMSSGKDW-----------------IIVRWEQFV-PP-------------DPRDLLSYTVSYRETEQ--GIEDEYEGQDAC-GNTEWRDFDVNR-------------DQTAHIITGLKPWTQYAILVKTYTKA-GA-REGSGAKSDIVYARTNADEPTHPLDVVVYSNSSNILIITWKPPNRPNGNVTHYIVKYKRQQEDVSSMES-RDYCQGGQLPHRPTQGL-----------EDIVNKE------------EEANNSTTG----DGTCCEC---PKSEDEI-RIEEGE------AAFQQEFENFLHNNVYHKRE------------NET-----------------------------------RAGRRRRELLRRVTAS------------------------------PFYSNQTMNTTL--------PTTN---------------------------------------------------------------------------------------------------------------------------------------------------------------------------------------------------------------------------------------------------------------------------------------------------------------------------------------------------------------------------------------------------------------------------------------------------------------------------------------------------------------------------------------------------------------------------------------------------------------------------------------------------------------------------------------------------------------------------------------------------------TFKDIIQMAGEIADGMSYLAAKKFVHRDLACRNCMVAQDRTVKIGDFGMTRDIYETDYYRKGGKGLLPVRWMSPESLKDGVFTSQSDVWSYGVVLWEMATLASQPYQGKSNEEVLKFV-IDGGMLEKPEGCPNKLYDLMKLCWQYRQSMRPTFLEIVEILSP--EL-QPHFAEVSFYHSLD-NHGREPLEMD------------DV-----------------------------------ALDSGADT------------------------------------------ETEM----YPSGS-----------------------------EFSSTPSPPCETPYS----------------------------------------------------------------------------------------------------------------------------------------------------------------------------HM-NGSHPQ----------------------------------------------------------------------------------------------------------------------------------------------------------------------------------------NGSMNLRI----------------------------PKSTLC---------------------------------------Spur_SPU_003915_INSR/1-1381              M--------------------MDGLARPARMGRTSVRSTTLLFFII-------------------------------------------------------------------------------------------------------------------------------------------------------------------------------------FIVCSLFCISNVA---GADAD---------------------------------------------------------------------------------------------------GVC-KSMDIRNSAEKF-----------------AKLENCTVIEGYLQIVLIDHA----TPSDYAGL----------SFPKLREITEYFVMFRVKNLQTLR--HIFPNLAVIRGDSLFF-NYALIIFEMFELQEIGLPSLQAVMRGSVRIEKNINLCYLSTVDWSLVQFQ----GMENNFIKGNKDQE---EC-FNFCPED-DGK--------RLCRKL-------TSSGD-----VAELCW--------TNEHCQK--VC------PASCPRS---C-NA-----AGRC-CHEQCIGGCSG--STASDCTACRHFL----HLGVCVGQCPNGTFQ--FKNRHCITGERCPS---------------GWRL--------FNEKCTEECPGGYMPD-FNDS--RHCLPCT-G-TCPKVC--KG--------------KTIQSVEDAADMRGCTYVHGT--LTISVH--GRGNVLKELERNLGMIEVISDALIVRRANSLVSLNFLRNLRKIEGRDG---GLEMDQYSLYVLANNNLQQLFDFSQ---HPNITIVNGKFFFHYNPKLCYSEIEELHNLTGIKEPL-SNVDVSLSSNGDQVACNTH--RLNLQ-FNFFGTF-----------------LMVNWSPPKGKG-------------DVREIVGYILSYREAPQ--RNVTELDGEDAC-GTSLWEVQYIPP-------------TETSTFVSNLKPWTQYAFMVRTYTIA-GA---QFSARSNIEYRLTPEAKASPPSSLKVISNSSSELIVTWKPPEHPNGNLTHYIITWEPQYLPIHQFDD-RDFCREKLPNLRKSESS-----------TEMETSE------------DEDTNTTMV----GAGCCMC---PIDPDEL-ARREED------ARFQKDFENQLHNTIYKRRP-------------DT------------------------------------RRRKKRDVPGSAIQD-----------AGTTPTTTSFITPIPTTPTSGFNLNGSLYPSSP------ATGVNASTTSPSRVEE-----------RVI-----GLVESFHVTGLEHFTEYFITLQACNS--------------KDCS--------RAAAMYGRSLPKESADQIESAVMINH-----TSTDRVHLTWAEPSNPNGIIVIHEIRFEMM-----------------------DSTENTECVSAEG-Y-KRVLGAELLGLKVGNYTAMVRAVSLAGEGPWTKPVMFSVPD---ITNT-----------------------------------------------------------------------------------------------------------------------------------------------------------------------------------------------------------------------------------------------------------------------------------VSIAAVII-IIVFIIGL---YSFLSPFHSYRKDQM--PDGVLYASVNPEYMSTSD-----MYVADEWEFPRDKLEIIRELGKGSFGMVYEGLAKGILP--EEEEISRVAIKSVQANASMRDRIEFLNEASVMKLI---DAHNVVRLLGVVSKGQPTYVIMEFMAQGDLKNW---LRARRPENQ------------------QDL---------------PSVPTLEQLLNMAAEIADGMSFLAARKYVHRDLSARNCLVSGEGTCKVADFGLARDIYQSDYYRKERGGMLPIRWMAPESVKDGVFQASSDVWSFGILLWEMATLGELPYQGLSNEEAGEYI-KGGNVLRPPENCPEKMQEIMMACWQYQEKLRPLFGEIILNLQDYGLL-RDSFAQNCFFLNEQLNASAAPADMS------SKEL-ETV----------------------------------SILDQNAKN------------------------------------------NENL----YTSMS--------------------------------------NNKPEGAS-----------------------------------------------------------------------------------------------------------------------------------------------------------------------------NGGNRA----------------------------------------------------------------------------------------------------------------------------------------------------------------------------------------GGSVEVPI-------------------------VTGPSSSPKDRNGSLTGNGSIPHGVGVGKCTE---------------CSkow_LOC100329033_ILGFR/1-1389           M----------------------------QLQQTAMIQYRRLFLLL-------------------------------------------------------------------------------------------------------------------------------------------------------------------------------------FTIVTLISRSE-----SRIEE---------------------------------------------------------------------------------------------------HVC-SSIDIRNGVEQF-----------------QQLDNCTVIEGYLQILLIDSA----NPSDYTHL----------KFPKLREITDFLLLFRVKGLTTLA--HIFPNLAVIRGHTLFF-DYALVIFEMQDLREIGLYSLTTLQRGGVRIEKNPSLCYLDTVNFDVLVQR-----SVKNYIKENKQQA---EC-VNMCQSS-RSR--------PCYQRT-------WLSAE-----SHDLCW--------TATHCQK--AC------PYNCHDN---CRTGI---EPLKCDCDSQCIGGCVG--NGVHNCVACRNFY----HDGRCMQNCPSDTLE--YLNRRCISRSACPR---------------GWKI--------YKNTCIKECPPDYTVN-ETDS--AECLPCV-G-PCPKVC--EG--------------RTVDSVASAQLLHGCTIINGS--LKITIS--GAGNPVAELEANLGLIELVEDYVEISHAFPVVSLSFLKRLKRIRGQN-----LHSSKYAIYVLDNQNLQHLFDWEQ---KPSLTMDRGKLFFHFNPNLCPSHITELENHVGLGGQERLDVDISTTTNGNQVACEMI--ELRLV-LWAFERH-----------------MLLGWTQYV-TD-------------DERDLLSFQVSWREAP---ENVTEFDGQDAC-GSSTWERVDLGP-------------KEYNHIITRLKPYTRYAVFVKTYTIA-GT---STGAKSVIEYATTKQDNPSAPMDLKVHPKSSSELLLKWNPPEEPNGNVTHYYVYISKLQDDVEAFSQ-QNYCEPGLKLPEHTAEP-------------IDTEK------------ESATNFTDG-------CCAC---PDPEKEL-KIAEEE-------AMRKAFENFLHNNVYVSRP----------IPNEQ------------------------------------YERIKRDELSQT--------------LPGVNTTQRILRDVAYSPGNNISVTIETQPEAPSE--DPVNGNSTTDGNEEEEEETVYD-------ISK-----VDRTDHLMVELDHFTMFYLKVQACND--------------AGCS--------PLAVSYARTLPDPLADNIPSNVTSTVI-TNKTHYREVLLEWEEPQKPNGLIIFYDIAYIKQKDDVVIETGMSGEVNENEEIQEKSEEARSICISYSQ-Y-KSLSGYLLSGLEAGNYSARVRASSLSGNGSWTASTYFVVPD---EPPTELPVIQ--------------------------------------------------------------------------------------------------------------------------------------------------------------------------------------------------------------------------------------------------------------K---------SPYSDGMVVLIGG-IVVFIKLG-------XFFCRYMNNGV--PNGVLYASYNPEYLSAAD-----VYVPDEWEVPREKIHLIRELGQGSFGMVYEGEAKHIQD--DEDK-RKVAVKTVNENASIRDRIEFLNEASIMKAF---NCHHVVRLLGVVSKGQPTLVVMELMARGDLKNW---LRSHRADEP------------------SNE--------------DKLAPTVGQILHMAAEIADGMAYLAAQKFVHRDLAARNCMVDDSGTVKIGDFGMTRDIYETDYYRKGGKGLLPVRWMGPESLKDGIFTSHSDVWSYGVVLWEMATLAEQPYQGLSNEQVLKYV-IDGNSLDKPTGCPDRMFELMALCWQYNPKMRPTFIELIENLESHGNI-EPSFTEVSFYHSVE-RHHTEDTKME-----------EAI-----------------------------------PLMDDFDHR-----------------------------------------NESE----QPNMN-----------------------------PSSPVRDGPQTSPLSSV--LVS---------------------------------------------------------------------------------------------------------------------------------------------------------------------HMPKNGVLN----------------------------------------------------------------------------------------------------------------------------------------------------------------------------------------GSAIKV------------------------------PKCTTC---------------------------------------Cgig_ir/1-1516                           M-----------------------------------------------------------------------------------------------------------------------------------------------------------------------------------------------------------------------------------------------------------------------------------------------------------------------------------------------------------IC-EDVDIRNDVNAF-----------------AELENCTVIEGNLKILLIEGA----SHQQYENL----------YFPDLVEITDFFLLYRVYGLKSLR--HIFPNLSIIRGHKLFF-NFALVVFELMDLEELGLIGLTTIERGAVRFEKNPQLCYIDTIDWTKIAVG--VQGMSEHYFKQNRIAK---EC-VDVCPSD--------------CASTPVIALTGEVSKE-----VKKRCW--------TKSYCQK--IL------TCEGLNRP--CQSGVC--DNGKC-CHPNCIGGCTG--ETDRDCKVCKNVVSMINNQAICQKQCLPGTYT--LRRRRCLLEYECTDVI-------------HYKLVKNN-QTGLPGDCVDKCLAGYLES--ADG--RTCNKCA-D-KCPKSC--NG--------------KKLETAAQSQDMFGCTIIVNH--LEIRIT--RGSNVAQELERNLGQIEEVQGYIWIHHSNTLLSLGFFKNLRKIHGKN-----SNSNNFSLLVHDNMNLQELFPENV---QNNLEILNGNMFFHYNRKLCYNKIKSFESKVKMDNKS--KNDISDSSNGDQMPCTVT--TLNLTVVKIAARI-----------------AFLRWDNFK-SS-------------DPRQLLSYVISWKEAPF--KNLSIYEGRDAC-DNDVWYTKDILNKMDKD-E------DYATALLLNLKPWTQYAVYVQTYTTS-SA---KYGAISSIVYFTTLPYYPTIPDKLRVKALAPGTLKIMWNPPANPHGNVTHYELYWRKRPLVHEKFNE-RDYCSEPLDTLLDQSDDL----------YDEEDKM------------DSSRDLGLSNNKTIQGECAC---PKTFEEE-EEERKE------REMQIAFENFLHNYVYLKRTDYGPPKPLPDLPTDLPTTSMDSKRAETLFKKNRRKFSNITLPTNLPLKPNPTLRRQRARRYSREASL---------------------------PYNASAVNGLKINLSD------SEENDTDVVNETEKDLPFNK-------VIVQTLVQKNDMSYIISNLGHFQDYRIEVIACQERDP-----RDPAKMKLCS--------IRAISLGRTLPDKNADNIDPKTIKIY--KMSNKTGEVIVYWEEPKEPNGLILSYDIQYSKVMK---------------------DVKPNTLCVPHLK-Y-KKIGGYKLQKLDPGNYSVQIRAISLAFTTNYTEPRYFVVSA---PTTTAHWPPE---------------------------------------------------------------------------------------------------------------------------------------------------------------------------------------------------------------------------------------------------------------------------TIIAVTVSGV-LLLILIVV----IGVWFIARSRFKKI--PDRPDFTSINPDY----E-----HYNPDEWEVERDKIQLIRELGQGSFGMVYEGVARDLYG--KGGE-IKVAVKTVNEHATYRERMEFLSEASRMKAF---SCNHVVRLLGVVSDGQPALLIMELMEKGDLKNF---LRMHRPVEE------------------DIDSTF----AGIKEHISRTPPTIKRIIQMAGEIADGMAYLADKKFVHRDLAARNCMVAEDLTVKIADFGMTRDIYETDYYRKGGKALLPVRWMAPESLKDGIFTSLSDVWSYGVVLWEMATLAAQPYQGLSNEEVLRYV-LNGRVMEKPEDCPDRLFELMQKCWRYRPKQRPTFKEILEELVP--EL-DPSFANKSYFFSDE-NKQDSLEYIE----EGDEDMDESKTPFI--------------QNEDECDMKGAEGGVDLPELPDQDID------------------------------------DDIDLGEDY----YPPGYQEAQHS-------------------YQNGELFPELRIDKVNNVLYSRSQADKL--------------------------------------------MLNGTEPCDCILTQEPNPDSH--PDQR-----------------------------------------FSSCSNPYSAIGSSDDSKDSSKS----------------------SSSSYAQM-NGVHIA----------------------------------------------------------------------------------------------------------------------------------------------------------------------------------------NGHIAMHH----------------------------LKTTKC---------------------------------------Lgig_LOTGIDRAFT_51396/1-1233             M---------------------------------------------------------------------------------------------------------------------------------------------------------------------------------------------------------------------------------------------------------------------------------------------------------------------------------------------------------------------------------------------------------------------EADFAKL----------SFPKLVEITGHILLYRVDGLRTLR--HIFPNLAVIRGQELFH-NYAIAAYQMQDLEELGLVSLTAIKRGAVRLEKNSKLCYVDTIDWEKLAAS---DNWKDNVFSENKDEQ---EC-VNICPDQ--------------CSRS-------KVKGK-----EAKRCW--------SSKDCQKGLDC------EKKCGKGI-FC-ND----ETGEC-CHNYCMGGCRG--PRKEDCLACKAVI----YQGICEPRCNPETYK--YMDRRCLLDKECMNMENHQK-----GE--EWKIIRGD--SGMEGKCIKECPNGYTKN-VTTPNIRECTKCS-A-HCPKVC--FG--------------RVIDSIDAAQTLTGCTSITGP--LEIEID--GGSNIVVELEKSLGEIEEISHYIKITRSYPIISLHFFKKLKLIKGEK-----LDQTYYSLNIFDNPNLQELFSPEV---TKNLRILNGKSKFNFNRKLCFYKIENFLNAVGLANTT-DALDDVKSTNGDSIPCEVN--TLNLSVQAVNHHS-----------------VILKWDQFK-TH-------------DIRPLLSYVVHYREVQS--KNINIFQGRDAC-SESVWKTKEVEPDKN----------SNPEEIISSLRPWTLYAAYIQTYTIG-RA---SSSAISNLTYFRTKPYYPSAPTNLEITAEVEGELKVTWDPPKLPNGNITYYIVKWRRQVLKREEFDK-RDYCKEPLDLNRDKKEKD-------------KKKE------------EAKKNLTLP---SGGQCCRC---PKTPQEE-EEENRI------RLNEIAFENALHDEVYVKRKREVPS------PNDI------------------------------------KSGKQKYLSGNTD---------N----------------------EDVDVKRLKGKVDG------KNSSDFNNETEENQEN-----------TSI-----VHSQEIHLPNLGHSQPYTVEVVACLSD-------KDKYGKEMCSHSHSQG--NSAIANARTLASKTADTINASTITVK-----PNKTEVMIKWEEPRKPNGLVIKFEIDYQKT-----------------------HNNDVTPCINYTK-Y-RSIKGYKLEKLQPGNYSFKIRATSLAGPGEWTPVKYFFIED---TGNDNGIEKK---------------------------------------------------------------------------------------------------------------------------------------------------------------------------------------------------------------------------------------------------------------------------VIIAIVI-GV-ILVLVITI------IVVWFAKESE----LANNLIVSHNPNYFPAGD-----VYIPDDWEVDRDKIELIREIGEGSFGMVYEGIATDLPP--YKGP-MNIAVKTVNDQAGVQDKMNFLKEACIMKAF---SCFHVVKLLGVVSQGQPAYVIMELMKNGDLKNY---LRSHRPDVE------------------DNN--------------GAPPPTLKEILQMAGEIADGMAYLADKKFVHRDLAARNCMVAEENTVKIGDFGMTRDIYMTDYYRKGGKGLLPVRWMAPESLKDGIFTTMSDVWSYGVVLWEMATLAAQPYQGLSNEEVLRYV-SEGKFMEKPEGCPDRLYDLMLKCWQYKDKNRPTFKEIIEMLVP--DL-HPSFKDVSYFFSDE-NKPP-------------------------------------------------------------------------------------------------------------------------------------------------------------------------------------------------------------------------------------------------------------------------------------------------------------------------------------------------DG-----------------------------------------------------------------------------------------------------------------------------------------------------------------------------------------------------------------------------------------------------------------------------Obim_Ocbimv22033156_IR/1-1472            MRRE-----GCEKNIDLI--LHDYCPNPTRGPDILCTATSCLFDGVC--------------------KEFENGDKRCT-------C--------------SHD----------------------------CSNE------------------------------------------------------------PTSQICGTDHK---------------IYANDCLLRKESCELQTAINKTHMDFC---------------------------------------------------------------------------------------------------ELC-SSIEIKNKIENF-----------------KQLEHCSVIEGYLRIILLDYM----KPEDYQNM----------RFPKLVEITDYLIMYRAYGLKTLR--DLFPNLSVIRGQELFY-NYALVAFEMPDLEEIGLSSLTVIQRGAVRFNKSPNLCYLETIDWTKITSE---GSNEAHSIADNKDPR---EC-VDQCPHT--------------C----------------------DHCW--------NSRECQK--NI------DCLCENRKGYC-QQ-----NGSC-CHENCLGGCRG--PTARDCLACRHVY----HDKECKRTCPPNTYK--FKNRRCLTERECRSVAY------------NFKLLEANMTTNRTALCTEHCPVGYIEN-PDDP--NRCLKCK-A-RCPKKC--PA--------------KEVKSISSIQSLKDCTEITGE--LILQHL--NG-VLAQELEANLGQIEEVHNFIKITRSDGLVSLSSFKSLKVIHGKK-----LEDDRYALYLRDNENIEELFSKDV---EKNLVIKRGKVFFHDNRRLCQHKIRELMNYTKLNASNHTEHDIS-TSNGDLIPCQTK--NLNLTIISTASAF-----------------AILKWNRFE-MD-------------DSREMLGYVIYYREVAD--KNVPLFEKQDLC-IDRLWKTVQKPKDRKEPKD------NQEFHVLTELKPWTLYATYLKTDTLS-TA---KSTGMSDVEYFRTTTGVPTNPTNLKITAKRVGELIVSWDPPKEPKGEVDHYFVYWQSEVLNVADYSI-RDYCENRLKPTAKQDEKR-----------------------------KPIANDT-----FNENCCEC---PKDKKVD-KTEESE------RQMQIFFENFLHNNVFVKRPPDVS------LSDDS------------------------------------HNRHRREAVIED---------------------------------PHAAGKYLPA----------HNNFSRPNASRQSTERPFLA-------MAV-----YNSRSVVIGNLAHFQEYNIEVIACHKS---------NENGKYCS--------NRAIVTAKTLPLEIADHINSSSVNAT--LIVNGTGDMLISWDPPAYVNGLIVTYEVAYKKAAKK--------------------NKDDSIVCITQRE-Y-QITRSCRLQKLDAGNYSYRIRATSLAALGNWTEYKFFLVPE---RPGSPPP-----------------------------------------------------------------------------------------------------------------------------------------------------------------------------------------------------------------------------------------------------------------------------LNTIMIVIITL-LSVLVTVL----IFTTICVRYGICSR--GKGASYVSVNPEYASARE-----VYEPDETELDRDMISLIRELGQGSFGMVYEGILFTSGD--DEKG-ITVAVKT----AMSADRHSFLKEATVMKLFGEESSSSGSRIIGLEPHTPGLRGHWETFEPPSLKGFGWDLEPTLPDDE------------------EYD--------------GRKPLTLHQTLCMAVEVADGMAYLADKKYVHRDLAARNCMVSLDSVVKIGDFGMTRDIYETDYYRKGGNALLPVRWMAPESLKDGIYTSLSDVWSYGVVIWEMATLAAMPYQGLSNSEVVKFV-SDGKIMEKPEGCPKRLYSLMLMCWQYKPKQRPSFKDIIKALEP--EQ-FPEFQNVSYYNSEE-NRHHMEEEAMAK-KMRGEDE-EQY--------------------------K--------PLYKNIST------------------------------------------SKDL----YTELHSDMSQS-------------------STAEALELQVIPSEADPLASTSGQLS--------------------------------------------MSGIGGSGEVSCGLRRSSSSLSANLPEEE------------------------------------------------------------SNKN----------------------GNSRISHK-NG---------------------------------------------------------------------IA---------------------------------------------------------------------------------------------------------------------NGHIQSQM----------------------------TRTPVC---------------------------------------Cele_daf-2/1-1846                        MTRM--NIVRCRRRHKILENLEEENLGPSCSSTTSTTAATEALGTTTEDMR----------------LKQQRSSSRAT----------------------EHDIV--------------------------------DGNHHDDEH---------------ITMRRLRLVKNS----RTRRRTTPDSSMDCYEENPPSQKTSINYSWISKKSS----------MTSLMLLLLFAFVQPCA------------------------------------------------------------------------SIVE-------------------------------KRC-GPIDIRNRPWDIKPQWSKLGDPNEKDLAGQRMVNCTVVEGSLTISFVLKHKTKAQEEMHRSLQPRYSQDEFITFPHLREITGTLLVFETEGLVDLR--KIFPNLRVIGGRSLIQ-HYALIIYRNPDL-EIGLDKLSVIRNGGVRIIDNRKLCYTKTIDWKHLITS-----SINDVVVDNAAEYAVTET-GLMCPRGACEEDKG----ESKCHYL-------EEKNQEQGVERVQSCW--------SNTTCQK--SCAYDRLLPTKEIGPG--C-DA-----NGDR-CHDQCVGGCER-VNDATACHACKNVY----HKGKCIEKCDAHLYL--LLQRRCVTREQCLQLNPVLS-----NKTVPIKA--------TAGLCSDKCPDGYQIN-PDDH--RECRKCV-G-KCEIVC--EIN-------------HVIDTFPKAQAIRLCNIIDGN--LTIEIRGKQDSGMASELKDIFANIHTITGYLLVRQSSPFISLNMFRNLRRIEAKS-----LFRNLYAITVFENPNLKKLFDS-----TTDLTLDRGTVSIANNKMLCFKYIKQLMSKLNIPLDP---IDQSEGTNGEKAICEDM--AINVSITAVNADS-----------------VFFSWPSFN-IT-----------DIDQRKFLGYELFFKEVPRIDENMTIEEDRSAC--VDSWQSVFKQYYETSNGEPTPD-IFMDIGPRERIRPNTLYAYYVATQMVLHAG---AKNGVSKIGFVRTSYYTPDPPT-LALAQVDSDAIHITWEAPLQPNGDLTHYTIMWRENEVS-PYEEA-EKFCTDASTPANRQHTKDPKETIVADKPVDIPSSRTVAPTLLTMMGHEDQQKTCAA----TPGCCSC---SAIEESS-EQNKKKRPDPMSAIESSAFENKLLDEVLMPRD---------------------------------------------------TMRVRRSIEDANRVSEELEKAENLGKAPKTLGGKKPLIHISKKKPSSSSTTSTPAPTIASM--YALTRKPTTVPGTRIRLYEIYEPLPGSWAINVSAL--ALDNSYVIRNLKHYTLYAISLSACQNM---------TVPGASCSIS------HRAGALKRTKHITDIDKVLNETIEWR---FMNNSQQVNVTWDPPTEVNGGIFGYVVKLKSKVD---------------------GSIVMTRCVGAKRGYSTRNQGVLFQNLADGRYFVSVTATSVHGAGPEAESSDPIVVM---TPG---------------------------------------------------------------------------------------------------------------------------------------------------------------------------------------------------------------------------------------------------------------------------------FFTVEIIL-GM-LLVFLILMSIAGCIIYYYIQVRYGKK-VKALSDFMQLNPEY-CVDN-----KYNADDWELRQDDVVLGQQCGEGSFGKVYLGTGNNVVSLMGDRF-GPCAIKINVDDPASTENLNYLMEANIMKNF---KTNFIVKLYGVISTVQPAMVVMEMMDLGNLRDY---LRSKREDEV------------------FNE----------TDCNFFDIIPRDKFHEWAAQICDGMAYLESLKFCHRDLAARNCMINRDETVKIGDFGMARDLFYHDYYKPSGKRMMPVRWMSPESLKDGKFDSKSDVWSFGVVLYEMVTLGAQPYIGLSNDEVLNYIGMARKVIKKPECCENYWYKVMKMCWRYSPRDRPTFLQLVHLLAA--EA-SPEFRDLSFVLTDN-QMILDDSEALDLDDIDDTDMNDQVVEVAPDVENVEVQSDSERRNTDSIPLK--QFKTIPPINATTSHS-----------------------TISIDETPMKAKQREGSLDEEYALMNHSGGPSDAEVRTYAGDGDYVERDVRENDVPTRRNTGASTSSYTGGGPYCLTNRGGSN---ERGAGF-------GEAVRLT--DGVGSGHLN-DDDYVEKEISSMDTRR------STGASSSSYGVPQTNWSGNRG--------------------------------ATYYTSKAQQAATAAAA-------------------------------AAAALQQQQNGGRGD-----------------------------------------------------------RL----------------------------------------------------------------------------------------------------------------------TQLPGTGHLQSTR--------------GGQDGDYIE---TEPKNYRN--NGSPSRNGNSRDIF-NGRSAFGENEHLIEDNEHHPLVTcas_LOC661524_ILR/1-1394                M---------------------------AATGTATTAQQRRSFSWL-----------------------------------------GPGPV--------LHAAL------------------------------------------------------------------------------------------------------------------LVLFGSAAFVAVAANQLQPNSDLAHQNEG---------------------------------------------------------------------------------------------------EIC-ESVDIRNTLDSF-----------------NRLKGCHVVEGFVQILLFDNV----NETELSLL----------SFPNLTEITDYLLLYRVNGLRSIG--QLFPNLSVIRGRSTFY-TYSFVVFEMSSLQEIGLYSLTDITRGLIRIDKNPSLCFVNSIDWEAIAHE-----KGEHFIKNLKSPN---EC--PICPGDEKEDESGNSVAHIHCHKAP------HRSDSENFDRDVHLCW--------NRQHCQK--IC------PTKCKHS---C-NA-----NLEC-CDESCLGGCSI--NDTKLCTVCKNLSMGFGAKKQCMSSCPPDYYQ--YLERRCILKNECKDMKRPLNFQQQGGPEKPFKI--------FNNSCILECPPNYMSN------DTHCIPCQ-G-NCIKKC--AG--------------VNVDSINLARQLKGCTHITSS--LEIQIR--GGRNVVNELEESLGMIEEIDGYLKVVRSFPLVSLNFLKNLKVIHGRQ-----LESQKYVFVVLDNQNLQELWNWEN---NKTLKIDTGRLFFHFNPKLCIREIEKLQNITHIVDVT--ELEVAKNSNGDKIACELQ--VLKVDKPKVVNSK----------------GVVLEWEPFK-ID-------------DPRKLLGYIVYSIEAPT--QNVTLYDGRDAC-GGDGWRVDDVAIPENE---------MNVTHPLTSLKPYTQYAFYVKTYTIA-TE---RRGAQSMISYFTTLPDTPTAPVGLEVVSNSSNSLKLSWRPPKSPNGKLTHYIVSLKRHNSTTPDLDEMEHFCKSPSSRPKHPIPS-----------VSVPPVT----------------STT-----TSNDTCQC---LETKPSTSSINEDV------EKSRIDFEDELHNAVYVRKP------------NFS------------------------------------ESRKRRDVDSEQ-----------------------------------------------------LNANRVFNETDEAGAYISF--------STV-----VTGTEFYMPNLRHYTVYEINVQACREKTN-----DKLDTENPCS--------SKNMKTHRTLKKKGADDIKQIEVK------NQSLGMVSLTWKEPEAPNGLIYCYTIEYKKLEIE--------------------NSKANEEYITHAR-FINQSRIYTLKALSPGNYSVRVSATTSGDYANFSPYAYFYIEE---RPSN---------------------------------------------------------------------------------------------------------------------------------------------------------------------------------------------------------------------------------------------------------------------------------TYVTLIVCML-ILVIILAL-----CAFCFYKKKKADK--ESMRLIPSVNPEYVPS-------VYVPDEWEVPRKKIELIRELGQGSFGMVYEGIAQDVR---GKAQ-IKCAVKTVNEHATNRERLEFLNEASVMKAF---DTAHVVRLLGVVSQGQPTLVIMELMANGDLKTY---LRSHRPDAE------------------VYDPAT-----------AKQPPTLKQILQMAIEIADGMAYLSAKKFVHRDLAARNCMVAEDLTVKIGDFGMTRDIYETDYYRKGTKGLLPVRWMAPESLKDGVFTSNSDVWSYGVVLWEMATLASQPYQGLSNDQVLRYV-IDGGVMERPENCPDKLYTLMRYCWQHKPSARPSFLKLCSLLLE--DA-STSFAQVSFYHSAA-GIEARSSRPT---PSPSQDD-PST-----------------------------------PLRIAGDH------------------------------------------DVNFSL-NSDDSNDEFETD-------------------AETHIRFPSIPVENKDGITTANGYVSGC-------------------------------------------------------------------PT-------------------------------------------------------------------------------------------------NG--------------------------------------------------------------------------------------------------------------------------------------------------------------------------------------------------------------------------------AATTQC---------------------------------------Tcas_LOC664271_ILR/1-1240                MQRD-------------------------------------------------------------------------------------------------------------------------------------------------------------------------------------------------------------------------------LVIIAVLFLFI-----NTGHG---------------------------------------------------------------------------------------------------KIC-PSIEIKNKPSEL-----------------EQLRNCTIIMGYLRIVLLDSVK---EESAFDNH----------TFPELTEIVDHLMLYRIPHLTSLN--RLFPNLRIIRGRRVFR-DYSLVIFKLNHLEEVGLKNLQHI-EGGIMIGGCKQLCYANTVDWNALGAF----AFHFQELP---------TCKSELCPKN--------------C---------------------VGRCW--------NKDTCQK----------PS----------TG------------------------------FCEFFE----DEGKCVKSCPRHKVH-NYDIMKCMTRKECETMLNG-----------TWWV--------FNDTCVETCPIYNNRD-KNAK--GGCVYAG-K-KYKKEC--RG--------------GLIEKLDVLQEMKDCTHINTS--LELRIT---QDYLVPYLEKYLGNVVSISGYLNVSRSGNIKSLEFFKNLELIKGEE-----LANNVYALYVFQNKNLEKLWDFDD---NFNLTIRHGSIQIHDNRFLCPSDIIRLEEIVGAKTDN--DTIIFRYSNGYNSVCNQK--NMTVKILENNPTN-----------------VTLAWQRADSTT-------------DNEVTIGYTIYYVPVLE-GNNITAFENKDEC-SVDGSYGIFV---------------RENKVQLVSLRPFTRYGYYFKIYTTN------PQEAKTPVYYFVTASTEPPPPIDFDAEAVSDTSVVLSWKPPSLFNGRFSHYELTTFVEEDYVPLIQQ-RNYCYFPHVEIEKPKVP-----IV----PKVPKLA------------EDLTNTT---------NCTC---SKKP----------------------FAQFFTEDVTCTN----------------------------------------------------GGRIDSCIRSIS-----------------------------------------------------HQPPSYFSGKRDIDNETQVK-------LFS-----SNATSYEFKNLRHYTLYVFYLRACNVRE--------DTGELLCG--------SLVMVTERTRKRKSADAIKSVRVA-------TEEKNARVRWSKPEESNSILVAYQVEYKRTDSE--------------------YSKPKSECLTEGL-FNTLGQEYNLLGLNPAKYAIRVQAISLAGPGTFSEWRYFEIVA---EPSSAYK------------------------------------------------------------------------------------------------------------------------------------------------------------------------------------------------------------------------------------------------------------------------------IYTPIIV----ISVIVCLL----GGGYYWYRKRYFKI--ERNHLITSINPDYVGP-------IYVEDDWEIDRNDIEIIRELGKGSFGTVYEGLIK-------SRQ-YPCAIKTISEKSSISSKMEFLNEGSVMKSFS--ETHHVIKLLGIVSRGDRPFVIMELMERGDLKSY---LRRCRGPSQ--------------------------------------NLTTNEIYRMASEIADGMAYLSVKKFVHRDLAARNCMVAADRTVKIGDFGMARDIYETDYYRKGTAGLLPIRWMAPESLADGVFTTDSDVWSYGVVLWEMATLAELPYQGLGAEEVLQFV-TSGRTLDMPPQCSDLLHKIMSACWKWRPNDRPTFFSIVDELVN--HV-GEDFRLVAFCFSRQ-GTELRLNAVPRV--------------------------------------------YNPPAMMGTTM------------------------------------------TGDEGFSHYDRSEEEVNL-------------------------------------------------------------------------------------------------------------------------------------------------------------------------------------------------------------------YM-NKSNRPTK------------------------------------FLPF--------------SNQRM---------------------------------------------------------------------------------------------------------------------------NKSSPYQL-SPGS-----------------------PSSTSS-FNSS----------------------------------Dmel_dInR_AAC47458.1/1-2148              MFNMPRGVTKSKSKRGKI----KMENDMAAAAATTAKTTACTLGHICVLCRQEMLLDTCCCRQAVEAVDSPASSEEAYSSSNSSSCQASSEISAEEVWFLSHDDIVLCRRPKFDEVETTGKKRDVKCSGHQCSNECDDGSTKNNRQQRENFNIFSNCHNILRTLHSLLLLMFNCGIFNKRRRRQHQQQHHHHYQHHHQQ---HHQQHLQRQQANVSYTKFLLLLQTLAAATTRLSLSPKNYKQQQQLQHNQQLPRATPQQKQQEKDRHKCFHYKHNYSYSPGISLLLFILLANTLAIQAVVLPAHQQHLLHNDIADGLDKTALSVSGTQTRWPRSESNPTMRLSQNVKPC-KSMDIRNMVSHF-----------------NQLENCTVIEGFLLIDLINDA----SP---LNR----------SFPKLTEVTDYIIIYRVTGLHSLS--KIFPNLSVIRGNKLFD-GYALVVYSNFDLMDLGLHKLRSITRGGVRIEKNHKLCYDRTIDWLEILAE---NETQLVVLTENGKEK---ECRLSKCPGEIRIE--------EGHDNT-A--IEGELNASCQLHNNRRLCW--------NSKLCQT--KC------PEKCRNN---C-ID-----EHTC-CSQDCLGGCVIDKNGNESCISCRNVS----FNNICMDSCPKGYYQ--F-DSRCVTANECITLTKFET-----NSVYSGIP--------YNGQCITHCPTGYQKS--ENK--RMCEPCPGG-KCDKEC--SS--------------GLIDSLERAREFHGCTIITGTEPLTISIKRESGAHVMDELKYGLAAVHKIQSSLMVHLTYGLKSLKFFQSLTEISGDP----PMDADKYALYVLDNRDLDELWGP-----NQTVFIRKGGVFFHFNPKLCVSTINQLLPMLASKPKFFEKSDVGADSNGNRGSCGTA--VLNVTLQSVGANS-----------------AMLNVTTKVEIGEPQKPSNATIVFKDPRAFIGFVFYHMIDPY--GNSTK-SSDDPC--DDRWKVSSPE--------------KSGVMVLSNLIPYTNYSYYVRTMAIS-SE---LTNAESDVKNFRTNPGRPSKVTEVVATAISDSKINVTWSYLDKPYGVLTRYFIKAKLINRP-TRNNN-RDYCTEPLVKAMENDLP-----------ATTPTKK--------------ISDPLAG-------DCKCVEGSKKTSSQ-EYDDRK------VQAGMEFENALQNFIFVPNIRKSKNG-----SSDKS------DGAEG------AALDSNAIPNGG---ATNPSRRRRDVALEPELDD-VE-----G--------------------SVLLRHVRSITDDTDAFFEKDDENTYKDEEDLSSNKQFYE-------VFAKELP-PNQTHFVFEKLRHFTRYAIFVVACREEIPSEKLRDTSFKKSLCS--------DYDTVFQTTKRKKFADIVMDLKVDLE--HANNTESPVRVRWTPPVDPNGEIVTYEVAYKLQK---------------------PDQVEEKKCIPAAD-F-NQTAGY-LIKLNEGLYSFRVRANSIAGYGDFTEVEHIKVEP---PPS---------------------------------------------------------------------------------------------------------------------------------------------------------------------------------------------------------------------------------------------------------------------------------YAKVFFWLLGIGLAFLIVSL----FGYVCYLHKRKVPS--NDLHMNTEVNPFYASM-------QYIPDDWEVLRENIIQLAPLGQGSFGMVYEGILKSFPP--NGVD-RECAIKTVNENATDRERTNFLSEASVMKEF---DTYHVVRLLGVCSRGQPALVVMELMKKGDLKSY---LRAHRPEER------------------DEAMMTYLNRIGVTG--NVQPPTYGRIYQMAIEIADGMAYLAAKKFVHRDLAARNCMVADDLTVKIGDFGMTRDIYETDYYRKGTKGLLPVRWMPPESLRDGVYSSASDVFSFGVVLWEMATLAAQPYQGLSNEQVLRYV-IDGGVMERPENCPDFLHKLMQRCWHHRSSARPSFLDIIAYLEP--QCPNSQFKEVSFYHSEA-GLQHREKERKER--HQLDAF-AAV-----------------------------------PL------------------------------------------------DQDL----QDREQQEDATT----------------PLRMGDYEQNSSLDQPPESPIAMVDDQGSHLPFSLPSGFIASSTPDGQTVMATAFQNIPAAQGDISATYVVPDADALDGDRGYE--I-YDPSPKCAELPTSR-SGSTGGGKLSGEQHLLPRKGRQPTIMSSSMPDDVIGGSSLQPSTASAASSNASSHTGRPSLKKTVADSVRNKANFINRHLFNHKRTGSNASHKSNASNAPSTSSNTNLTSHPVAMGNLGTIESGGSGSAGSYTGTPRFYTPSATPGGGSGMAISDNPNYRLLDESIASEQATILTTSSPNPNYEMMHPPTSLVSTNPNYMPMNETPVQMAGVTISHNPNYQPMQAPLNARQSQSSSDEDNEQEEDDEDEDDDVDDEHVEHIKMERMPLSRPRQRALPSKTQPPRSRSVSQTRKSPTNPNSGIGATGAGNRSNLLKENWLRPASTPR---------PPPPNGF-IGREA-----------------Hvul_ilr_LOC100214733/1-1477             MMRN-----------------------------------VQSFYFL-----------------------------------------------------------------------------------------------------------------------------------------------------------------------------FLLIVLNFHVVLSAVCIGQRA---------------------------------------------------------------------------------------TTTIW----------LNQN-GDC-------QDVGFC-----------------QYLQNCTCWHGNLVVKSTKYY----DEENFKPY-----------FPKLREITGYLLI----SLCTLKFFHLFPGLTVIRGGDLIL-NYALVIY-YNEIKEVYFPSLTAILNGGVHIGRNHRLCYVNTIRWKSIIKDIHQTGQYGIYLESNK-----LNC-DLGCLK-------------GHCHPAP-----GHDGDP-----KAQYCWGPGPKKQQNKAQCQR--FC------NTQCGPEG--CLDG----SDHIC-CHHECLGGCSA-INSTNTCHACRKYR--IKSTGQCVSKCPRKQYL--VDKFLC--QESCPYWSIN-------STEYHHYL--------WQGECVTKCPVNYISN--NQT--KKCEKCKSGMKCNTVC--KYQDVMADGTLYNGALIRVPSDISKKGLVGCSVFEGS--LTFQLQ--EGTGKAEDSLNELKSLKVLKGHLKIQKS-SLKSLNFLSSLEVIETPQN---ALLHNKYVMAVYENSQLSELWPG-----NESIIVSDGGIFFQYNPRLCPLHIRNLQDRIHYKNGSKVTGEVSLQNNGHKVLCDTQ--MLVMHVEEFIPPDLNMETDMTAIECNSFKCVKVTWNFTM-TS-------------AYNNILFYAIYFKELQS--NQEAVVQLDNECQNNDDWNVITVDIPKIES-LEQSL--FLQSKIISKLTPYTRYAFYIKEIVSK------GEERSSHIHYINISQDLPSEPLGVEASFLSENKILLKWRAPSKPNGIITAFKIYYNKPDYSFWEEQKVLDWCSRDASRDKNAKDV-----------AGYPVNK------------ENYNQYCNI-------SCVCDEEKENSKAI-KADREA------HNFNVEFQTELMRVLFTKNK----------------------------------------------FSYRNKNKSPPKIDFSKNIS------------------------------LILSNKILT-----------STSTTVTQAKIEIIEEPK---------VTVN----GNIFSYVISGLDYFEDYELKVCGCTV--------------VGCRRPSSTINLDCGIVQARTGVNLTADNLDSKMVRVQ-----VQLDSYNISWIAPHKPNAVILKYEISIRYAL----------------------DKDALVICRPG---Y---LPTYIIRKSRFGNYVAKIRAISPAGNGSWTEEIHFKVAE---LSVTKNN-----------------------------------------------------------------------------------------------------------------------------------------------------------------------------------------------------------------------------------------------------------------------------NQLIIGIISAV-SAVIVALLV--FILLYMFLHRKLEKD--VQGVLYASVNPEYMNSKE-----VYIPDEWELNREKIELIRELGQGSFGMVFEGIAHGIG---DHAE-LRVAVKTTNENASIHDRIQILQEASIMKAF---NCNHVVKLIGVVSQGQPTFVVMELMGRGDLKSY---LKERRPDD------------------------------------GGIPLMRQEIYQMVAEIADGMAYLAARKFVHCDLAARNCMVASDFTVKIGDFGMARDIYERNYYRKDGKSLLPIRWMAPESLKDGIFSTASDVWSFGVVLWEICTLASQPYQGKTNEQVLNFV-LSNGHLDYPEGCDYQLREFMSLCWHRDPKMRPSFLEIVHVLEN--EV-DDDFVMVSFYHEMK-RKALEDIYMK-----------SES-----------------------------------YIKSDAYT------------------------------------------MSDG----YTKGDGNMQN--------------------------MLSRSQNRKSAIEKSKERLS--------------------------------------------ISSLDSGTYVE---KYDANDTPEEIPKKK-KRPRS--------------------KRNSAVDS-------------------------------------------------------------NACETK--------------------------------------------------------PMLRV-------------------------------------------------------------------------------ESLYDNHDAF----------------------------------SENMQYGD-TPVG------------KSDLMH-----PETNRE---------LRLSEIF-YGKPIP---------------V3. IGF2R	a. IGF2RHsap_IGF2R/1-2491                   M---------------------GAAAGR--------------------------------------------------SPHLGPAPARR---PQRSLLLLQLLLLVAA------PGSTQ-AQAAPFPELCSY-TWEAVDTK-NNVL--YKINICGSVDIVQ-------CGPSSAVCMHDLKTRTYHSVGDSVLR--------SATRSLLEF--------------NTTVSCD-QQGTNHRVQSSIAFLCGKTLGTPEFVTA-TECVHYFEWRTTAACKKDIFKANKEVPCYVFDEELRKHDLNPLIKLSGAY-LVD--DSDPDTSLFINVCRDIDTLRDPGSQ----LRACPPGTAACLVRG-HQAFDVGQPRD-GLKLVRKDRLVLSYVRE-EAGKLDFCDGHSPAVTITFVCPSERR--EGT-IPKLTAKSNCRYEIEWITEYACHRDYLE-SKTCSLSGEQQDVSIDLTPLAQSGG-------SS-YI-------SDGKE-YLFYLNVCGETEI-QFC----NKK--QAAVCQVKKSDTSQVKAAGRYHNQTLRYSDGDLTLIYFGGDECS-SGFQRMSVINFECNKTAGNDGKGTPVFTGEVDCTYFFTWDTEYACV-KEKEDLLCGA--TDGK-KRYDLSALVRHAE--PEQNWEAVDGSQT---ETEKKHFFINICHRVLQEGK-ARGCPEDAAVCAVDK----NGSKNLGKFISSPMKE--KGNIQLSYSDGDDC---GHGKKIKTNITLVCKPGDLESAPVLRTSGEGGCFYEFEWHTAAAC----------------------------------------------VLSKTEGENCTVFDSQAGFSFDLSPLTK----KNGA-YKVETKKYDFYINVCGPVSVS-PCQ------PDSGACQVAKSDE--KTWNLGLSNAKLSYYDGMIQLNYRGGTPYNNE--RHTPRATLITFLCDRDAGVGFPEYQEE---DNSTYNFRWYTSYACPEEPLECVVTDPSTLEQYDLSSLAKSEGGLGGNWYAMDNSGEHVTWRKYYINVCRPLNPV------PGCNRYASACQMKYEKDQGSFTEVVSISNLGMAKTGPVVED-SGSLLLEYVNGSACTTSDGRQTTYTTRIHLVCSRGRLNSHPIFSLNWE-CVVSFLWNTEAACPIQTTTDTDQACSIRDPNSGFVFNLNPLNS-----SQGYNVSGI---GKIFMFNVCGTMPVCGTIL-------GKPASGCEAETQTEELKNWKPARPV-GIEKSLQLSTEGFITLTYKGPL--SAKGTADA-FIVRFVCNDDVYSGPLK--FLHQDIDSGQGI-RNTYFEFETALACVPSPVDCQVTDLAGNEYDLTGLSTVRKPWTAV--DTSVDGRKRTFYLSVCNPLPYIP----GCQGSAVGSCLVS-----EGNSWNLGVVQMSPQAAAN----GSLSIMYVNGDKCG-----NQRFSTRITFECAQISG-SPAFQLQ--DGCEYVFIWRTVEACPVVRVE---GD-NCEVKDPRHGNLYDLKPL--GLNDTIVSA---GEYTYYFRVCGKLSSD--VCPTSDKSKVVSSCQEKREPQ---GFHKVAGLLTQKLTYENGLLKMNFTGGDT-CHK----VYQRSTAIFFYCDRGT---------QRPVFLKETSD--CSYLFEWRTQYAC----PPFDLTECSFKDGAGN-SFDLSSLSRYSDNWEAIT--GTGDPEHYLINVCKSLAPQAGTEPCPPEAAACLLG------GSKPVNLGRVRDGP-QWRDGIIVLKYVDGDLCP-DGIRKKSTTIRFTCSESQ-VNSRPMFISAVE-DCEYTFAWPTATACPMKS---NEHDDCQVTNPSTGHLFDLSSLSGRAGFTA--AYSEKGLVYMSICG--ENENCP-PGVGACFG------QTRISVGKANKRLRYVD-QVLQLVYKDGSPCPSKSGLSY--KSVISFVCRPEAR--------PTNRPMLISLDKQTCTLFFSWHTPLACEQATECSVRN------------GSSIVDLSPLIHRTGGYEAYDE---SEDDASDTNPDFYINICQPLNPMHGVPCPAGAAVCKVPI--DGPPIDIGRVAGPPI---LNPIANE---IYLNFESSTPC--LADKHFNYTSLIAFHCKRGVSMGTPKLLR-TSECDFVFEWETPVV-CPDEV---RMDGCTLTDEQLLYSFNLSSLSTST---FKVTRD--SRTYSVGVCTFAVGP-EQGGC-KDGGVCLLSGTKGA--SFGRLQSMKLDYRH-QDEAVVLSYVNGDRCPPETDDGVPCVFPFIFNGKSYEECIIESRAK--LWCSTTADYDRDHEWGFCRHSNSYRTSSIIFKCDEDEDIGRPQVFS---------------------------------EVRGCDVTFEWKTKVVCPPKKLECKFVQK--HKTYDLRLLSSLTGSWSLVHNG-VSYYINLCQKIYKGPLG--CSERASICRRTTTGDVQVLGLVHTQKLGVIGDK------VVVTYSKGYP----CGG-------NKTASSVIELTCTKTV-GR-PAFKRFD--IDSCTYYFSWDSRAACAVKPQEVQMVN-GTITNPINGKSFSLGD--------IYFK---LFRASGDMR----TNGDNYLYEIQLSS------ITSSRNPACSG-ANICQVKPNDQHFSRKVGTSD--KTKYYLQDGDLDVVFASSSKCGKDKTKSVSSTIFFHCDPLV---EDGIPEFSHETADCQYLFSWYTSAVCPLGV------------GFDSENPGDDGQMH--KGLSERSQAVGAVLSLLLVALTCCL--LALLLYKKERRETVISKLTTCCRR-SSN--VSYKYSKVNKEEETDENETEWLMEEIQLPPP--RQGKEGQENGHITTKSVK--ALSSLHGDDQDSEDEVLTIPEVKVHSGRGAGAESSHPVRNAQSNALQEREDDRVGLVRGEKARKGKSSSAQ---------QKTVSSTKLVSFHDDSDEDLLHI-----------Mmus_IGF2R/1-2483                   M--------------------------R--------------------------------------------------AVQLGPVPSGP---RVALLPPLLLLLLLAA------AGSAQ-AQAVDLDALCSY-TWEAVDSK-NNAV--YKINVCGNVGISS-------CGPTSAICMCDLKTENCRSVGDSLLR--------SSARSLLEF--------------NTTMGCQ-PSDSQHRIQTSITFLCGKTLGTPEFVTA-TDCVHYFEWRTTAACKKDIFKADKEVPCYAFDDKLQKHDLNPLIKLNGGY-LVD--DSDPDTSLFINVCRDIDSLRDPSTQ----LRVCPAGTAACLLKG-NQAFDVGRPKE-GLKLLSKDRLVLTYVKE-EGEKPDFCNGHSPAVTVTFVCPSERR--EGT-IPKLTAKSNCRYEVEWITEYACHRDYLQ-SESCSLSSEQHDITIDLSPLAQYGG-------SP-YV-------SDGRE-YTFFINVCGDTKV-SLC----NNK--EAAVCQEKKADSTQVKIAGRHQNQTLRYSDGDLTLIYSGGDECS-SGFQRMSVINFECNKTAGKDGRGEPVFTGEVDCTYFFTWDTKYACI-KEKEDLLCGA--INGK-KRYDLSVLARHSE--SEQNWEAVDGSQA---ESE-KYFFINVCHRVLQEGK-ARNCPEDAAVCAVDK----NGSKNLGKFVSSPTKE--KGHIQLSYTDGDDC---GSDKKISTNITLVCKPGDLESAPVLRAARSDGCFYEFEWHTAAAC----------------------------------------------VLSKTEGENCTVLDAQAGFSFDLSLLTK----KNGA-YKVETEKYDFYINVCGPVSMD-PCQ------SNSGACQVAKSG---KSWNLGLSSTKLTYYDGMIQLSYRNGTPYNNE--KHTPRATLITFLCDRDAGVGFPEYQEE---DNSTYNFRWYTSYACPEEPLECMVTDPSMMEQYDLSSLVKSEGGSGGNWYAMENSREHVTRRKYYLNVCRPLNPV------PGCDRYASACQMKYENHEGSLAETVSISNLGVAKIGPVVEE-SGSLLLEYVNGSACTTSDGQLTTYSTRIHLVCGRGFMNSHPIFTFNWE-CVVSFLWNTEAACPIQTITETDQACSIRDPSSGFVFNLSPLNDS----AQGHVVLGI---GKTFVFNICGAMPACGTVA-------GKPAYGCEAETQIEDIKDLRPQRPV-GMERSLQLSAEGFLTLTYKGS---SPSDRGTA-FIIRFICNDDIYPGAPK--FLHQDIDSTRGI-RNTYFEFETALACTPSLVDCQVTDPAGNEYDLSALSMVRKPWTAV--DTSAYGKRRHFYLSVCNPLPYIP----GCHGIALGSCMVS-----EDNSFNLGVVQISPQATGN----GSLSILYVNGDRCG-----DQRFSTRIVFECAQTSG-SPMFQFV--NNCEYVFVWRTVEACPVIREE---GD-NCQVKDPRHGNLYDLKPL--GLNDTIVSV---GEYTYYLRVCGKLSSD--VCSAHDGSKAVSSCQEKKGPQ---GFQKVAGLLSQKLTFENGLLKMNYTGGDT-CHK----VYQRSTTIYFYCDRTT---------QKPVFLKETSD--CSYMFEWRTQYAC----PPFNVTECSVQDAAGN-SIDLSSLSRYSDNWEAVT--RTGATEHYLINVCKSLSPHAGTEPCPPEAAVCLLN------GSKPVNLGKVRDGP-QWTDGVTVLQYVDGDLCP-DKIRRRSTIIRFTCSDNQ-VNSRPLFISAVQ-DCEYTFSWPTPSACPVKS---NTHDDCQVTNPSTGHLFDLSSLSGRAGINA--SYSEKGLVFMSICE--ENENCG-PGVGACFG------QTRISVGKASKRLSYKD-QVLQLVYENGSPCPSLSDLRY--KSVISFVCRPEAG--------PTNRPMLISLDKQTCTLFFSWHTPLACEQATECTVRN------------GSSIIDLSPLIHRTGGYEAYDE---SEDDTSDTTPDFYINICQPLNPMHGVPCPAGASVCKVPV--DGPPIDIGRVTGPPI---FNPVANE---VYLNFESSTHC--LADRYMNYTSLITFHCKRGVSMGTPKLIR-TNDCDFVFEWETPIV-CPDEV---KTQGCAVTDEQLLYSFNLTSLSTST---FKVTRD--ARTYSIGVCTAAAGL-GQEGC-KDGGVCLLSGNKGA--SFGRLASMQLDYRH-QDEAVILSYVNGDPCPPETDDGEPCVFPFIYKGKSYDECVLEGRAK--LWCSKTANYDRDHEWGFCRQTNSYRMSAIIFTCDESEDIGRPQVFS---------------------------------EDRGCEVTFEWKTKVVCPPKKMECKFVQK--HKTYDLRLLSSLTGSWDFVHEG-NSYFINLCQRVYKGPLD--CSERASICKKSATGQVQVLGLVHTQKLEVIDET------VIVTYSKGYP----CGG-------NKTASSVIELTCAKTV-GR-PAFKRFD--SVSCTYYFYWYSRAACAVRPQEVTMVN-GTLTNPVTGKSFSLGE--------IYFK---LFSASGDMR----TNGDNYLYEIQLSS------ITSSSYPACAG-ANICQVKPNDQHFSRKVGTSD--MTKYYVQDGDLDVVFTSSSKCGKDKTKSVSSTIFFHCDPLV---KDGIPEFSHETADCQYLFSWYTSAVCPLGV------------DFEDESAGPE---Y--KGLSERSQAVGAVLSLLLVALTGCL--LALLLHKKERRETVINKLTSCCRR-SSG--VSYKYSKVSKEEETDENETEWLMEEIQVPAP--RLGKDGQENGHITTKAVKAEALSSLHGDDQDSEDEVLTVPEVKVHSGRGAEVESSQPLRNPQRKVLKEREGERLGLVRGEKARKGKFRPGQ---------RKPTAPAKLVSFHDDSDEDLLHI-----------Ggal_IGF2R/1-2561                   MPPPLPTTVKRNHRPQHVPSGAVRGAGRKRSHTPRPPPGGRARHVIAMPAHDGGHMTRQWAGGGHMTCRWRPALLDVPGQPLRPGPARAAAMARAAFAPLLLVLLCLP-LGDGVAAPVS-PDEPFFQDLCSY-TWEAIDTD-KHVL--YKINLC--FGVEE-------CGRSSAVCAYDVDKRAYMSVGSLTLR--------EISKTLLVF--------------NTTSKCS-QQGSEHRIQSNINFLCGKTLGTPEFVTA-TECVHYFEWRTFVACKKNLFKPVKEVPCYVFDEDLKKHDLSPLIRVPGHY-LVD--DSDND-SLFINICRDIGR---SSGE----TMNCPAGSAACLIHE-GHAYDVGRPQD-QLKRHDKDRLILSYERTYNDEKLNFCLGHNPAVTITFVCPSKRG--EESAGPKLTAKTNCRYEVEWVTEYACHRDYLE-SKSCVLTNEQHDVSIDLSPLTLSPD-----YVTP-YLAK-----DDKEE-YYYYLNVCGRTGA-GNC----EGSMTYTSACQV-KSSTNQKKVTGRFENQTLRYSDGDLTLIYPNGDACS-SGFQRMTVINFECNETAGNDGRGTPVFTGEVDCTYFFTWYTKYACV-KEREDLLCRV--ADKK-KHYDLSPLIRSSE--SAQNWEAVDSNLS---EEWRKRYYINVCHKVLKRGG-ASGCPDAAAICSVDK---KNNSKNLGTFVSPPKKV--GENIQLTYSNGGSC---GGNKKIKTVITLICRPGDLESAPILI--SEGVCSFTFDWYTAAAC----------------------------------------------VLSKMEGDNCQVSDPQAGFSFDLSPLTK----KNGH-YTVNTEEYLFYINVCGSVPNE-LCH------TQSAACQVTQRKD--QFWSLGLPNSRLSYYDGLIQLTYKNGTAYNNE--KKTQRSTLITFLCDRQAGIGQPEYQVE---DNYTYNFRWYTEYACPEMPLECIVTDPNTMDQYDLSSLAKSE-KRGENWYAMDNSGPN-ERKKYYINVCRPLLAV------PGCDRRASVCQMEYRHDHDSYYEVTSISNLGVASKELVVER-LGHILLTYANGSVCINADGERTSYTTTIHFVCSRGTLNSSPRFISIQE-CVVTFLWETEAACPIKETKDDSQSCSVRDPNSGFLFNLQPLAA-----EKGYTTTGI---GKTYLLNICEAMPECGKIN-------GKPAAGCEAD-------NLTSVRMV-ELDKTLYLSSEGFLTLTYRGPL-LVESGKSDT-FTVTFICNDS-YPGELK--FVREEINSMLNI-HDTFFEFHTALACAPAPVDCQITDAAGNEYDLSDLSKEGKPWVAI--DTSKDAKKRTFFLNVCKPLPFVP----GCPGGAIGSCVKY-----ADKSKNLGVIQINPQAATD----GSLSIIYLNGDMCK----DKRRYSTRIIFQCDQTMG-SPVLEQE--DNCEFVFVWRTLAACPVHKAE---GE-DCQVKDPRYGHVYNLKPL--SSKDIKVST---DEYDYYFRVCGEITE---HCRPG--AHSVSSCQVKKTDS---TFRKVAGLLTEKLTFKNGLIMINYTSGEK-CHK----IYERSTAILFYCDKTT---------SEPVFLKETPD--CTYMFEWHTQYAC----PPVKSTECSYRDDEGN-FYDFSSLTRHRENWEATD--ISTSTKIYYINVCKPLVPYGAAHSCPPDAAASLVE------GIKCVSLGEVAEGP-RWENGISTLKYINGELCP-DKIRRKTTILRLKCDESK-IESKPELIMAIE-DCEYSFLWFTAAACPLKS---NVQNDCRVTNPATGHLFDLTSLKRESGYTI--TDSHNRKIELNVCAE-AKSSCA-NGAAVCIT----DGPKTLNAGKLSKTLTYED-QVLKLVYEDGDPCPTDLKMKH--KSYFSFVCKSDAG--------DDSQPVFLSFDEQTCTSYFSWHTSLACEEEVSCSVLN------------GSSVIDLSPLIHRTGYYEAFV-----DGDQSDVSPDFYINICEPLNPIKDVNCPPGAAVCMVPV--NESPIDIGRVTEPPK---LNEAVNE---VYITYNSTTPC--QINNKLNYTSLIVFHCSQGTSLGKPKMIQ-KLDCSFVFEWETPVV-CPDRV---KTLGCSVTDEQLHYTFNLTSLSGRS---FEVLSG--SSRYHVSVCSKAAADVSQGKC-KDGAVCMTSGSGVS--SFGSIKEMKMNYSR-QDETVILQYTGGDRCPPVTEKGELCVFPFKYKGKSYDKCITEEKNR--PWCATAVDYQGDGKWGFCVNATARRASTIIFVCDENAGSGSPRLLS---------------------------------DMLGCAVTFEWRTQAVCPPKKMECKFVQK--HRTYDLRILSSLTGSWVFYHNG-NSYYLNLCQRVYEGPTG--CPERASICRKSNNGDVEVLGLVHTQKLNVTGDT------VYISYSGGQE----CGK-------NKKIMTIIELRCAKTV-GM-PMLQRFD--EENCAYFIIWDTRAACAVKPQEVEVVN-GTVINPATGKNFSLGD--------VYNK---LYMASGDIR----TNGDQYVYEIQLSG------ITNSSFPECSE-ANICQVKTSE-RRFRKIGWAK--KAKYYVEDDDLDVIFSSDSRCGKDKSKFVSSSIFFHCSPHV---QEGIPEFLHETADCQYLFTWYTSAVCPLISTIAP--------GIHGGQSDQEAQVY--KGLSRRSQAVGAVLSVLLVVLTACL--IILLFYKKERRETVVNKITNCCRR-TSG--VSYKYTKINSEEEANENETEWLMEEIAAPNQ--RTVKGVQENGHVTTKSVTSDAFTSLHVDDLDSEDEVLTIPDVKIQTGRGLD-KSKHPQKKPPR----FASDDKAYLLNGGKERKAKAKPGQ---------QQAQNSTNSVSFHDDSDEDLLNV-----------Locu_IGF2R/1-2496                   M-----------------------------------------------------------------------------GPPFEHIPTIT--CHVVVVRGIITVLTLLS------SIAIS-DESLWYQELCRF-KWEAVDPD-GNVH--YDIKLCSSSPSTT-------CGETCAICAKNLTTKQSLSVGEVSML--------TASPNVMTF--------------NTTQKCS-DPNAKQNFQSSISFLCGKTMGTPEFVTV-SECVYYFEWRTYVACKKDKFKPHKEVPCYVFDADGKKHDLNPLIKITDGY-LVD--DSDTDVDLYINICRSITY----------SGNYCPEGSSACLITSKGEYLDMGHPTE-SLELLDKDRLMLRYIGV-TEGKPEFCGDHTPAVTITFVCPSGRQ--EGS-DPRLTAKENCRYEVEWVTEYACHRDYLE-TSDCKFSSAEHDISIDLTPLHIDAN-----SETP-YRATS--TSSDGKDTYFYYLNVCGETSA-GEC----RDTKGSISSCQV-KQDGSLTKVAGRYKNQILRYSDGDLTLIYPGGNSCS-SGFQRMTIINFECNQTAGNNGKGSPVFVGESDCTYFFNWQTSHACV-KEKENLLCRV--TDKK-KRYDLSALTRYSKPGSNQNWEAVDSTSS---KSEKKHFYINICHRILQNEA-TKGCPEEPAVCAVGQ---DGKARNLGKFVSSPIKD--NDNIRLTYTEGDTC---KKDTKIKTVITLLCKPGDLESAPLLRSVSSDECVYEFEWHTAAAC----------------------------------------------VMSMTVGDNCIVSNPQAGFSFDLSPLRN----ADG--YSVSSGDYKYFLNVCGSVKEG-ICT------ANAGACQVDKSG---SSWNLGDFNSKLSYYDGLIHLNYRNGSKYNNR--LHTQRSTFISFLCDREAGVGLPEYQVE---DNYTYNFKWYTSYACPEMPIECMVTDLKTMQQYDLSRLSKSE-GSDENWQAMDSSQPG-NRRKYYINVCRPLNHI------PGCDRYASVCEMKYEVQEGIMVEKVSVSNLGIAKKGPMIED-ENHLLLEYSDGSACVNEDGNRMAYTTRIHLVCSRGILSSGPRFLMNQN-CTATFLWETEAACVIST-SEESNTCTLKDPSTGFEFNLQPLAS-----KTGYEALGN---GKKFKVNICDSLDECGT---------DKPAAGCEIE-------DGKPLGPV-GVTKSLQFSTEGLLTLTYKGDL-DVATGISKT-YIINFICDQDIYPGTLN--LVTEEMSSTTHVTHDVFFEVHTALACAPAPVNCEVTDSFGNEYDLSDLSRDDAPWQAI--DTSADAKQRTFYINICKPLPLVR----GCPGGPLGSCASF-----PDHSFNLGYIQASPQAAPD----GSLSIVYLNGDKCG-----QSRYSTRIIFQCDDSPG-SPVFDRL--NGCEYVFIWRTSEACPIHRAQ---GD-HCRVRDPRSGYVFDFTKL--SGKDYEVKS---GQYNYRFSVCSPLHTK--KCKNS---ESASSCQEDTAQQ---DFPRIAGLATQNLTYEDGLIMINYTNGEK-CHK----IYERSTAIVFICDHSKN-------YGTPVFLKETLD--CTYMFEWHTELAC----LPFKTIECSFKDGNGN-SYDLSPLSLHNTNWVVEPMIGSTQ-KRYYINVCKSLVQQNGSWECPSSAASCMKN------GSQYVSLGEVESGL-QWENNVLFLQYINGEKCP-DGKRNRMTIIRFKCDEDK-VDSKPTLITAIE-DCVYTFMWFTATACPLKS---NVHKDCKVTNPVTGYLFDLNVLNIKGGYTVYDSTNKKKLIRLNVCGGVADSGCG-PDAGVCIK----DTKTAVNAGLVSKTLTYLD-QVVQLTYENGDLCPGSSSLRH--KSIFSFVCKSQAS--------SADGPVLVATDKEKCTHFFSWHTPLICEQQVSCSVKN------------GSSIIDLTPLIHKTGYYSATD-----AELGRDNSPDFYINICQPLNPIPGVTCPPGAAVCLDPV--DGLPIDIGRITGPPQ---INEAINE---VYISFNSNTVC--LSDKSKNYTSLILFSCQRGTDLGSPQMIR-KSPCSYVFEWATPVV-CSDSV---STSGCALKDDQLQFTFSLSSLTGGS---YQVPSG--SKSYQINVCAPVTD----AKC-QDSAVCLVLNNAAY--SFGNFKAMSMDYRH-EDQAIIMKYGSGDPCPTVTTKGEVCTFPFKYHKKSYNSCTTDGRTNGDLWCATTDDYNRDQKWGFCTNATGKMSSTIIFKCDRSADPGSPQLLS---------------------------------ETLGCATTFEWKTSVACPPKKMQCKVVSN--HKTYDLHALSSLTAPWKFSHGS-DSYYINFCQEIHGGLTD--CPVTAAVCRQTKTKKTQTLGLFYTQQISVKDEK------IYINYSRGDE-V--CEN-------GIQAKTIVQLECGKTM-GI-PTFQSLD--EKNCEFWLHWETRAACVMTQQEVQMIN-GTITIPDTGASFSLGA--------IYFR---MHTAYGDIR----SNGDKYIYNIQLSG------ITD-TSSGCLG-ANICQVKVSG-EYKRKIGHSS--SAKYYVKDGNLDVVIPSDSLCGRDKSKNVSSTIIFQCNPSA---GEGIPEFLLEADDCQYLFHWHTSVVCDLVAV------------MDSGKNDKSDSGL--IGLSGRSQAVGAVLSVLLVVLTACL--LILLLYKRERRELVLQKVTGCCRR-GGN--ISYKYSKISTEEDGAEDETEWLMEEVATPDSLSRVAKECQENGHITTKPVNADAFNAFPLDEQDSEDEVLTVPGVRIQSSQPSL-EWQNGKASPKRPLLEQESDDDLVEFLGEKKTRPKCK-GK---------TQRKRAENLTSFHDDSDEDLLKSHRCYDTTRQAACint_LOC100186527_IGF2R/1-2335      M-------------------------------------------------------------------------------------------IIKYTLYFAAILFIASPTYAEVFQSCENVHGKNLSQLT--GNWTTYRDG-GDTVQHYLIDICQTVNQAG-------CD-GSSICWVQ-NNGVGKSLGDAAMPPNVMYQSASSVSFYLEY--------------NTTKLCQSNSNGTFFYRSSITFECGKTLGSPVVISS-DICSVDFIWETSLACRTSEYDAINEVRCYAYDSNHHKRDLESLIKRVGGH-KVTDPTSSISDKIYINVCRDIRS--VGGDSEFSNVGQCPEGSAACLVKE-GVGYALGFVGS-PLSIGENNRMVLTYHGE-ASNSPPECLGNIPQVKINFVCPGADR--GGSRPPIITSSTNCQYSIEWWTEAACITDFVT-STTCGLDLARHGVDLDLSPLTLHGNH---------YTVNI----HDTAD-TVFYINVCEATG--TSC-GA-KRDKTKNTVCQT-TSSNTLGYIGGELDGYRVKYFDGQLTLTYKDGDRCHGNGFRRTSVILFQCNKTA---GVGSPIFSHELHCTYYFEWQTSHACI---AADPSCHL--VDEKGQNYDLSPLTRTLD-SHNTNWEVLDGSETA--SNNRRRYFVNVCGEVIRDAD-TVNCPPSSSVCLVQG---ANTPLSFGSFSRSKITA-KSNTIMLNYT-GGVC---ASGGMKTALITMYCRLGDLESSPVLKYRSSDDCVVSMTWHTAAAC----------------------------------------------PLKTVTGTGCMVSDEDSGLTFDLRRLTQ----SEHM-YKIPVGDYDYYLNVCGELKNT-PCNNQN---NHPAVCQSKRSGQ-MRSFTTGQVSDKLTYYDGFIKLVYTDGDSYSN---KLHNRSTQISFLCDNSRGVGHPEFVNE---GNFTYLFNWYTSYVCPAASVQCMATDPNTGDQYDLSSLAKSEDNEDANWSAMGGEDTVKGKTKYYLNVCRPVNEVQGTSAMDSCDALAAACSTTVDANG---QEKVGISNLGKPTSAPIIES-KGHLSITYTNGEACV-ENGVSKNFTTTIHFVCVKGAISSTPSFIEKSA-CSVSFMWNTETACPITS--ITQGSCEVVDPNSRLTYNLRPLMR---KADNPYRVDGVH--GYQYYINICGSVSSCQATT--------SPIGACRVESY-----TNAIDQKA-TNTSSLSYSDDGQLSLTYQSSQ-RNQDGSFLT-YVVNFICNHTVAVGEPK--LTDVDGHI-------IRLDFQTSLACQPDTVDCIVADDMGNQYDLTPLSNPNHPWDVV--DTQV---SRRFYVSVCRPLPAVS----GCPGGGTGACFIQHATDNTIQGHSLGLIQSNPTAVTQNGTASTVSIRYMGGDVCE--ADHTMRYSTRILFICSTTKGRSPVLQPNSDTPCDFVFVWETPFACPIKKSITKKGH-PCSVVEPLYQMTFDLSTMT-STTDYTVSGG--TEWDFKLNICSKLNNGIGACEQS------GACQVSKTKA---NKVHTAGNPNTTLIYDDAVIKLTYLHGER-CHN---NQFERTSEFRFTCHHHGN--------DKPTFINETHD--CTYLFDWPTPKAC----PPIKIIECTAQDSEGH-QYDLSALSLMHDNHMISSSQTG---RSYYINVCRSMLHRPT-VTCPPNSAACVRN-----ADNSFTNLGSVDSSP-HFVDGKLQLCYTHGDNCR--GNQNYTTTVTFECSQTSAVQTTPIVTSTED-ECNVQILWTTSAACPVQQG--NTLGSCKTSNPATGHEFDLSSLESTTFHTT--QALHGKEYHINVCEKVNDPHCP-ANSGNCLH----GSGSYISYGEANANLTYTD-GALALVYEHGDICPADDRYRH--STEISFVCHRSTSPNLQAPETPSTTPIHISDDEDTCTHYYTWSTYLACEQQFPCAVNDM--------TSAVPRIIDLSPFVRESGHYPTISNI-------VGDRGTYFINICRPLQPIQGVTCPPGSAACEVIP--GHQPQSLGSIDPTHHSMSYESETNT---VSLVYHSNRRCTTSSNATATVTTQIVFSCLQGAGKGEPRLTAVTSDCVYLFSWPTDLILCDSQTTSPSSGECKFTDEAKKLTYDLSKLETKT-----ITDT--IGSIQVSLCQPLSS---PPEC-QGAAVCYED-TSHHKVSLGSASTRTFSAGFSSEYPLTAHYADGAVCG--------------------------------------------------RTPNKHRSTTILFICDMN--PTEPELIH---------------------------------NGDECLYTVYWKTPLVCPPVSESCVVADTTLGLTFDLSLLSSLTHAWHFRNNG-YSYYFNLCRAVHTTGSHQ-CKDGSSVCRKK-GAEAKSFGIVSSQKTSVVSAT-----EIEITYTGGDKGL--CTS------GTAPLKTIIRLQCSPTIMGN-PQFESFS--ENSCELRVAWKTRIACSDSPTSVTPDRTGKFVDPKSGVTFDLSP--------LMNKR--SWVAGGDLR----EDTSSYKYMISIRGNPDISSVNPSYSQACGDIATVCQYKKDT-NFVRKIGELSPHSPTYVMSDGTLELNFTSNDKCGKDPKKTSYVIISLSCGDN----GLGSPTFFYESNDCGYYFHWYTSVMCADIEIVSS----------NKTNSRSNTPVSNPGSSTGQHSNAGGIRG-----WSFC-----------DRRGFVYS--RNCVP------------------------------------------------------------------------------------------------------------------------------------------------------------------------------Blan_BL04963_IGFR2_evm0/1-2431      M-------------------------------------------------------------------------------------------------------------------------------ISLYQLWEARGD--NNVQ--YNISICSQLALPTP------CS-GNSVCRL--QDNTTANIGNFNST-LQIEEDRHGGGFRMEL--------------FSDEDC--PGHPGHKIGSSINIQCGRTLGFPEFVEA-KPCTTYFDWDSTVACTSVQNPAQKEVKCYVYDTDGKKRDLTPLIKQSGGY-LV---DSAGERDFYINVCRDIT----AGGS----TSPCPPGTAGCRVIG-GEAVDMGRPTT-DLQMGEDGRLFLHYNST-DGDAPTGCNGVKPTVHVTFVCPNGDG-RGGSKDPLLTLDTDCQYQVEWWTEYACPAEYLT-TTTCSFSKQQHDIDIDLSPLNVPEG-----TNPWPYKITT--TGDDGKS-YDYWLNVCGAVG--ISC-GDNDDDGSHTSVCQT-SADT--AHIAGAI-GHTLRYSDGELTLTYKHGEVCKHNGFNRTSVITFRCNKTADNDGKGRPVFNSEEDCSYFFDWDTSYACL-QHEEGRSCRVSSPDGK-KRYDLSTLVQE----GPLNWEALDGRHEHEGGDNDERFFMNVCGEVITLEQ-AADCPQGAAACAISDT--GGGATNIGRYQTDPVLE--GNHIKLTYTDGDSC---HDSLTWSTVIRLVCSPGDLESAPVLLRRSDEKCLYEFEWHTAAAC----------------------------------------------PLGRKSGYDCRVFDDDAGFSFDLTPLSRAGTTADSF-YNVTNGKYDYLINVCGDINGTKSCEDRN---TKAGACQVDRSDN--KAKSLGVFNSHLNYYDGLINLTYSEGDPYHD----KVNRSTHIAFLCDPKAGSGQPKFLAE---DNHFYYFKWYTKYACPEQPIECVVTDPNTHTQYDLSSLAKSEDDNGENWSYLDDTDPN-NKKKYYINVCRPINPV------SGCDTYAAVCQTSFATGE----EAVTLPNMGMAELRPTIEGQAGHLLLTYSNGGPCTGPDGVARNYTTKIHLICQRGSLSSSPRFLEQMG-CEFVFTWETEAACGIKV--ETQEDCTIPDPNSGYIFNLQPLTRGDRTEKKYYDVTSSD--GQAFKLNICGSVK-DGCPQYCDEDGHCEDASVCSIA-------TGKDKALAHARDAQLEFSDAGQLTLRYEGVR-DEGSGQVTS-VIIHFVCRTQVALGEPK--FLRQEGLS-------FLFEFATSLACPPAPVDCLVTDTAGNLYDLTPLSRTDQNWEPLNPESTPGHKDERYHINVCRPLVVTDNMDYSCPGGPLGSCMTG-----PQGNKNLGVVQSNPQAAGN-----SLSIRYYNGDMCR----DNVHYSTVIVFECSLTQG-TPTFQTTTQDGCEYAFLWRTPSACPIRRAV---GS-NCRVRDPLYGFEFDLSPLYNYTHDYRVRT---QQYEYNLNVCGPIHEGTGVCDAKV-HKDVSACQKTLPGVEPE--VYVTGTFNKTVVYEDGQILLNYTGGEK-CHT----VYNRSTAINFYCDHCADGKCKDGPKPGPVYVNETSD--CTYVFEWATSYAC----PPFKIVECVYHDDKGN-QFDLTPLSLSHGNWEIIPTSRSSNNEIFYINVCRSVVHSKG-VQCPYNAGACMKLPEAGDASSKFMNLGEVQAGP-WFEDGNLVLNYTLGANCTADGSRKKMTVIKFVCDEDS-VGNGPQFLGSTD-GCIYTFIWFTDVACPLKADQ-PSEGHCKAKNPLTGYEFDLSGLNKKDSYYQVLDQTKEHAYQINVCGAVKGSKCEGDNIGVCQAEL-IHGERTFNGGKSNSNLDYNE-GILELTYKQGDHCH-KDQFER--SSVISFVCANRTD---------NGRPVFID-ESEDCTYYFSFHTPLACETQVPCIVDN------------GTNSFDLSPLIKAEGHFMAPS---------IGGTGVYYINICRPLNPIPGVRCPPGASACMKED--NKSPVSLGRVFEQPK---INPVTKE---ITIIYKHGSKC--ESNPDYNNTAQIVFRCEQGTSVGSPQFSYKTDDCVYFFEWKTNVV-CAAPKPL-QTTECTYTDPSLKYTFNLTSLRSTGDNGYEVEDNVQGGKYSLNVCGTVPR----SGCDSDTAVCVTL-ADGKKISAGKFNTQTFKY---EGNLLTLSYSDGTTCRG----------------------------------------------------SEKYRTDILFKCADE-QNSQPAIFS----------------------------------MTGCSYTFMWKTSLACPPASQPCAINQN--GQTYDLNLLSQETNSWRFRDKFGNKYYLNLCQPVHQGPPR--CDPNAAACRQRPDGTVDNLGVVSSQTMNVDDTG------LKVVYSEGEAGV--CDDH-SRRSNSDSASIEIQFSCGSTV-GG-PQLHSID-SDGRCHFVVNWRSAIACATERQAVTPDDSGKIRDPFSQSVIPISQ--------LYNNNGNSWRVTGDLR----PNRDVYVYYIRMDD-------VGIQEDTCRG-AAVCQVKEKD-SFMKVLGKPN--MINYYMQDGALEVQILSDTKCGRRQEKKTSTTIIFHCSDYI---QKGQPEFFSESHDCEYIFNWYTNVACLKDDVEESVSIPPPEQDLPDDGTGIHHFIS------QHKSSAATVVGIMLAIVIICV--LVIIFHKQERRQAFVLKVKSCCRR-GDYSHPTYRYTKFMSNGDED---GTLLMGGLDSDEE--------SGNSQ----------------SEEASDDFMVE-----FDA------ESHQQNRAKRKKKTKPKADENHLEEVRXPFQKTKPKADE------------NHLEEVRPYHDDSDEDMLDV-----------Bflo_BRAFLDRAFT_216256/1-2276       ----------------------------------------------------------------------------------------------------------------------------------------------------------------------------------------------------------------MEL--------------FSDEDC--PGYPGHKIGTSINIQCGRTLGFPEFVEA-KPCTTYFDWDSTVACTSVQHPAQKEVKCYVYDTDGKKRDLTSLIKQSGGY-LV---DSAGERDFYINVCRDIT----PGGP----TSSCPPGTAGCRVIG-GEAVDMGRPTT-DLQMGEDGKLFLHYNGT-DGDAPSGCNGVKPTVHITFVCPNGDG-RGG--DPLLTLDTDCQYQVEWWTEFACPAEYLT-TTTCSFSKQQHDIDIDLSPLRVEEGRVVVLADYYCYK----------NA-YDYWLNVCGAVG--ISC-GDNDNDGTKTGVCQT-SADT--AHIAGKVLGHTLRYSDGELTLTYKGGEVCRHNGFNRTSVITFRCNKTAGNDGKGRPVFNSEEDCSYFFDWDTSYACL-QHEEGRSCRVSSPDGK-KRYDLSTLVQE----GPLNWEALDGRHEHGGGDNDERFFMNVCGEVITLEQ-AADCPQGAAACAISDT--GGGATNIGRYQTDPVLE--GNHIKLTYTDGDSC---HDSLTWSTVIRLVCSPGDLESAPVLLRRSDEKCLYEFEWHTAAAC----------------------------------------------PLGRKSGYDCRVFDDDAGFSFDLTPLSRAGTTADSF-YNVTNGKYDYLINVCGDINGTQSCEDRN---AKAGACQVDRSNN--NAKSLGVFNSHLNYYDGLINLTYTGGDPYHD----KVNRSTHIAFLCDPKAGPGQPRFLAE---DNHFYYFKWYTKYACPEQPIECVVTDPNTHTQYDLSSLAKSEDDNGENWSYLDDSDPN-NKKKYYINVCRPINPV------TGCDTYAAVCQTSFATGE----EAVTLPNMGMAESRPTIEGQAGHLLLTYSNGGPCTGPDGVARTYTTKIHLICQRGSLSSSPRFLEQMG-CEYVFTWETEAACGIKV--EKQEDCTIPDPNSGYIFNLQPLTRGDRTEKKYYDVTSSD--GQAFRLNICGSVK-DGCPQYCDEDGHCEDASVCSVA-------SGKDKALAHASDAQLEFSDAGQLTLRYDGVR-DEGSGMWRSYIIVHFVCRTQVALGEPK--FLRQEGLT-------FLFEFSTSLACPPAPVDCLVTDTAGNLYDLTPLSRTDHNWEPLNPESTPGHKDERYHINVCRPLVVTDNMDYNCPGGPLGSCMTG-----PQGNKNLGVVQSNPQAAGN-----SLSIRYYNGDMCR----DNVHYSTVIVFECSLTQG-TPTFQTTTQDGCEYAFLWRTPSACPIRRAI---GN-NCRVRDPLYGFEFDLSPLYNYTRDYRVRT---QQYEYSLNVCGPLHEGTGACDAKV-HKDVSACQKTLSGVEPENHLYLSGTFNKTVVYEDGQILLNYTGGEK-CHT----VYTRSTAINFYCDHCADGKCKDGPKPGPVYVNETSD--CTYVFEWATSYAC----PPFKIVECVYHDDKGN-QFDLTPLSLSHGNWEIIPTSRSSNNEIFYINVCRSVVHSKG-VQCPYNAGACMKLPESGDASTKFLNLGEVQGGP-WYEDGNLVLNYTLGANCTADGSRKKMTVIKFVCDTDS-VGNGPQFLGSTD-GCIYTFIWFTDVACPLKADQ-SSEGHCKAKNPLTGYEFDLSELNKKDSYYQVLDQTKEHAYQINVCGAVKGSKCKGDDI-----EL-IHGQRTFNGGKSNSNLDYNE-GILELTYKQGDHCH-KDQFER--STVISFVCANRTD---------NGRPVFID-ESEDCTYYFSFHTPLACETQVPCIVDN------------GTNSFDLSPLIKAEGHFMAPS---------IGGTGAYYINICRPLNPIPGVRCPPGASACMKED--NKNPVSLGRVFEQPK---INPVTKE---ITIIYKHGSRC--ESNPDYNNTAQIVFRCEQGTSVGSPQFSYKTDDCVYFFEWKTNVV-CAAPKPV-QTTQCTYTDPAIKYTFNLTSLRSTGDNGY--EDNVQGGKYSLNVCGTVPR----AGCDPDTAVCVTL-ADGKKISAGKFNTQTFKY---EGNLLTLSYSDGTTCRG----------------------------------------------------SEKYSTNIIFKCADE-ENSQPAIFSVSLCGYTASLQGDISFCLLITRWGCAWSLVWVNGMTGCSYTFMWKTSLACPPASQPCAINQN--GQTYDLNLLSQETNSWRFRDKFGNKYYLNLCQPVHQGPPR--CDPNAAACRQRPDGTVDNLGVVSSQTMNVDDTG------LKVVYSEGGAGV--CDDH-SRRSNSDTASIEVQFSCGSTV-GG-PQLHSID-SNGRCHFVVNWMSAIACATTRQPVTPED-GKIRDPFSQSIIHISQ--------LYNDNG----VTGDLR----PNRDVYVYYIRMDD-------VGIQEDQCRG-AAVCQVKEQD-KFMKILGKPD--MINYYMQDGALEVQILSDTRCGRREEKKTSTTIIFHCSEYI---ERGQPEFFSESHDCEYIFNWYTKVACLKDDVEE------------NDGTGIHHFIS------QHKSSAATVVGIMLAIVIICV--LVIIFHKQERRQAFVRKMKSCCGRGGDLSHPTYRYTKV---------------------------------------------------------------------------------------------------------------------------------------------------------------Bbel_LOC109481228/1-2460            M------------------AAAVHLWHR----------------------HD----------------------------------------PKTSMFWTISSLFFVFQLVSRTQGDCV-AGNYNLEPIQ--GLWEARGD--NNVQ--YNISICSQLTLPAP------CN-GNSVCRL--QSNVTSNIGNFNSS-LQIDEDRHGGGFRMEL--------------YSDDDC--PGHPGHKIGTSINIQCGRTLGFPEFVEA-KPCTTYFDWDSTVACTSVQRPAQKEVKCYVYGVDGKKRDLTPLIRQNGGY-LV---DSAGERDFYINVCRDITP--ETGAP----TSSCPPGTAGCRVIG-GEAVDMGRPTT-DLQMGEEGRLFLHYNGT-DGDAPAGCNGVKPTVHITFVCPNGDG-RGGSKDPLLTLDTDCQYQVEWWTEYACPAEYLT-TTTCSFSKQQHDIDIDLSPLRVQEG-----TNPWPYKVTT--TGDDGKT-YDYWLNVCGAVG--IQC-GDNDPDGSHTSVCQT-SADS--AHIAGAT-GHTLRYSDGELTLTYKHGEVCRHNGFNRTSVITFRCNKTADNNGRGRPVFNGEEDCSYFFDWDTSYACL-QHEEGRSCRVSSPDGK-KRYDLSTLVQE----GPLNWEALDGRHEHEGGDNDERFFMNVCGEVITLEQ-AADCPQGAAACAISDT--GGGTTNIGRYQTDPVLE--GNHIKLTYTDGDSC---HNSLTWSTVIRLVCSPGDLESAPVLLRRSDEKCLYEFEWHTAAAC----------------------------------------------PLGRKSGYDCRVFDDDAGFSFDLTPLSRAGTTADSF-YNVTNGKYDYLINVCGDINGTKSCEDRN---PKAGACQVDRGNN--NAKSLGIFNSHLNYYDGLINLTYSGGDLYHD----KVNRSTHIAFLCDPKAGPGQPQFLAE---DNHFYYFKWYTKYACPEQPIECVVTDPNTHTQYDLSSLAKSEDDNGENWSYLDDSDPN-NKKKYYINVCRPINPV------SGCDTYAAVCQTSFANGE----EAVTLPNMGMAESRPTIEGQAGHLLLTYSNGGPCTGPDGVARTYTTKIHLICQRGSLSSSPRFLEQMG-CEFVFTWETEAACGIKV--ETQEDCTIPDPNSGYIFNLQPLTRGDRTQKKYYDVTSSD--GQAFRLNICGSVKDDGCPQYCDGDGHCEDASVCSVS-------SGKNKALAHARDAQLEFSDAGQLTLRYYGVR-DEGSGQVTS-VMIHFVCRTQVALGEPK--FLRQEGLA-------FLFEFATSLACPPAPVDCLVTDTAGNLYDLTPLSRTDRNWEPLNPESTPGHKDERYHINVCRPLVVTDNMDYSCPGGPLGSCMTG-----PLGNKNLGVVQSNPQAAGN-----SLSIRYYNGDMCR----DNVHYSTVIVFECSLTQG-TPTFQTTTQDGCEYAFLWRTPSACPIRRAV---GN-NCRVRDPLYGFEFDLSPLYNYTHDYRVRT---QQYEYSLNVCGPLHEGTGMCDAKV-HKDVSSCQKTLPGVEPVV-AHVTGTFNKTVVYEDGQILLNYTGGEK-CHT----VYNRSTAINFYCDHCADGRCKDGPKPGPVYVNETSD--CTYVFEWATSYAC----PPFKIVECVYHDKEGN-QFDLTPLSLSHGNWEIIPTSRSSNNEIFYINVCRSVVHSRG-VQCPYNAGACMKLPEAGDAGEKFMNLGEVQSGP-WYEDGNLVLNYTLGENCTADGSRKKMTVIKFVCDADS-VGNGPQFLGSTD-GCIYTFIWFTDVACPLKAEQ-PSEGNCKAKNPLTGYEFDLNGLNKKDSYYQVLDQTKEHAYQINVCGAVKGSKCDGDDLGVCQAEL-IHGTRTFSGGKFNSNLDYNE-GSLELTYKHGARCH-KDQFER--SSVVSFVCANRTD---------NGRPVFID-ESEDCTYYFSFHTPLACETQVPCIVDN------------GTNSFDLSPLIKAEGHFMAPS---------IGGTGVYYINICRPLNPIPGVRCPPGASACMKED--DKNPVSLGRVFEQPK---INPVTKE---ITIIYKHGSRC--ESNPNYNNTAQIVFRCEQGTSVGSPQFSYKTDDCVYFFEWKTNVV-CAAPKPV-QTTECTYTDPALKYTFNLTSLRSTGDNGYEVEDNVQGGKYSLNVCGTVPR----AGCNKDSAVCVTL-ADGKKISAGKFNTQTFKY---EGNLLTLSYSDGTTCRG----------------------------------------------------SEKYSTNIIFKCAEE-QNSQPAIFS----------------------------------MTGCSYTFMWKTSLACPPASQPCAIQQN--GQTYDLNLLSQETNSWRFRDKFGNKYYLNLCQPVHQGPPR--CDPNAAACRQRNDGTVDNLGMVSSQTMNVDDTG------LQVMYSEGGAGV--CDDH-ARRSNSNAASIEIQFSCGSTV-GG-PQLHSID-SNGRCHFVVNWRSAIACATERESVTPDESGKIRDPFSQSVIPISQ--------LYNNNGNSWRVTGDLR----PNRDVYVYYIRMDD-------VGIQEDQCRG-AAVCQVKEKD-SFMKVLGKPN--MINYYMQDGALEVQILSGTKCGRKQEKKTSTTIIFHCSEYI---ERGQPEFFSESHDCEYIFNWYTSVACLKDDVEESVSIPPPEQNLPDDGTGIPQFIS------RHKSSAATVVGIMLAIVIICV--LVIIFHKQERRQAFVHKMKSCCGR-GDYSHPTYRYTKFMSNGDED---GTLLMGGLDSDEE--------SGNSQ----------------SEEASDDFMVE-----FDA------ESHQQNRAKRKK-------------------KTKPKSDE------------NHTEEVRPYHDDSDEDMLDV-----------Spur_SPU_019695_IGF2R/MPR/1-2390    M---------------------VDWFIR----------------------------------------------------------------CQ--------------------AGTCW-----------TLGTWTAQTTV-DGTASTFYLNLCENVASSPGSTG---CDTTVSSCLK--QGDAFTNVGNHNDT-LQLTINENTGGFRLDYRGSVC-------------------NVTEKYQTTIDFKCGTTLGSPNFLEN-SGCTYFFEWYSTVACSSVNGPALNEVPCSIYDSKGKERDLSPLIKLKGGY-LV---ESPDADDLYINVCRDITA--DTSGP----TAGCPAGSAGCLVKK-GLSVGMGTPHL-KLESLSDDELKLHYGGA---GVLDSCNGFVPSVTVVFMCPREQGRFAATRGPIQLSSTNCQYQIQWQTEYACEVDMLS-TDTCAFNSSTHGIDFDLSPLTKPDGEF--------YLIES------DPD-YYFYINICADMTAFANC--------EGMAVCQVARLNTVVGTSAGRNSNHELRYSDGELSLIYKGGDVCNHNKFQRTSVISFQCNKTK---DLGQPEFVTETECTYFFQWQTKYACMADAPSPTSCRL--ANGK-QRYDLSSLARK----TGSNWLVLDGRHSHS-ETTESEYFINVCSEVVQGDVASEGCPQGSSACVVDTVGTTKTGKSLGKYLTSPVWQ--NNLITMSYTEGDVC---KGTTKKRTDITFLCSPGDLESAPILVRKSDDGCQYDMEWHTAAACPLASKWGTDCRVFDDDAGDLESAPILVRKSDDGCQYDMEWHTAAACPLASKWGTDCRVFDDDAGVSFDLSPLKK----ADGH-YKVSVGEYDYYINVCSDIKLS-PCNDASHTLPHPAACQARKDAS-GVSYVLGQTNTSLGYFDGIIKLTYLNGKPYNSNGGTVTARMTEIAFLCDPEAGVGTPQFFQE---NNATYFFRWNTKYACPQPPIECVVTDEANSEQYDLSSLSKALEE--ENWSFVDERDAA-NRKKYYINVCRPINPH------AECGAFAGVCQTGFTTSDQQTSETALIPNLGVPKSPPTIES-SGHLLLTYQNGSDC----GESKQITSHIHFACNKGTLASTPRLLEVVDSCTYSFLWETEAACPIGV--STGENCTVRDSNSDYVFNLQPLIK-----QEGYQVFGPT--NEVFKLNVCGAVPVQSCPA---------GSGSCLSP----------TLSLG-SASSTLIFSDEGLLTLEYTGGTFNPQTAQYPS-TVISFLCRRNASQTSPSPVFVRQEGSR-------YIFNFETPLACLPESVDCLISDSNGNQYDLSPLAKDSGNWEAV--DTRQNYNGLSYHINVCRPINKGDPATSSCPGGPIGACQISES---DNSAYNLGYVQAMPEAATD----GALSLRYVNGDICH----AKFHRSTRINFECSDTPG-SPVFQTES-AECEYLFSWETPAACPLKRVT---GQGNCTVRVPEFGFQFNLSSLYNKTHDYTVTS---TEHEYTINVCGPLSTTMGECASS---DGVAACQKLQDGT-----FKNAGVFNKNLTYANGVLKLNYTTGSV-CHT----VYKRSTAINFYCDENVIGK------GEPHYIQETSD--CTYIFEWATPLAC----PPFKVVECSYRDGTS--QYDLAPLSKMTDNYRVNAAGNNLD----------------G-EFCPNNAAACLQTVDST-GKKSYISLGEVGQGP-TMENGHLVLRYESGATCPDKTGRKRTTVINIQCDKNS-VGTAPMLDHVDG-DCQYHFLWLSNYACDITEDIVKPKGDCTAMNPISGFRFDFNSLKKQAGYTV--MGSDSHKYVINVCDALASSVCDGSNIGSCQEEL-VVQGNHFNAGVFNKNLFFDD-SILFLRYDFGHACH-NNHFNR--SSVINFVCPHQKD--------TVGEPVFIA-ESDDCTYYFSWHTSLACEKFIQCSVVN------------GSDTISLAPLINDQGYHLATTL--------LGDGSLTYINLCRPLNPIPGLTCHPNSAACQVK---GGDPVNLGRVSEGPK---IG-ADGR---LSITYTQGDHC--SSDGTKNVSSVIKFFCKQGVTQGTPTLEF-IEDCIYQFTWGTNVV-CPATAPE-SDHDCTFFNTALQYRFDFTALSKEN--QAVVASS--AKTMKLKLCGLSSD--QSGEC-KGAAICLQAGQDKY--SLGQLSTQTISQ---EEEIFKVEYTGGSTCP--------------------------------------------------SSEGKKRQTTIILSCDPKPSKTSPTFFS---------------------------------SDENCHYSFHWATAAACPADTRPCDLAYQ--GNVYDLSPLSQITGSFQFQDSSKNMYYMNLCQPAYGTPED--CPPEASMCRQRPDGQVDVLGMVKTQQLTAKGDVP----DIVVTFFEGSQ-VDDCGSPDQPDSPSIPASSSITFKCANKM-GN-PVFQRTELVDNKCQFQFLWESKVACKEQRVPVTLVE-KKITDPETNVIMDFTE--------IIDHG--TWEARGDNRKEVTGDPSSYVYSINMNDNQ--DNVPGDVSHVCQD-SAVCQTKESDQQFFRDVGTRS--SRKFFIEDELVEMEVTKPKSCGKDNTEDVVTTVIFQCETDS---RNGNPEFYYESSNCQYYFLWLTSVVCTEGKIV----------IVPDDGGGNDTVGT------SNHKQASIIILILIIIIILCS--LAIVFHKKERRSAVYWRVRSCCPG-AYSKVPTYSYSQLTNGDPEESNDSRSLFAG--------------QEDEELLT-----------------------------------------------------------------------------------------ATRRNPFEFHDDSDDDILPH----------VSkow_LOC100366918_M6PR/1-2447       M-----------------MTGGVRGFYR------------------------------------------------------------------KLLSCMCLVLYFVQYSTVAVNAECK-IGGFDLETLNQW-TWEVRAADWNNIVSIYHINICKKFGTTESTAG---CQ-DSIVCEE--KDNVFTNIGNYTET-LANSIE-SSGGLRVDYKGIVCPIKVVTGGIYTGPEDE-PEDEIEYLQTTINFRCGKTLGSPEFVGK-FGCTYFFDWYTSAVCKSLSTEAKSEVPCYVYDANNKKRDLNPLIKTTGGY-LI---DTEGEESFFINVCRDINP--QTGGA----TQNCHESAAACLIKG-DTAYDMGVPSMGRLEIIDDNTLKLHYEAT-VTPALAECNGVKPKVIMRFVCPKDEGIRGGGKDPMMLSSINCQYEIEWFTEYACEEEYSV-SSTCQLTQDTHGIDIDLSLLQKPSE-----SEDK-YIAEG--KGWDDKM-YRYYLDVCKNTD--IEC--D-GDHARDPAVWQT-SEDSNWCTVAGSNKDRILRFSDGELTLIYRHGDQCSHNGLERSSFISFKCNAAS---GIGEPKFVTEEECSYFFEWETKHACR-DHARDSNCHVTSSDGK-QQFDLSTLVKT----EGSNWEALQHDES------DERYFLNVCGDILSSDPFTTNCPPGSAGCKIGT---DTGAQSLGKYSSSLIIE--GGNLKLTYSEGSPCVDTDSSIHMETTITFVCSPGDLESGPTLIRK--NKCLYEFEWHTAAAC----------------------------------------------PLGKRKGDHCKVVDDEAGFSFDLSNLKK-----EDN-YKVTYSMYDYYINVCKEVTGT-GATNCD---GTAGACQKQTTGD--NAWITGLPSSELEYYDGVIRLTYSNGDPYRTTNNGQINRQTQIAFLCKPDAGNGTPEFIGE---GNYTYSFRWYTKYACPQIPIECTVTDPDTHKQYDLSSLSKAEED--ENWSVID---PY-TIQKYYINVCRSINPV------AGCSTYAAACQTQINSQF---KEVIGIPNMGIASSRPTIES-SESILLEYTDGQDCTDVDNKIVKTAIRIHFICTPGNLESVPRFVEKIGNCQYSFMWNTEAACAIDD--TKGEECAVKDPNSEYTFNLQPLKR-----KTYYTVTTEEQPSQTFKINICDNVISSECSG---------SSAACLLD-------NGKWKSIA-SKSTSPEFSDDGQLTLKYEGVR-DVNSGQFVN-VVITFLCRTSIQLGEPK--FVRKEGIT-------YLFDFETALACRPQSVDCLVYDSHANQYDLSSLSRESGNWEAV--DTRVGYEHLRYHINVCRPLNSAPNGDYICPGGPIGGCQTSDN--DPKQNFNLGYIQAMPEAGID----GSLSIQYLNGDQCH----GKYHRSTRINFECSTIQG-SPVFQDET-PECEYVFTWETPSACKLQQNI---GS-ECKVKDPRYGIEFDLSPLRNEAHDYHVTV---GGYTYLVNVCGALKEGSGVCTGE-----VGSCQLLENGT-----PVDAGKYNQELKYDNGMLALNYTTGQK-CHS---DIYERATLLQFTCDEEVEGTNV----SSISFFKETDD--CTYVFSWRTRYAC----PPSKIVECSYRDQDGE-QYDLTPLSEMTHNYVVIPTIPGYKKEIYYINVCRSLKSEQD-INCPVNAAACLEY-ETDDGTKKWESLGTVSGEP-FMVNGQLQLHYELGDTCPGNPDKKKSAFITFSCQEDR-LESSPEFSYAVE-NCEYYFFWITKSACKLNHQE-VVTNDCTVENPESGLVFDLNVLKKDEGYKVL-NTEHGHLFDINICAPLKTSQCTGSNVGSCQSQVGINSGQAINAGIYNRKLFYND-GIISLNYSNGDPCP-DNKYHR--NSIFSFICNQDGDIG------NLGVPVYLD-DTDECTYYFSWHTPLVCENYIQCSVVKY---------GIEDLFIDLTPLITENGGYLAVSSLGDVPASDLKSSDTYYINLCRPLNPIAGAKCPPGSSACRVRE--GEAPISLGKAAEEPY---IT-AEDE---VAIKYVHGSKC--PQDEKKQLSMLITFHCKPGISETAPSVFG-YANCTYLVEWDTDVV-CPKSKPAPATSACTYYDSALDYTFDLSPLVV-------LDDD--GVEYDINICAPVPG--AKGSC-ENAGVCKHHGTNNYGVSLGSASHQEFMY---DSE-LKLMYSKGDGCPGVV-----------------------------------------------SEEDVLQTTTIIFECDETIGHGKPELFL---------------------------------LGGICEYVFKWKTSLACPPSSEPCVTAYN--GKIYDLQVLSRATGHWEVKDKD-NVYFINLCQPVSEAK----CPEGSAICVRKSDGSYESLGLAKTQHMK-----------------------------------GIVEITNIELQCSTTV-GKGPVIRSAD--SESCNFYFEWQSRVACSIEKEFVQMHD-EKVTDPVSNGVIDLSP--------LIDKG--LYTVDGDIR--LNGKLEQYVYLINLKKDG-LDSVEGDTDGKCKG-AAICQYKKDSNSMYRNIGMAS--PKSLTMEADILEIVVTNLGTCHKDNIDDVTSTIVFYCDNEADPSQLGVPEFQYESMNCQYFFNWHTPAVCVQAQLESSI------------SNRLSATVK------DNPGTVGIVILIVLLSITICI--LLIVFHKPERRSAFARKVRGCCCP-GYT-VPSYKYSKLADNDVESD-----LLMNIPEREY------------------------------DDDSEEEPANPVEVVASA------PEPKPKTKKDKKSKREKPDEK----DKKKKKKSKTKKDEPLLDMDVDIKNGPFKGKSIAYHDDSDEDMLNI-----------Cgig_CGI_10023625_M6PR/1-2378       MPY-------------------IY------------------------------------------------------------------------------------------TEKCL-VNNNDFSAVERFGPWEVISYD-GNNT--YQINLCGRVPTYSAGVGKPNCHNDTVVCMV--QDGQAFSLANYTNNTINAPSNDQEAEMWIVRNGDEC-----------------PDLAGENLNSIINLKCGKTLGFPKFLEH-TYCTSYFEWRTSYACRNMPV-SHHEVPCYVYNGKGQLIDLSALVKTQGGY-LV---DSAEGWEFYINVCRDITA--GTGDK----TSQCPPGSAACRVKN-DTSIDMGHTDK-KLQVDARGHPSLTYTSN---VTVPGCT-AKPKTTIQFVCPES----GGSEDPQLEFDFNCEYRVLWKTEHACPDVAVT-SSTCRLHNPQRNLDIDLSPLTNSPG-----VDKP-YEVYVNASGPGSRP-MRYYINVCGELG--IQC-PD-DESVRGVAVCQTMVGNSSWGHVLGKTDHQKLKYVDGLLTLTYKGGEKCNHNHFQRETIINFHCNHSAAYNGQGWPVFNREEDCSYLFDWDTKYACL-DHPVIEECRV--NHNG-KRFDLSPLVKH----TGKNWNVLEGGGQ----DGLSMYYINICSDLIHSDQ-AARCKSDSSVCLLDN----SGAHSLGRYLQNPTFDPRSNTIQITYTEGDSC--RDNTQKKSSVVTFICKPGDMDSGPVFLRRSLDECVYEFEWQTAAAC----------------------------------------------VLSRETGSGCKVYNEDLGINFDFNKLQA----AEGHYYNVTNGDYDYILNLCGPVKNT-LCDQKASQVTNPGVCQVKHGGTAADAFVTGQANTNLTYYDGLVKIKYEQGAMYNSN--PPTPRRTEITLICDRHAGRGSPVFVDEGLTSSGTYTFAWNTEYACPSSPID----------------LSRGTDE--ENWAVVDESNPG-SRKKFYLNVCTPLKEIPVG---SGCSPFAAVCQTSYQNSQ----EQLVFDNLGEVTSGPTVEG-EGLLTLRYVSVKNEC----QGKNWTSTIHFVCKKGALAKGPNPPQKIGDCEYSFVWETEAACPIADTNIEGKNCSIKDRNSDYTFDLSPLRKS--GDQDFYEVTAMG--GYKIRMNLCGSIKSPHCAA-IE----GAESAACLETA------AGLSQGLA-TLSHTLDYSEDGRLMMTFDGVR--QQNGQRTQ-VIVLFLCRQNIPLGAPE--FVRKEENA-------YYFDFKTSLACRPQPVDCQVQDEKGTQYDLSPLARSGTNWQVL--DTRPGFSDLTYYINVCRPINSVA--GSTCPGGPVGGCQVST----SGRAFNMGYIQSQPVVAGN----GTLTLRYIGGDHCHKGKPNEAPRSTRINFFCSPSEH-SPAFEAET-DTCEYIFNWLTPAACPVQRIV---GQ-NCVVSDPLYGYSFDFNKLRKADANYLVTS---GEYAYELNVCGPLVKSS-KCTDS----SIASCQTKPADS---NFHFDAGHSSSSLVYDSGEITLTYDNGHL-CHQ----KYNRSTVITFVCDQSKTGL------AGPTFLNETED--CVYQFVWPTAQAC----PPFKVADCGLRDPSGG-QYDLSSLSLNDDNYQTIDHVAK---KKYIFNVCRSLVHQKG-ETCPFNSAACIVDLGVTDPKKKFHNIGEVSEQQVQFLDGHLQLVYTNGEACKEDPTKHVTTRILLYCSKDS-IDTSPAGHFQV--GCEHRFVWQTAAACPIQTSK-GDGANCTVTNPSTGYAFDLSSLKRSEGYTV--DDRKDHKFTLNVCGAVAGTTCV-ASTGTCQVEK-KGEMRAFNAGKWNANLQYDD-GILFLNYSGGDKCH-NNQFER--NVIISFICSQGAG---------QGQPEFIA-ETGDCTYQFVWHTELACEEQVRCSVEK------------DGYTVDLSPLIKMSGHHLAVS----TAGHGTDTDGTFYINICRPLNPIYGKLCPPGASACQDRV--GKPPISLGKARTRPQ---LDPVSNK---IVLLYDHGSPC--PSNPAANITSKIVFNCKPGPLAGVPVLEY-VAGCQYIFEWDTNVV-CERNNTDTAKGKCVYQDPISGALYNFTGLRKAQ--PIKLYQN--ALQYLISLCEGLGA--EYPGC-EQASLCRWNGTLGH--GYGQVTTGEFIS---VEEQLKLAYKDGKSCNG------------------------------------------------------KSEGFMNFECDPNIYPGSPKIIF----------------------------------AKECSATFLWLTRAVCATVADQCTLAYN--GHVYDLSVLSRQQGSWNLTDAQRNTYWINICQGIHDGPDPQICSERSASCLRTTDGKIHNLGTVTSQTLSMEEDGK----TLRLEYTSNDL-A--CTGK-HRRSDNLRTRTIIHFECGNSV-GG-PVYIPRQPNSDECVFEFRWKSTVACHVERHSVQENK-GKIIDPESSSDTYKRKYDFIVVIYLFVEDVNSWVSSAHEFTLVTVPGESTEYQIDLAG----------TSTSCPPGTTICKVDKSQ-ATKTSLGLTT--GHQFYVEDDIVEVVFQTEQICDKDKQKKISSILQFHCNPW----GDSKPQFLFDSMDCTYMFTWENPHSCAHPVVVNPLPL-----PVDKAGTGNGSTGS------RSGSTVGTIVAVFLSAIVICL--LLIVFHKKERRDAVASRLRGLFRR-HSS---SYRYSEIPSSEVED------LLPEISASDL------GIQGSVQVTT----------------DTED---------------------------------------------------------------ISILRSEESPAVISYHDDSDEELLA------------Obim_Ocbimv22008285_IGF2R/MPR/1-2284 M-----------------------AWTR------------------------------------------------------------------FWLSLIVAVFIHGVYVLCDNEKGCK-IGDYDFSPLDLSYPWMSVSLS-NDT---YWISICSAVNHDK-------CPTGSSVCRE--RSGSFISVGNYTSS-PSMFEN-EDKEITVSFTGGVC-----------------PSNSSAHLRTDILFKCGKTLGSPNFLSQDNSCTEVFEWESLVACKKRPEKVVKEVPCYVY-VKGKKRDLTPLIKLKGAYKLVT--DSDGSPHFVTNICRAITE--DPADQ-----LGCPKGSASCGKIQ-KFLVDAGQPTT-ALRAIDERTLQLVYKSE---TSASSCV-NTPQTVITFICPAN----GRVSHPPLVSQSGCLFEIDWITQYACEESEISVKNSLKLIREKDNIDFDLTPLSKEN-----------YIVDV--TEKDGSK-YKISLAIGHT----IECFGSVDDDKYHPSVCQRKPNNKEFARALGNEQHSVLRYSDGHLTLTYKEGQPCSSN-FKRKTIIEFHCNKSAVN---SKPVYRYEEYCSYYLDWYTPYACI-DHPLQEKCSV--VDNG-KLFDLSSLRYT----SDNNWVALNGHEY----NDDAEILLNVCHDIMPSGK-AVKCPTDSAICLKKF---DGTTKSLGRYQHEPVYNDVTKTLQLNYTDGDKC----RSGKIFTVITFFCSPGISDNAPVVVTKSSDDCSYYLEWHTAAAC----------------------------------------------VVTKKKGADCQVENKI---------------------MKV--------------------------------------------SKNVGHANSILTYEDGILKLIYENGDKYNTD--PPIARRSVITFVCDTKASPGRPEFIKE---SNSTYWFLWYTKFACVSHPVECTFTDEQTHEQYDLSSLTKMS----DNWVTTGIDAPV---QKYYINVCRSLNHVPVG---TGCDVHSAVCSTKFEGGK----EVIAHENLGEVVKG-LVKK-GDNIVLEYTNGKACLDSGYTLTNYSTTIHFHCTKKITEHGPSLLSINSICHYVFLWNTPAACVQTSTESNQEKCSIKDTNSGYIYNLQPLIR-----DGSYSIVSQ---SHKFYLNICAPVNLPGCTM-SD----NSKASVCEQV-------SSTVTGLA-AVSTELELTDR-HLQLVYAGKR--LSDGSLFK-VTIIFVCAKEKELGSVS--LVRTTKSN-------YIFQFDTPLACPPQTVDCVVQDRLGNQYDLSQLTKQNGNWELP-------VGNMKYIINICSPINNYT--GNTTCAGNVGGCQIAP----GRGEYNMGYVQGKPIALSD----GTLSLRYRNGDLCHKGTDKQSHRSTRINFFCSTVEH-FISFGSET-EFCEYIFTWRTPAACPIKRIT---GK-FCKVVDPLYKNEFDLSVLR-KSTDYHVIG---GGYDFLLNVCGSLNTELENCNKN---TGSAACQTRSSDP---AFVINIGKASDELVYEDGIIFLTYKDGKSNCHK----KFSRKTIITFTCDPNVEGT------TGPHYIEEKED--CSYLFEWPTKHVC----PKITTSNCKIQDVKGN-EYDFSGLRLSSNNYIYLS--PEHEKQKFILNVCQTLVHRKG-ETCPSSSAACSINLNETDTTKKYHSIGELGSNPLELFGAYPQLVYKNGEPCR--EGKNSSTYITFQCDLSA-VDSSPKEYYYIESKCEHHFLWATREACPLSQKA-SHNNTCRVTDESTGVTFDLSQLKKSKGYTI--SDQKEHSFQLNICGAVANSQCS-KESSSCQTNT--YANRSFNCGNANSNLQFKE-GVIFLNYSGGDKCH-GGKFER--NTIINFVCNPSKG---------IGAPVYLA-ESKNCTYYFSWHTDLVCEKQVRCTVIK------------GDQMFDLSPLVQMSGHHVAEG-----LAPSEDPGASYYINICRPLNPIFGSFCPPLASVCETNV--GENGVSLGSVSEGPY---IDPNDQA---VTIHYKNGAPC---PNSKQNRSTIIKFRCKVGPSLGHPVLMDAIDSCTYIFNWDTNLV-CDKSSPD-AAKNCVYKDTRTLIEYDFSSLYNSA--VDKIPAD-PSGNFLIRVCGPVSG--VSSNC-EKSGACYVD-KNNHEVNYGKASDAVFSI---DQNLISLTYRNGDLCS--------------------------------------------------SESGTRASTKILFLCNNTAGLGKPVLYE----------------------------------KTPCQLVFLWKTRLACPPAVKDCFLSYL--NSSYDLSSLAN-TQSWKAMAITGETYWINVCQSLYTRPKSQNCSSNGAAVCVESKNEVMTIGSTSRIVLYVDRNSPVNNPVIILQYSSDTL-V--CSN--------KRATTIIRFSCSETL-GK-PVFSKKN--LETCTYEFNWDTYLACKEDRETLKEVN-GVLIDDKSSSRIYFGK--------QTQGK--TFRVE------ETKGTEKFYYDISFEGKL-YPDDENEGSDKCKN-AAVCQRKKGDSSYYKNLGSAT--KKRFYMDAAYMDVEITSPEKCHSNPSKNIVSVFEFFCTHLG---SQENPTFVYESNECVFLFYWHSSIACSYYNVSDP------------SQNKEVSVAS------SNYHIIVIAVSVTFSIIVIAV--LLKILMKPEQKQIMKENIKNIFTR-NRT--VKARYINVPQIDDGDD-EGLLVMED------------------------------------------------------------------------------------------------------------------EHNITYHDDSDEDMI-L-----------Dmel_lerp/1-917                     M--------------------------R--------------------------------------------------------------------------------------------------------------------------------------------CS-----------------------------------------------------------------------------------------------------------------------------------------------LQ-----------------------------------------------------------------------------------------------------------------------------------------------------------------------------------------------------------KDC-------------------------------------------------------------------------------------------------------------------------------------------------------------------------------------------------------------------------------------------------------------------------------------------------------------------------------------------------------------------------------------------------------------------------------------------------------------------------------------------------------------------------------------------------------------------------------------------------AAPTQEN---------------------------------------------------------------------------------------------------------------------------------------------------------------------------------------------------------------------------------------------------------------------------------------FRQMRFPIVI-----------------------------------------------------SLLLLLFCSGESVA------------------------------------------ADAANQLKFSTT-----ECKLKEPIYGSTFDFSGLH-SDLAHVVKSMNIGGDQFEFNICGNLSR---TCNGE---SNVAACLKRQGKE------YILGR-QHELFYNNGNMFLKYKSGAK-CDNGTADKPNYQLHVMFSCDYTLD--------AQPMHVTPYANEVCSFYISYRTPLACLSIPEGLQSNSCRVGDTKSNGTFDLMPLS--DSNYHT----SNRQGAFFVINVCKPVLYGEN-SMCPAGSSVCLFDSKATNPKERFINFGNVQTHP-VVEKGQLLLRHESPTPCAKNSSANYTSVIYFSCDKFI-RNAHPEFAGLGADSCTYQFNFATPLAC-------NDLKPCTAFT-STNELLDLSSLSSKPARTLL---KDGKNYTIAVCAH-AGAPCQ-ENGGACYE----QNSTTISLGNSNSQLRFNQTGSLYLLYEDGAECS-TATGMRRWSTKIEFVCANNATKDNGASTAGGSDSLKII-EDSNCQLLIQYQTPLACREPIRCKATTYVDHTNDGLGSSGDELIDLTPLISATDNYEARVELPASMEHLVPKTTKFFLNVCRPLVPKYQLGCAGGSAACMAKVTAAGAPEE-ERSMGFPL---VSLTQRNRTFAELAYLKGDPC--PTDNTSELSTHILFNCNMRAGRGQPVLRS-VEDCAYRFEWETNVF-CPPHECTFSADTCDLVHDELGRRFNFKSAPFTKDGKIEIDYN--ATKMSVNICGAHRK--AMTDY-SQALVNIFFTHESP--NCGREGTMNVQ--------------------------------------------------------------------------------IRLICSDQ-TESSSTISS----------------------------------DQQCNLLYVQRTPSICE---------------------------------------FLSL------GATQQNFESNGSTSSSTSSSSTSTTTSTTTQASPA---------------------------------------------------GK-P-------------------TKAATSTTTTTVGPDP-----------------------------------------------------------------------------AAT------------PIGPTA----------------------------------------------------------------------------------------------------------------SVGTILGAILS-VTFCVTCLGLLAFSPARRQ----RIRRLFRR-SNS---AVRYSRVQSNEEAN-----LLLEP----------------NGEFT-----------------------------------------------------------------------------------------------------ESDDDML------------L	b. Additional M6PR sequencesBL06085_cuf1_gene=M6PR/1-160                              ---------------------------------------------------------------------------------------------------------------------------------------------------------------------------------------------------------------------------------------------------------------------------------------------------------------------------------------------------------------------------------------------------------------------------------------------------------------------------------------------------------------------------------------------------------------------------------------------------------------------------------------------------------------------------------------------------------------------------------------------------------------------------------------------------------------------------------------------------------------------------------------------------------------------------------------------------------------------------------------------------------------------------------------------------------------------------------------------------------------------------------------------------------------------------------------------------------------------------------------------------------------------------------------------------------------------------------YKDQ-----------------------------------------------------------------------VATIAKD------------------------------------------------------------------------------------------------------------------------------------------------------------------------------------------------------EYLYSWNPCTPFTEGPAGDPDS--CTDMAACQISNDQ--QN--FFGLGTHDSAQFATDTDPDTGGTVVTVVYQTSG-------------SGRASEVLLKCTTGA--TVFT-VLGE----LQQ--GQYIFQLESPCCC-------------------------------------------------------------------PGAAAGCAD-------MGGGISGGTI-------------------------------FLIALISVIVV---------------------------------------------------------------------------------------------------------------------YFVG-GMIVMK---------------------------------------------------------------------------------------------------------------------------------------------------------------------------------------------------------------------------------------------------------------------------------------------------------------------------------------------------------------------------------------------------------------------------------------------------------------------------------------------------------------------------------------------------------------------------------------------------------------------------------------------------------------------------------------------------------------------------------------------------------------------------------------------------------------------------------------------------------------------------------------------------------------------------------------------------------------------------------------------------------------------------------------------------------------------------------------------------------------------------BL23560_evm0_gene=M6PR/1-272                              M----------------------------------------------------------------------------------------------------------------------------------------------------------------------------------------------------------------------------------------------------------------------------------------------------------------------------------------------------------------------------------------------------------------------------------------------------------------------------------------------------------------------------------------------------------------------------------------------------------------------------------------------------------------------------------------------------------------------------------------------------------------------------------------------------------------------QYVAALFVAVSL-------------------------------------------VARIAG---------------------------------------------------------------------------------------------------------------------------------------------------------------------------------------------------------------------------------ECVID-----------------------------------------------------------------------------------------------------------TAEELQDKEKKLLERLAPLT-----------------------------------------------------------------KKNELFTFKDD-----------------------------------------------------------------------------DPY----------------------------------------------------------------------------------------------------------------------------------------------------------------------------------------------------QYDYKARICSAVKSS------S--KPDAGIIQVDKMN--GT--EHSLGKINKVNIKAGTNW------MMLEYRGGDKYHSHCNG-----LERTSVIMILCDSSTVEGTMN-IIEE-HKKSNEGDCYYLFELYSNVVC-------------------------------------------------------------------SAKSS-----------IPGGLSVGSV-------------------------------LVLIFVVVVGC---------------------------------------------------------------------------------------------------------------------YFLF-GFLYQRYVVGAK--------------------------------------------------------------------------------GMEQI------------------------------------------------------------------------------------------------------------------------------PHFSF----------WKDFGNLQ----------------------------------------------------------------------------------------------------------------------------------------------------------------------------------------------------------------------------------ADGCELV------------------------------------------------------------------------CRTQEAG-------------------------------------------------------------------------------------------------------------------------------------------------------------------------------------------------------------------------------------------------------------------------------------------------------------------------------------------------------------------------PPRTYKGIGDDQLGLDD-----------------------------------------------------------------------------------------------------------------------------------DDERDDHLLPM-----------BL08860_evm0_gene=M6PR/1-226                              -------------------------------------------------------------------------------------------------------------------------------------------------------------------------------------------------------------------------------------------------------------------------------------------------------------------------------------------------------------------------------------------------------------------------------------------------------------------------------------------------------------------------------------------------------------------------------------------------------------------------------------------------------------------------------------------------------------------------------------------------------------------------------------------------------------------------------------------------------------------------------------------------------------------------------------------------------------------------------------------------------------------------------------------------------------------------------------------------------------------------------------------------------------------------------------------------------------------------------------------MDDGSGSIDLSALA-----------------------------------------------------------------GS-GTAAFNGE------------------------------------------------------------------------ATKWPDE-----------------------------------------------------------------------------------------------------------------------------------------------------------------------------------------------------NWQYSYNPCTPFDMQ------S--CLSVAACQVKSDGS-GE--SYDIGSQDTVLFSMEGND------VVITYNAFD-------------YQRHTIVKLVCSSS---TVFT-VDGE----DPEASTSFYFTLESPECCVKGSGPGPN-------------------------------------------------------PTGPNPGPGPT--------ITVSISVGSI-------------------------------MCIIFFPTVCI---------------------------------------------------------------------------------------------------------------------YIVA-GVLINKFARERE--------------------------------------------------------------------------------GRDVV------------------------------------------------------------------------------------------------------------------------------PNLAF----------WSTLPGLI----------------------------------------------------------------------------------------------------------------------------------------------------------------------------------------------------------------------------------KDGFLFTW-------------------------------------------------------------------ASMPCRKK-------------------------------------------------------------------------------------------------------------------------------------------------------------------------------------------------------------------------------------------------------------------------------------------------------------------------------------------------------------------------------GYTEI------------------------------------------------------------------------------------------------------------------------------------------------------------------BL09106_evm0_gene=M6PR/1-67                               MGPR----------------------------------------------------------------------------------------------------------------------------------------------------------------------------------------------------------------------------------------------------------------------------------------------------------------------------------------------------------------------------------------------------------------------------------------------------------------------------------------------------------------------------------------------------------------------------------------------------------------------------------------------------------------------------------------------------------------------------------------------------------------------------------------------------------------------------------------------------------------------------------------------------------------------------------------------------------------------------------------------------------------------------------------------------------------------------------------------------------------------------------------------------------------------------------------------------------------------------------------------------------------------------------------------------------------------------------------------------------------------------------------------------------------------------------------------------------------------------------------------------------------------------------------------------------------------------------------------------------------------------------------------------------------------------------------------------------------------------------------------------------------------------------------------------------------------------------------------------------------------------------------------LTVFVLV---------------------------------------------------------------------------------------------------------------------YVAG-GMLFLRFARGAE--------------------------------------------------------------------------------GTEMI------------------------------------------------------------------------------------------------------------------------------PNYEF----------WADFPHLV----------------------------------------------------------------------------------------------------------------------------------------------------------------------------------------------------------------------------------KDGFTFA---------------------------------------------------------------------TRPCRGEEA----------------------------------------------------------------------------------------------------------------------------------------------------------------------------------------------------------------------------------------------------------------------------------------------------------------------------------------------------------------------------YAYEKI------------------------------------------------------------------------------------------------------------------------------------------------------------------BL20088_cuf1_gene=M6PR/1-278                              MAA-------------------N------------------------------------------------------------------------------------------------------------------------------------------------------------------------------------------------------------------------------------------------------------------------------------------------------------TKE-----------------------------------------------------------NLR------------------------------------------------------------ISWFLYY-----------------------------------------------------------FLIFLT-------------------------------------------------------------------FQRTTVR----------------------------------------------------------------------------------------------------------GEM--------------------------------------------------------------------------------------------------------------------------------------------------------------------------------------------------------------------------------------------------------------------------------------------------------------------------------------------------------------------------------------QCVKT-----------------------------------------------------------------------------------------------------------GPCSCEMADGSGRIDLITLA-----------------------------------------------------------------EVGEPMVYDEQ---------------------------------------------------------------------------GRD------------------------------------------------------------------------------------------------------------------------------------------------------------------------------------------------------LNFYSFNPCLPFEEPNGID----VCANVAVCMYREVDEVDT--YYNCGEHLYSDFVYNNET---GY-IDLVYTGGYELG----------DVRHTIIHLVCGLIF---DLD-IQGY---QEET--RTVVMTMATPCACVTGCE------------------------------------------------------------------------------GFKDLSAGSV-------------------------------LLIIFTVSLGF---------------------------------------------------------------------------------------------------------------------YFAA-GAFYLHTVREKQ--------------------------------------------------------------------------------GCDMI------------------------------------------------------------------------------------------------------------------------------PHRHM----------WTELFSLI----------------------------------------------------------------------------------------------------------------------------------------------------------------------------------------------------------------------------------RDGCCFV---------------------------------------------------------------------LGPCRGNNPD---------------------------------------------------------------------------------------------------------------------------------------------------------------------------------------------------------------------------------------------------------------------------------------------------------------------------------------------------------------------------VMYSQY-------ERPS---------------------------------------------------------------------------------------------------------------------------------EYERPEYDGPV-----------BL39205_cuf0_gene=M6PR/1-124                              --------------------------------------------------------------------------------------------------------------------------------------------------------------------------------------------------------------------------------------------------------------------------------------------------------------------------------------------------------------------------------------------------------------------------------------------------------------------------------------------------------------------------------------------------------------------------------------------------------------------------------------------------------------------------------------------------------------------------------------------------------------------------------------------------------------------------------------------------------------------------------------------------------------------------------------------------------------------------------------------------------------------------------------------------------------------------------------------------------------------------------------------------------------------------------------------------------------------------------------------------------------------------------------------------------------------------------------------------------------------------------------------------------------------------------------------------------------------------------------------------------------------------------------------------------------------------------------------------------------------------------------------------------------------------------------------------------------------RSLDVVLECTPDS--DDLT-VRGE----IQT--NGYQFTLGSRHSC-------------------------------------------------------------------VKQGPWPG--------LAVSISFGSI-------------------------------LCIIFFPTLLL---------------------------------------------------------------------------------------------------------------------YCVG-GVAFNKFARGKG--------------------------------------------------------------------------------GRELV------------------------------------------------------------------------------------------------------------------------------PNVYF----------WSELPGVI----------------------------------------------------------------------------------------------------------------------------------------------------------------------------------------------------------------------------------KDGFRFA---------------------------------------------------------------------TSPCTSPQAR--------------------------------------------------------------------------------------------------------------------------------------------------------------------------------------------------------------------------------------------------------------------------------------------------------------------------------------------------------------------------DTAYDQI------------------------------------------------------------------------------------------------------------------------------------------------------------------BL14604_cuf0_gene=M6PR/1-229                              V---------------------------------------------------------------------------------------------------------------------------------------------------------------------------------------------------------------------------------------------------------------------------------------------------------------------------------------------------------------------------------------------------------------------------------------------------------------------------------------------------------------------------------------------------------------------------------------------------------------------------------------------------------------------------------------------------------------------------NSV--------------------------------------------------------------------------------------------------------------------------------------------------------------------------------------------------------------------------------------------------------------------------------------------------------------------------------------------------------------------------------------DCNTI-----------------------------------------------------------------------------------------------------------NSCSCSMADGSGVIDLSPLA-----------------------------------------------------------------STDGTPRYKGY-----------------------------------------------------------------------KATQPPD------------------------------------------------------------------------------------------------------------------------------------------------------------------------------------------------------QYVYDWNPCQPFTDG------D--CVSVAGCQSDPDG--GD--NYALGTQDSASFGSADDG----T-VLLAYSS---------------GVRTLLVTLTCTTTP--TTFT-VIGE----DQTVGSTYGFELRSPCAC-------------------------------------------------------------------PGATASCVN-------IGSGLSGGTV-------------------------------FIIIFIVTVSV---------------------------------------------------------------------------------------------------------------------YLIA-GVLYMSFVRNAT--------------------------------------------------------------------------------GIERI------------------------------------------------------------------------------------------------------------------------------PNIGF----------WRDLPALI----------------------------------------------------------------------------------------------------------------------------------------------------------------------------------------------------------------------------------KDGGKLL---------------------------------------------------------------------ISPCSKSSS------------------------------------------------------------------------------------------------------------------------------------------------------------------------------------------------------------------------------------------------------------------------------------------------------------------------------------------------------------------------------YSNI------------------------------------------------------------------------------------------------------------------------------------------------------------------BL02394_cuf1_gene=M6PR/1-260                              MAS----------------------------------------------------------------------------------------------------------------------------------------------------------------------------------------------------------------------------------------------------------------------------------------------------------------------------------------------ITMHRKL------------------------------------------------------------------CV----VVVIL---------------------------------------------------------------------------------------------VLYLVNV------------------------------------------------------------------------------------------------------------CI---------------------------------------------------------------------GQT--------------------------------------------------------------------------------------------------------------------------------------------------------------------------------------------------------------------------------------------------------------------------------------------------------------------------------------------------------------------------------------TCDTI-----------------------------------------------------------------------------------------------------------DSCSCTMSDGSGVVNLRPLV-------------------------------------------------------------------SGTPTYKDY-----------------------------------------------------------------------SATQVPD------------------------------------------------------------------------------------------------------------------------------------------------------------------------------------------------------DYLYSWSPCTPFSEAGSDDPAS--CTNVAVCQVSKDA--TV--SYGLGTQDSAAFTVTSDPVLGQT-LNLVYTMPQ-------------GGRVAGVTLQCSKGA--TTFS-VTGE----VTT--GAYAFELSSPCAC-------------------------------------------------------------------PGAGPGCA---------GGGLSGGSV-------------------------------FLIILFALAGV---------------------------------------------------------------------------------------------------------------------YVIA-GAIFMKFVKGAQ--------------------------------------------------------------------------------GSEVI------------------------------------------------------------------------------------------------------------------------------PNVAF----------WKSLPGYV----------------------------------------------------------------------------------------------------------------------------------------------------------------------------------------------------------------------------------KDGVFFI---------------------------------------------------------------------LSPCRGTKG------------------------------------------------------------------------------------------------------------------------------------------------------------------------------------------------------------------------------------------------------------------------------------------------------------------------------------------------------------------------------YDSV------------------------------------------------------------------------------------------------------------------------------------------------------------------Hsap_M6PR/1-277                                           M-----------------------------------------------------------------------------------------------------------------------------------------------------------------------------------------------------------------------------------------------------------------------FPF-------------------------------------------------------------------------------------------------------------------------------------------------------------------------------------------------------------------------------------------------------------------------------------------------------------------------------------------------------------------------------------------------------------------------------------------------------------------------------------------------YSC-----------------------------------------WRTG-LLLLLL-------------------------------------------AVAVRESW---------------QTEE-----------------------------------------------------------------------------------------------------------------------------------------------------------------------------------------------------------KTCDLV----------------------------------------------------------------------------------------------------------GEKGKESEKELALVKRLKPLF-----------------------------------------------------------------NKSFESTVGQG----------------------------------------------------------------------------SD------------------------------------------------------------------------------------------------------------------------------------------------------------------------------------------------------TYIYIFRVCREAGNH---------TSGAGLVQINKSN--GK--ETVVGRLNETHIFNGSNW------IMLIYKGGDEYDNHCGK-----EQRRAVVMISCNRHTLADNFN-PVSE--ERGKVQDCFYLFEMDSSLAC-------------------------------------------------------------------SPEIS--------------HLSVGSI-------------------------------LLVTFASLVAV---------------------------------------------------------------------------------------------------------------------YVVG-GFLYQRLVVGAK--------------------------------------------------------------------------------GMEQF------------------------------------------------------------------------------------------------------------------------------PHLAF----------WQDLGNLV----------------------------------------------------------------------------------------------------------------------------------------------------------------------------------------------------------------------------------ADGCDFV------------------------------------------------------------------------CRSKPRN-------------------------------------------------------------------------------------------------------------------------------------------------------------------------------------------------------------------------------------------------------------------------------------------------------------------------------------------------------------------------VPAAYRGVGDDQLG-EE-----------------------------------------------------------------------------------------------------------------------------------SEERDDHLLPM-----------Mmus_M6PR/1-278                                           M-----------------------------------------------------------------------------------------------------------------------------------------------------------------------------------------------------------------------------------------------------------------------FPF-------------------------------------------------------------------------------------------------------------------------------------------------------------------------------------------------------------------------------------------------------------------------------------------------------------------------------------------------------------------------------------------------------------------------------------------------------------------------------------------------SGC-----------------------------------------WRTELLLLLLL-------------------------------------------AVAVRESW---------------QIEE-----------------------------------------------------------------------------------------------------------------------------------------------------------------------------------------------------------KSCDLV----------------------------------------------------------------------------------------------------------GEKDKESKNEVALLERLRPLF-----------------------------------------------------------------NKSFESTVGQG----------------------------------------------------------------------------SD------------------------------------------------------------------------------------------------------------------------------------------------------------------------------------------------------TYSYIFRVCREASNH---------SSGAGLVQINKSN--DK--ETVVGRINETHIFNGSNW------IMLIYKGGDEYDNHCGK-----EQRRAVVMISCNRHTLAANFN-PVSE--ERGKVQDCFYLFEMDSSLAC-------------------------------------------------------------------SPEVS--------------HLSVGSI-------------------------------LLVIFASLVAV---------------------------------------------------------------------------------------------------------------------YIIG-GFLYQRLVVGAK--------------------------------------------------------------------------------GMEQF------------------------------------------------------------------------------------------------------------------------------PHLAF----------WQDLGNLV----------------------------------------------------------------------------------------------------------------------------------------------------------------------------------------------------------------------------------ADGCDFV------------------------------------------------------------------------CRSKPRN-------------------------------------------------------------------------------------------------------------------------------------------------------------------------------------------------------------------------------------------------------------------------------------------------------------------------------------------------------------------------VPAAYRGVGDDQLG-EE-----------------------------------------------------------------------------------------------------------------------------------SEERDDHLLPM-----------Ggal_M6PR/1-328                                           MGRA----------------------------------------------------------------------------------------------------------------------------------------------------------------------------------------------------------------------------------------------------------------RVVRQPWLLLW------------------------------------------------------------------------------------------PLGSA---------------PAR-RLR----------------------------------------------------------------------------------------------PVPEQPSF-------------------------------------------------------------------------------------------------LRRAAVR----------------------------------------------------------------------------------------------------------AERTVHSSRM-------------------------------------SSHC-----------------------------------------HTSAVLVVFMA-------------------------------------------LAAGVGAE---------------PLSE-----------------------------------------------------------------------------------------------------------------------------------------------------------------------------------------------------------KSCDVV----------------------------------------------------------------------------------------------------------GDESTESQMEKALLKKLEPLS-----------------------------------------------------------------QIRFNTTVEIG---------------------------------------------------------------------------TTE------------------------------------------------------------------------------------------------------------------------------------------------------------------------------------------------------NYAYHFRVCREVNSS--------LHDFAGLVQMDRQS--GK--TTVIGRINETQVFNGSDW------IMLIYKGGDSYGRHCSG-----EKRRAVIMISCKRGITASSFS-IISE--EREKEQDCFYLFEMDSSVAC-------------------------------------------------------------------PAEDS--------------HLSTGSI-------------------------------LLITFSALVTV---------------------------------------------------------------------------------------------------------------------YIVG-GFLYQRLIVGAK--------------------------------------------------------------------------------GMEQF------------------------------------------------------------------------------------------------------------------------------PHFAF----------WQDLGNLV----------------------------------------------------------------------------------------------------------------------------------------------------------------------------------------------------------------------------------ADGCDFV------------------------------------------------------------------------CRSKPRN-------------------------------------------------------------------------------------------------------------------------------------------------------------------------------------------------------------------------------------------------------------------------------------------------------------------------------------------------------------------------VPAAYRGVGDDQLG-DE-----------------------------------------------------------------------------------------------------------------------------------SEERDDHLLPM-----------Locu_M6PR_XP_006642487.1/1-268                            M--------------------------------------------------------------------------------------------------------------------------------------------------------------------------------------------------------------------------------------------------------------------------------------------------------------------------------------------------MCRGL-----------------------------------------RLA-------------------------------------------------------------------------------------------------------------------------------------------------------------------------------------------------------------------------------------------------------------------------------------------------------------------------------------------------------------------------------------------------------------------GWAS-----LSL-------------------------------------------LLALTGKG---------------QASN-----------------------------------------------------------------------------------------------------------------------------------------------------------------------------------------------------------MNCTLA-------------------------------------------------------------------------------------------------------------SGISDREQKVLNLLEPIS-----------------------------------------------------------------NQDFSTSSTDG------------------------------------------------------------------------------------------------------------------------------------------------------------------------------------------------------------------------------------------------------------------------------------TYTYYFRICGDAHGA----------GSAGVVQEDSKS--KK--ATVIGRYNSTQAFNGSDW------VMLIYGGGDNYTSHCGK-----EQRRAMVLISCNRRVSAGNFE-VMSE--ERDKARDCFYLFEMDSSYVC-------------------------------------------------------------------PET----------------HLSIGSI-------------------------------LLIVVFCFLCV---------------------------------------------------------------------------------------------------------------------YLVG-GFLYQRLVVGAK--------------------------------------------------------------------------------GMEQF------------------------------------------------------------------------------------------------------------------------------PNLSF----------WQGIGNLT----------------------------------------------------------------------------------------------------------------------------------------------------------------------------------------------------------------------------------ADGCDFV------------------------------------------------------------------------CRSRAHE-------------------------------------------------------------------------------------------------------------------------------------------------------------------------------------------------------------------------------------------------------------------------------------------------------------------------------------------------------------------------APPTYRGVPTEPLG-EE-----------------------------------------------------------------------------------------------------------------------------------PEERDDHLLPM-----------Cint_M6PRa_KH.L119.16.v1.A.SL1-1/1-288                    A----------------------------------------------------------------------------------------------------------------------------------------------------------------------------------------------------------------------------------------------------------------------------------------------------------------------------------------------------KHIGL---------------------------------------GLK-------------------------------------------------------------------------------------------------------------------------------------------------------------------------------------------------------------------------------------------------------------------------------------------------------------------------------------------------------------------------------------------------------------------KMLKPLLLSLFM-------------------------------------------VLHTQAE----------------QLAK-----------------------------------------------------------------------------------------------------------------------------------------------------------------------------------------------------------NDCTLL--------------------------------------------------------------------------------------------------------------KQNQQSQELLKRLDPLK-----------------------------------------------------------------GKIFTYKFKDE-------------------------------------------------------------------------INGGD------------------------------------------------------------------------------------------------------------------------------------------------------------------------------------------------------EYDYTLSICSGVTAN------QFNAPGVGVLQYNKLK--QE--YKRVGLINSTVVKGGTDW------IMLTYKHGDRYHSHCTSPGSELSRRQAHIMIVCDPNELEGQFK-VLYEHNDEDTESSCFYMMEISSRAAC-------------------------------------------------------------------SHVN---------------GLSTGSI-------------------------------LLIIFVSVTSA---------------------------------------------------------------------------------------------------------------------YLLI-GVVYKRCVYGSK--------------------------------------------------------------------------------GFEQI------------------------------------------------------------------------------------------------------------------------------PNIVF----------WRMCGNLQ----------------------------------------------------------------------------------------------------------------------------------------------------------------------------------------------------------------------------------ADGCDLL------------------------------------------------------------------------CRTRGSG------------------------------------------------------------------------------------------------------------------------------------YI-----------------------------------------------------------------------------------------------------------------------------------------------------------------------------------------------------------------------------------DSKPYQGVADDQLD-DEID-------------------------------------------------------------------------------------------------------------------------------------DEHLLPM-----------Cint_M6PRb_KH.S1537.1.v1.A.ND3-2/1-159                    ------------------------------------------------------------------------------------------------------------------------------------------------------------------------------------------------------------------------------------------------------------------------------------------------------------------------------------------------------------------------------------------------------------------------------------------------------------------------------------------------------------------------------------------------------------------------------------------------------------------------------------------------------------------------------------------------------------------------------------------------------------------------------------------------------------------------------------------------------------------------------------------------------------------------------------------------------------------------------------------------------------------------------------------------------------------------------------------------------------------------------------------------------------------------------------------------------------------------------------------------------------------------------------------------------------------------------------------------------------------------------------------------------------------------------------------------------------------------------------------------------------------------------------------------------------------------------------------------------------------------------------------------------ALQIAS----GD--KYDIGSQTTAVFVQVGDQ------IAINYTANSL-----LG-----DSRHSQVTLTCSKGK-QDNFI-VKGE----VTPNSGFYKFELVGNTLC-------------------------------------------------------------------PKKS-------------GGGLSAGSV-------------------------------LLIIFFVLLFV---------------------------------------------------------------------------------------------------------------------YLVG-GILYNRYKNEET--------------------------------------------------------------------------------GLDML------------------------------------------------------------------------------------------------------------------------------PNKEF----------WASLPGLI----------------------------------------------------------------------------------------------------------------------------------------------------------------------------------------------------------------------------------ADGIKFI---------------------------------------------------------------------FGGCKASPS-----------------------------------------------------------------------------------------------------------------------------------------------------------------------------------------------------------------------------------------------------------------------------------------------------------------------------------------------------------------------------SYDNI------------------------------------------------------------------------------------------------------------------------------------------------------------------Hsap_IGF2R/1-2491                                         MGAAA----------------------------------GRSPH-------------------------------------LGPAPARR---PQRSLLLLQLLLLVA-----APGSTQAQAAPFPELCS-------------YTWEAVDTK-NNVL--YKINICGSVDIVQ-------CGPSSAVCMHDLKTRTYHSVGDSVLR--------SATRSLLEFNTTVSC---------------DQQGTNHRVQSSIAFLCGKTLGTPEFVTA-TECVHYFEWRTTAACKKDIFKANKEVPCYVFDEELRKHDLNPLIKLSGAY-LVD--DSDPDTSLFINVCRDIDTLRDPGSQ----LRACPPGTAACLVRG-HQAFDVGQPRD-GLKLVRKDRLVLSYVRE-EAGKLDFCDGHSPAVTITFVCPSE---RREGTIPKLTAKSNCRYEIEWITEYACHRDYLE-SKTCSLSGEQQDVSIDLTPLAQSGG--------SSYI-------SDGKE-YLFYLNVCGETE-IQFC------NKKQAAVCQVKKSDTSQVKAAGRYHNQTLRYSDGDLTLIYFGGDECS-SGFQRMSVINFECNKTAGNDGKGTPVFTGEVDCTYFFTWDTEYACV-KEKEDLLCGA--TDGKKRYDLSALVRHAE--PEQNWEAVDGSQT---ETEKKHFFINICHRVLQEGK-ARGCPEDAAVCAVDK----NGSKNLGKFISSPMKE--KGNIQLSYSDGDDC---GHGKKIKTNITLVCKPGDLESAPVLRTSGEGGCFYEFEWHTAAACVLS----------------------------------------------KTEGENCTVFDSQAGFSFDLSPLTK----KNGA-YKVETKKYDFYINVCGPVSVS---PCQ------PDSGACQVAKSDE--KTWNLGLSNAKLSYYDGMIQLNYRGGTPYNNER--HTPRATLITFLCDRDAGVGFPEYQEE---DNSTYNFRWYTSYACPEEPLECVVTDPSTLEQYDLSSLAKSEGGLGGNWYAMDNSGEHVTWRKYYINVCRPLNPV-------PGCNRYASACQMKYEKDQGSFTEVVSISNLGMAKTGPVVED-SGSLLLEYVNGSACTTSDGRQTTYTTRIHLVCSRGRLNSHPIFSLNWE-CVVSFLWNTEAACPIQTTTDTDQACSIRDPNSGFVFNLNPLN-----SSQGYNVSGI---GKIFMFNVCGTM--PVCGTILGKPASGCEAETQTEELKNWKPARPV-GIEKSLQLSTEGFITLTYKGPL--SAKGTADA-FIVRFVCNDDVYSGPLK--FLHQDIDSGQGI-RNTYFEFETALACVPSPVDCQVTDLAGNEYDLTGLSTVRKPWTAV--DTSVDGRKRTFYLSVCNPLPYIP----GCQGSAVGSCLVS-----EGNSWNLGVVQMSPQAAAN----GSLSIMYVNGDKCG-----NQRFSTRITFECAQISG-SPAFQLQ--DGCEYVFIWRTVEACPVVRVE---GD-NCEVKDPRHGNLYDLKPL--GLNDTIVSA---G-EYTYYFRVCGKLSSD--VCPTSDKSKVVSSCQEKREPQ-GF--HKVAGLLTQKLTYENGL-------LKMNFTGGDT----CHK----VYQRSTAIFFYCDRGT---------QRPVFLKETSDCSYLFEWRTQYAC-----PPFDLTECSFKDGAGN-SFDLSSLSRYSDNWEAITGTGDP--EHYLINVCKSLAPQAGTEPCPPEAAACLLG------GSKPVNLGRVRDGP-QWRDGIIVLKYVDGDLCP-DGIRKKSTTIRFTCSESQ-VNSRPMFISAVE-DCEYTFAWPTATACPMKSNE---HDDCQVTNPSTGHLFDLSSLSGRAGFTAAYS--EKGLVYMSICG--ENENC-PPGVGACF------GQTRISVGKANKRLRYVD-QVLQLVYKDGSPCPSKSGLSY-KSVISFVCRPEARP--------TNRPMLISLDKQTCTLFFSWHTPLACEQATECSVRN------------GSSIVDLSPLIHRTGGYEAYDESED-DASD-----TNPDFYINICQPLNPMHGVPCPAGAAVCKVPI--DGPPIDIGRVAGPPI---LNPIANEIYLNFESSTPCL--ADKHFNYTSLIAFHCKRGVSMGTPKLLR-TSECDFVFEWET--PVV-CPDEV---RMDGCTLTDEQLLYSFNLSSLSTST---FKVTRDSRT--YSVGVCTFAVG-PEQGGC-KDGGVCLLSGTKGA--SFGRLQSMKLDYR-HQDEAVVLSYVNGDRCPPETDDGVPCVFPFIFNGKSYEECIIESRAK--LWCSTTADYDRDHEWGFCRHSNSYRTSSIIFKCDEDEDIGRPQVFS---------------------------------EVRGCDVTFEWKTKVVCPPKKLECKFVQK--HKTYDLRLLSSLTGSWSLVHNG-VSYYINLCQKIYKGPLG--CSERASICRRTTTGDVQVLGLVHTQKLGVIGD------KVVVTYSKGYP---CGG-------NKTASSVIELTCTKTV-GRPAFKRFD--IDSCTYYFSWDSRAACAVKPQEVQMVN-GTITNPINGKSFSLGDIYFK----------------LFRASGDMRT----NGDNYLYEIQL------SSITSSRNPACSG-ANICQVKPNDQHFSRKVGTSD--KTKYYLQDGDLDVVFASSSKCGKDKTKSVSSTIFFHCDPLV---EDGIPEFSHETADCQYLFSWYTSAVCPLGVG--------FDSENPGDDGQMHKGLSERSQAVGAVLSLLLVALTCCL--LALLLYKKERRETVISKLTTCCRR---SSNVSYKYSKVNKEEET-DENETEWLMEEIQLPPP--RQGKEGQENGHITTKSVK--ALSSLHGDDQDSEDEVLTIPEVKVHSGRGAGAESSHPVRNAQSNALQEREDDRVGLVRGEKARKGKSSSAQQKTV-----------SSTKLVSFHDDSDEDLLHI-----------Mmus_IGF2R/1-2483                                         M---------------------------------------RAVQ-------------------------------------LGPVPSGP---RVALLPPLLLLLLLA-----AAGSAQAQAVDLDALCS-------------YTWEAVDSK-NNAV--YKINVCGNVGISS-------CGPTSAICMCDLKTENCRSVGDSLLR--------SSARSLLEFNTTMGC---------------QPSDSQHRIQTSITFLCGKTLGTPEFVTA-TDCVHYFEWRTTAACKKDIFKADKEVPCYAFDDKLQKHDLNPLIKLNGGY-LVD--DSDPDTSLFINVCRDIDSLRDPSTQ----LRVCPAGTAACLLKG-NQAFDVGRPKE-GLKLLSKDRLVLTYVKE-EGEKPDFCNGHSPAVTVTFVCPSE---RREGTIPKLTAKSNCRYEVEWITEYACHRDYLQ-SESCSLSSEQHDITIDLSPLAQYGG--------SPYV-------SDGRE-YTFFINVCGDTK-VSLC------NNKEAAVCQEKKADSTQVKIAGRHQNQTLRYSDGDLTLIYSGGDECS-SGFQRMSVINFECNKTAGKDGRGEPVFTGEVDCTYFFTWDTKYACI-KEKEDLLCGA--INGKKRYDLSVLARHSE--SEQNWEAVDGSQA---ESE-KYFFINVCHRVLQEGK-ARNCPEDAAVCAVDK----NGSKNLGKFVSSPTKE--KGHIQLSYTDGDDC---GSDKKISTNITLVCKPGDLESAPVLRAARSDGCFYEFEWHTAAACVLS----------------------------------------------KTEGENCTVLDAQAGFSFDLSLLTK----KNGA-YKVETEKYDFYINVCGPVSMD---PCQ------SNSGACQVAKSG---KSWNLGLSSTKLTYYDGMIQLSYRNGTPYNNEK--HTPRATLITFLCDRDAGVGFPEYQEE---DNSTYNFRWYTSYACPEEPLECMVTDPSMMEQYDLSSLVKSEGGSGGNWYAMENSREHVTRRKYYLNVCRPLNPV-------PGCDRYASACQMKYENHEGSLAETVSISNLGVAKIGPVVEE-SGSLLLEYVNGSACTTSDGQLTTYSTRIHLVCGRGFMNSHPIFTFNWE-CVVSFLWNTEAACPIQTITETDQACSIRDPSSGFVFNLSPLND----SAQGHVVLGI---GKTFVFNICGAM--PACGTVAGKPAYGCEAETQIEDIKDLRPQRPV-GMERSLQLSAEGFLTLTYKGS---SPSDRGTA-FIIRFICNDDIYPGAPK--FLHQDIDSTRGI-RNTYFEFETALACTPSLVDCQVTDPAGNEYDLSALSMVRKPWTAV--DTSAYGKRRHFYLSVCNPLPYIP----GCHGIALGSCMVS-----EDNSFNLGVVQISPQATGN----GSLSILYVNGDRCG-----DQRFSTRIVFECAQTSG-SPMFQFV--NNCEYVFVWRTVEACPVIREE---GD-NCQVKDPRHGNLYDLKPL--GLNDTIVSV---G-EYTYYLRVCGKLSSD--VCSAHDGSKAVSSCQEKKGPQ-GF--QKVAGLLSQKLTFENGL-------LKMNYTGGDT----CHK----VYQRSTTIYFYCDRTT---------QKPVFLKETSDCSYMFEWRTQYAC-----PPFNVTECSVQDAAGN-SIDLSSLSRYSDNWEAVTRTGAT--EHYLINVCKSLSPHAGTEPCPPEAAVCLLN------GSKPVNLGKVRDGP-QWTDGVTVLQYVDGDLCP-DKIRRRSTIIRFTCSDNQ-VNSRPLFISAVQ-DCEYTFSWPTPSACPVKSNT---HDDCQVTNPSTGHLFDLSSLSGRAGINASYS--EKGLVFMSICE--ENENC-GPGVGACF------GQTRISVGKASKRLSYKD-QVLQLVYENGSPCPSLSDLRY-KSVISFVCRPEAGP--------TNRPMLISLDKQTCTLFFSWHTPLACEQATECTVRN------------GSSIIDLSPLIHRTGGYEAYDESED-DTSD-----TTPDFYINICQPLNPMHGVPCPAGASVCKVPV--DGPPIDIGRVTGPPI---FNPVANEVYLNFESSTHCL--ADRYMNYTSLITFHCKRGVSMGTPKLIR-TNDCDFVFEWET--PIV-CPDEV---KTQGCAVTDEQLLYSFNLTSLSTST---FKVTRDART--YSIGVCTAAAG-LGQEGC-KDGGVCLLSGNKGA--SFGRLASMQLDYR-HQDEAVILSYVNGDPCPPETDDGEPCVFPFIYKGKSYDECVLEGRAK--LWCSKTANYDRDHEWGFCRQTNSYRMSAIIFTCDESEDIGRPQVFS---------------------------------EDRGCEVTFEWKTKVVCPPKKMECKFVQK--HKTYDLRLLSSLTGSWDFVHEG-NSYFINLCQRVYKGPLD--CSERASICKKSATGQVQVLGLVHTQKLEVIDE------TVIVTYSKGYP---CGG-------NKTASSVIELTCAKTV-GRPAFKRFD--SVSCTYYFYWYSRAACAVRPQEVTMVN-GTLTNPVTGKSFSLGEIYFK----------------LFSASGDMRT----NGDNYLYEIQL------SSITSSSYPACAG-ANICQVKPNDQHFSRKVGTSD--MTKYYVQDGDLDVVFTSSSKCGKDKTKSVSSTIFFHCDPLV---KDGIPEFSHETADCQYLFSWYTSAVCPLGVD--------FEDESAGPE---YKGLSERSQAVGAVLSLLLVALTGCL--LALLLHKKERRETVINKLTSCCRR---SSGVSYKYSKVSKEEET-DENETEWLMEEIQVPAP--RLGKDGQENGHITTKAVKAEALSSLHGDDQDSEDEVLTVPEVKVHSGRGAEVESSQPLRNPQRKVLKEREGERLGLVRGEKARKGKFRPGQRKPT-----------APAKLVSFHDDSDEDLLHI-----------Ggal_IGF2R/1-2561                                         MPPPLPTTVKRNHRPQHVPSGAVRGAGRKRSHTPRPPPGGRARHVIAMPAHDGGHMTRQWAGGGHMTCRWRPALLDVPGQPLRPGPARAAAMARAAFAPLLLVLLCLPLGDGVAAPVSPDEPFFQDLCS-------------YTWEAIDTD-KHVL--YKINLCFGVEE---------CGRSSAVCAYDVDKRAYMSVGSLTLR--------EISKTLLVFNTTSKC---------------SQQGSEHRIQSNINFLCGKTLGTPEFVTA-TECVHYFEWRTFVACKKNLFKPVKEVPCYVFDEDLKKHDLSPLIRVPGHY-LVD--DSDND-SLFINICRDIGR---SSGE----TMNCPAGSAACLIHE-GHAYDVGRPQD-QLKRHDKDRLILSYERTYNDEKLNFCLGHNPAVTITFVCPSKRG--EESAGPKLTAKTNCRYEVEWVTEYACHRDYLE-SKSCVLTNEQHDVSIDLSPLTLSPD------YVTPYLAK-----DDKEE-YYYYLNVCGRTG-AGNC----EGSMTYTSACQVKS-STNQKKVTGRFENQTLRYSDGDLTLIYPNGDACS-SGFQRMTVINFECNETAGNDGRGTPVFTGEVDCTYFFTWYTKYACV-KEREDLLCRV--ADKKKHYDLSPLIRSSE--SAQNWEAVDSNLS---EEWRKRYYINVCHKVLKRGG-ASGCPDAAAICSVDK---KNNSKNLGTFVSPPKKV--GENIQLTYSNGGSC---GGNKKIKTVITLICRPGDLESAPILISE--GVCSFTFDWYTAAACVLS----------------------------------------------KMEGDNCQVSDPQAGFSFDLSPLTK----KNGH-YTVNTEEYLFYINVCGSVPNE---LCH------TQSAACQVTQRKD--QFWSLGLPNSRLSYYDGLIQLTYKNGTAYNNEK--KTQRSTLITFLCDRQAGIGQPEYQVE---DNYTYNFRWYTEYACPEMPLECIVTDPNTMDQYDLSSLAKSE-KRGENWYAMDNSGPN-ERKKYYINVCRPLLAV-------PGCDRRASVCQMEYRHDHDSYYEVTSISNLGVASKELVVER-LGHILLTYANGSVCINADGERTSYTTTIHFVCSRGTLNSSPRFISIQE-CVVTFLWETEAACPIKETKDDSQSCSVRDPNSGFLFNLQPLA-----AEKGYTTTGI---GKTYLLNICEAM--PECGKINGKPAAGCEAD-------NLTSVRMV-ELDKTLYLSSEGFLTLTYRGPL-LVESGKSDT-FTVTFICNDS-YPGELK--FVREEINSMLNI-HDTFFEFHTALACAPAPVDCQITDAAGNEYDLSDLSKEGKPWVAI--DTSKDAKKRTFFLNVCKPLPFVP----GCPGGAIGSCVKY-----ADKSKNLGVIQINPQAATD----GSLSIIYLNGDMCK----DKRRYSTRIIFQCDQTMG-SPVLEQE--DNCEFVFVWRTLAACPVHKAE---GE-DCQVKDPRYGHVYNLKPL--SSKDIKVST---D-EYDYYFRVCGEITEH---CRPG--AHSVSSCQVKKTDS-TF--RKVAGLLTEKLTFKNGL-------IMINYTSGEK----CHK----IYERSTAILFYCDKTT---------SEPVFLKETPDCTYMFEWHTQYAC-----PPVKSTECSYRDDEGN-FYDFSSLTRHRENWEATDISTST--KIYYINVCKPLVPYGAAHSCPPDAAASLVE------GIKCVSLGEVAEGP-RWENGISTLKYINGELCP-DKIRRKTTILRLKCDESK-IESKPELIMAIE-DCEYSFLWFTAAACPLKSNV---QNDCRVTNPATGHLFDLTSLKRESGYTITDS--HNRKIELNVCAE-AKSSC-ANGAAVCITD----GPKTLNAGKLSKTLTYED-QVLKLVYEDGDPCPTDLKMKH-KSYFSFVCKSDAGD--------DSQPVFLSFDEQTCTSYFSWHTSLACEEEVSCSVLN------------GSSVIDLSPLIHRTGYYEAFVDG---DQSD-----VSPDFYINICEPLNPIKDVNCPPGAAVCMVPV--NESPIDIGRVTEPPK---LNEAVNEVYITYNSTTPCQ--INNKLNYTSLIVFHCSQGTSLGKPKMIQ-KLDCSFVFEWET--PVV-CPDRV---KTLGCSVTDEQLHYTFNLTSLSGRS---FEVLSGSSR--YHVSVCSKAAADVSQGKC-KDGAVCMTSGSGVS--SFGSIKEMKMNYS-RQDETVILQYTGGDRCPPVTEKGELCVFPFKYKGKSYDKCITEEKNR--PWCATAVDYQGDGKWGFCVNATARRASTIIFVCDENAGSGSPRLLS---------------------------------DMLGCAVTFEWRTQAVCPPKKMECKFVQK--HRTYDLRILSSLTGSWVFYHNG-NSYYLNLCQRVYEGPTG--CPERASICRKSNNGDVEVLGLVHTQKLNVTGD------TVYISYSGGQE---CGK-------NKKIMTIIELRCAKTV-GMPMLQRFD--EENCAYFIIWDTRAACAVKPQEVEVVN-GTVINPATGKNFSLGDVYNK----------------LYMASGDIRT----NGDQYVYEIQL------SGITNSSFPECSE-ANICQVKTSE-RRFRKIGWAK--KAKYYVEDDDLDVIFSSDSRCGKDKSKFVSSSIFFHCSPHV---QEGIPEFLHETADCQYLFTWYTSAVCPLISTIAPG----IHGGQSDQEAQVYKGLSRRSQAVGAVLSVLLVVLTACL--IILLFYKKERRETVVNKITNCCRR---TSGVSYKYTKINSEEEA-NENETEWLMEEIAAPNQ--RTVKGVQENGHVTTKSVTSDAFTSLHVDDLDSEDEVLTIPDVKIQTGRGLD-KSKHPQKKPPRF----ASDDKAYLLNGGKERKAKAKPGQQQAQ-----------NSTNSVSFHDDSDEDLLNV-----------Locu_IGF2R/1-2496                                         MGPPF----------EHIPT---------------------------------------------ITC------------------------HVVVVRGIITVLTLL-----SSIAISDESLWYQELCR-------------FKWEAVDPD-GNVH--YDIKLCSSSPSTT-------CGETCAICAKNLTTKQSLSVGEVSML--------TASPNVMTFNTTQKC---------------SDPNAKQNFQSSISFLCGKTMGTPEFVTV-SECVYYFEWRTYVACKKDKFKPHKEVPCYVFDADGKKHDLNPLIKITDGY-LVD--DSDTDVDLYINICRSITY----------SGNYCPEGSSACLITSKGEYLDMGHPTE-SLELLDKDRLMLRYIGV-TEGKPEFCGDHTPAVTITFVCPSG---RQEGSDPRLTAKENCRYEVEWVTEYACHRDYLE-TSDCKFSSAEHDISIDLTPLHIDAN------SETPYRATST--SSDGKDTYFYYLNVCGETS-AGEC----RDTKGSISSCQVKQ-DGSLTKVAGRYKNQILRYSDGDLTLIYPGGNSCS-SGFQRMTIINFECNQTAGNNGKGSPVFVGESDCTYFFNWQTSHACV-KEKENLLCRV--TDKKKRYDLSALTRYSKPGSNQNWEAVDSTSS---KSEKKHFYINICHRILQNEA-TKGCPEEPAVCAVGQ---DGKARNLGKFVSSPIKD--NDNIRLTYTEGDTC---KKDTKIKTVITLLCKPGDLESAPLLRSVSSDECVYEFEWHTAAACVMS----------------------------------------------MTVGDNCIVSNPQAGFSFDLSPLRN----ADG--YSVSSGDYKYFLNVCGSVKEG---ICT------ANAGACQVDKSG---SSWNLGDFNSKLSYYDGLIHLNYRNGSKYNNRL--HTQRSTFISFLCDREAGVGLPEYQVE---DNYTYNFKWYTSYACPEMPIECMVTDLKTMQQYDLSRLSKSE-GSDENWQAMDSSQPG-NRRKYYINVCRPLNHI-------PGCDRYASVCEMKYEVQEGIMVEKVSVSNLGIAKKGPMIED-ENHLLLEYSDGSACVNEDGNRMAYTTRIHLVCSRGILSSGPRFLMNQN-CTATFLWETEAACVISTSE-ESNTCTLKDPSTGFEFNLQPLA-----SKTGYEALGN---GKKFKVNICDSL--DECGT--DKPAAGCEIE-------DGKPLGPV-GVTKSLQFSTEGLLTLTYKGDL-DVATGISKT-YIINFICDQDIYPGTLN--LVTEEMSSTTHVTHDVFFEVHTALACAPAPVNCEVTDSFGNEYDLSDLSRDDAPWQAI--DTSADAKQRTFYINICKPLPLVR----GCPGGPLGSCASF-----PDHSFNLGYIQASPQAAPD----GSLSIVYLNGDKCG-----QSRYSTRIIFQCDDSPG-SPVFDRL--NGCEYVFIWRTSEACPIHRAQ---GD-HCRVRDPRSGYVFDFTKL--SGKDYEVKS---G-QYNYRFSVCSPLHTK--KCKNS---ESASSCQEDTAQQ-DF--PRIAGLATQNLTYEDGL-------IMINYTNGEK----CHK----IYERSTAIVFICDHSKN-------YGTPVFLKETLDCTYMFEWHTELAC-----LPFKTIECSFKDGNGN-SYDLSPLSLHNTNWVVEPMIGSTQ-KRYYINVCKSLVQQNGSWECPSSAASCMKN------GSQYVSLGEVESGL-QWENNVLFLQYINGEKCP-DGKRNRMTIIRFKCDEDK-VDSKPTLITAIE-DCVYTFMWFTATACPLKSNV---HKDCKVTNPVTGYLFDLNVLNIKGGYTVYDSTNKKKLIRLNVCGGVADSGC-GPDAGVCIKD----TKTAVNAGLVSKTLTYLD-QVVQLTYENGDLCPGSSSLRH-KSIFSFVCKSQASS--------ADGPVLVATDKEKCTHFFSWHTPLICEQQVSCSVKN------------GSSIIDLTPLIHKTGYYSATDAE---LGRD-----NSPDFYINICQPLNPIPGVTCPPGAAVCLDPV--DGLPIDIGRITGPPQ---INEAINEVYISFNSNTVCL--SDKSKNYTSLILFSCQRGTDLGSPQMIR-KSPCSYVFEWAT--PVV-CSDSV---STSGCALKDDQLQFTFSLSSLTGGS---YQVPSGSKS--YQINVCAPVTD----AKC-QDSAVCLVLNNAAY--SFGNFKAMSMDYR-HEDQAIIMKYGSGDPCPTVTTKGEVCTFPFKYHKKSYNSCTTDGRTNGDLWCATTDDYNRDQKWGFCTNATGKMSSTIIFKCDRSADPGSPQLLS---------------------------------ETLGCATTFEWKTSVACPPKKMQCKVVSN--HKTYDLHALSSLTAPWKFSHGS-DSYYINFCQEIHGGLTD--CPVTAAVCRQTKTKKTQTLGLFYTQQISVKDE------KIYINYSRGDE--VCEN-------GIQAKTIVQLECGKTM-GIPTFQSLD--EKNCEFWLHWETRAACVMTQQEVQMIN-GTITIPDTGASFSLGAIYFR----------------MHTAYGDIRS----NGDKYIYNIQL------SGITD-TSSGCLG-ANICQVKVSG-EYKRKIGHSS--SAKYYVKDGNLDVVIPSDSLCGRDKSKNVSSTIIFQCNPSA---GEGIPEFLLEADDCQYLFHWHTSVVCDLVAV--------MDSGKNDKSDSGLIGLSGRSQAVGAVLSVLLVVLTACL--LILLLYKRERRELVLQKVTGCCRR---GGNISYKYSKISTEEDG-AEDETEWLMEEVATPDSLSRVAKECQENGHITTKPVNADAFNAFPLDEQDSEDEVLTVPGVRIQSSQ-PSLEWQNGKASPKRPLLEQESDDDLVEFLGEKKTRPKCK-GKTQRK-----------RAENLTSFHDDSDEDLLKSHRCYDTTRQAACint_LOC100186527_IGF2R/1-2335                            M-------------------------------------------------------------------------------------------IIKYTLYFAAILFIA-----SPTYAE-----VFQSCENVHGKNLSQLT--GNWTTYRDG-GDTVQHYLIDICQTVNQAG-------CDGSS-ICWVQ-NNGVGKSLGDAAMPPNVMYQSASSVSFYLEYNTTKLCQ--------------SNSNGTFFYRSSITFECGKTLGSPVVISS-DICSVDFIWETSLACRTSEYDAINEVRCYAYDSNHHKRDLESLIKRVGGHKVTDPTSSISD-KIYINVCRDIRS--VGGDSEFSNVGQCPEGSAACLVKE-GVGYALGFVGS-PLSIGENNRMVLTYHGE-ASNSPPECLGNIPQVKINFVCPGAD--RGGSRPPIITSSTNCQYSIEWWTEAACITDFVT-STTCGLDLARHGVDLDLSPLTLHGN---------HYTVNI----HDTAD-TVFYINVCEATG--TSCGA--KRDKTKNTVCQTTS-SNTLGYIGGELDGYRVKYFDGQLTLTYKDGDRCHGNGFRRTSVILFQCNKTA---GVGSPIFSHELHCTYYFEWQTSHACI---AADPSCHLV-DEKGQNYDLSPLTRTLD-SHNTNWEVLDGSET--ASNNRRRYFVNVCGEVIRDAD-TVNCPPSSSVCLVQG---ANTPLSFGSFSRSKITA-KSNTIMLNYT-GGVC---ASGGMKTALITMYCRLGDLESSPVLKYRSSDDCVVSMTWHTAAACPLK----------------------------------------------TVTGTGCMVSDEDSGLTFDLRRLTQ----SEHM-YKIPVGDYDYYLNVCGELKNT---PCNNQN---NHPAVCQSKRSGQ-MRSFTTGQVSDKLTYYDGFIKLVYTDGDSYSNK---LHNRSTQISFLCDNSRGVGHPEFVNE---GNFTYLFNWYTSYVCPAASVQCMATDPNTGDQYDLSSLAKSEDNEDANWSAMGGEDTVKGKTKYYLNVCRPVNEVQ-GTSAMDSCDALAAACSTTVDANG---QEKVGISNLGKPTSAPIIES-KGHLSITYTNGEACVE-NGVSKNFTTTIHFVCVKGAISSTPSFIEKSA-CSVSFMWNTETACPITSIT--QGSCEVVDPNSRLTYNLRPLMR---KADNPYRVDGVH--GYQYYINICGSV--SSCQA-TTSPIGACRVESY-----TNAIDQKA-TNTSSLSYSDDGQLSLTYQSSQ-RNQDGSFLT-YVVNFICNHTVAVGEPK--LTDVDG-------HIIRLDFQTSLACQPDTVDCIVADDMGNQYDLTPLSNPNHPWDVV--DTQV---SRRFYVSVCRPLPAVS----GCPGGGTGACFIQHATDNTIQGHSLGLIQSNPTAVTQNGTASTVSIRYMGGDVCE--ADHTMRYSTRILFICSTTKGRSPVLQPNSDTPCDFVFVWETPFACPIKKSITKKGH-PCSVVEPLYQMTFDLSTMT-STTDYTVSG---GTEWDFKLNICSKLNNGIGACEQS------GACQVSKTKA-NK--VHTAGNPNTTLIYDDAV-------IKLTYLHGER----CHN---NQFERTSEFRFTCHHHGN--------DKPTFINETHDCTYLFDWPTPKAC-----PPIKIIECTAQDSEGH-QYDLSALSLMHDNHMISSSQTG---RSYYINVCRSMLHRPT-VTCPPNSAACVRN-----ADNSFTNLGSVDSSP-HFVDGKLQLCYTHGDNCR--GNQNYTTTVTFECSQTSAVQTTPIVTSTED-ECNVQILWTTSAACPVQQGNT--LGSCKTSNPATGHEFDLSSLESTTFHTTQAL--HGKEYHINVCEKVNDPHC-PANSGNCL----HGSGSYISYGEANANLTYTD-GALALVYEHGDICPADDRYRH-STEISFVCHRSTSPNLQAPETPSTTPIHISDDEDTCTHYYTWSTYLACEQQFPCAVNDM--------TSAVPRIIDLSPFVRESGHYPTISNIVG----------DRGTYFINICRPLQPIQGVTCPPGSAACEVIP--GHQPQSLGSIDPTHHSMSYESETNTVSLVYHSNRRCTTSSNATATVTTQIVFSCLQGAGKGEPRLTAVTSDCVYLFSWPT--DLILCDSQTTSPSSGECKFTDEAKKLTYDLSKLETKT-----ITDTIGS--IQVSLCQPLSS---PPEC-QGAAVCYED-TSHHKVSLGSASTRTFSAGFSSEYPLTAHYADGAVCG--------------------------------------------------RTPNKHRSTTILFICDMN--PTEPELIH---------------------------------NGDECLYTVYWKTPLVCPPVSESCVVADTTLGLTFDLSLLSSLTHAWHFRNNG-YSYYFNLCRAVHTTGSHQ-CKDGSSVCRKK-GAEAKSFGIVSSQKTSVVSA-----TEIEITYTGGDKG-LCTS------GTAPLKTIIRLQCSPTIMGNPQFESFS--ENSCELRVAWKTRIACSDSPTSVTPDRTGKFVDPKSGVTFDLSPLMNKR---------------SWVAGGDLRE----DTSSYKYMISIRGNPDISSVNPSYSQACGDIATVCQYKKDT-NFVRKIGELSPHSPTYVMSDGTLELNFTSNDKCGKDPKKTSYVIISLSCGDN----GLGSPTFFYESNDCGYYFHWYTSVMCADIEIVSSN----KTNSRSNTPVSNPGSSTGQHSNAGGIRGWSFC----------------DRRGFVYSR--NCVP---------------------------------------------------------------------------------------------------------------------------------------------------------------------------------Blan_BL04963_IGFR2_evm0/1-2431                            M-----------------------------------------------------------------------------------------------------------------------------------------ISLYQLWEARGD--NNVQ--YNISICSQLALPT------PCSGNS-VCRL--QDNTTANIGNFNSTL-QIEEDRHGGGFRMELFSDEDC----------------PGHPGHKIGSSINIQCGRTLGFPEFVEA-KPCTTYFDWDSTVACTSVQNPAQKEVKCYVYDTDGKKRDLTPLIKQSGGY-LVD--SAGER-DFYINVCRDITA----GGS----TSPCPPGTAGCRVIG-GEAVDMGRPTT-DLQMGEDGRLFLHYNST-DGDAPTGCNGVKPTVHVTFVCPNGDG-RGGSKDPLLTLDTDCQYQVEWWTEYACPAEYLT-TTTCSFSKQQHDIDIDLSPLNVPEG-----TNPWPYKITTT--GDDGKS-YDYWLNVCGAVG--ISCGDN-DDDGSHTSVCQTSA-DT--AHIAGAI-GHTLRYSDGELTLTYKHGEVCKHNGFNRTSVITFRCNKTADNDGKGRPVFNSEEDCSYFFDWDTSYACL-QHEEGRSCRVSSPDGKKRYDLSTLVQE----GPLNWEALDGRHEHEGGDNDERFFMNVCGEVITLEQ-AADCPQGAAACAISDT--GGGATNIGRYQTDPVLE--GNHIKLTYTDGDSC---HDSLTWSTVIRLVCSPGDLESAPVLLRRSDEKCLYEFEWHTAAACPLG----------------------------------------------RKSGYDCRVFDDDAGFSFDLTPLSRAGTTADSF-YNVTNGKYDYLINVCGDINGTK--SCEDRN---TKAGACQVDRSDN--KAKSLGVFNSHLNYYDGLINLTYSEGDPYHDK----VNRSTHIAFLCDPKAGSGQPKFLAE---DNHFYYFKWYTKYACPEQPIECVVTDPNTHTQYDLSSLAKSEDDNGENWSYLDDTDPN-NKKKYYINVCRPINPV-------SGCDTYAAVCQTSFATGE----EAVTLPNMGMAELRPTIEGQAGHLLLTYSNGGPCTGPDGVARNYTTKIHLICQRGSLSSSPRFLEQMG-CEFVFTWETEAACGIKVET--QEDCTIPDPNSGYIFNLQPLTRGDRTEKKYYDVTSSD--GQAFKLNICGSVK-DGCPQ-YCDEDGHCEDASVCS-IATGKDKALAHARDAQLEFSDAGQLTLRYEGVR-DEGSGQVTS-VIIHFVCRTQVALGEPK--FLRQEG-------LSFLFEFATSLACPPAPVDCLVTDTAGNLYDLTPLSRTDQNWEPLNPESTPGHKDERYHINVCRPLVVTDNMDYSCPGGPLGSCMTG-----PQGNKNLGVVQSNPQAAGN-----SLSIRYYNGDMCR----DNVHYSTVIVFECSLTQG-TPTFQTTTQDGCEYAFLWRTPSACPIRRAV---GS-NCRVRDPLYGFEFDLSPLYNYTHDYRVRT---Q-QYEYNLNVCGPIHEGTGVCDAK-VHKDVSACQKTLPGVEPE--VYVTGTFNKTVVYEDGQ-------ILLNYTGGEK----CHT----VYNRSTAINFYCDHCADGKCKDGPKPGPVYVNETSDCTYVFEWATSYAC-----PPFKIVECVYHDDKGN-QFDLTPLSLSHGNWEIIPTSRSSNNEIFYINVCRSVVHSKG-VQCPYNAGACMKLPEAGDASSKFMNLGEVQAGP-WFEDGNLVLNYTLGANCTADGSRKKMTVIKFVCDEDS-VGNGPQFLGSTD-GCIYTFIWFTDVACPLKADQPS-EGHCKAKNPLTGYEFDLSGLNKKDSYYQVLDQTKEHAYQINVCGAVKGSKCEGDNIGVCQAEL-IHGERTFNGGKSNSNLDYNE-GILELTYKQGDHCH-KDQFER-SSVISFVCANRTDN---------GRPVFID-ESEDCTYYFSFHTPLACETQVPCIVDN------------GTNSFDLSPLIKAEGHFMAPSIG------------GTGVYYINICRPLNPIPGVRCPPGASACMKED--NKSPVSLGRVFEQPK---INPVTKEITIIYKHGSKCE--SNPDYNNTAQIVFRCEQGTSVGSPQFSYKTDDCVYFFEWKT--NVV-CAAPKPL-QTTECTYTDPSLKYTFNLTSLRSTGDNGYEVEDNVQGGKYSLNVCGTVPR----SGCDSDTAVCVTL-ADGKKISAGKFNTQTFKY---EGNLLTLSYSDGTTCRG----------------------------------------------------SEKYRTDILFKCADE-QNSQPAIFS----------------------------------MTGCSYTFMWKTSLACPPASQPCAINQN--GQTYDLNLLSQETNSWRFRDKFGNKYYLNLCQPVHQGPPR--CDPNAAACRQRPDGTVDNLGVVSSQTMNVDDT------GLKVVYSEGEAG-VCDDHSR-RSNSDSASIEIQFSCGSTV-GGPQLHSID-SDGRCHFVVNWRSAIACATERQAVTPDDSGKIRDPFSQSVIPISQLYNNNG-------------NSWRVTGDLRP----NRDVYVYYIRM------DDV-GIQEDTCRG-AAVCQVKEKD-SFMKVLGKPN--MINYYMQDGALEVQILSDTKCGRRQEKKTSTTIIFHCSDYI---QKGQPEFFSESHDCEYIFNWYTNVACLKDDVEESVSIPPPEQDLPDDGTGIHHFISQHKSSAATVVGIMLAIVIICV--LVIIFHKQERRQAFVLKVKSCCRR-GDYSHPTYRYTKFMSNGDE-D---GTLLMGGLDSDEE--------SGNSQ----------------SEEASDDFMVE-----------FDAESHQQNRAKRKKKTKPKADEN--HLEEVRXPFQKTKPKADENH-----------L-EEVRPYHDDSDEDMLDV-----------Bflo_BRAFLDRAFT_216256/1-2276                             M--------------------------------------------------------------------------------------------------------------------------------------------------------------------------------------------------------------------------ELFSDEDC----------------PGYPGHKIGTSINIQCGRTLGFPEFVEA-KPCTTYFDWDSTVACTSVQHPAQKEVKCYVYDTDGKKRDLTSLIKQSGGY-LVD--SAGER-DFYINVCRDITP----GGP----TSSCPPGTAGCRVIG-GEAVDMGRPTT-DLQMGEDGKLFLHYNGT-DGDAPSGCNGVKPTVHITFVCPNGDG-RGG--DPLLTLDTDCQYQVEWWTEFACPAEYLT-TTTCSFSKQQHDIDIDLSPLRVEEGRVVVLADYYCYK----------NA-YDYWLNVCGAVG--ISCGDN-DNDGTKTGVCQTSA-DT--AHIAGKVLGHTLRYSDGELTLTYKGGEVCRHNGFNRTSVITFRCNKTAGNDGKGRPVFNSEEDCSYFFDWDTSYACL-QHEEGRSCRVSSPDGKKRYDLSTLVQE----GPLNWEALDGRHEHGGGDNDERFFMNVCGEVITLEQ-AADCPQGAAACAISDT--GGGATNIGRYQTDPVLE--GNHIKLTYTDGDSC---HDSLTWSTVIRLVCSPGDLESAPVLLRRSDEKCLYEFEWHTAAACPLG----------------------------------------------RKSGYDCRVFDDDAGFSFDLTPLSRAGTTADSF-YNVTNGKYDYLINVCGDINGTQ--SCEDRN---AKAGACQVDRSNN--NAKSLGVFNSHLNYYDGLINLTYTGGDPYHDK----VNRSTHIAFLCDPKAGPGQPRFLAE---DNHFYYFKWYTKYACPEQPIECVVTDPNTHTQYDLSSLAKSEDDNGENWSYLDDSDPN-NKKKYYINVCRPINPV-------TGCDTYAAVCQTSFATGE----EAVTLPNMGMAESRPTIEGQAGHLLLTYSNGGPCTGPDGVARTYTTKIHLICQRGSLSSSPRFLEQMG-CEYVFTWETEAACGIKVEK--QEDCTIPDPNSGYIFNLQPLTRGDRTEKKYYDVTSSD--GQAFRLNICGSVK-DGCPQ-YCDEDGHCEDASVCS-VASGKDKALAHASDAQLEFSDAGQLTLRYDGVR-DEGSGMWRSYIIVHFVCRTQVALGEPK--FLRQEG-------LTFLFEFSTSLACPPAPVDCLVTDTAGNLYDLTPLSRTDHNWEPLNPESTPGHKDERYHINVCRPLVVTDNMDYNCPGGPLGSCMTG-----PQGNKNLGVVQSNPQAAGN-----SLSIRYYNGDMCR----DNVHYSTVIVFECSLTQG-TPTFQTTTQDGCEYAFLWRTPSACPIRRAI---GN-NCRVRDPLYGFEFDLSPLYNYTRDYRVRT---Q-QYEYSLNVCGPLHEGTGACDAK-VHKDVSACQKTLSGVEPENHLYLSGTFNKTVVYEDGQ-------ILLNYTGGEK----CHT----VYTRSTAINFYCDHCADGKCKDGPKPGPVYVNETSDCTYVFEWATSYAC-----PPFKIVECVYHDDKGN-QFDLTPLSLSHGNWEIIPTSRSSNNEIFYINVCRSVVHSKG-VQCPYNAGACMKLPESGDASTKFLNLGEVQGGP-WYEDGNLVLNYTLGANCTADGSRKKMTVIKFVCDTDS-VGNGPQFLGSTD-GCIYTFIWFTDVACPLKADQSS-EGHCKAKNPLTGYEFDLSELNKKDSYYQVLDQTKEHAYQINVCGAVKGSKCKGDDI-----EL-IHGQRTFNGGKSNSNLDYNE-GILELTYKQGDHCH-KDQFER-STVISFVCANRTDN---------GRPVFID-ESEDCTYYFSFHTPLACETQVPCIVDN------------GTNSFDLSPLIKAEGHFMAPSIG------------GTGAYYINICRPLNPIPGVRCPPGASACMKED--NKNPVSLGRVFEQPK---INPVTKEITIIYKHGSRCE--SNPDYNNTAQIVFRCEQGTSVGSPQFSYKTDDCVYFFEWKT--NVV-CAAPKPV-QTTQCTYTDPAIKYTFNLTSLRSTGDNGY--EDNVQGGKYSLNVCGTVPR----AGCDPDTAVCVTL-ADGKKISAGKFNTQTFKY---EGNLLTLSYSDGTTCRG----------------------------------------------------SEKYSTNIIFKCADE-ENSQPAIFSVSLCGYTASLQGDISFCLLITRWGCAWSLVWVNGMTGCSYTFMWKTSLACPPASQPCAINQN--GQTYDLNLLSQETNSWRFRDKFGNKYYLNLCQPVHQGPPR--CDPNAAACRQRPDGTVDNLGVVSSQTMNVDDT------GLKVVYSEGGAG-VCDDHSR-RSNSDTASIEVQFSCGSTV-GGPQLHSID-SNGRCHFVVNWMSAIACATTRQPVTPED-GKIRDPFSQSIIHISQLYNDNG-----------------VTGDLRP----NRDVYVYYIRM------DDV-GIQEDQCRG-AAVCQVKEQD-KFMKILGKPD--MINYYMQDGALEVQILSDTRCGRREEKKTSTTIIFHCSEYI---ERGQPEFFSESHDCEYIFNWYTKVACLKDDVEE------------NDGTGIHHFISQHKSSAATVVGIMLAIVIICV--LVIIFHKQERRQAFVRKMKSCCGRGGDLSHPTYRYTKV------------------------------------------------------------------------------------------------------------------------------------------------------------------Bbel_LOC109481228/1-2460                                  MAAAV-------------------------------------------------HL---WHRH-------DP--------------------KTSMFWTISSLFF-------VFQLVSR----TQGDCV-AGNYNLEPIQ--GLWEARGD--NNVQ--YNISICSQLTLPA------PCNGNS-VCRL--QSNVTSNIGNFNSSL-QIDEDRHGGGFRMELYSDDDC----------------PGHPGHKIGTSINIQCGRTLGFPEFVEA-KPCTTYFDWDSTVACTSVQRPAQKEVKCYVYGVDGKKRDLTPLIRQNGGY-LVD--SAGER-DFYINVCRDITP--ETGAP----TSSCPPGTAGCRVIG-GEAVDMGRPTT-DLQMGEEGRLFLHYNGT-DGDAPAGCNGVKPTVHITFVCPNGDG-RGGSKDPLLTLDTDCQYQVEWWTEYACPAEYLT-TTTCSFSKQQHDIDIDLSPLRVQEG-----TNPWPYKVTTT--GDDGKT-YDYWLNVCGAVG--IQCGDN-DPDGSHTSVCQTSA-DS--AHIAGAT-GHTLRYSDGELTLTYKHGEVCRHNGFNRTSVITFRCNKTADNNGRGRPVFNGEEDCSYFFDWDTSYACL-QHEEGRSCRVSSPDGKKRYDLSTLVQE----GPLNWEALDGRHEHEGGDNDERFFMNVCGEVITLEQ-AADCPQGAAACAISDT--GGGTTNIGRYQTDPVLE--GNHIKLTYTDGDSC---HNSLTWSTVIRLVCSPGDLESAPVLLRRSDEKCLYEFEWHTAAACPLG----------------------------------------------RKSGYDCRVFDDDAGFSFDLTPLSRAGTTADSF-YNVTNGKYDYLINVCGDINGTK--SCEDRN---PKAGACQVDRGNN--NAKSLGIFNSHLNYYDGLINLTYSGGDLYHDK----VNRSTHIAFLCDPKAGPGQPQFLAE---DNHFYYFKWYTKYACPEQPIECVVTDPNTHTQYDLSSLAKSEDDNGENWSYLDDSDPN-NKKKYYINVCRPINPV-------SGCDTYAAVCQTSFANGE----EAVTLPNMGMAESRPTIEGQAGHLLLTYSNGGPCTGPDGVARTYTTKIHLICQRGSLSSSPRFLEQMG-CEFVFTWETEAACGIKVET--QEDCTIPDPNSGYIFNLQPLTRGDRTQKKYYDVTSSD--GQAFRLNICGSVKDDGCPQ-YCDGDGHCEDASVCS-VSSGKNKALAHARDAQLEFSDAGQLTLRYYGVR-DEGSGQVTS-VMIHFVCRTQVALGEPK--FLRQEG-------LAFLFEFATSLACPPAPVDCLVTDTAGNLYDLTPLSRTDRNWEPLNPESTPGHKDERYHINVCRPLVVTDNMDYSCPGGPLGSCMTG-----PLGNKNLGVVQSNPQAAGN-----SLSIRYYNGDMCR----DNVHYSTVIVFECSLTQG-TPTFQTTTQDGCEYAFLWRTPSACPIRRAV---GN-NCRVRDPLYGFEFDLSPLYNYTHDYRVRT---Q-QYEYSLNVCGPLHEGTGMCDAK-VHKDVSSCQKTLPGVEPVV-AHVTGTFNKTVVYEDGQ-------ILLNYTGGEK----CHT----VYNRSTAINFYCDHCADGRCKDGPKPGPVYVNETSDCTYVFEWATSYAC-----PPFKIVECVYHDKEGN-QFDLTPLSLSHGNWEIIPTSRSSNNEIFYINVCRSVVHSRG-VQCPYNAGACMKLPEAGDAGEKFMNLGEVQSGP-WYEDGNLVLNYTLGENCTADGSRKKMTVIKFVCDADS-VGNGPQFLGSTD-GCIYTFIWFTDVACPLKAEQPS-EGNCKAKNPLTGYEFDLNGLNKKDSYYQVLDQTKEHAYQINVCGAVKGSKCDGDDLGVCQAEL-IHGTRTFSGGKFNSNLDYNE-GSLELTYKHGARCH-KDQFER-SSVVSFVCANRTDN---------GRPVFID-ESEDCTYYFSFHTPLACETQVPCIVDN------------GTNSFDLSPLIKAEGHFMAPSIG------------GTGVYYINICRPLNPIPGVRCPPGASACMKED--DKNPVSLGRVFEQPK---INPVTKEITIIYKHGSRCE--SNPNYNNTAQIVFRCEQGTSVGSPQFSYKTDDCVYFFEWKT--NVV-CAAPKPV-QTTECTYTDPALKYTFNLTSLRSTGDNGYEVEDNVQGGKYSLNVCGTVPR----AGCNKDSAVCVTL-ADGKKISAGKFNTQTFKY---EGNLLTLSYSDGTTCRG----------------------------------------------------SEKYSTNIIFKCAEE-QNSQPAIFS----------------------------------MTGCSYTFMWKTSLACPPASQPCAIQQN--GQTYDLNLLSQETNSWRFRDKFGNKYYLNLCQPVHQGPPR--CDPNAAACRQRNDGTVDNLGMVSSQTMNVDDT------GLQVMYSEGGAG-VCDDHAR-RSNSNAASIEIQFSCGSTV-GGPQLHSID-SNGRCHFVVNWRSAIACATERESVTPDESGKIRDPFSQSVIPISQLYNNNG-------------NSWRVTGDLRP----NRDVYVYYIRM------DDV-GIQEDQCRG-AAVCQVKEKD-SFMKVLGKPN--MINYYMQDGALEVQILSGTKCGRKQEKKTSTTIIFHCSEYI---ERGQPEFFSESHDCEYIFNWYTSVACLKDDVEESVSIPPPEQNLPDDGTGIPQFISRHKSSAATVVGIMLAIVIICV--LVIIFHKQERRQAFVHKMKSCCGR-GDYSHPTYRYTKFMSNGDE-D---GTLLMGGLDSDEE--------SGNSQ----------------SEEASDDFMVE-----------FDAESHQQNRAKRKK---------------------KTKPKSDENH-----------T-EEVRPYHDDSDEDMLDV-----------Spur_SPU_019695_IGF2R/MPR/1-2390                          M--------------------------------------------------------VDW----FIRC-------------------------------------------------------QAGTCWTL-----------GTWTAQTTV-DGTASTFYLNLCENVASSPGSTG---CDTTVSSCLK--QGDAFTNVGNHNDTL-QLTINENTGGFRLDYRGSV-C------------------NVTEKYQTTIDFKCGTTLGSPNFLEN-SGCTYFFEWYSTVACSSVNGPALNEVPCSIYDSKGKERDLSPLIKLKGGY-LVE--SPDAD-DLYINVCRDITA--DTSGP----TAGCPAGSAGCLVKK-GLSVGMGTPHL-KLESLSDDELKLHYGGA---GVLDSCNGFVPSVTVVFMCPREQGRFAATRGPIQLSSTNCQYQIQWQTEYACEVDMLS-TDTCAFNSSTHGIDFDLSPLTKPDG--------EFYLI------ESDPD-YYFYINICADMTAFANC--------EGMAVCQVARLNTVVGTSAGRNSNHELRYSDGELSLIYKGGDVCNHNKFQRTSVISFQCNKTK---DLGQPEFVTETECTYFFQWQTKYACMADAPSPTSCRL--ANGKQRYDLSSLARK----TGSNWLVLDGRHSH-SETTESEYFINVCSEVVQGDVASEGCPQGSSACVVDTVGTTKTGKSLGKYLTSPVWQ--NNLITMSYTEGDVC---KGTTKKRTDITFLCSPGDLESAPILVRKSDDGCQYDMEWHTAAACPLASKWGTDCRVFDDDAGDLESAPILVRKSDDGCQYDMEWHTAAACPLASKWGTDCRVFDDDAGVSFDLSPLKK----ADGH-YKVSVGEYDYYINVCSDIKLS---PCNDASHTLPHPAACQARKDAS-GVSYVLGQTNTSLGYFDGIIKLTYLNGKPYNSNGGTVTARMTEIAFLCDPEAGVGTPQFFQE---NNATYFFRWNTKYACPQPPIECVVTDEANSEQYDLSSLSKALEE--ENWSFVDERDAA-NRKKYYINVCRPINPH-------AECGAFAGVCQTGFTTSDQQTSETALIPNLGVPKSPPTIES-SGHLLLTYQNGSDC----GESKQITSHIHFACNKGTLASTPRLLEVVDSCTYSFLWETEAACPIGVST--GENCTVRDSNSDYVFNLQPLIK-----QEGYQVFGPT--NEVFKLNVCGAVPVQSCPA----GSGSCLSPTL----------SLG-SASSTLIFSDEGLLTLEYTGGTFNPQTAQYPS-TVISFLCRRNASQTSPSPVFVRQEG-------SRYIFNFETPLACLPESVDCLISDSNGNQYDLSPLAKDSGNWEAV--DTRQNYNGLSYHINVCRPINKGDPATSSCPGGPIGACQISES---DNSAYNLGYVQAMPEAATD----GALSLRYVNGDICH----AKFHRSTRINFECSDTPG-SPVFQTES-AECEYLFSWETPAACPLKRVT---GQGNCTVRVPEFGFQFNLSSLYNKTHDYTVTS---T-EHEYTINVCGPLSTTMGECASS---DGVAACQKLQDGT-----FKNAGVFNKNLTYANGV-------LKLNYTTGSV----CHT----VYKRSTAINFYCDENVIGK------GEPHYIQETSDCTYIFEWATPLAC-----PPFKVVECSYRDGTS--QYDLAPLSKMTDNYRVNAAGNNLD----------------G-EFCPNNAAACLQTVDST-GKKSYISLGEVGQGP-TMENGHLVLRYESGATCPDKTGRKRTTVINIQCDKNS-VGTAPMLDHVDG-DCQYHFLWLSNYACDITEDIVKPKGDCTAMNPISGFRFDFNSLKKQAGYTVMGS--DSHKYVINVCDALASSVCDGSNIGSCQEEL-VVQGNHFNAGVFNKNLFFDD-SILFLRYDFGHACH-NNHFNR-SSVINFVCPHQKDT--------VGEPVFIA-ESDDCTYYFSWHTSLACEKFIQCSVVN------------GSDTISLAPLINDQGYHLATTLLG-----------DGSLTYINLCRPLNPIPGLTCHPNSAACQVK---GGDPVNLGRVSEGPK---IG-ADGRLSITYTQGDHCS--SDGTKNVSSVIKFFCKQGVTQGTPTLEF-IEDCIYQFTWGT--NVV-CPATAPE-SDHDCTFFNTALQYRFDFTALSKENQ--AVVASSAKT--MKLKLCGLSSD--QSGEC-KGAAICLQAGQDKY--SLGQLSTQTISQ---EEEIFKVEYTGGSTCP--------------------------------------------------SSEGKKRQTTIILSCDPKPSKTSPTFFS---------------------------------SDENCHYSFHWATAAACPADTRPCDLAYQ--GNVYDLSPLSQITGSFQFQDSSKNMYYMNLCQPAYGTPED--CPPEASMCRQRPDGQVDVLGMVKTQQLTAKGDV----PDIVVTFFEGSQVDDCGSPDQPDSPSIPASSSITFKCANKM-GNPVFQRTELVDNKCQFQFLWESKVACKEQRVPVTLVE-KKITDPETNVIMDFTEIIDHG---------------TWEARGDNRKEVTGDPSSYVYSINMNDNQ--DNVPGDVSHVCQD-SAVCQTKESDQQFFRDVGTRS--SRKFFIEDELVEMEVTKPKSCGKDNTEDVVTTVIFQCETDS---RNGNPEFYYESSNCQYYFLWLTSVVCTEGKIVI----------VPDDGGGNDTVGTSNHKQASIIILILIIIIILCS--LAIVFHKKERRSAVYWRVRSCCPG-AYSKVPTYSYSQLTNGDPE-ESNDSRSLFAG--------------QEDEELLTAT-------------------------------------------------------------------------------------------RRNPFEFHDDSDDDILPH----------VSkow_LOC100366918_M6PR/1-2447                             M-----------------MTGGVR------------------------------------------------------------------GFYRKLLSCMCLVLYFV-----QYSTVA-----VNAECK-IGGFDLETLNQ-WTWEVRAADWNNIVSIYHINICKKFGTTESTAG---CQDSI-VCEE--KDNVFTNIGNYTETL--ANSIESSGGLRVDYKGIV-CPIKVVTGGIYTGPEDEPEDEIEYLQTTINFRCGKTLGSPEFVGK-FGCTYFFDWYTSAVCKSLSTEAKSEVPCYVYDANNKKRDLNPLIKTTGGY-LID--TEGEE-SFFINVCRDINP--QTGGA----TQNCHESAAACLIKG-DTAYDMGVPSMGRLEIIDDNTLKLHYEAT-VTPALAECNGVKPKVIMRFVCPKDEGIRGGGKDPMMLSSINCQYEIEWFTEYACEEEYSV-SSTCQLTQDTHGIDIDLSLLQKPSE------SEDKYIAEGK--GWDDKM-YRYYLDVCKNTD--IECD---GDHARDPAVWQTSE-DSNWCTVAGSNKDRILRFSDGELTLIYRHGDQCSHNGLERSSFISFKCNAAS---GIGEPKFVTEEECSYFFEWETKHACR-DHARDSNCHVTSSDGKQQFDLSTLVKT----EGSNWEALQHDES------DERYFLNVCGDILSSDPFTTNCPPGSAGCKIGT---DTGAQSLGKYSSSLIIE--GGNLKLTYSEGSPCVDTDSSIHMETTITFVCSPGDLESGPTLIRK--NKCLYEFEWHTAAACPLG----------------------------------------------KRKGDHCKVVDDEAGFSFDLSNLKK----EDN--YKVTYSMYDYYINVCKEVTGTGATNCD------GTAGACQKQTTGD--NAWITGLPSSELEYYDGVIRLTYSNGDPYRTTNNGQINRQTQIAFLCKPDAGNGTPEFIGE---GNYTYSFRWYTKYACPQIPIECTVTDPDTHKQYDLSSLSKAEED--ENWSVID---PY-TIQKYYINVCRSINPV-------AGCSTYAAACQTQINSQF---KEVIGIPNMGIASSRPTIES-SESILLEYTDGQDCTDVDNKIVKTAIRIHFICTPGNLESVPRFVEKIGNCQYSFMWNTEAACAIDDTK--GEECAVKDPNSEYTFNLQPLKR-----KTYYTVTTEEQPSQTFKINICDNVISSECSG----SSAACLLD-------NGKWKSIA-SKSTSPEFSDDGQLTLKYEGVR-DVNSGQFVN-VVITFLCRTSIQLGEPK--FVRKEG-------ITYLFDFETALACRPQSVDCLVYDSHANQYDLSSLSRESGNWEAV--DTRVGYEHLRYHINVCRPLNSAPNGDYICPGGPIGGCQTSDN--DPKQNFNLGYIQAMPEAGID----GSLSIQYLNGDQCH----GKYHRSTRINFECSTIQG-SPVFQDET-PECEYVFTWETPSACKLQQNI---GS-ECKVKDPRYGIEFDLSPLRNEAHDYHVTV---G-GYTYLVNVCGALKEGSGVCTGE-----VGSCQLLENGT-----PVDAGKYNQELKYDNGM-------LALNYTTGQK----CHS---DIYERATLLQFTCDEEVEGTNV----SSISFFKETDDCTYVFSWRTRYAC-----PPSKIVECSYRDQDGE-QYDLTPLSEMTHNYVVIPTIPGYKKEIYYINVCRSLKSEQD-INCPVNAAACLEY-ETDDGTKKWESLGTVSGEP-FMVNGQLQLHYELGDTCPGNPDKKKSAFITFSCQEDR-LESSPEFSYAVE-NCEYYFFWITKSACKLNHQEVV-TNDCTVENPESGLVFDLNVLKKDEGYKVLNT-EHGHLFDINICAPLKTSQCTGSNVGSCQSQVGINSGQAINAGIYNRKLFYND-GIISLNYSNGDPCP-DNKYHR-NSIFSFICNQDGDIGN------LGVPVYLD-DTDECTYYFSWHTPLVCENYIQCSVVKY---------GIEDLFIDLTPLITENGGYLAVSSLGDVPASD---LKSSDTYYINLCRPLNPIAGAKCPPGSSACRVRE--GEAPISLGKAAEEPY---IT-AEDEVAIKYVHGSKCP--QDEKKQLSMLITFHCKPGISETAPSVFG-YANCTYLVEWDT--DVV-CPKSKPAPATSACTYYDSALDYTFDLSPL-------VVLDDDGVE--YDINICAPVPG--AKGSC-ENAGVCKHHGTNNYGVSLGSASHQEFMY----DSELKLMYSKGDGCPGVV-----------------------------------------------SEEDVLQTTTIIFECDETIGHGKPELFL---------------------------------LGGICEYVFKWKTSLACPPSSEPCVTAYN--GKIYDLQVLSRATGHWEVKDKD-NVYFINLCQPVSEAK----CPEGSAICVRKSDGSYESLGLAKTQHMK----------------------------------GIVEITNIELQCSTTVGKGPVIRSAD--SESCNFYFEWQSRVACSIEKEFVQMHD-EKVTDPVSNGVIDLSPLIDKG---------------LYTVDGDIRL--NGKLEQYVYLINLKKDG-LDSVEGDTDGKCKG-AAICQYKKDSNSMYRNIGMAS--PKSLTMEADILEIVVTNLGTCHKDNIDDVTSTIVFYCDNEADPSQLGVPEFQYESMNCQYFFNWHTPAVCVQAQLESSI------------SNRLSATVKDNPGTVGIVILIVLLSITICI--LLIVFHKPERRSAFARKVRGCCCP-G-YTVPSYKYSKLADNDVESD------LLMNIPEREY------------------------------DDDSEEEPANPVEVV------ASAPEPKPKTKKDKKSKREKPDEK------DKKKKKKSKTKKDEPLLDMDVDIKNGPFKGKSIAYHDDSDEDMLNI-----------Cgig_CGI_10023625_M6PR/1-2378                             M----------------------------------------------------------------------PYI-------------------------------------------------YTEKCL-VNNNDFSAVERFGPWEVISYDGNNT---YQINLCGRVPTYSAGVGKPNCHNDTVVCMV--QDGQAFSLANYTNNTINAPSNDQEAEMWIVRNGDE-C----------------PDLAGENLNSIINLKCGKTLGFPKFLEH-TYCTSYFEWRTSYACRNMPV-SHHEVPCYVYNGKGQLIDLSALVKTQGGY-LVD--SAEGW-EFYINVCRDITA--GTGDK----TSQCPPGSAACRVKN-DTSIDMGHTDK-KLQVDARGHPSLTYTSN---VTVPGCT-AKPKTTIQFVCPES----GGSEDPQLEFDFNCEYRVLWKTEHACPDVAVT-SSTCRLHNPQRNLDIDLSPLTNSPG------VDKPYEVYVNASGPGSRP-MRYYINVCGELG--IQCPD--DESVRGVAVCQTMVGNSSWGHVLGKTDHQKLKYVDGLLTLTYKGGEKCNHNHFQRETIINFHCNHSAAYNGQGWPVFNREEDCSYLFDWDTKYACL-DHPVIEECRV--NHNGKRFDLSPLVKH----TGKNWNVLEGG----GQDGLSMYYINICSDLIHSDQ-AARCKSDSSVCLLDN----SGAHSLGRYLQNPTFDPRSNTIQITYTEGDSCR--DNTQKKSSVVTFICKPGDMDSGPVFLRRSLDECVYEFEWQTAAACVLS----------------------------------------------RETGSGCKVYNEDLGINFDFNKLQA----AEGHYYNVTNGDYDYILNLCGPVKNT---LCDQKASQVTNPGVCQVKHGGTAADAFVTGQANTNLTYYDGLVKIKYEQGAMYNSNP--PTPRRTEITLICDRHAGRGSPVFVDEGLTSSGTYTFAWNTEYACPSSPID----------------LSRGTDE--ENWAVVDESNPG-SRKKFYLNVCTPLKEIPVG----SGCSPFAAVCQTSYQNSQ----EQLVFDNLGEVTSGPTVEG-EGLLTLRYVSVKN----ECQGKNWTSTIHFVCKKGALAKGPNPPQKIGDCEYSFVWETEAACPIADTNIEGKNCSIKDRNSDYTFDLSPLRKS--GDQDFYEVTAMG--GYKIRMNLCGSIKSPHCAAIEGAESAACLET------AAGLSQGLA-TLSHTLDYSEDGRLMMTFDGVR--QQNGQRTQ-VIVLFLCRQNIPLGAPE--FVRKEE-------NAYYFDFKTSLACRPQPVDCQVQDEKGTQYDLSPLARSGTNWQVL--DTRPGFSDLTYYINVCRPINSVA--GSTCPGGPVGGCQVST----SGRAFNMGYIQSQPVVAGN----GTLTLRYIGGDHCHKGKPNEAPRSTRINFFCSPSEH-SPAFEAET-DTCEYIFNWLTPAACPVQRIV---GQ-NCVVSDPLYGYSFDFNKLRKADANYLVTS---G-EYAYELNVCGPLVKS-SKCTDS----SIASCQTKPADS-NF--HFDAGHSSSSLVYDSGE-------ITLTYDNGHL----CHQ----KYNRSTVITFVCDQSKTGL------AGPTFLNETEDCVYQFVWPTAQAC-----PPFKVADCGLRDPSGG-QYDLSSLSLNDDNYQTIDHVAK---KKYIFNVCRSLVHQKG-ETCPFNSAACIVDLGVTDPKKKFHNIGEVSEQQVQFLDGHLQLVYTNGEACKEDPTKHVTTRILLYCSKDS-IDTSPAGHFQV--GCEHRFVWQTAAACPIQTSKGD-GANCTVTNPSTGYAFDLSSLKRSEGYTVDDR--KDHKFTLNVCGAVAGTTC-VASTGTCQVEK-KGEMRAFNAGKWNANLQYDD-GILFLNYSGGDKCH-NNQFER-NVIISFICSQGAGQ---------GQPEFIA-ETGDCTYQFVWHTELACEEQVRCSVEK------------DGYTVDLSPLIKMSGHHLAVSTAG--HGTD-----TDGTFYINICRPLNPIYGKLCPPGASACQDRV--GKPPISLGKARTRPQ---LDPVSNKIVLLYDHGSPCP--SNPAANITSKIVFNCKPGPLAGVPVLEY-VAGCQYIFEWDT--NVV-CERNNTDTAKGKCVYQDPISGALYNFTGLRKAQP--IKLYQNALQ--YLISLCEGLGA--EYPGC-EQASLCRWNGTLGH--GYGQVTTGEFIS---VEEQLKLAYKDGKSCNG------------------------------------------------------KSEGFMNFECDPNIYPGSPKIIF----------------------------------AKECSATFLWLTRAVCATVADQCTLAYN--GHVYDLSVLSRQQGSWNLTDAQRNTYWINICQGIHDGPDPQICSERSASCLRTTDGKIHNLGTVTSQTLSMEEDG----KTLRLEYTSNDL--ACTGKHR-RSDNLRTRTIIHFECGNSV-GGPVYIPRQPNSDECVFEFRWKSTVACHVERHSVQENK-GKIIDPESSS-----DTYKRKYDFIVVIYLFVEDVNSWVSSAHEFTLVTVPGESTEYQIDLAG----------TSTSCPPGTTICKVDKSQ-ATKTSLGLTT--GHQFYVEDDIVEVVFQTEQICDKDKQKKISSILQFHCNPW----GDSKPQFLFDSMDCTYMFTWENPHSCAHPVVVNPLPLP-----VDKAGTGNGSTGSRSGSTVGTIVAVFLSAIVICL--LLIVFHKKERRDAVASRLRGLFRR----HSSSYRYSEIPSSEVE-D------LLPEISASDL------GIQGSVQVTT----------------DTEDISIL------------------------------RSEE-----------------------------------SPAVISYHDDSDEELLA------------Obim_Ocbimv22009958_Ocbimv22008285_IGF2R/M6PR_merge/1-2284 M-----------------------------------------------------------------------------------------AWTRFWLSLIVAVFIHG-----VYVLCD-----NEKGCK-IGDYDFSPLDLSYPWMSVSLS-NDT---YWISICSAVNHDK-------CPTGSSVCRE--RSGSFISVGNYTSSP-SMFEN-EDKEITVSFTGGV-C----------------PSNSSAHLRTDILFKCGKTLGSPNFLSQDNSCTEVFEWESLVACKKRPEKVVKEVPCYVY-VKGKKRDLTPLIKLKGAYKLVT--DSDGSPHFVTNICRAITE--DPADQ-----LGCPKGSASCGKIQ-KFLVDAGQPTT-ALRAIDERTLQLVYKSE---TSASSCV-NTPQTVITFICPAN----GRVSHPPLVSQSGCLFEIDWITQYACEESEISVKNSLKLIREKDNIDFDLTPLSKE-----------NYIVDVT--EKDGSK-YKISLAIGHT----IECFGSVDDDKYHPSVCQRKPNNKEFARALGNEQHSVLRYSDGHLTLTYKEGQPCSSN-FKRKTIIEFHCNKSAVN---SKPVYRYEEYCSYYLDWYTPYACI-DHPLQEKCSV--VDNGKLFDLSSLRYT----SDNNWVALNGHEY----NDDAEILLNVCHDIMPSGK-AVKCPTDSAICLKKF---DGTTKSLGRYQHEPVYNDVTKTLQLNYTDGDKC----RSGKIFTVITFFCSPGISDNAPVVVTKSSDDCSYYLEWHTAAACVVT----------------------------------------------KKKGADCQVEN----------KIMK---------------------------------------------------------VSKNVGHANSILTYEDGILKLIYENGDKYNTDP--PIARRSVITFVCDTKASPGRPEFIKE---SNSTYWFLWYTKFACVSHPVECTFTDEQTHEQYDLSSLTKMS----DNWVTTGIDAPV---QKYYINVCRSLNHVPVG----TGCDVHSAVCSTKFEGGK----EVIAHENLGEVVKG-LVKK-GDNIVLEYTNGKACLDSGYTLTNYSTTIHFHCTKKITEHGPSLLSINSICHYVFLWNTPAACVQTSTESNQEKCSIKDTNSGYIYNLQPLIR-----DGSYSIVSQ---SHKFYLNICAPVNLPGCTMSDNSKASVCEQV-------SSTVTGLA-AVSTELELTDR-HLQLVYAGKR--LSDGSLFK-VTIIFVCAKEKELGSVS--LVRTTK-------SNYIFQFDTPLACPPQTVDCVVQDRLGNQYDLSQLTKQNGNWELP-------VGNMKYIINICSPINNYT--GNTTCAGNVGGCQIAP----GRGEYNMGYVQGKPIALSD----GTLSLRYRNGDLCHKGTDKQSHRSTRINFFCSTVEH-FISFGSET-EFCEYIFTWRTPAACPIKRIT---GK-FCKVVDPLYKNEFDLSVLR-KSTDYHVIG---G-GYDFLLNVCGSLNTELENCNKN---TGSAACQTRSSDP-AF--VINIGKASDELVYEDGI-------IFLTYKDGKS---NCHK----KFSRKTIITFTCDPNVEGT------TGPHYIEEKEDCSYLFEWPTKHVC-----PKITTSNCKIQDVKGN-EYDFSGLRLSSNNYIYLSPEHEK--QKFILNVCQTLVHRKG-ETCPSSSAACSINLNETDTTKKYHSIGELGSNPLELFGAYPQLVYKNGEPCR--EGKNSSTYITFQCDLSA-VDSSPKEYYYIESKCEHHFLWATREACPLSQKASH-NNTCRVTDESTGVTFDLSQLKKSKGYTISDQ--KEHSFQLNICGAVANSQC-SKESSSCQTN--TYANRSFNCGNANSNLQFKE-GVIFLNYSGGDKCH-GGKFER-NTIINFVCNPSKGI---------GAPVYLA-ESKNCTYYFSWHTDLVCEKQVRCTVIK------------GDQMFDLSPLVQMSGHHVAEGLA---PSED-----PGASYYINICRPLNPIFGSFCPPLASVCETNV--GENGVSLGSVSEGPY---IDPNDQAVTIHYKNGAPCP---NSKQNRSTIIKFRCKVGPSLGHPVLMDAIDSCTYIFNWDT--NLV-CDKSSPD-AAKNCVYKDTRTLIEYDFSSLYNSAV--DKIPADPSG-NFLIRVCGPVSG--VSSNC-EKSGACYVD-KNNHEVNYGKASDAVFSI---DQNLISLTYRNGDLCS--------------------------------------------------SESGTRASTKILFLCNNTAGLGKPVLYE----------------------------------KTPCQLVFLWKTRLACPPAVKDCFLSYL--NSSYDLSSLAN-TQSWKAMAITGETYWINVCQSLYTRPKSQNCSSNGAAVCVESKNEVMTIGSTSRIVLYVDRNSPVNNPVIILQYSSDTL--VCSNK--------RATTIIRFSCSETL-GKPVFSKKN--LETCTYEFNWDTYLACKEDRETLKEVN-GVLIDDKSSSRIYFGKQTQGK---------------TFRVEE------TKGTEKFYYDISFEGKL-YPDDENEGSDKCKN-AAVCQRKKGDSSYYKNLGSAT--KKRFYMDAAYMDVEITSPEKCHSNPSKNIVSVFEFFCTHLG---SQENPTFVYESNECVFLFYWHSSIACSYYNVSDPS------------QNKEVSVASSNYHIIVIAVSVTFSIIVIAV--LLKILMKPEQKQIMKENIKNIFTR---NRTVKARYINVPQIDDG-D-DEGLLVMED--------------------------------------------------------------------------------------------------------------------EHNITYHDDSDEDMIL------------Dmel_lerp/1-917                                           M----------------------R----------------------------------------------------------------------------------------------------------------------------------------------------------------------------------------------------------C--------------------------------------------------------------------------------------------------------------------------------------------------------------SLQ--------------------------------------------------------------------------------------------------------------------------------------------KDC---------------------------------------------------------------------------------------------------------------------------------------------------------------------------------------------------------------------------------------------------AAPT---------------------------------------------------------------------------------------------------QENF----------------------------------------------------------------------------------------------------------------------------------------------------------------------------RQMRF------------------------------------------------------PIVIS---LLLLLFCSGESVAA--------------------------------------------------------------------------------------------------------------------------------------------DAANQLKFSTT--------------------------------------------------------------------------------------------------------------------------------------------------------------------------------------------------------------------------------------------------ECKLKEPIYGSTFDFSGLH-SDLAHVVKSMNIG-GDQFEFNICGNLSRT---CNGE---SNVAACLKRQGK------EYILGR-QHELFYNNGN-------MFLKYKSGAK----CDNGTADKPNYQLHVMFSCDYTLDAQPMH-VTP---YANEV--CSFYISYRTPLACLSIP-EGLQSNSCRVGDTKSNGTFDLMPLS--DSNYHTSNRQG----AFFVINVCKPVLYGEN-SMCPAGSSVCLFDSKATNPKERFINFGNVQTHP-VVEKGQLLLRHESPTPCAKNSSANYTSVIYFSCDKFI-RNAHPEFAGLGADSCTYQFNFATPLAC-------NDLKPCTAFT-STNELLDLSSLSSKPARTLLKD---GKNYTIAVCAH-AGAPC-QENGGACYEQ----NSTTISLGNSNSQLRFNQTGSLYLLYEDGAECSTATGMRRWSTKIEFVCANNATKDNGASTAGGSDSLKII-EDSNCQLLIQYQTPLACREPIRCKATTYVDHTNDGLGSSGDELIDLTPLISATDNYEARVEL---PASMEHLVPKTTKFFLNVCRPLVPKYQLGCAGGSAACMAKVTAAGAPEEERSMGFPLVSL-TQRNRTFAELAYLKGDPCP--TDNTSELSTHILFNCNMRAGRGQPVLRS-VEDCAYRFEWET--NVF-CPPHECTFSADTCDLVHDELGRRFNFKSAPFTKDGKIEIDYNATK--MSVNICGAHRK--AMTDY-SQALVNIFFTHESP--NCGREGTMNVQIR--------------------------------------------------------------------------------LICSDQ-TESSSTISS----------------------------------DQQCNLLYVQRTP---------------------------------------------SICEFLSLGATQQNFESNGSTSSSTSSSSTSTTTSTTTQASPA--------------------------------------------------GKP-------------------TKAATSTTTTTVGP-------DPAATP---------------------------------------------------------------------------------------IGPTA----------------------------------------------------------------------------------------------------------SVGTILGAILS-VTFCVTCLGLLAFSPARRQ----RIRRLFRR----SNSAVRYSRVQSNEEA------NLLLEP----------------NGEFT-------------------------------------------------------------------------------------------------------ESDDDMLL------------4. IRSHsap_IRS1/1-1242                   --------MAS-----------------------------------------------------------------------------------------------------------------------------------------------------------------------------------------------------------------------------------------------------PPESD-GFSDVRKVGYLR--------------------------------------------KPKSMHKRFFVLRAASEAG-----------GPARLEYYENEKKWRHKSSA-------------------PKRSIPLESCFNI-NKRADSKNKHLVALYTRDEHFAIAADSE-AEQDSWYQALLQLH-NRAKGHHDGAAALG------------AGGGGGSCSG------SSGLGEAGEDLSYGD-VPPGP--------------------AFKE----------------VWQVILKPKGLGQT----KNL-IGIYRLCLTSKTISFVKLN--SE----------------AAAVVLQLMNIRRCGHS--ENFFFIEVGRSAVTGPGEFWMQVDDSVVAQNMHETILEAMRAMSD------------------EFRPRSKSQSSS-----NCSNPISVPLR-RH------HLNNPPPSQVGL-----TRRSRTESITATSPAS----------------------------------------------------MVGG------------KPG-SFRVRASS----------DGEGTMS--------RPA---SVDGSPVSPSTNRTHAH---------RHR------------------------GSAR------------LHPPLNHSRS----IPMPASRC-SPSA--------TSPVSLSSSST---------------SGHGSTSDCL------------------FPRRSSASVSGSPSDGGFISSD-EYGSSPCDFRS----------SFRSVTPDSLGH---------------TPPARG---EEELSNYICMGGKGPST---LTAPNGHYI--LSRGGNGHR-------CTPGTGLGTSPA-LAGDEAASAADLDNRFRKRTHSAGT---SP----TITHQKTPSQSSVASIEEYTEMMP----AYPPG-GGSGGRL--PGHRHSAF--VPTRSYPEEGLEMHPLE-RRGGHHRPDSST-----------------LHTDDGYMPMSPGVAPVP---SGRKGSGDYMP---MSP------KSVSAP-----QQIINPIR----------------------------------RH-PQRVDPNGYMMMSPSGGCSPDIGGGPSSSSSSSNAVPSGTSYGKLWTNGVGGHHSHVLPHPKPPVESSGGKLL----PCTGDYMNMSPVGDS--------------------NTSSPS----DCYYGPED-------------PQHK--PVLSYYSLPR--------SFKHTQRPG-----EPEEGARHQHLRLSTSS-------GRLLYAA-TADDSSSST----SSDSLGG----GYCGARLEPSLPHP-----HHQVLQPHLPR----KVD-TAAQTN----SRLARPTRLSL-GDPK--------------------------------------------ASTLPRAREQQQQQQPLLHPPEPKSPGEYVNIEFGSDQ-----------------------------SGYLSGPVAFH---------SSPSVRCPSQLQPAPREEETGTEEYMKMDLGP--------GRRAAWQESTGVEMGRLGPAP----------------PGAASICRPTRAVP----------------------SSRGDYMTMQM-----------SCPRQSYVD----TSPAAPVSYADMRTGIAAEEVSLPRATMAA-ASSSSAASASPTGPQGA---------------------AELAA-----HSSLLGG------------PQGPGGMSAFTRVNLSPNRNQSAKVI------RADPQGCR---------RRHSSET---------FSST--------PSATRVGNTVPFGA-GAAVGG-GG----------GSSSSSEDVKRHSSASFENVWLRPGE----------------------------------------------------------LGGAPKEPAKLCGAAGGLENG-LNYIDLDLVKDFKQCPQECTP-----EPQPPPPPPPHQ-----PLGSGESS-----------------------STRRSSEDLSAYASISFQKQPEDRQ--------------------------------------Hsap_IRS2/1-1338                   --------MAS--------------------------------------------------------------------------------------------------------------------------------------------PPRHGPPGPASG---------------------------------------------------------------------------------------DGPNLNNNNNN-NNHSVRKCGYLR--------------------------------------------KQKHGHKRFFVLRGPGAGGDEATAGGGSAPQPPRLEYYESEKKWRSKAGA-------------------PKRVIALDCCLNI-NKRADAKHKYLIALYTKDEYFAVAAENE-QEQEGWYRALTDLV-SEGRAAAGDAPP--------------AAAPAASCSASLPGALGGSAGAAGAEDSYGLVAPATA--------------------AYRE----------------VWQVNLKPKGLGQS----KNL-TGVYRLCLSARTIGFVKLN--CE----------------QPSVTLQLMNIRRCGHS--DSFFFIEVGRSAVTGPGELWMQADDSVVAQNIHETILEAMKALKE----------------LFEFRPRSKSQSSG----SSATHPISVPGA-RRH----HHLVNLPPSQTGL-----VRRSRTDSLAATPPAA--------------------------------------------------------------------KCS-SCRVRTAS----------EGDGGAAAGAAAAGARPV---SVAGSPLSPGPVRAPLS---------RSHTLSG-------------------GCGGRGSKVA----LLPAGGALQHSRS----MSMPVAHS-PPAA--------TSPGSLSSS-----------------SGHGSGSYPPPPGPHPPLPHPLHHGPGQRPSSGSASASGSPSDPGFMSLD-EYGSSPGDLRAFC--------SHRSNTPESIAE---------------TPPARDGGGGGEFYGYMTMDRPLSHC-----------------GRSYRRVS-----------------------GDAAQDLDRGLRKRTYSLTT----P------ARQRPVPQPSSASLDEYTLMRA----TFS-G---SAGRLCPSCPASSPK--VAYHPYPEDYGDI------EIGSHRSSSSN-----------------LGADDGYMPMTPGAALAG-SGSGSCRSDDYMP---MSP------ASVSAP-----KQILQPRAA------AAAAAAVPSAGPAGPAPTS----AAGRTF---PASGGGYKASSP-AESSPE-----------------DSGYMRMWCGS------------KLSMEHADGKL-----LPNGDYLNVSPSDAV--------------------TTGTPP----DFFSAALH----------PGGEPLRGVPGCCYSSLPR--------SYKAPYTCG---------GD--SDQYVLMSS-----PVGRIL-----EEERLEPQATPGPSQAASA----------FGAGPTQP----PHPVVPSPVRPS--GGRPE-GFLGQR----GRAVRPTRLSL-------------------EG----------------------------LPSLPSMHEYP-------LPPEPKSPGEYINIDFGEPG---------ARLSPPAPPLLASAASSSSLLSASSPASSLG---SGTPGTSSDSR------------QRSPLSDYMNLDFSS------------PKSPKPGAPSG---------HPV----------GSLDGLLSPEASSPYPPLPPRPSASPSSSLQPPPPPPAPGELYRLPPASAVATAQGPGAASSLSSDT-------GDNGDYTEMAFGVAA----TPPQPIAAPPKPEAARVASP---------------------------TSGVK-----RLSLME---------------QVSGVEAFLQASQPPDPHRGAKVI------RADPQGGR---------RRHSSET---------FSST--------TTVTPVSP-----------------------------SFAHNPKRHNSASVENVSLRKSSEGGVGVGP--GGGDEPPTSPRQLQP--APPLAPQGRPWTPGQPGGLVGC-----------PGSGGSPMRRETSAGFQNG-LNYIAIDVREEPGLP------------PQPQPPPPPLPQ----PGDKSSWGRTRSLGGLISAVGVGSTGGGCGGPGPGALPPANTYASIDFLSHH-LKEATIVKE--------------------------------Hsap_IRS4/1-1257                   --------MAS----CSFTRDQAT------------------------------------RRLR--------------------------------------GAAAAAAAA--------------------------------------LAAVVTTPLLSSGTPTALI-------------------------GTGSSC----PGA-----------MWLS----------------------TATGSRSDSE--SEEEDLPVGEEVCKRGYLR--------------------------------------------KQKHGHRRYFVLKLETAD------------APARLEYYENARKFRHSVRAAAAAAAAAASGAAIPPLIPPRRVITLYQCFSV-SQRADARYRHLIALFTQDEYFAMVAENE-SEQESWYLLLSRLI-LESKRRRCGTLG-------------------------------AQPDGEPAALAAAA-AAEPP--------------------FYKD----------------VWQVIVKPRGLGHR----KEL-SGVFRLCLTDEEVVFVRLN--TE----------------VASVVVQLLSIRRCGHS--EQYFFLEVGRSTVIGPGELWMQVDDCVVAQNMHELFLEKMRALCA-----------------DEYRARCRSYSIS-----IGAHLLTLLSA-RR------HLGLVPLEPGGW-----LRRSRFEQFC--------------------------------------------------------------------------------HLRAIG----------DGEDEML------FTRRF--VTPSEPVAHSRRGRLHLP---------RGR------------------------RSRRAV----------SVPASFFRRL----APSPARPR-HPAEAPNNGARLSSEVSGSGSGNFGE-EG---NPQGKEDQEGSGGDYM-------------------PMNNWGSGNGRGSGGGQGSNG-QGSSSHSSGGNQCSGEGQGSRGGQGSNGQGSGG---------------NQCSRD--GQGTAGGHGSGGGQRPG---------------GGHGSGGGQGPGDGHGSGGGKNSGGGKGSGSGKGSDGDGERGKSLKKRSYFGKL---------TQSKQQQMPPPPPPPPPPPPAGGTG-------GKGKSGGRF--RLYFCVDRGATKECKEAKEVKDAEIPEGAARGPHRARAFD-----------------EDEDDPYVPMRPGVA------TPLVSSSDYMP---MAP------QNVSAS-----KKRHSRSP---------------------------------------FEDSRGYMMMFPRVS-PPPAPSP---------------------------------PKAPDTNKEDDSKDN----DSESDYMFMAPGAGA--------------------IPKNPR--------------------NPQGGSSSK--SWSSYFSLPN--------PFRSSPLGQ---------ND--NSEYVPMLP-------GKFLGRGLDKEVSYNWDPKDAASKPSG------------EGSFSKPGDG------GSPSKPS--------DHEPPK----NKAKRPNRLSF-ITKG--------------------------------------------YKIKPKPQKPTH------EQREADSSSDYVNMDFTKR----------ESNTPAP--------------STQGLPDSWG---------IIAEP------------RQSAFSNYVNVEFGV-------------PFPNPANDLS---------DLLR---------------AIPRA---------------------NPLSLDSARWPLPPL---------P-LSATGSNAI-------EEEGDYIEVIFNSAM----TPAMALAD---------------------------------------SAIRYDAETGRIYVVD-------------PFSECCMDISLSPSRCSEPPPVARLL------QEEEQ-ER---------RRPQSRSQS------FFAAA--------RAAVSAFPTDSLERDLSPSSA-PAVASAAEPTLALSQVVAAASALAAAPGIG----AAAAAAGFDSA-----------SARWFQPVAN-------------------------------AADAEAVRGAQDVAGGSNPGAHN-PSANLARGDNQA------GG-AAAAAAAPEPPPRSRRVPRPPERED-----------------------------SDNDDDTHVRMDFARRDNQFDSPKRGR--------------------------------Mmus_IRS1/1-1231                   --------MAS-----------------------------------------------------------------------------------------------------------------------------------------------------------------------------------------------------------------------------------------------------PPDTD-GFSDVRKVGYLR--------------------------------------------KPKSMHKRFFVLRAASEAG-----------GPARLEYYENEKKWRHKSSA-------------------PKRSIPLESCFNI-NKRADSKNKHLVALYTRDEHFAIAADSE-AEQDSWYQALLQLH-NRAKAHHDGA----------------GGGCGGSCSG------SSGVGEAGEDLSY-D-TGPGP--------------------AFKE----------------VWQVILKPKGLGQT----KNL-IGIYRLCLTSKTISFVKLN--SE----------------AAAVVLQLMNIRRCGHS--ENFFFIEVGRSAVTGPGEFWMQVDDSVVAQNMHETILEAMRAMSD------------------EFRPRSKSQSSS-----SCSNPISVPLR-RH------HLNNPPPSQVGL-----TRRSRTESITATSPAS----------------------------------------------------MVGG------------KPG-SFRVRASS----------DGEGTMS--------RPA---SVDGSPVSPSTNRTHAH---------RHR------------------------GSSR------------LHPPLNHSRS----IPMPSSRC-SPSA--------TSPVSLSSSST---------------SGHGSTSDCL------------------FPRRSSASVSGSPSDGGFISSD-EYGSSPCDFRS----------SFRSVTPDSLGH---------------TPPARG---EEELSNYICMGGKGAST---LAAPNGHYI--LSRGGNGHR-------YIPGANLGTSPA-LPGDEAAGAADLDNRFRKRTHSAGT---SP----TISHQKTPSQSSVASIEEYTEMMPA---AYPPG-GGSGGRL--PGYRHSAF--VPTHSYPEEGLEMHHLE-RRGGHHRPDTSN-----------------LHTDDGYMPMSPGVAPVP---SNRKGNGDYMP---MSP------KSVSAP-----QQIINPIR----------------------------------RH-PQRVDPNGYMMMSPSGSCSPDIGGG-SSSSSSISAAPSGSSYGKPWTNGVGGHHTHALPHAKPPVESGGGKLL----PCTGDYMNMSPVGDS--------------------NTSSPS----ECYYGPED-------------PQHK--PVLSYYSLPR--------SFKHTQRPG-----EPEEGARHQHLRLSSSS-------GRLRYTA-TAEDSSSST----SSDSLGG----GYCGARPESSLTHP-----HHHVLQPHLPR----KVD-TAAQTN----SRLARPTRLSL-GDPK--------------------------------------------ASTLPRVREQQQQQQSSLHPPEPKSPGEYVNIEFGSGQ-----------------------------PGYLAGPATSR---------SSPSVRCPPQLHPAPR-EETGSEEYMNMDLGP--------GRRATWQESGGVELGRIGPAP----------------PGSATVCRPTRSVP----------------------NSRGDYMTMQI-----------GCPRQSYVD----TSPVAPVSYADMRTGIAAEKASLPRPTGAA-PPPSSTASSSVT-PQGA--------------------TAEQAT-----HSSLLGG------------PQGPGGMSAFTRVNLSPNHNQSAKVI------RADTQGCR---------RRHSSET---------FSAP-----------TRAGNTVPFGA-GAAVGGSGG----------GGGGGSEDVKRHSSASFENVWLRPGD----------------------------------------------------------LGGVSKESAPVCGAAGGLEKS-LNYIDLDLAKERSQD---CPS-----QQQSLPPPPPHQ-----PLGSNEGN-----------------------SPRRSSEDLSNYASISFQKQPEDRQ--------------------------------------Mmus_IRS2/1-1321                   --------MAS--------------------------------------------------------------------------------------------------------------------------------------------APLPGPPASAGG---------------------------------------------------------------------------------------DGPNLNNNNNN-NNHSVRKCGYLR--------------------------------------------KQKHGHKRFFVLRGPGTGGDEASAAGGSPPQPPRLEYYESEKKWRSKAGA-------------------PKRVIALDCCLNI-NKRADAKHKYLIALYTKDEYFAVAAENE-QEQEGWYRALTDLV-SEGRSGEGG-----------------SGTTGGSCSASLPGVLGGSAGAAGCDDNYGLVTPATA--------------------VYRE----------------VWQVNLKPKGLGQS----KNL-TGVYRLCLSARTIGFVKLN--CE----------------QPSVTLQLMNIRRCGHS--DSFFFIEVGRSAVTGPGELWMQADDSVVAQNIHETILEAMKALKE----------------LFEFRPRSKSQSSG----SSATHPISVPGA-RRH----HHLVNLPPSQTGL-----VRRSRTDSLAATPPAA--------------------------------------------------------------------KCT-SCRVRTAS----------EGDGGAAGGAGTAGGRPM---SVAGSPLSPGPVRAPLS---------RSHTLSA-------------------GCGGRPSKVT----LAPAGGALQHSRS----MSMPVAHS-PPAA--------TSPGSLSSS-----------------SGHGSGSYPLPPGSHPHLPHPLHHPQGQRPSSGSASASGSPSDPGFMSLD-EYGSSPGDLRAFS--------SHRSNTPESIAE---------------TPPARD-GSGGELYGYMSMDRPLSHC-----------------GRPYRRVS-----------------------GDGAQDLDRGLRKRTYSLTT----P------ARQRQVPQPSSASLDEYTLMRA----TFS-G---SSGRLCPSFPASSPK--VAYNPYPEDYGDI------EIGSHKSSSSN-----------------LGADDGYMPMTPGAALRS-GGPNSCKSDDYMP---MSP------TSVSAP-----KQILQPRL----------AAALPPSGAAVPAPPS----GVGRTF---PVNGGGYKASSP-AESSPE-----------------DSGYMRMWCGS------------KLSMENPDPKL-----LPNGDYLNMSPSEAG--------------------TAGTPP----D-FSAALR----------GGSEGLKGIPGHCYSSLPR--------SYKAPCSCS---------GD--NDQYVLMSS-----PVGRIL-----EEERLEPQATPGAGTFGAA-----------GGSHTQP----HHSAVPSSMRPSAIGGRPE-GFLGQR----CRAVRPTRLSL-------------------EG----------------------------LQTLPSMQEYP-------LPTEPKSPGEYINIDFGEAG---------TRLSPPAPPLLASAASSSSLLSASSPASSLG---SGTPGTSSDSR------------QRSPLSDYMNLDFSS------------PKSPKPSTRSG---------DTV----------GSMDGLLSPEASSPYPPLPPRPSTSPSSLQQ--PLPPAPGDLYRLPPASA-ATSQGPTAGSSMSSEP-------GDNGDYTEMAFGVAA----TPPQPIVAPPKPEGARVASP---------------------------TSGLK-----RLSLMD---------------QVSGVEAFLQVSQPPDPHRGAKVI------RADPQGGR---------RRHSSET---------FSST--------TTVTPVSP-----------------------------SFAHNSKRHNSASVENVSLRKSSEGSSTL----GGGDEPPTSPGQAQPLVAVPPVPQARPWNPGQPGALIGC-----------PGGSSSPMRRETSVGFQNG-LNYIAIDVRGEQGSL------------AQSQPQ----------PGDKNSWSRTRSLGGLLGTVGGSGASGVCGGPGTGALPSASTYASIDFLSHH-LKEATVVKE--------------------------------Mmus_IRS3/1-495                    --------MKP---------------------------------------------------------------------------------------------------------------------------------------------AGTGPTVSSGG------------------------------------------------------------------------------------ECTDVSLGSPFPWP-CLPDVRLCGHLR--------------------------------------------KQKSQRRRFFVLRA----------------DPPRLECYESEKKFLASGCR----------------PPRPRRTVSLEGACTI-SKRADARQRHLIVIYTSDSSLGVAAASE-AEQQAWYSALLEVR-ATAAAAAATAMG-------------------------------FSPQEAPESWIFA---------------------------PFQD----------------VWPVTLRSKGLGRA----QGLSSGSYRLCLGSGALSLLRKPG-SK--GSRDSRAT-----PPPVLRLSLLSVRRCGHA--DSFFFLELGRSAPIGPGELWLQAPDAVVAQSIHETVLAAMKRLGS------------------NAAGKAEPPPQG-------NPPKSVPAA-----------PTPTPYEIPAS--------------------------------------------------------------------------------------------AAQARSPSE--------------------------------------------------------RAK----Q--------DYF-----------------------------------------------KPLE-------------------------------------------------------------------------------------RMGSTHS-------------------------------Y----------------KGLDLGGNYITMGVR----------------------------------------------------------------------------------------------------------------------------------------------------------------------------------------------------------------NDYVH---MGG-------------------------------------------------------------------------------------------------------------------------------------------GEAGDYMWMAPPG----------------------LPPTPAR------------------------------------ADPN-----------------------KQLEDCESTEYVPMNR----FLPGPFYY---------------------------------------------------------------------------ELKARESELGH--------------------------------------------------------------------------PGAHCSL--------------RDRWRPTV-----------------AQPRSSQ--------------------------GSELSGDYMSIP----------------------------------------------------------------------------------------DYV--------------------------------------------------------------------------------------------------------------------------------------------------------------GTDSA------------RLGSLDS---------------------------------------------------------------------------------------------------------------------------------C-------------------------------LNYVDLDL-----------VP----------------------PLEV---------------------------PGAAPGKSPHSYASIKF----------------------------------------------Mmus_IRS4/1-1216                   --------MAS----CSFSGHQAL------------------------------------RRLRA-------------------------------------SAAAAASAA--------------------------------------LAAVATTPLLSSGTRTALI-------------------------GTGSSC----PGA-----------MWLS----------------------TATGSRSDSE--SEEEDLPVGDEVCKRGYLR--------------------------------------------KQKHGHRRYFVLKLETAD------------APARLEYYRNARKFRHSVRAAAAAAEAAASGAAVPALIPPRRVIILYQCFSV-SQRADARYRHLIALFTQDEYFAMVAENE-SEQESWYLLLSRLI-LESKRRRCGTLG-------------------------------ALPDGEPAALAAAA-AAEPP--------------------FYKD----------------VWQVVVKPRGLGHR----KEL-SGVFRLCLTDEEVVFVRLN--TE----------------VASVVVQLLSIRRCGHS--EQYFFLEVGRSTVIGPGELWMQVDDSVVAQNMHELFLEKMRALCA-----------------DEYRARCRSYSIS-----IGAHLLTLLST-RR------HLGLLPLEPGGW-----LRRYGLEQFC--------------------------------------------------------------------------------RLRAIR----------ERE-EML------FTRRF--ISPREPPPPFRRGRGHLP---------RAR------------------------RSRRAA----------SVPPSLFRRS----APSPGRIP-QPEDVPNDRAR---EASGSSSGNTEE--------KDKEGEEGNRGDCI-------------------PMNNWGSGNGRGSGGGRGSSG-QGSSSQGSGGR------QGSGGGQGSGGQGAGG---------------NQCSGN--GQGTAGGHGSGG--------------------GGHGSGGGQRPGDGHGSGGGKNSGSGK-------NSDDGDRGKSVKKRSYFGKF---------TQSKQQQT--LPPPPPPPPAAGATG-------GKGKSGGRF--RLYFCADRG-TKERKEAKEVRDMETSGGATRGPYRARAFD-----------------EDEDDPYVPMRPGVA------APLACSSDYMP---MAP------QNSSAS-----TKRHSRSP---------------------------------------FEDSRGYMMMFPRVSPPPPVPSA---------------------------------PKAPDTNKGDDSKDN----DSDSDYMFMAPGAGA--------------------IPKNPP--------------------NAQGGSSSK--SWSSYFSLPS--------PFQSSPLGQ---------SD--HSEYVPMLP-------GKFLGSGLHKEASFSQGTKNVSSKPST------------EASFSKPEDK------GSSAKPS--------DDVPPM----NKAKEPNHLSF-IAKG--------------------------------------------TQVKPKPLNPTQ------ERREAAGSRDYINIDFIKR----------ERLVLPS--------------SAQGLPDMRG---------VVTDP------------APTAFSGYLNVEFGV-------------PFPNPTIRLS---------DLLR---------------VLPGA---------------------NSIPLAGTRWPFP-------------GSAIGS--I-------VEAGEYIEVIFNPAM----TPAMSFAD---------------------------------------SAICYDAQTGQIYVVD-------------PFSECCMDVSLSPGRCSEPPPVARLR------REEAQ-ER---------RRPQSRSQS------LFAST--------RAAVSAFPTDSLDRDFPAASA-VIAAPAEAPLLAVSRALAVVSALAAAPSIGDVFAGFRAAAGVDSA-----------SARGFQPVAG----------------------------------AQAVREFQDLAAGWNPGALN----HRARGEDLA-----------AGAAAPPPPPRQIWVLRPQERAD-------------------------SEDDDDDDDDIYVRMDFARRDYRK---------------------------------------Ggal_IRS1/1-1178                   --------MAS----------------------------------------------------------------------------------------------------------------------------------------------------------------------------------------------------------------------------------------------------PTDNNEGFFSDVRKVGYLR--------------------------------------------KPKSMHKRFFVLRAASES------------GPARLEYYENEKKWRHKSGA-------------------PKRSIPLESCFNI-NKRADSKNKHLVALYTKDEHFAIAADSE-LEQESWYQALLQLH-NRAKGHHHLHHHHH------------HHHSDVTFGGS-----SVGLGEAGED-SYGE-VAPGP--------------------AFKE----------------VWQVILKPKGLGQT----KNL-IGIYRLCLTNKTISFVKLN--SD----------------AAAVVLQLLNIRRCGHS--ENFFFIEVGRSAVTGPGEFWMQVDDSVVAQNMHETILEAMRAMSE------------------EFRPRSKSQSSS-----NCSNPISVPLRSRH------HINNPPPSQVGL-----SRRSRTESVTATSPAGGGGG------------------------------------------------GMGG------------KPS-SFRVRASS----------DGEGTMS--------RPA---SVDGSPVSPSANRTHSH---------RHR------------------------GNSR------------LHPPLNHSRS----IPMPSSRC-SPSA--------TSPVSLSSSST---------------SGHGSTSDCL------------------FPRRSSASVSGSPSDGGFISSD-EYGSSPCDFRS----------SFRSVTPDSLGH---------------TPPARG---DEEL-NYICMGGKAASSCCSLAAPNGHFIPRTCHPQQQPRYP--STSCCPRAG------------SEDVADLDKAFRKRTHSAGT---SP----TISHQKTPSQSSVASIEEYTEMLP----SYPCG----GSRL--PSYRHSAF--VPTHSYPEECLEMHHLD---GSHHRTNSA------------------PHTDDGYMPMSPGVAPLP-SGGAAPKGGDYMP---MSP------KSVSAP-----QQIINPGR--------------------------------GGRHPPATVDSNGYMMMSPSGSYSPDSG---------------SAGYGKIWTNGAG-------HHPKLSVESNEGKLP----CGGGDYINMSPASGS--------------------TTSTPP----DCYFGAAGQPG----VEEAAAALHK--PIYSYFSLPR--------SFKHVHRRGGGP--AGEEGS--PQPRVALGS-------GRLLYA---AEDSSSST----SSDSLGGP-------------------GGPEGPASHSQPPR----KVD-TAVQTK----GRLARPTRLSL-GGPK--------------------------------------------ASTLPRAREQP----PLLLPPEPKSPGEYVNIEFVPGD----------------------------------KPPFPS---------AAPGL-------PRPP-GGEAAEEYMNMELGP----------PRARCPG----------------------------AFAAAAARPGRGAA----------PPGR------------DYVSMQL-----------GGSCSDCADSPSPSSPAPLLGYADVRAGRSAA--EKPPPAAAA----------SPELPRPP---------------------AELAA-APPRSSSLLG---------------GPGAGSAFTRVSLSPGRNQSAKVI------RADPQGGR---------RRHSSET---------FSST--------PSAARGAAG----------GG-GGGGGPGAPFPCGGAGGAEEVKRHSSASFENVWLRPAAG--------------------------------------------------------------EPPSASRGPGAALENG-LNYIDLDLVKDCSHRRHHLHPPAEGASGPGGKPPQPRS-----PRGSS-----------------------------HSSDDLSAYASISFQKREEP----------------------------------------Ggal_IRS4/1-1164                   --------MASGMNGPGGGGGAGAA------VRGGE----EEPGARHAG-----GGAVPQQREPG-------------------------------------AAGEGEPAAGDGGR------------------------CPSPQPHHLLLLLRRSPSASLCPPAEGP-------------------------AGRAA---------------------LG----------------------RGGQPPPAGRAAPPASSS-GGDDVRKCGYLR--------------------------------------------KQKHGHKRYFVLRADSPL------------APARLEYYDSEKKFKSSLRAGGGGAAPVC-------CPPPKRVIPLYQCFTV-SRRADAKHKHIIALYTKDEYFAMLAENE-AEQEAWYEAISELM-SQTKRGCLEQEDQ------------------------------AEQQVDEDDEHYGTSLRPGT--------------------VFKE----------------VWQVNVKPKGLGQT----KNL-TGVYRLCLSSKAIHLVKLN--SE----------------VPSVHLQLMNIRRCGHS--ENFFFIEVGRSASIGPGELWMQVDDSVVAQNMHETFLETMKALKA----------------FAEFRPRSKSQSSGG---GGGTNPISFITT-RR------HLGNLPPSQTGL-----QRRSRTESVVGGTPPT-------------------------------------------------------------------TKSSNSYRFRTSS----------EGEGTMT--------RPF--RSVTGSLIHLNTARMNLG---------RQE------------------------GSGRYVRA--------AFSSSYHTRS----ASLPVSH--FPST--------TSPISVSSS-----------------SGHGSASDML-------------------TRPSSSSVCGSPSDGGFISSD-EYGSSPGDFRYF---------RVRSNTPDSLGN---------------TPPIRE---ENCLSEYMSMSKQQADD----------------------------------------------NSRDDYMEAEKCFRKRTYSLTK----P---TSVAVQQKTTQ-TTALLDEDS-----------VG---NHGR---LLYSETSK-LKGNHELEYSDSNL------DSICNQSRS-------------------KARDDGYMPMMPGVA------SSLSSNSDYLP---MTP------KSMSVP-----KQINNSWS-------------------------------------PSQVDSRGYMMMFPKASSSPVRS-----------------------------------PLTGFISKGSNEK------IVNNEYMDMSPGNS---------------------APKHPG----DSNYIHTS-------------SASK--GFSSYFSLPR--------SFKALTGQN---------GD--HSEYVPMSS------PGKLLYG---GPENVKST----NSEVLS------------------------NGISKSPVAKG----SDE-GLVQ------NRATRPTRLPL-GTRG--------------------------------------------SNTIPRMYDRT-------VPPEPASPGEYINIDFNEK----------ASNTPYS-------------LSAEGSPSSLG---------SSSDH------------RQSPLSDYMSVDLDV-------------QSPKVAKELS---------NSLT-------DISIYASSSIPRN-------------------------QQNPDYARLSF-----------GTACVSAAN-------NRTDDYTEMTFNMAA----TPPRPFAA-ESDDGVKMDSP---------------------------SSIVN-----RLCIVD---------------RYAGSSSFSVPSA--EPPMGPKVI------RADPQ-GR---------RRHSSET---------FSSA--------GTVTTSSS-----------------------------FFTDSSKRHSSASFDNVWLKPDESI----------------------------------------------------------SDGQESKMSRDTSTGFQNG-LNYIALNLCDDPISC------------EASTGTPTCHL------------------------------------QNGTSSLDSGAYVSIDFTRSDGLKCNAARKD--------------------------------Locu_irs1/1-1079                   --------MAS----------------------------------------------------------------------------------------------------------------------------------------------------------------------------------------------------------------------------------------------------PTTDHD-CFSDVKKVGYLR--------------------------------------------KPKSMHKRFFVLRTASVS------------GPARLEYYENEKKWRHKSGA-------------------PKRSIALESCFNI-NKRADSKNKYLVALYTKDEYFAIAADSE-PEQDLWYQALVELH-NRGKIHD-----------------------------------SAGGSGFGED-TYGE-SRPGP--------------------AFKE----------------VWQVILKPKGLGQT----KNL-IGIYRLCLTNKTISFVKLN--SD----------------AAAVVLQLMNIRRCGHS--ENFFFIEVGRSAVTGPGEFWMQVDDSVVAQNMHETILEAMKAMSE------------------EFRPRSKSQSSS-----NCSNPISVPLR-RH------HHNNPPPSQVGL-----SRRSRTESITATSPAGPGK-----------------------------------------------------------------HGN-SFRVRASS----------DGEGTMS--------RPA---SVDGSPVSPSTTRTHSH---------RHR------------------------GSSR------------LHPPLNHSRS----IPMPSSRC-SPSA--------TSPVSLSSSST---------------SGHGSTSDCL------------------YPRRSSASVSGSPSDGGFISSD-EYGSSPCDFRN----------SFRSVTPDSLGH---------------TPPARE----EELNNYISMVKPN-------LLPNGH------HNRSHQR-------CTP--------------SKVEEAELEKGFRKRTHSSGTS--SP----TISHQKTPSQSSTASLEEYTEMMP----TYQC-----------RLYRHSAF--VPTHSYPEECLDLHIEG----------SRT-----------------NHTDDGYMPMSPGVAPVP------AKTDDYMP---MSP------KSVSAP-----QQIINPRQ-------------------------------------HPRVDSNGYMMMSPSGSCSPD-----------------NTNYGKIWTNGA---------NPKLSVESNEGK------VSCGDYINMSPASGS--------------------TTSTPP----DCYFNPVE-------------EPPK--PLYSYFSLPR--------SFKHAHRKA---------AE--SQLRISVSS-------GRLVY----GEDSSSST----SSDSLGG-----------------------QENGQQAVKPK----KAD-EYAQAK----GRLARPTRLSLDNNNK--------------------------------------------ASTLPRTREHP-------FPPEPKSPGEYVNIEFNDKSF-------------------------SASLASLFSPVCAG---------SSPAR------------PEQNSSEYMNMDLGAHGSKPSYPSKPTADSTGCAADYA---------------------VTAAAAAAVPAAAAC----------RASRG-------QQSCDYVSMQL-----------SAPSAGCAE-------APMLSYTEMGTGAAK----TPTQSLSP----------HPEMP-PL---------------------SSVSS-AA--CSSLMS---------------QMSGTSAFTRVNSSPNRIQGAKVI------RADPQ-GR---------RRHSSET---------FSST--------PAASGGVA----------------------------LLCGDDVKRHSSASFENVWLKQGESYV---------------------------------------------C---------LREEQQPGVSSLNSATPFENG-LNYIDLDLVKDFNNQ-----------EWTSLQPKSSNQ-----PCGST------------------------------SGDDLSAYASISFQKPDEIRT--------------------------------------Locu_irs2b/1-1069                  --------MAS--------------------------------------------------------------------------------------------------------------------------------------------SPTTGHLFS-----------------------------------------------------------------------------------------------NVHVNN-NNNLIKKCGYLK--------------------------------------------KQKHGHKRFFVLKEQSDG------------FPARLEYYENEKKWKNKSA--------------------AKRVIPLDSCLNI-NKRADAKHKHLIALYTKDEYFAVAAENE-QEQESWYIVLTELM-NEGKVYDGSASTS-----------------------------TSSLSGF-EEANYGLITPASA--------------------VYKE----------------VWQVNLKSKGLGQS----KNL-TGVYRLCLSSRTISFVKLN--SE----------------APSVILQLMNIRRCGHS--DNFFFIEVGRSASTGPGELWMQADDSVVAQNIHETILEAMKAMKE----------------LSEFRPRSKSQSSG-------TNPISVPT--RR------HLNNLPPSQTGL-----PRRSRTDSMAATSPAN--------------------------------------------------------------------KFT-SCRIRTAS----------EGDGTMS--------RPV---SVNGSPISPSAIRTHLS---------RSNTVTT-----------------------RPSRM-------FQTSSLQHSKS----MSMPVSHS-PPSA--------TSPVSLSSS-----------------SGHGSASDTI-----------------QRPSSGSASVSGSPSDGGFMSFD-DYGSSPGDMKHCL--------GNRSNTPESIAD---------------TPPSRE---GSDLYGYMTMERPVNSIF----------------NLNGRRFC-----------------------KDDILDSEKAYRKRTYSLTT----P------PQQRATSQVSSASLDEYTLMRA----TYTNG---SSGR---SSYAASPK--VTYTPYPEDYGDI------EIGSHKSSSS------------------NLGDDGYMPMTPGVAPQA------SKKENYMP---MSP------TSVSAP-----KQIINPRS-------------------------------------HPETNGNRYRTNSP-GSCSLD-----------------DNGYMRMWCGS------------KLSVESSDGK------LTNGDYMNMSPIDHC--------------------VSLTPP----DYFLSSLT------------NESAR--PSYSFNSLPR--------SYKSQGSKN---------GD--SDQYVVMNL-----QNQKII-----EESNYCST----ASTAAVV-----------------------NHFAPSPVRQS----RNE--NLLQR----GRASRPTRLSL-------------------DT----------------------------LRTLPSMNEHP-------LPAEPKSPGEYINIDFSET----------TRYSPPS-------------LSTESPASSLG---------STNDQ------------RRSPLSDYMNIDLNA-------------QSPKSVEAPA---------DTMNV-------MSALSCSIQPDA-----------------------------EYLKTQI-----------ASVCLSGST---------KDDYTEMTFGVTN----TPPQPISQ--KSEKAQSMSP---------------------------TSGVK-----RLNLME---------------QVSGVEAFLLPSTPPDPDRGAKVI------RADPQ-GR---------RRHSSET---------FSST--------TTVTPVFP-----------------------------SFAHDPKRHSSASVENVSLRKSEGT----------------------------------------------------------DEEYGSPMCRETSAGFQNG-LNYIALDLMDENLGNC-----------ETLVKLKTARYL-----------------------------------KGGISGLDASPYTSLGFLKET----ATAVKG--------------------------------Locu_irs4a/1-1107                  --------MLLLETQQRAIGSKTTV------TTNGDTSVGEIPSSLINI-----GNVQYQQHLPL-------------------------------------SFHHSSQQAQQQ-----------------------------YSEENLQDELLRKPSLSLVQEEVAV-------------------------GTTAA--------------------------------------------------PVAAAAGAAARSD-GLDDVRKCGYLR--------------------------------------------KQKHGHKRFFVLRGQSHL------------GPSRLEYYDSEKKFRNSTRSTATA-------------CPPKRVIPLYQCFTV-NKRADAKSKHLIALYTKDEYFAMVAENE-QDQEDWYAAISDLM-NEGKRGHLDSEE--------------------------------------LDDGYGT-VTPGT--------------------VFKE----------------VWQVNVKPKGLGQT----KNL-TGVYRLCLSSKSIHLVKLN--SE----------------TPCVNLQLMNIRRCGHS--ESFFFIEVGRSSSIGPGEIWMQVDDSVVAQNMHETILETMKALKA----------------FAEFRPRSKSQSSS-------SNPLAFITT-RR------HLGNLPPSQTGL-----QRRSRTETIVGTPPAS--------------------------------------------------------------------KSG-GYRFRTSS----------EGEGTMS--------RPF--RSVTGSLIHLNAARMNLG---------RQEG-----------------------GGGRYVRA--------AAGAPCHMRS----ASLPVSH--FPST--------TSPVSVSSS-----------------SGHGSASDTL-------------------TRPSSASICGSPSDGGFNSSD-EYGSSPGDFRYF---------RVRSNTPDSLGN---------------TPPIRE---ENSLNDYMAMDRHREGL-------------------GCSEGP-----------------------REDCVEVDKGFRKQAHSLSR----PGSSSGVAVYQKMTQ-TTSSLDEPCAEAS------LVG---SSGH---RACSVSPK--SGFCPYPEDYYQA------KPPAYQHQG-------------------NPKDDGYMPMMPGVVP--------KRSADYVP---MQP------KTSSAP-----QQIVSPRC-------------------------------------SQYVDSEGYMMMLPSGGYSPVQTC----------------------------------------PQSSNGR------VSNGPYMDMSQNGAPTHRK----------RSNDCHCAQTPP----D---------------------LPK--SYSSYFSLPR--------SYKAPLRES---------RE--SDEYVPMCS------PAKPAYAV-----------IECSRRVVP------------------------SGPSPTPGMHN----GCQ-ASVGGD----GRAVRPTRLSL-ERHN--------------------------------------------------FHSSL-------IVCEPPSPGEYINIDYEQK----------ALHTPCS-------------LSAEGSPSSLG---------SSSEQ------------RRSPLPDYMSLDLDN-------------AFPKGC-----------------------------SPQPMPVAPR------------------------NPTDYSLPQA-----------STPSS-----------GRTDDYTEMTFNLGG----EP---------------SSP---------------------------SALVK-----QLRIIE---------------PYPALPAL-------SPPVEPKVI------RADPQ-GR---------RRHSSET---------FSSS--------PSAPSSAPQ-------------------------SSALLMDGSKRHSSASFDSVWLRMEGTGSLEVA-------------------------------------GDVGC-------------SSSSRMCRNTSAGYQNG-LNYIALDLREDPQGCCDLV--------SPSVPPPP--------P-----------------------------------LAESGAYARIKFPKSDGLTAASKG----------------------------------Cint_LOC100185541_irs1-like/1-276  --------MA--------------------------------------------------------------------------------------------------------------------------------------------------PEPD--------------------------------------------------------------------------------------------------YSA-MSTDIVLKGTLR--------------------------------------------KAKTWNKRFFVLRDG---------------NPPKLEYYENERKWKVNK---------------------PKRQISLGNPWNI-DKKKDTKHEFLIVIFTKDEYFTMAAESA-DVQQTWISALLKTV-RPAAG--------------------------------------------------------LT--------------------MFKY----------------IWQVVLENKELEMSSS-NLSL-HGQHRICLTHDSMVFVACNDRCN----------------NTPAEILITNIRLCGHK--ENMFFIEPGRASGIGNGKLWMDVGDDAMAAQMHTTILNVMYALRQEE-------------ASCSYRGRSNSSGS---------NSSRTRTT-RT------H-HNPPPSQIGMGKISSAKRNRCDSL--------------------------------------------------------------------------------------------------------------------------------------------------------------------------------------------------------------------------------------------------------------------------------------------------------------------------------------------------------------------------------------------------------------------------------------------------------------------------------------------------------------------------------------------------------------------------------------------------------------------------------------------------------------------------------------------------------------------------------------------------------------------------------------------------------------------------------------------------------------------------------------------------------------------------------------------------------------------------------------------------------------------------------------------------------------------------------------------------------------------------------------------------------------------------------------------------------------------------------------------------------------------------------------------------------------------------------------------------------------------------------------------------------------------------------------------------------------------------------------------------------------------------------------------------------------------------------------------------------------------------------------------------------------------------------------------------------------------------------------------------Blan_BL06168_IRS1_cuf12/1-1050     MRRGAPCRMSRDLRSYRGQISADGS------VRSADSSCGVCRG--------------------------------------------------------------------------------------------------------------------------------------------------------------------------------------------------------------------------SRHDVCWRGHARGR-------AETRLPAEAQGAS-------CGPGC----------ESATMKKRYFVLHAKSSA------------GAARLEYFDNEKKWRHGAG--------------------PKRTIFLHHCFNI-NKKEDGKHKLMIVLYTRDECFSLVADSA-DEHEGWLAVLRDLH-ESGSQEG---------------------------------------------------FISKQ--------------------NFEH----------------VWQVTLKPKGLGSS----KNL-TGQYRLCLTANSITLLKMS--SE----------------EPAMDFQLTQIRRCGHV--ESFFLMEVGRSAVTGAGELWMQVEDTLVAQNMHEAILSSMKAISS----------------DDEFRPRSRSSGSS--------NPLPVPAG-RR------MTGTAPTMALSHT-LPTRSRMRCDSLP-TAPGRGGA-----------------------------------------------------------------------RYRTTS----------EGEMKTN------------------SPVSPSRNRTRLS---------AIHQRSS----------------------PRSV----------LGAHGMFNRS----VSAA-------------------------T--------------------GFTSDQY-----------------SHPLPISRGLMEYTSDGSTVSMD-EYDSSPASSDHYL---QRYVPSGRSITPDSPSH---------------TPIREE-GG---GDGYVPMAPAFPGC-----------------------------------------------------------------------YP-------TTRSAPQ--------------------DVG--------------------YPTRSTPQEGSYMPMSGGIGRGSPTTLS-------------------SRLEDGYMPMSPGTPP---TMTDMRLDESYMS---MSP------SSVSAP-----RQIASRRP----------------------------------------ANGDPYVTMSP-GSRSPD-----------------HPEYMQIWANQ------------RPDNRQNNNN------NDDSSYMSMSPVAQALP------------------NTTIP-----DTYIVYSP----------------------SAESSPP---------VTTVIEKG-------QMEA--TDGYVHMEM-----QP--------------RRT----TNTAED----DNYMAMDFSAK---------ESERLSSSLPAE-------HRLSSS---PGGTVRPTTLRL-TNQHEYINVAPTAAPKDLDGYAVMDYISGDGGVASSDESRTSAADGKTSTSESGIYSGE-------ESGRPRSPGEYVNIDYSTK----------GADG-ASG-------------------------------------------------PEGDGSEYMNLTYPS-----------EKPSPK----------------------------------------------------------------IHVSDYSLMAP-----------VSIA------------------------------ERPGK-----------GAGK----------------------------PGLLK-----QDSLTS---------------EDEGLSSQGVIRHSPVFSTSADISAVKNS-KDSPEGGASPVSGSALAASPVSGSAL------AASPVSGSA----LAVSPVSGSALA---VSPASG-------------SALAASPVSVSVSGGSYENVSLGSQKGGAAGRT-----------------------------------------------------QSQSSQPNSRHSSVSSEKE-LNYISLDLPSDEGEG------GA-AGDD-NPVAKATRS-----PRSSV--------------------------S-SEDKADENPYASIDFTKSEGLRNTSPKYHDRDG-----------------------RF---Blan_BL20922_IRS-like_partial/1-225 --------------------------------------------------------------------------------------------------------------------------------------------------------------------------------------------------------------------------------------------------------------------------------------------------------------------------------------------------------------------------------------------------------------------------------------------------------------------------------------------------------------------------------------------------EH----------------VWQVTLKPKGLGSS----KNL-TGQYRLCLTANSITLLKMS--SE----------------EPAMDFQLTQIRRCGHV--ESFFLMEVGRSAVTGAGELWMQVEDTLVAQNMHEAILSSMKAISS----------------DDEFRPRSRSSGSS--------NPLPVPAG-RR------MTGTAPTMALSHT-LPTRSRMRCDSLP-TAPGRGGA-----------------------------------------------------------------------RYRTTS----------EGEMKTN------------------SPVSPSRNRTRLS---------AIHQRSS----------------------PRSV----------LGAHGMFNRS----VSAAGSLSQSPIS--------GSPLR---------------------------------------------------------------------------------------------------------------------------------------------------------------------------------------------------------------------------------------------------------------------------------------------------------------------------------------------------------------------------------------------------------------------------------------------------------------------------------------------------------------------------------------------------------------------------------------------------------------------------------------------------------------------------------------------------------------------------------------------------------------------------------------------------------------------------------------------------------------------------------------------------------------------------------------------------------------------------------------------------------------------------------------------------------------------------------------------------------------------------------------------------------------------------------------------------------------------------------------------------------------------------------------------------------------------------------------------------------------------------------------------------------------------------------------------------------------------------------------------------------------Bflo_BRAFLDRAFT_124878/1-1203      MRPGAPCRMSRDLRSYRAQISAGDR------VSSPDRSCGVCRGSRHDVCWRGHSRGRAETRIPA---ETQGG-----------------------------SYGPGCESARGEGEGGYGTGRGASLLEGRVRVGTVRAGVRRKGLGQSLPWSEERAKVSTGQSMRGGSGWVRYGPEYEGWVR---------VGTSKARVQVHPNGASGSVLARVRLYWTGCKYGQGVNGRKGVREGPGLVRTRVRLRGEAMVSMAQSLRE-EPESAREMVTVC----------------MGLGAV----------GR----------SGLTMKKRYFVLHAKSSA------------GAARLEYFDNEKKWRHGAA--------------------PKRTIFLHHCFNI-NKKDDGKHKHMIVLYTRDECFSLVADTA-DEHDTWLAVLKDLH-ESGSQEG---------------------------------------------------FISKQ--------------------NFEH----------------VWQVTLKPKGLGSS----KNL-TGQYRLCLTANSITLLKMN--SE----------------EPAMDFQLTQIRRCGHV--ESFFLMEVGRSAVTGAGELWMQVEDTLVAQNMHEAILSSMKAISS----------------DDEFRPRSRSSGSS--------NPLPVPAG-RR------MTGTVPTMALSHT-LPTRSRMRCDSLP-TAPGRGGA-----------------------------------------------------------------------RYRTTS----------EGEMKTN------------------SPVSPSRNRTRLS---------AIHQRSS----------------------PRSV----------IGSHGMFNRS----VSAAGSLSQSPIS--------GSPLSPSPT--------------------GFASDQY-----------------AHPLPISRGLMEYTSDGSTVSMD-EYDSSPASSDHYL---HKYTSLPTVSTLTTYSK---------------GGCNQG-GGCSQGGGYVPMAPAFPGG-----------------------------------------------------------------------YP--------TRSAPQ--------------------DVG--------------------YPTRSTPQEGSYMPMSGGIGRGSPTTLS-------------------SRLEDGYMPMSPGTPP---TTTDMRLDESYMS---MSP------SSVSAP-----RQIVTRRP----------------------------------------ANGDPYVTMSP-GSRSPD-----------------HAEYMQIWANQ------------RPDNRQNNNN------NDDSSYMSMSPVAQALP------------------NTS-------NTYIVYSP----------------------SAESSPP----------AAVIEKG-------QMEA--TDGYVHMEM-----QPRR------TGTETARRT----GQTTQD----DNYMAMDFGAKGSERL---SASERLSSSLPAE-------HHLSSS---PGGTNRPTTLRL-TNQHEYINVTPTAAPN--DGYAVMDYISGDGGVASSDESRTSAADGKTSTSESGIYSGE-------ESGRPRSPGEYVNIDYSTK----------GADGPGS---------------------------------------------------EGDGSEYMNLTYPS-----------EKPSPK----------------------------------------------------------------IHVSDYSLMAP-----------VSIA------------------------------ERPEK-----------GAGKS---------------------------PGLLK-----QDSLTS---------------EDEGLSSQGVIRHSPVFSTSADISVVKNS-KESPEGGASPVTV---------ETAA------GASPVT-----------------------------------------AVTGASP----VSGGSYENVSLGSRQGG--GLT-----------------------------------------------------QS--SQPGSRHSSVSSEKE-LNYISLDLPSDEGEG------AA-GDD--NPVAKATRS-----PRSSV--------------------------S-SEDKADENPYASIDFTKSEGLRNTSPKYHDRDG-----------------------RF---Bflo_BRAFLDRAFT_132514/1-704       --------------------------------------------------------------------------------------------------------------------------------------------------------------------------------------------------------------------------------------------------------------MMSAGGD-TPEDVRKHGYLR----------KLKVGPTGQGAS-------LPEGR----------GGWTMKKRYFVLHAKSSA------------GAARLEYFDNEKKWRHGAA--------------------PKRTIFLHHCFNI-NKKDDGKHKHMIVLYTRDECFSLVADTA-DEHDTWLAVLKDLH-ESGSQEG---------------------------------------------------FISKQ--------------------NFEH----------------VWQVTLKPKGLGSS----KNL-TGQYRLCLTANSITLLKMN--SE----------------EPAMDFQLTQIRRCGHV--ESFFLMEVGRSAVTGAGELWMQVEDTLVAQNMHEAILSSMKAISS----------------DDEFRPRSRSSGSS--------NPLPVPAG-RR------MTGTVPTMALSHT-LPTRSRMRCDSLP-TAPGRGGAR----YRTTSEGE---------------------------------------------------MKTNSPVRYRTTS----------EGEMKTN------------------SPVSPSRNRTRLS---------AIHQRSS----------------------PRSV----------IGSHGMFNRS----VSAAGSLSQSPIS--------GSPLSPSPT--------------------GFASDQY-----------------AHPLPISRGLMEYTSDGSTVSMD-E----------------RYVPSGRSLTPDSPSH---------------TPIREE-GG---GDGYVPMAPAFTGG-----------------------------------------------------------------------YP--------TRSAPQ--------------------DVG--------------------YPTRSTPQEGSYMPMSGGIGRGSPTTLS-------------------SRLEDGYMPMSAGDPAHDNTTTDMRLDESYMS---MSP------SSVSAP-----RQIVTRRP----------------------------------------ANGDPYVTMSP-GSRSPD-----------------HAEYMQIWANQ------------RPDNRQNNNN------NDDSSYMSMSPVAQALP------------------NTSIP-----DTYIVYSP----------------------SAESSPP----------AAVIEKG-------QMEA--TDGYVHMEM-----QPRR------TGTETARRT----GQTTQD----DNYMAMDFGAKGSERL---SASERLSSSLPAE-------HHLSSS---PGGTNRPTTLR-----------------------------------------------------------------------------------------------------------------------------------------------------------------------------------------------------------------------------------------------------------------------------------------------------------------------------------------------------------------------------------------------------------------------------------------------------------------------------------------------------------------------------------------------------------------------------------------------------------------------------------------------------------------------------------------------------------------------------------------------------------------Bbel_LOC109465793/1-966            --------------------------------------------------------------------------------------------------------------------------------------------------------------------------------------------------------------------------------------------------------------MMSGGGD-TPEDVRKHGYLK--------------------------------------------KLKTMKKRYFVLHAKSSA------------GAARLEYFDNEKKWRHGAA--------------------PKRTIFLHHCFNI-NRKEDGKHKHMIVLYTRDECFSLVADTA-DEHDVWLAVLKELH-ESGSQEG---------------------------------------------------FISKQ--------------------NFEH----------------VWQVTLKPKGLGSS----KNL-TGQYRLCLTANSITLLKMN--SE----------------EPAMDFQLTQIRRCGHV--ESFFLMEVGRSAVTGAGELWMQVEDTLVAQNMHEAILSSMKAISS----------------DDEFRPRSRSSGSS--------NPLPVPAG-RR------MTGTVPTMPLSHT-LPTRSRMRCDSLP-TAPGRGGA-----------------------------------------------------------------------RYRTTS----------EGEMKTN------------------SPVSPSRNRTRLS---------AIHQRSS----------------------PRSV----------IASHGMFNRS----VSAAGSLSQSPIS--------GSPLSPSPT--------------------GFASDQY-----------------SHPLPISRGLMEYTSDGSTVSMD-EYDSSPASSDHYL----QXVPRGSCLPXTLYTRL---GR--------LQPGLQR-EG---GTDMCPMAPAFPGG-----------------------------------------------------------------------YP--------SRSAPQ--------------------DVG--------------------YPARSTPQDGSYMPMSGAIGRGSPTTLS-------------------SRLEDGYMPMSPGTPP---TTTDMRLDESYMS---MSP------SSVSAP-----RQILHRRH----------------------------------------GNGDPYVTMSP-GSRSPD-----------------HPEYMQIWANQ---------NQPRPDNRQNNNN------NDDSSYMSMSPVAQALP------------------NTS-------NTYIVYSP----------------------SSDSPPP----------AAVIEKG-------QMEA--SDGYVHMEM-----QPRR------PNNTSA---------TTEE----DNYMAMDFSAK--------SESERLSSSLPAE-------HRLSSS---PGGTVRPTTLRL-TNQHEYINVTPTAAS---EGYAAMDYISGDGGVASSDESRTSAADGKTSTSESGIYSGE-------ESSRPRSPGEYVNIDYSTK----------GTDGPGSG-------------------------------------------------AEGDGSEYMNLTYPS-----------EKPSPK----------------------------------------------------------------IHVSDYSLMAP-----------VTIA------------------------------EKP--------------VGKP---------------------------PGLLK-----QDSLTS---------------EDEGLSSQGVIRHSPVFSTSADISVVKNSTKESPEGGASPASV-------------------------------------------------------------------------------SGSYENVSLGSQKGGACSLA-----------------------------------------------------QSQ-SQPGSRHSSVSSEKE-LNYISLDLPSDEGEG------VA-AGDEQHPVAKATRS-----PRSSV--------------------------S-SEDKADENPYASIDFTKSEGLRNTSPKYHDRDG-----------------------RF---Spur_SPU_011063_IRS/1-1015         --------MSS-------------------------------------------------------------------------------------------------------------------------------------------------QKENVPI--------------------------------------------------------------------------------------------PCGVEL-SIGDIEKSGYLRKLKFHDVVFMLTSLAP--PGNSQCVALIGLPAGFANLHCACYVDPLETMRKKYFVLRSESIT------------GPSRLEYYDNEKKFRLGGE--------------------AKRTVPLSACFNI-NKKSDAKHKHAIALYTRDDCFSLVADDE-ESKQEWLRVLLEQQ-RAGGD-----------------------------------------------------GKPVP--------------------AFEH----------------VWQVTVKPKGLGSS----RQL-SGIYRLCLTTSTISLVRMN--SE----------------NEGLEFSLSAIRRCGHS--DCFFFMEVGRQSVTGPGELWMQVEDTVIAQSIHEASLEAMRSVKQ----------------QEEMRPRSHSSTMSSGNPGSDSKSHSASGS-RR------QSNTLGP----------SWRTRCDSMPVPNRAMPGSRSGSRSRGNSEGEDGQSKEEHAIASSSSPVSNL----TNSVMRPRTCSEGGGGHSTVPRARSSLLKFGNKLRPRTSS----------EGEKGMK------------------APDSPTKTRGRFSI-------PSLTSRSS-------------------LSSGP------------IRPLSVH-RM----VAQPMSNS-PPIP--------NSPLSESPQSYLDE--------RYQSEGHSSDDHYFT----------------------------------------RFGSS----------------GGRSRTPDSPSH---------------VSIREE----------------------------------------------------------------------------------------------------------------------------------------------------------------------------------------------------SQEANDEYMSMSPSAA----SKVVVDEPDSYMA---MTP------SNRSRP-----SSVNLAPS----------SS---PASHITPSPSQRLAPSSGSGLKAPSNEDEGYMMMGPSPGSKRATPITIT-------------------------------------PQAGSTS------AEPSTYMLMNPSA----------------------NRPSPS-------------------------SCNS--PSLAWAPAPM--------SISPIGGGGGGAFTTNSLGK--SPDKKHRNSA--GQEDASLLKV---GSSSHQRS----HSEPVV----DTYCNMEFEAKGSQVSHK-----------------ACENNNINKKDV--------------TQRHS------------M------------------------------------------------PPMEP----KYINVNYEEK----------NEQLESS------------VQVTVDAPDCIEGNAMVRNIVESANKG-LDVVKNGSP-ESGVNGEYMNMSFGK-----------GRNGPKGITDLSKRQVPPLLLDPMTH--------------------------------------------PDSHDYINVQ----------------------------------TDIREGVVK---------------------SSP---------------------------------------------------------------------RHSPVSPPSPSWLQ-----RPVAE---Q-------LLRKSLEDL-------SFSQLSNSI----------------------------------------------PSRGSANSLTGVGSNRNSDTMSPGA------------------------------------------L----------SSHHSSRRSSQSSLSGERE-LNYIDVDIGQPASEK------SG-SS-TPTLREKRSRS-----PFRRI--------------------------PNEDKNCDQVNYSTIDFTKSEGLRSVTSTL--RD--------------------A---RY---Skow_LOC102809894_IRS1b-like/1-911 M----PA-MSS-------------------------------------------------------------------------------------------------------------------------------------------------PQIERAKTAHRE------------------------------------------------------------------------------EEQHDIQ--QQHDTMVTEKDIKLQGYLK--------------------------------------------KLKTMKKKYFVLRAQTKS------------GPARLEYYDSEKKFRANAA--------------------PKRSVVISTCFNI-NRKTDSKHKYALALYTRDDCFTILMETS-EELEEWLMEMFKLQ-QSGSKEG---------------------------------------------------LPLQP--------------------AFEH----------------VWQVTLKPRGLGNS----KSL-TGSYRLCLTSTTVNLVKMN--VE----------------NEDRVFPLSSIRRCGHS--DCFFFMEVGRSTVTGAGELWMQVEDTVIAQNMHESILSAMKNMSH---------------LDDDMRPRSRSTGAST---PDVTKPITVP---RR--------PGTPGSMASLN----RSRTRCDSMPAHSRTLGPGGKMDDSKGD-------------------------------------------------RSPGSIFKLGGRFRPRTSS----------EGDKHYR----------------SNRPDSPSKTRSRTI---------GSQGRSN-------------------LGSSR------------LRPYTPISRS----VSQPMSNS-PPVS--------NSPLSESPQ-----------------QSSSFPDEHF-------------------FRHLSNATVISPD--GSRTPE-EYGSSPVDQHYHR--------YGRVSTPDSPSR---------------TPIREE--SVESGDDYMPMS------------------------------------------------------------------------------P---SAVSQGQAIPK-----------------------------------------------------------------------V------------------AASKDGYMSMDP-------RTKSISTPESYVN---ISP---------------------------------------------------------GNSTPLTGDIDIAYMSMSPPVTATSYIKECST----------N--------------------QESGSKTEPSNVKLA----THDEGYMDMAPVGDAMPK-------IEPLQI----KTVTPTLTPIDSYMGFTP------------GSIGN-----DTHNAPV--------------------------TQ--SPSYTNVDI------KPKVVYA---TGASLKTP----KQLPTL----DTYMPMSYESQSVEK--------------------QVE-ESANQQTM--------------TGKH-----------------------------------------------------------------------DYINIDLSK--------------------------------------------------------------------KKQNLGDYAEMDIGQ-----------GHSSQKTGLKSSRPQRLPLPADRLEKVELGARKSPKLSPSLSPRQPK-----------------DFKKMSHHPPEYINIEI-----------GT-----------------------------------------------------------------------------------------------------------------------------------------KDKMRKDLE-----------SKNRAERTI-------KMSVPRPKD-IPHPAVTSTHFQ---------------------------------STATSSDNYLNVEVPNTSSG---------------------------------------------------------------SPGKQNTSPQPQAAVLNYISLDLGPSEDVE----------ENGQNPSPHSPRS-----PLRR---------------------------TSESDDKTESAYASIDFTKSEVLKNISTSLRE-------------------------TRF---Cgig_IRS1/1-1383                   M-------MEN-----------------------------------------------------------FD---------------------------------------------------------------------------------SEDDNISRASSN----------------------------------------------------RW-----------------------------------SVMAFEL-PGSDILKTGYLK--------------------------------------------KLKTKKDKFFVLRSTSSS------------GPARLEYHDSEKKFKAGQL--------------------PKRQIHLHKCFNI-NKKSDTRQKNCIALYLVDECFAVIVKDA-AEMQVWLDLMLEHQ-YEYLTDEQLPYPH------------------------------YGYWEISLTLLYPVTLKPLS----VICRRKYIGYSI----SYQD---------------YIWQVEIKPKGLGVS----KHL-CGGYRFCLQD-TVCFVRNN--SD----------------KVDFEIQVI----CVHG--RVVRTVAALCQKRIGSRGLEFKA-----------------------------------PAMREPFRSRSNTSSSGR-----------------------------------------IRDAEIESI-------------------------------------------------------------------------------ESRSREGS----------HGQSSLRNK-KKKVPRPGSALSPEIPPIMPQISTSPSSTHKVTSDSFRERGFSNSMEQQKARHDSFTNKKPEIYGSSPGGDLTIVKHLDVKTPNSLETTN-SYLVMSPTQRSVSPLRDPQE----RSGSSASEHEPMPKMETGYMDMKPGSVGSGGAS--------------------TDPGYIDMSLSTPPADKGAISQM-NSGYIPMGTGHAV--------RLTASAPIPIRQPKEPGYMEMGPSSQPLPQIKE---GGSGEAYLPMTPSANSPL-------------------------------PGTDTLRPAK-MICYLSDDSMSGD--LPKRSFSLGS---RP---NTKTMRHHTSHVEPKTKEEAT----------------DNGRWLSAPHLIAQKKAQASHSYMNSESSL------ASSPCQSLFSEDSMMEMEYRP-------RATSESYRPRTSSVGKIL--SQSRQRSSSYGQQSKLAQLAHDVRRKVGSF-----ESVRNSAI-------------------------------DKQLFHRSSNDSIGHLSFSSKASSSES------------------------------------LRLSSRNSEYVDMHLDK---SNDTGYIDMSIGTPKSTKSSACHSRSSSNQS----LSSSPAI--VNSFGIKQEPIHNVKIIKSSDGNSQKS-SSLNVSSRSSI-------ASKSPCGSG-------RESE--DESYVPYAPGISGDSQGQ-----ESRSGSMSDKKTSSRSNSLSS---DRRPGSRSGSFGSEKNPDSRSNSFGSRPGPRSGSFKAD-----------PKTHKSSRLGR-HSPKT-------------------------------------------SLVSPEAQKSGDR-----KAAKQSDDNQYIDYEPGSVDIY------VKTHNPQAG-----------SKDGECNTTIVS--------RSTVQK------------PNAQVPQY-SMSTDPLFSAMLSLKQKGVESEKSESEMV---------------KNSEANSQGKEDTTQPKT-------------------------MSSESYSYMEYA--------PDTSITVDSTF----KSPEVARSYFSHEASSKEENENKAPKNIAE----KEDQTGSPKLPSSSQPESANDGEGRKRKTSRKESGEAVVRQVMPMKTVLLDNSEEDDGYVGLDFGDNKRGTDS----QHLSRPPLRVNIT---EGTSVDGD---------LYMRQNSRQKYSPVNNGAKFNSVTPQTGMKKKTSTSSLQD--------------------------QDTLFKEDKKETEKSCESLNLKSPIPLSVTSSQELQKQNSMPCMAMESDPCNNIKREEDLI--------NRRSCSDLASEYEEMSLPAKGSKSGSTQQLSNEPA-LNYAKLDL-------------------GSCEEIPADQR-----PRQTLHPS-----------------------SPDETGPPVQGYAEIDFEMSDNLKNARSKEKQ------------------------PVKFSIELgig_LotgiG137373_irs1/1-269       --------MSD-------------------------------------------------------------------------------------------------------------------------------------------------------------------------------------------------------------------------------------------FEALPVHHGQRTFER-PGSDIKKKGSLR--------------------------------------------KLKTMKKKFFVLRGTSSS------------GPARLEYYDSEKKFNLNHA--------------------PKRSIDLHTCFNI-NKRVDRKHNYAIALYTKDDCFTVLAENK-AEQDDWLSVLLEYQ-LQYVYHDENREHF-------------GELLLYSCSMREPS--LSNLSNVITHLYIMS-VGNKK--------------------IVAH----------------VWQVNIKSKGLALSPE-HKHL-RGCHRLCLSSQNICIVKLN--TD----------------RPIYVFQLATVRSLGIS--DCLFRMEVGSMASTGPGELWMQVEDSSTAHTMHDCIFQ------------------------------------------------------------------------------------------------------------------------------------------------------------------------------------------------------------------------------------------------------------------------------------------------------------------------------------------------------------------------------------------------------------------------------------------------------------------------------------------------------------------------------------------------------------------------------------------------------------------------------------------------------------------------------------------------------------------------------------------------------------------------------------------------------------------------------------------------------------------------------------------------------------------------------------------------------------------------------------------------------------------------------------------------------------------------------------------------------------------------------------------------------------------------------------------------------------------------------------------------------------------------------------------------------------------------------------------------------------------------------------------------------------------------------------------------------------------------------------------------------------------------------------------------------------------------------------------------------------------------------------------------------------------------------------------------------------------------------------------------------------------------------------------------------------------------------------------Obim_Ocbimv22029756_partial/1-231  ----------------------------------------------------------------------------------------------------------------------------------------------------------------------------------------------------------------------------------------------------------------------------------------------------------------------------------MKKKFFVLRGNSNS------------GPARLEYYDSEKKYRSYSV--------------------PKRSIELNSCFNI-NKKIDPKHKHAVSLFTKDDCFSIVAENA-EEQDAWLDCLLDLQ-NTPY-------------------------------------------------------------------------------HFEY----------------VWQVTVKPKDLGAT----KNM-TGQFRLCLTNHEISLVRLN--SE----------------IPEVTFQLGYIRRCGHR--DCLFFMEVGRLAPTGHGELWMQVEDQATARNMHDCILGWRQVLWL----------------LERLRKNVKNRSFS----------------------------------------------------------------------------------------------------------------------------------------------------------------------------------------------------------------------------------------------------------------------------------------------------------------------------------------------------------------------------------------------------------------------------------------------------------------------------------------------------------------------------------------------------------------------------------------------------------------------------------------------------------------------------------------------------------------------------------------------------------------------------------------------------------------------------------------------------------------------------------ATLVYHSIHGL---------------------------------------------------------------------------------------------------------------HCE---------------------------------------------------------------------------------------------------------------------------------------------------------------------------------------------------------------------------------------------------------------------------------------------------------------------------------------------------------------------------------------------------------------------------------------------------------------------------------------------------------------------------------------------------------------------------------------------------------------------------------------------MTWFGL---------------------------------------------------------------------C-------------------------------------------------------------Cele_ist-1/1-1093                  --------MSEEEESTDNSGKPPIEHPHDREVKSEKQSSNVLPGILRRAE--STDDAHQVRRSSMRPYESFDGLKNDGILKRKRSFFVKTVSFSATVSELMLPAEPTSGEPKNDGEY----------------VETM--FDATEEKKQETIAPKQEETVKKLSMAAREER-IRESRQ-QGQLKEKRLKNPDSKGDTSHD---KPTRE----------TW-------------------------KPLAVEDLPKDEDPDEF-GIPEVYKCGNCL-------------------------------VGF----------APVKKKKLMFVTLTERC-----------------LELHESEKSYRAGKA--------------------AKHMVDLSMSFNVHSEHYDAKLKKCLCLMGPDETICMRPEGGILTIEGWRRAIVKLI-HESRRRKMDRVPR-----------------------------------------------PED--------------------IFDAAYDVRVCLFPKNLE--KYVESLKTDGFTNICTVAKEL-LGKKRLCLYPNTLAIVDLC--IEPTAYGLPPAGFPPFRASSMFILERNTVAYYGFR--ENYFYVRIGKGSPYRGFELLFQVDTNEVCKEIYSRLRALADRDLENR---------KQESIRRPERPRVSTLQSV-------KPG-------KE--------------------ETGLREALLESYG------------------------------------------------------------------------------HLRKKNSTQSERGEIVVPEQERKMSDN------RP----------------------------RLRTQDIKT--------------------------------------------------VTKPELKSEEPIRPR------LPPLKFNAN-----------------KPSGEFLLSL-----------------QREKELMEEARKNGYDGRTHNPD----GTPREIKKD---------FLDTVCPQAAAP---------------EVVIKE--KIINNDTYTLMGPADWGKLEDI----------------------------------------VKDDYSDSGDSCYSSRRGTGSQPT---RPAASHLACQMQNRTQ------------------SFGAKQQTFQNRL------------PPTVNLPDSERKI-------SAANQGTSNQLDL--------------PQEDPRKRAFSLGSK----NFFNLIGLNDFRR---LVS----KRHRTSSP-----NHTSTSGI------------------------------------------SLNSSNASPSASSN---------------------------------------------------------FLASSEYLEHARTDSFGS------------------ARSSPKS------------------------------LHTQRTSSPK------------------------RRSD-EDLISIDFSR---------------LGKNSASDT----KRFPFGGGGPGGSFDYDREKREKEDNNR-RDREKAAMDLKR----KEE-------WEARELAEKKRELER-----------------------------------------------KIQAKKDRKLEKGRG-----KDREKDKDHDH-----------------DKEFRPKAD-----------SGIADCTPSSSF--------SGKKDH------------KNDGSSDSAYMD------------------------------------------------------------------------------------KEGLKYL---------------------ADI----KKRKKEQGAIDTTNSSSS--------SL-----------------------------------------STIVS--------------------------------------------------------IEDNK-----------VTRRITET------------------VETKVVTADLAK---------------------------ALGAIDPNRRSSACIE--------INRKPSI-----------------------------------------C----------------------TAIMEERE----------------------GE-ASETDSRGEPSDRKTTAA-PVTR---------------------------KPASTGSSMSPFRRLKF-------------------------------------LSFRK----Tcas_LOC103313904_irs1/1-918       --------MSG-----------------------------------------------------------------------------------------------------------------------------------------------------------------------------------------------------------------------------------------------------------SEGGVVRSGYLK--------------------------------------------KLKTSRKKFFVLRAETAD------------ASARLEYYDSERKFNNGLP--------------------PKRSIPLKNCFNI-NKRLDTKHKHVIALYTKDDCFCVVLDND-DDLDSWLKALLKLQ-HGEEVVDG--------------------------------------------------ETPKP--------------------TFEH----------------VWQVTVLNRGLGNA-----GL-TGNYRLCLTDKTLSLIKRD--CE----------------KPTIELNLSNIRSCGNL--RDFFFLEIGRSSTLGAGELWMQTEDNNIAQNVHSTVFHAMSTNSS---------------SKDELGPKSRNRSSS-------ATESSKPNM-TR------KIHIAPKCHIFLP-PDVFHTSDTQVIPAGSSTQ----------------------HPLVSGVASFYQHLIQRGAKASERRHS------------ISAGSLTHCGVISHQRTQSLPLANP--APLSDHSHS--------RK--------RPKCMSRERCDSMPS-------RARTTSE--------------------GNHRHLNP--------YRPASLYGRD--------ISH--SPPL--------GSPVSPPCS-----------------TDSAGSSYSL-------------------TDETDVCTEMEPSLG-------RYGHSLTPDEA----------IAEEDCPDSPPY----------------------------NNYISMTLH----------------------------------------------------SSDDGYVDMSPRGRHHNSPT--------ASMSSVTSGTPSTDMRFAEYPLEKVT---SYLAD---DDGRPA-RAYSVGSK--------PERYKKY-------------------------------------------------------TEMGGNSDNLRARALSV------GSKKPP-----ARVLPPHG---------------------------------------------YHHPGTKSSSAPILTNSRG-------------------------------QGSYNSIGPMDDLMEMD-FSHNSGYMDMKPVNSGYVE-------MKPGRK----PETSPY---VDMSSG----------------------------SSPA------KPSYISPAATT---------TQ--TEVYMEMDP-------------------------RKNHSEYLSMS---------------------FTKKLPSPGSPG----TPE-GYVEMS---LGRGHNPSQLTE----------------------------------------------------------------------------DYANMSLNNTKKRPNRKKDKNRSEPIN-------------IQGGPNPRSLL---LARK-------------------HSTGTPPTMYLPLVE-------------ATPHDSKDSS----------------SSSINTPSSSSTIFPIS--------------------LNSPSKATPDYIPMDF----------------------------DKSDYVNYD---------------------------------------------------------------------------------------PKPGVGEFRPINE-----------------SNDDE-GRAY----EVLRPSSSES---------CSTIGGSR----PASVSSAH------------------------------------------------------------------------------------------------------------------------------------MHYASLDLDEGGNRS----------------------------PRTVKG-------------------------SGGEQGEVTLTYAAIDFVKSEGLKHNAVAATN-----------------------AKVKH---Dmel_chico/1-968                   --------MAS----------------------------------------------------------------------------------------------------------------------------------------------------------------I------------------------------------------------------------------------------------------SDDGMALSGYLK--------------------------------------------KLKTMKKKFFVLYEETST------------SAARLEYYDTEKKFLQRAE--------------------PKRVIYLKNCFNI-NRRLDTKHRFVIVLSSRDGGFGIVLENE-NDLRKWLDKLLVLQ-RNIANSNGTAHS-----------------------------------------------------------------------PYDH----------------VWQVVIQKKGISEK----VGI-TGTYHCCLTSKSLTFVCIG--PEKTPNGEDR--------VASIEILLTTIRRCGHASPQCIFYVELGRQSVLGSGDLWMETDNAAIATNMHNTILSAMSAKTESNTNLINVYQNRPDLSHEPMRKRSSSANEA-------SKPINVNVI-QNSQNSLELRSCSSPHNYGFG------RERCDSLP------------------------------------------------------------------------------------------------TRNGTLS----------------------ESSNQTYFGSNH----GLRSNTISG------------------------------------IRPHSTNKHSNSPTFTMPLRC--SESE--------ESSISVDES-----------------DDNGSFSHYR-----------------LNTRSSETAIPEENID--------DFASAE---------------LFSKVTEQNVSD----------------------------ENYIPMNPVNP--------------------------------------------------TDAIHEKEKADMQRLEDASLHFNFP---------------------EHASEKLA--KDFDLDSDNQCCRPI-RAYSIGNK--VEHLKFNKRLGHL-----NDTGQNPNRVRAYSVGSKSKIPRCDLQRVVLVEDNKHEFTANRS----QSSITKEGTSYGSSANRQK------KSTSAPLLSLKNQINSDRMSDLMEIDFSQATNL-----------------EKQKFIKNNEIPKYIENVFPKAPRTDSSSLT--------------------------------LHATSQKDIFNGTKLNNTAITSEDGYLEMKPVG----------------------NGYTPSS---NCLPMKVEKL-----------------KLSDYQTAPPL-----------------------------TATAAPVHD---------------LNKISTYNI-------------------------------------------------SAE-----------KWREQPSRSEE-----------------------------------------------------------------------------------------------KKSNSPLND------------NTFSSKPTNVE---------STS--------------KSHDVHSANQIDCEKVC---------AQSSDKLNNHLA---------DKIV--ENNNLDIGGHEEKKLVHS-------------------------ISSEDYTQIK----------------------------DKSNDFTKFNEAGYKI-----------------LQIKSD---------------------------SSLIS----------------------------------------------SKLY--QKGIHKDNL-ER---------SQRLTES---------VNTI-----PDNATATAVSSS------------------------SLTKFNINSAKPAAAADSRSTGTDPSTPQNILQIKDL----NFP---------------------------------------------------SRSSSRISQPE-LHYASLDLPHCSGQN----------------------------PAKYLKRGSRESP------------------PVSACPEDGNTYAKIDFDQSDSSSSSSNIFNT-------------------------------Hvul_LOC100192280/1-717            --------MSQ-------------------------------------------------------------------------------------------------------------------------------------------------------------------------------------------------------------------------------------------------------------DKYIRKGTLLK-------------CPHKPLN---------P-------------FKQKWQSRWFVLYDNSEH------------GRVRIEYYDSEKFWQQEK---------------------SKRVISLSNCVSI-KLVWNKEYNHVIEIVIPDRTIHLAAQSK-EEQKLWFKDMCEIIFGNSSKSSLVQVVKKLSSEHDIGYHSLSRTTKEEIDNSPRS--LHTVPEVSHEVDSGDLLIKENKETRKINKVSSVGSELSSLVSFESGFDDASSMTSTITEGCTYPVSIRATIASQK----AGM-NGKYLLQVTPLSLLLIDIP--SQ----------------CVICEWPIQFLRRYGRG--RTKFSFEASEKCKHGKGVYTFDTLDGDSIFHIVHAHVQGISAKNHSS-------------VHEGFKPIGRSFHN-------------------------------------------SEGKLLDKLS------------------------------------------------------------------------------EISFKGDSHI-------GEGSNDVLNV--------------------------------------------------------------------------------------------------------NPSD------------SVSQSGTLQ-------------RSYGVDSNNT------------------------------------LKRDMSIGSGIS-----------------------------------------LEIAQN--------------------------------------------------------------------ISDNTEESKHFRKRFENFDP--------------RTRCEILESSFDEEPLDDMG--KSDDDD--------------------LVHTLDHKPIRNL------ETLCEKPEKDNQPFL-------------NHEDKKKKPVKRSSSLKL-----------FAS---LKP---------NSS-----KELKKSNS---------------------------------------VRRSSSFRNRFFKSKSTDV-------------------------------------------NKDSDKKYKN---ISKSDYDLTTDSQPTQP------------------TDLTPP-------------------------------SLTPNISPPSPCIISKHNKVKEIIAKA------I--ED-------DMSR---------------SSETEFQEKESPVQNRSIR-----RKFPVRHKSNEMLGN-------------------GNE-----------KNVFTPSRLSS--------------------------------------------------------------------------------------------------------------------------------------------------------------------------------------------------------------------------------------------------------SFAGYDYR-------------------GN----------------------------------------------------------------------------AAVCH----------------------------------------------------VSNSDKDNEKEE--------LKRLQRRT---------------------QSV-----------------------------------------------------------------------------------------------------------------------------------------MRKVEL-------------------------------------------------------------------------------YEGIRRQADEHKINNYTFWRKRQQ---------------------LGKY---Hvul_LOC101240709/1-892            --------MKS------------------------------------------------------------------------------------------------------------------------------------------LLVALDEDSIEGKSF----------------------------------------------------------------------------------FAMED---KNKSHTL-RQDNILMKGILK--------------------------------------------SLQNNKDQLFVLYKKGFA------------SIARLESYENIKQPDY-----------------------PSKVIFLSDCFDV-IRTSIKRNPFTIILFTKNERLLVSAETE-RDCMLWLTAIEQER------------------E-----------------------------------ACGSFVYNEP--------------------KYLK----------------SWIVNVRCRG-------NFSL-KGWYLLGVTLTQLDFVSQS--VA----------------GVVSSLDVKSIERSGYT--ENSFYMEIWMCNTL--CLLWLDMEDPQSACSIDQLIKQLTGKGKKSFT-------D-SPLLRKSYKDKAKNITQ-----------INLLSK-AS-----------------------LYSTDEDEISSASS--------------------------------------------------------------------------LCSSRSNS----------SDFQKLN----------------------------------------KFKNSSS-------------------------------------------------FCSSPELH--PPDHKN------KYPYCSSSSSNVSEC-------NDISLRDGEANENCF--------------SSLHNTSASSSLADDESD---VFLDLEQAVSTNDL---------------------------------------TSVYRN-------PTYVDFSSKSLQ---HLS--------------------------------------I----NTDIEERQKKSKLKT---------------LLRRRFSKNLEKASIDKEKLSISDLHSSIFAQ--------------------LEFRPSPSVFKNI------RTRSGINEDCTKTMT-------------KSLNSDIEPVYESVR----------SPNDFAQSKFLSPN----SEKVYGA-----NDFATSRR-------------------------------------TL------YRNALP-----------------------------------------------PIPPQRITSRFSFD-GVDTTDFFNKKKEKDS--------------------KKDNPYE---EVSYEKMI---------------NK--SLSTIEIPPHI-----TQGYEQMNAES-------SIGE--SKSYEEISGG-------------PLTRNKINDTHRDVSTKSFS-----KAMVFKLTENDSLNFDDEEDSYAKISPSPRDF-QSKENNGVLKT----FMAIKKNDLSI-GCQSP------------LECS------------------------RVISRGTPAHSTPTH-----------------------------------NREMPVL-------------NRNKISPSISG--------------------------LKMNLSSYTNVSPSF-----------------------------------------------------PHS------------------------PSHDFDYSVPYK-----------NEIK------------EEELTYLNNSYLSSNT---TPEY-------------------------------------------ESMLY-----------------------------------------------------PNKLDDNA----------------------------YDNIGNQI----------------------------------------------NKRCKTLAYADVLIDKPS--------------------------------------------------------------------------------LNYSDKDK--------ELL------------------------------------------------------------KEDECCYMQIDFEKSQGVSEAIKDLRTKQSPTNHLSSSNKNDYLTDLSYKTFKKFNNT5. PIK3CHsap_PIK3CA/1-1068                            -----------------------------------------------------------------------------------------------------------------------------------------------MPP---------------------------------------------------------------------RPSSG----ELWGIH--------------------------------------LMPPRILVECL-----------------------------------------------------LPNG------------------------------------------------------------------------------------MIVTL---------------------ECL----------------------------------------------------------------------------------------------------------------------------------------REA---------TLITIKHELFKEARKY--------P----------------------------------------LHQLLQDE-------------------------------------------------------------------------SSYIFVSVTQEA-----------------------------EREEFFDETRRLC----------DLRLF---------------QPFL----------------KV-----IEP-----VGNRE------------EKILNREIGFAIGM-------PVC----EFDMVKDPEVQDFRRNILNVCKEAVDLRDLNSPHSRAMYVYPPNVESSPEL--PKHIYNKLDK---------GQIIVVIWVI-VSPN-------------------NDKQKYT-LKINHDCVPEQVIAEAIR----KKTRSMLLSSEQLKLCVLEYQGKYILKVCGCDEYFLE-------------KYPLSQYK---------YIRSCIM---------LGRMPNLMLMA-----KESLY-SQLPM------------------------------------------------------------------------------------------DCFTMPSYSRRI------------------STATPYMNGE--------------------------------------------------------------------------TSTKSLWVINSA--LRIKILCATYVNVNI-----RDIDKIYVRTGIYHGGEPLCDNVNT---QRVP--CSN----------------P-RWN-EWLNY-DIYIPDLPRAARLCLSICSVKGRKGAK-------------------EEHCPLAWGNINLFDYTDTLVSGKMALNLWPVPHGL----------------EDLLNPIG----------VTGSNPN------------------------K-ET----PCLELEFDWFS-SV----------------------------------VKFPDMSVIE-------------------------------EHANWSVSREAGFSYS--HAGLSNRL----AR-----D--NELRENDKEQLKAIS-----TRDPLS--EITEQEKDFLWSHRHYCVT-IP-EILPKLLL--SVKWNSRDEVA---QMYCLVKD---W--PPIKPEQAMELLDCNYPDPMVRGFAVRCLEKYLTDDKLSQYLIQLVQVLKYEQY-------------------------------------------------------------------------------------------------------------------------------------LDNLLVRFLLKKALTNQRIGHFFFWHLK-SEMH-----------------NK--------TVSQRFGLLLESYCRACGM-------YLKHLNRQVEAMEKLINLTDILKQEK--KDETQK---------VQMKFLVEQMRR-PDF-----MDALQGFLSPLNPAHQLGNLRL------------------------------------EECRIMSSAKRPLWLNWENPDIM-------SELLFQNNEIIFKNGDDLRQDMLTLQIIRIMENIWQNQGLDL-----------RMLPYGCLSIGDCVGLIEVVRNSHTIMQIQCKGGL--KGALQFNSHTLHQWLKDKNK--GEI-------------------------YDAAIDLFTRSCAGYCVATFILGIGDRHNSNIMVKD----DGQLFHIDFGHFLDHKKKKFGYKRERVPFVLTQDFLIVISK-GAQECTKTREFERFQEMCYKAYLAIRQHANLFINLFSMMLGSGMPELQSFDD--IAYIRKTLALD-KTEQEALEYFMKQMNDAHHGGWTTKMDWIFHTIKQHALN----------------------------------------------------------------------------------------------------------------------------------------------------------------------------------------------------------------------------------------------------------------------------------------------------------------------------------------------Hsap_PIK3CB/1-1070                            -------MCFSFI----------------------------------------------------------------------------------------------------------------------------------MP-----------------------------------------------------------------------PAMA-DILDIWAVDSQI-----------------------------------ASDGSIPVDFL-----------------------------------------------------LPTG------------------------------------------------------------------------------------IYIQL---------------------EVP----------------------------------------------------------------------------------------------------------------------------------------REA---------TISYIKQMLWKQVHNY--------P----------------------------------------MFNLLMDI-------------------------------------------------------------------------DSYMFACVNQTA-----------------------------VYEELEDETRRLC----------DVRPF---------------LPVL----------------KL-----VTR-----SCDPG------------EK-LDSKIGVLIGK-------GLH----EFDSLKDPEVNEFRRKMRKFSEEKILSLVGLSWMDWLKQTYPPEHEPSI----PENLEDKLY---------GGKLIVAV-----HFE-------------------NCQDVFS-FQVSPNMNPIKVNELAIQ----KRLTIHGKED-------EVSPYDYVLQVSGRVEYVFG-------------DHPLIQFQ---------YIRNCVM---------NRALPHFILVE-----CCKIK-KMYEQ------------------------------------------------------------------------------------------EMIAIEAAINRNSS----------------NLPLPLPPKK-------------------------------------------------------------------------TRIISHVWENNNP--FQIVLVKGNKLNT-------EETVKVHVRAGLFHGTELLCKTIVS---SEVS-GKND----------------H-IWN-EPLEF-DINICDLPRMARLCFAVYAVLDKVKTKKSTKTINPSKY-QTIRKAGKVHYPVAWVNTMVFDFKGQLRTGDIILHSWSSFPDEL---------------EEMLNPMG----------TVQTNPY------------------------TENA----TALHVKFPENKKQP----------------------------------YYYPPFDKII-------------------------------EKAAEIA----------------------SSDSANV-S--SRGGKKFLPVLKEIL-----DRDPLS--QLCENEMDLIWTLRQDCREIFP-QSLPKLLL--SIKWNKLEDVA---QLQALLQI---W--PKLPPREALELLDFNYPDQYVREYAVGCL-RQMSDEELSQYLLQLVQVLKYEPF-------------------------------------------------------------------------------------------------------------------------------------LDCALSRFLLERALGNRRIGQFLFWHLR-SEVH-----------------IP--------AVSVQFGVILEAYCRGSVG-------HMKVLSKQVEALNKLKTLNSLIKLNA--VKLNRAK---------GKEAMHTCLKQ-SAY-----REALSDLQSPLNPCVILSELYV------------------------------------EKCKYMDSKMKPLWLVYNNKVFG-------EDS----VGVIFKNGDDLRQDMLTLQMLRLMDLLWKEAGLDL-----------RMLPYGCLATGDRSGLIEVVSTSETIADIQLNSSNV-AAAAAFNKDALLNWLKEYNS--GDD-------------------------LDRAIEEFTLSCAGYCVASYVLGIGDRHSDNIMVKK----TGQLFHIDFGHILGNFKSKFGIKRERVPFILTYDFIHVIQQ-GK--TGNTEKFGRFRQCCEDAYLILRRHGNLFITLFALMLTAGLPELTSVKD--IQYLKDSLALG-KSEEEALKQFKQKFDEALRESWTTKVNWMAHTVRKDY-----------------------------------------------------------------------------------------------------------------------------------------------------------------------------------------------------------------------------------------------------RS-----------------------------------------------------------------------------------------Hsap_PIK3CG/1-1102                            -------ME---------------------------------------------------------------------------------------------------------------------------------LENYKQP------------------------------------------------VVLREDNCRR--------RRRMKPRSA--------------------------------------------AASLSSMELIPIEFV-----------------------------------------------------LPTSQRKC-----------------------------------------------------------------------------KSPETALL---------------------HVA----------------------------------------------------------------------------------------------------------------------------------------GHG---------NVEQMKAQVWLRALETS-----VAAD---------------------------------------FYHRL---------------------------------------------------------------------------GPHHFLLLYQKKG-----------------------------QWYEIYDKYQVVQ----------TLDCLRYWK------ATHRSPGQI----------------HL-----VQR----HPPSEE------------SQAFQRQLTALIGY-------DVT----DVSNVHDDELEFTRRGLVTPRMAEVASRD------PKLYAMHPWVTSK-PL--PEYLWKKIAN---------NCIFIVI-------H-------------------RSTTSQT-IKVSPDDTPGAILQSFFTKMA-KKKSLMDIPE-------SQSEQDFVLRVCGRDEYLVG-------------ETPIKNFQ---------WVRHCLK---------NGEEIHVVLDTPPDPALDEVR-----------------------------------KEEWPLVD-----------------------------------------------------DCTGVTGYHEQLTIH---------------------------------------------------------------------------------------------GKDHESVFTVSLWDCDRK--FRVKIRGIDIPVLPR-----NTDLTVFVEANIQHGQQVLCQRRTS---------PKPFTEE------------V-LWN-VWLEF-SIKIKDLPKGALLNLQIYCGKAPALSSKAS-------AESPSSESKGKVQLLYYVNLLLIDHRFLLRRGEYVLHMWQISGKGED--------------QGSFNADKL---------TSATNPD------------------------KENS----MSISILLDNYC-HP----------------------------------IALPKHQPTP-------------------------------DPEGDRVR--------------------------------AEMPNQLRKQLEAII-----ATDPLN--PLTAEDKELLWHFRYESLK-HP-KAYPKLFS--SVKWGQQEIVA---KTYQLLARREVWDQSALDVGLTMQLLDCNFSDENVRAIAVQKL-ESLEDDDVLHYLLQLVQAVKFEPY-------------------------------------------------------------------------------------------------------------------------------------HDSALARFLLKRGLRNKRIGHFLFWFLR-SEIA-----------------QS-------RHYQQRFAVILEAYLRGCGT------AMLHDFTQQVQVIEMLQKVTLDIKSLS--AEKYDVSS-------QVISQLKQKLEN-LQN-----SQLPESFRVPYDPGLKAGALAI------------------------------------EKCKVMASKKKPLWLEFKCADPTAL----SNET----IGIIFKHGDDLRQDMLILQILRIMESIWETESLDL-----------CLLPYGCISTGDKIGMIEIVKDATTIAKIQQSTVG---NTGAFKDEVLNHWLKEKSP-TEEK-------------------------FQAAVERFVYSCAGYCVATFVLGIGDRHNDNIMITE----TGNLFHIDFGHILGNYKSFLGINKERVPFVLTPDFLFVMGTSGK---KTSPHFQKFQDICVKAYLALRHHTNLLIILFSMMLMTGMPQLTSKED--IEYIRDALTVG-KNEEDAKKYFLDQIEVCRDKGWTVQFNWFLHLVLGIKQGE--------------------------------------------------------------------------------------------------------------------------------------------------------------------------------------------------------------------------------------------------KHSA---------------------------------------------------------------------------------------Hsap_PIK3CD/1-1044                            -----------------------------------------------------------------------------------------------------------------------------------------------MP-----------------------------------------------------------------------PGVD-CPMEFWTK---------------------------------------EENQSVVVDFL-----------------------------------------------------LPTG------------------------------------------------------------------------------------VYLNF---------------------PVS----------------------------------------------------------------------------------------------------------------------------------------RNA---------NLSTIKQLLWHRAQYE--------P----------------------------------------LFHMLSGP-------------------------------------------------------------------------EAYVFTCINQTA-----------------------------EQQELEDEQRRLC----------DVQPF---------------LPVL----------------RL-----VAR-----EGDRV------------KKLINSQISLLIGK-------GLH----EFDSLCDPEVNDFRAKMCQFCEEAAARRQQLGWEAWLQYSFPLQLEPSAQTWGPGTLRLP-----------NRALLVNV-----KFE-------------------GSEESFT-FQVSTKDVPLALMACALR----KKATVFRQPL-------VEQPEDYTLQVNGRHEYLYG-------------SYPLCQFQ---------YICSCLH---------SGLTPHLTMVH-----SSSIL-AMRDE------------------------------------------------------------------------------------------QSNPAPQVQKPR------------------AKPPPIPAKK--------------------------------------------------------------------------PSSVSLWSLEQP--FRIELIQGSKVNA-------DERMKLVVQAGLFHGNEMLCKTVSS---SEVS-VCSE----------------P-VWK-QRLEF-DINICDLPRMARLCFALYAVIEKAKKARSTK-----------KKSKKADCPIAWANLMLFDYKDQLKTGERCLYMWPSVPDEK---------------GELLNPTG----------TVRSNPN------------------------TDSA----AALLICLPEVAPHP----------------------------------VYYPALEKIL-------------------------------ELGRHSEC--------------------------------VHVTEEEQLQLREIL-----ERRGSG--ELYEHEKDLVWKLRHEVQEHFP-EALARLLL--VTKWNKHEDVA---QMLYLLCS---W--PELPVLSALELLDFSFPDCHVGSFAIKSL-RKLTDDELFQYLLQLVQVLKYESY-------------------------------------------------------------------------------------------------------------------------------------LDCELTKFLLDRALANRKIGHFLFWHLR-SEMH-----------------VP--------SVALRFGLILEAYCRGSTH-------HMKVLMKQGEALSKLKALNDFVKLSS--QKTPKPQ---------TKELMHLCMRQ-EAY-----LEALSHLQSPLDPSTLLAEVCV------------------------------------EQCTFMDSKMKPLWIMYSNEEAG------SGGS----VGIIFKNGDDLRQDMLTLQMIQLMDVLWKQEGLDL-----------RMTPYGCLPTGDRTGLIEVVLRSDTIANIQLNKSNM-AATAAFNKDALLNWLKSKNP--GEA-------------------------LDRAIEEFTLSCAGYCVATYVLGIGDRHSDNIMIRE----SGQLFHIDFGHFLGNFKTKFGINRERVPFILTYDFVHVIQQ-GK--TNNSEKFERFRGYCERAYTILRRHGLLFLHLFALMRAAGLPELSCSKD--IQYLKDSLALG-KTEEEALKHFRVKFNEALRESWKTKVNWLAHNVSKDN-----------------------------------------------------------------------------------------------------------------------------------------------------------------------------------------------------------------------------------------------------RQ-----------------------------------------------------------------------------------------Hsap_PIK3C2A/1-1686                           -------MAQISS------------NSGFKECPSSHPEPTRAKD---------------------------------------------------VDKEEALQMEAEALAKLQKDRQVT---------------------------------------DNQRG--FELSSSTRKKAQVYNKQDYDLMV-------FPESD-----------SQK-----RALDIDVEKLTQAELEKLL---LDDSF----------ETKKTPVLPVTPILSPSFSAQLYFRPTIQRGQ-----------------WPPGLPGPSTYA---------------LP-------------------------------------------------SIYPST-YSKQ-AAFQN----GFNPRMPTFPS--TEPIYLSLP--------------------GQSPYFSYPLTPATP-------FHPQGSLPIYRPV--------------VSTDMAKLFD---------------------------------------------------------------------KIASTSEFL--KNG------------KARTDLEITDSKVS-----NLQV-------------------SPKSEDISK-------FDWLDLDPLSKPKVDNVEVLD-------------------HEEEKN--VSSLLAKDPWDAVLLEERSTA----------------NCHLERKVNGKS-----------------------------LSVATVTRSQSLN------------------------------IRTT----------------QL------------AKAQGH----------ISQKDPNGTSSLPTGS-------SLL----QEVEVQNEEMAAFCRSITKL---------------KTKFPYTNHRTN------PGYLLSPVTAQRN-ICGENASVKVSI-----DIE-------------------GFQLPVT-FTCDVSSTVEIIIMQALC----WVHDDLNQ----------VDVGSYVLKVCGQEEVLQN-------------NHCLGSHE---------HIQNCRK---------WDTEIRLQLLT-----FSAMC-QNLARTAEDDETPVDLNKHLYQIEKPCK-EAMTRHPVEELLDSYHNQVELALQIE-----------------NQHRAVDQVIKAVRKICSALDGVETLAITESVKKLKRAVNLPRSKTADVTSLFGGEDTSRSSTR------------GSLNPENP-------VQVSINQLTAAIYDL--LRLHANSGR-----------SPTDCAQSSKSVKEAWTTTEQ--LQFTIFAAHGISSNWV----SNYEKYYLICSLSHNGKDLFKPIQS---KKVG-TYKNFFYL------------I-KWD-ELIIF-PIQISQLPLESVLHLTLFGILNQSSGSSPD-----------SNKQRKGPEALGKVSLPLFDFKRFLTCGTKLLYLWTSSH---------------------TNSVPG---------TVTKKGY------------------------VMER----IVLQVDFPSPA-FD----------------------------------IIYTTPQVDR-------------------------------SIIQQ-------------------------HN-----L--ETLENDIKGKLLDIL-----HKDSSL--GLSKEDKAFLWEKRYYCFK-HP-NCLPKILA--SAPNWKWVNLA---KTYSLLHQ---W--PALYPLIALELLDSKFADQEVRSLAVTWI-EAISDDELTDLLPQFVQALKYEIY-------------------------------------------------------------------------------------------------------------------------------------LNSSLVQFLLSRALGNIQIAHNLYWLLK-DALH-----------------DV--------QFSTRYEHVLGALLSVGGK------RLREELLKQTKLVQLLGGVAEKVRQAS--GSARQVV-------------LQRSMER-VQS-----FFQKNKCRLPLKPSLVAKELNI------------------------------------KSCSFFSSNAVPLKVTMVNADPM-------GEE----INVMFKVGEDLRQDMLALQMIKIMDKIWLKEGLDL-----------RMVIFKCLSTGRDRGMVELVPASDTLRKIQVEYGV----TGSFKDKPLAEWLRKYNP-SEEE-------------------------YEKASENFIYSCAGCCVATYVLGICDRHNDNIMLRS----TGHMFHIDFGKFLGHAQMFGSFKRDRAPFVLTSDMAYVING-GE---KPTIRFQLFVDLCCQAYNLIRKQTNLFLNLLSLMIPSGLPELTSIQD--LKYVRDALQPQ-TTDAEATIFFTRLIESSLG-SIATKFNFFIHNLAQLRFSGL--PSNDEPILSFSPKTYS-FRQDGRIKEVSVFTYHKKYNPDKHYIYVVRILREGQIE--PSFVFRTFDEFQELHNKLSIIFPLWKLPGFPN--------RMVLGRTHIKDVAAKRKIELNSYLQSLMNASTDVAECDLVCTFF-----HPLLRDEKAEG----------IARSAD--AGSFSPTPGQIGGAVKLSISYR--NGTLFIMVMHIKDLV--TEDGADPNPYVKTYLLPDNHKTSKRKTKISRKTRNPTFNEMLVYSGYSKETLRQRELQLSVLSAESL---RENFFLGGVTLPLKDFNLSKETVK----WYQLTAATYL------Hsap_PIK3C2B/1-1634                           -------MSST---------------QGNGEHWKSLESVGISRK--------------------------------------------------ELAMAEALQMEYDALSRLRHDKEEN--------------------------------------------------------------------------------------------RAKQNADPSLISWDEPGVDFYS-------------------KPAGRRTDLKLLRGLSGSDPTLNYNSL---------------------SPQEGPPNHSTSQGP-----------------QPGSDP-----------------------------------------------WPKGSLS-------------------------GDYLYIFDG--------------------SDGGVSSSPGPGDIE-------GSCKKLSPPPLP-----------------PR-------------------------------------------------------------ASIWDTPPLPPR--KGSPSSS----KISQPSDINTFSLVEQLPGKLLEHRILE------EEEV--LGGGG------------------------QGRLLGSVDYDGINDA------------------------------------ITRLNLKSTYDAEMLRDATRG-----------WK---EGRGPLDFSKDT------------------------------SGKPVARSKTMP----------------------------PQVPPR----------------TY-ASRYGNRKNATPGKNRR---------ISAAPVGSRPHTVANGH-------ELF----EVSEERDEEVAAFCHMLDIL---------------RSGSDIQDYFLT-------GYVWSAVTPSPE-HLGDEVNLKVTV-----LCD-------------------RLQEALT-FTCNCSSTVDLLIYQTLC----YTHDDLRN----------VDVGDFVLKPCGLEEFLQN-------------KHALGSHE---------YIQYCRK---------FDIDIRLQLME-----QKVVR-SDLARTVNDDQSPSTLNYLVHLQERPVK-QTISRQALSLLFDTYHNEVDAFLLADGD----------------FPLKADRVVQSVKAICNALAAVETPEITSALNQLPPCPSRMQPKI-------QKDPSVLAVR--------------------------ENREKVVEALTAAILDL--VELYCNTFNADFQT-------AVPGSRKHDLVQEACHFARS--LAFTVYATHRIPIIWA----TSYEDFYLSCSLSHGGKELCSPLQT---RRAH-FSKYLFHL------------I-VWD-QQICF-PVQVNRLPRETLLCATLYALPIPPPGSSSE-----------ANKQRRVPEALGWVTTPLFNFRQVLTCGRKLLGLWPATQ---------------------ENPSAR---------WSAPNFH------------------------QPDS----VILQIDFPTSA-FD----------------------------------IKFTSPPGDK-------------------------------FSPR--------------------------YE-----F--GSLREEDQRKLKDIM-----QKESLY--WLTDADKKRLWEKRYYCHS-EV-SSLPLVLA--SAPSWEWACLP---DIYVLLKQ---W--THMNHQDALGLLHATFPDQEVRRMAVQWI-GSLSDAELLDYLPQLVQALKYECY-------------------------------------------------------------------------------------------------------------------------------------LDSPLVRFLLKRAVSDLRVTHYFFWLLK-DGLK-----------------DS--------QFSIRYQYLLAALLCCCGK------GLREEFNRQCWLVNALAKLAQQVREAA--PSARQGI-------------LRTGLEE-VKQF----FALNGSCRLPLSPSLLVKGIVP------------------------------------RDCSYFNSNAVPLKLSFQNVDPL-------GEN----IRVIFKCGDDLRQDMLTLQMIRIMSKIWVQEGLDM-----------RMVIFRCFSTGRGRGMVEMIPNAETLRKIQVEHGV----TGSFKDRPLADWLQKHNP-GEDE-------------------------YEKAVENFIYSCAGCCVATYVLGICDRHNDNIMLKT----TGHMFHIDFGRFLGHAQMFGNIKRDRAPFVFTSDMAYVING-GD---KPSSRFHDFVDLCCQAYNLIRKHTHLFLNLLGLMLSCGIPELSDLED--LKYVYDALRPQ-DTEANATTYFTRLIESSLG-SVATKLNFFIHNLAQMKFTG----SDDRLTLSFASRTHT-LKSSGRISDVFLCRHEKIFHPNKGYIYVVKVMRENTHE--ATYIQRTFEEFQELHNKLRLLFPSSHLPSFPS--------RFVIGRSRGEAVAERRREELNGYIWHLIHAPPEVAECDLVYTFF-----HPLPRDEKAMGT--SP-----APKSSDG---TWARPVGKVGGEVKLSISYK--NNKLFIMVMHIRGLQL-LQDGNDPDPYVKIYLLPDPQKTTKRKTKVARKTCNPTYNEMLVYDGIPKGDLQQRELQLSVLSEQGF---WENVLLGEVNIRLRELDLAQEKTG----WFALGSRSHGTL----Hsap_PIK3C2G/1-1486                           -------MAYSWQTDPN--------PNESHEKQYEHQEFLFVNQ---------------------------------------------------PHSSSQVSLGFDQIVDEISGK---------------------------IP----------------------------------------------------------------H-YESEIDENTFFVPTAPKWDSTG---------------------------HSLNEAHQISLNEFTSKSRELSWHQVSK------------APAIGFSPSV----------------------LPKPQNTNKEC--------------------------------------------------------------------SWGS----------------------------------------------------------------------------------PIGKHH---GA-DDSRF-------------------------------------------------SILAP-------------------------------SFTSLDKINLEKEL-----------------------------ENENHNYHI-----------GFESSIPPTN-----------------------------------------------------------------------SSFSSDFMPKEENK------------RSGHV-------------NIVEPSLMLL--------------------------KGSLQPGM----------------------------------------------WESTWQKNIESIGCSI-------QLV----EVPQSSNTSLASFCNKVKKI---------------RERYHAADVNFN------SGKIWSTTTAFPY-QLFSKTKFNIHI-----FID-------------------NSTQPLH-FMPCANYLVKDLIAEILH----FCTND------------QLLPKDHILSVCGSEEFLQN-------------DHCLGSHK---------MFQK------------DKSVIQLHLQK-----SREAP-GKLSRKHEEDHSQFYLN---QLLEFMHI-WKVSRQCLLTLIRKYDFHLKYLLK--------------------TQENVYNIIEEVKKICSVLGCVETKQITDAVNELSLILQR------------KGENFYQSSET---------------SAKGL-------IEKVTTELSTSIYQL--INVYCNSFYADFQP----------VNVPRCTSYLNPGLPSH--LSFTVYAAHNIPETWV----HSYKAFSFTCWLTYAGKKLCQVRNY---RNIP-DKKLFFFL------------V-NWN-ETINF-PLEIKSLPRESMLTVKLFGIA----------------------CATNNANLLAWTCLPLFPKEKSIL-GSMLFSMTLQSEPP-------------------VEMIT----------PGVWDVS------------------------QPSP----VTLQIDFPATG-WE----------------------------------YMKPDSEENR-------------------------------------------------------------SN-----L--EEPLKECIKHIARLS-----QKQTPL--LLSEEKKRYLWFYRFYCNN-EN-CSLPLVLG--SAPGWDERTVS---EMHTILRR---W--TFSQPLEALGLLTSSFPDQEIRKVAVQQL-DNLLNDELLEYLPQLVQAVKFEWN-------------------------------------------------------------------------------------------------------------------------------------LESPLVQLLLHRSLQSIQVAHRLYWLLK-NAEN-----------------EA--------YFKSWYQKLLAALQFCAGK------ALNDEFSKEQKLIKILGDIGERVKSAS--DHQRQEV-------------LKKEIGR-LEEF----FQDVNTCHLPLNPALCIKGIDH------------------------------------DACSYFTSNALPLKITFINANPM-------GKN----ISIIFKAGDDLRQDMLVLQLIQVMDNIWLQEGLDM-----------QMIIYRCLSTGKDQGLVQMVPDAVTLAKIHRHSGL----IGPLKENTIKKWFSQHNH-LKAD-------------------------YEKALRNFFYSCAGWCVVTFILGVCDRHNDNIMLTK----SGHMFHIDFGKFLGHAQTFGGIKRDRAPFIFTSEMEYFITE-GG---KNPQHFQDFVELCCRAYNIIRKHSQLLLNLLEMMLYAGLPELSGIQD--LKYVYNNLRPQ-DTDLEATSHFTKKIKESLE-CFPVKLNNLIHTLAQMSAIS----PAKSTSQTFPQESCL-LSTTRSIERATILGFSKKSS----NLYLIQVTHSNNE---TSLTEKSFEQFSKLHSQLQKQFASLTLPEFPH--------WWHLPFTNSD---HRRFRDLNHYMEQILNVSHEVTNSDCVLSFFLSEAVQQTVEESSPVYL---------GEKFPDK------------KPKVQLVISYE--DVKLTILVKHMKNIH--LPDGSAPSAHVEFYLLPYPSEVRRRKTKSVPKCTDPTYNEIVVYD--EVTELQGHVLMLIVKS--------KTVFVGAINIRLCSVPLDKEK------WYPLGNSII-------Hsap_PIK3C3/1-887                             -------MG--------------------------------------------------------------------------------------------------------------------------------------------------------------------------------------------------------------------------------------------------------------------------------------------------------------------------------------------------------------------------------------------------------------------------------------------------------------------------------------------------------------------------------------------------------------------------------------------------------------------------------------------------------------------------------------------------------------------------------------------------------------------------------------------------------------------------------------------------------------------------------------------------------------------------------------EAE----------------------------KFHYIYSCD--------------------------LDINVQLKI----GSLE----------------GK-REQKSYK-----------------------------------------AVLEDPMLKFSG-------------------------LYQ--------------------------------------------------------------------------------------------------------------------------------------------------------------------------------------------------------------------------------------------------------------------------------------------------ETCSDLYVTCQVFAEGKPLALPVRT--------SYKAFSTR------------W-NWN-EWLKL-PVKYPDLPRNAQVALTIWDVY-----------------------GPGKAVPVGGTTVSLFGKYGMFRQGMHDLKVWPNVEADGSEPTKTPGRTSSTLSEDQMSRLAK---------LTKAHRQGHMVKVDWLDRLTFREIEMINESEKRSS--NFMYLMVEFRCVKCDDK-EYGIVYYEKDGDES---------SPILTSFELVKVPDPQMSM------------------------------ENLVESKHHKLARSLRSGPSD----------HD--------LKPNAATRDQLNIIV-----SYPPTK--QLTYEEQDLVWKFRYYLTN-QE-KALTKFLK--CVNWDLPQEAK---QALELLGK---W--KPMDVEDSLELLSSHYTNPTVRRYAVARL-RQADDEDLLMYLLQLVQALKYENFDDIKNGLEPTKKDS----------------QSSVSENVSNS---GINS-------------AEIDSS--QIIT----------------------------SPLPSV---------SSPPPASKTKEVPDGENLEQDLCTFLISRACKNSTLANYLYWYVI-VECE-----------------DQDTQQ-RDPKTHEMYLNVMRRFSQALLKGDKSVRVMRSLLAAQQTFVDRLVHLMKAVQRES--GNRKKKN-----------ERLQALLGD-NE---KMNLSDVELIPLPLEPQVKIRGIIP------------------------------------ETATLFKSALMPAQLFFKTED---------GGK----YPVIFKHGDDLRQDQLILQIISLMDKLLRKENLDL-----------KLTPYKVLATSTKHGFMQFI-QSVPVAEVLDTEGS------------IQNFFRKYAP-SENGP---------------------NGISAEVMDTYVKSCAGYCVITYILGVGDRHLDNLLLTK----TGKLFHIDFGYILGR-----DPKPLPPPMKLNKEMVEGMGG------TQSEQYQEFRKQCYTAFLHLRRYSNLILNLFSLMVDANIPDIALEPDKTVKKVQDKFRLD-LSDEEAVHYMQSLIDESVH-ALFAAVVEQIHKFAQ----------------------------------------------------------------------------------------------------------------------------------------------------Y-----------------------------------------------------------------------------------------------------------------------------------------------------------------------------WRK-------------Mmus_PIK3CA/1-1068                            -----------------------------------------------------------------------------------------------------------------------------------------------MPP---------------------------------------------------------------------RPSSG----ELWGIH--------------------------------------LMPPRILVECL-----------------------------------------------------LPNG------------------------------------------------------------------------------------MIVTL---------------------ECL----------------------------------------------------------------------------------------------------------------------------------------REA---------TLVTIKHELFREARKY--------P----------------------------------------LHQLLQDE-------------------------------------------------------------------------TSYIFVSVTQEA-----------------------------EREEFFDETRRLC----------DLRLF---------------QPFL----------------KV-----IEP-----VGNRE------------EKILNREIGFVIGM-------PVC----EFDMVKDPEVQDFRRNILNVCKEAVDLRDLNSPHSRAMYVYPPNVESSPEL--PKHIYNKLDK---------GQIIVVIWVI-VSPN-------------------NDKQKYT-LKINHDCVPEQVIAEAIR----KKTRSMLLSSEQLKLCVLEYQGKYILKVCGCDEYFLE-------------KYPLSQYK---------YIRSCIM---------LGRMPNLMLMA-----KESLY-SQLPI------------------------------------------------------------------------------------------DSFTMPSYSRRI------------------STATPYMNGE--------------------------------------------------------------------------TSTKSLWVINSA--LRIKILCATYVNVNI-----RDIDKIYVRTGIYHGGEPLCDNVNT---QRVP--CSN----------------P-RWN-EWLNY-DIYIPDLPRAARLCLSICSVKGRKGAK-------------------EEHCPLAWGNINLFDYTDTLVSGKMALNLWPVPHGL----------------EDLLNPIG----------VTGSNPN------------------------K-ET----PCLELEFDWFS-SV----------------------------------VKFPDMSVIE-------------------------------EHANWSVSREAGFSYS--HTGLSNRL----AR-----D--NELRENDKEQLRALC-----TRDPLS--EITEQEKDFLWSHRHYCVT-IP-EILPKLLL--SVKWNSRDEVA---QMYCLVKD---W--PPIKPEQAMELLDCNYPDPMVRSFAVRCLEKYLTDDKLSQYLIQLVQVLKYEQY-------------------------------------------------------------------------------------------------------------------------------------LDNLLVRFLLKKALTNQRIGHFFFWHLK-SEMH-----------------NK--------TVSQRFGLLLESYCRACGM-------YLKHLNRQVEAMEKLINLTDILKQEK--KDETQK---------VQMKFLVEQMRQ-PDF-----MDALQGFLSPLNPAHQLGNLRL------------------------------------EECRIMSSAKRPLWLNWENPDIM-------SELLFQNNEIIFKNGDDLRQDMLTLQIIRIMENIWQNQGLDL-----------RMLPYGCLSIGDCVGLIEVVRNSHTIMQIQCKGGL--KGALQFNSHTLHQWLKDKNK--GEI-------------------------YDAAIDLFTRSCAGYCVATFILGIGDRHNSNIMVKD----DGQLFHIDFGHFLDHKKKKFGYKRERVPFVLTQDFLIVISK-GAQEYTKTREFERFQEMCYKAYLAIRQHANLFINLFSMMLGSGMPELQSFDD--IAYIRKTLALD-KTEQEALEYFTKQMNDAHHGGWTTKMDWIFHTIKQHALN----------------------------------------------------------------------------------------------------------------------------------------------------------------------------------------------------------------------------------------------------------------------------------------------------------------------------------------------Mmus_PIK3CB/1-1064                            -----------------------------------------------------------------------------------------------------------------------------------------------MP-----------------------------------------------------------------------PAMA-DNLDIWAVDSQI-----------------------------------ASDGAISVDFL-----------------------------------------------------LPTG------------------------------------------------------------------------------------IYIQL---------------------EVP----------------------------------------------------------------------------------------------------------------------------------------REA---------TISYIKQMLWKQVHNY--------P----------------------------------------MFNLLMDI-------------------------------------------------------------------------DSYMFACVNQTA-----------------------------VYEELEDETRRLC----------DVRPF---------------LPVL----------------KL-----VTR-----SCDPA------------EK-LDSKIGVLIGK-------GLH----EFDALKDPEVNEFRRKMRKFSEAKIQSLVGLSWIDWLKHTYPPEHEPSV----LENLEDKLY---------GGKLVVAV-----HFE-------------------NSQDVFS-FQVSPNLNPIKINELAIQ----KRLTIRGKED-------EASPCDYVLQVSGRVEYVFG-------------DHPLIQFQ---------YIRNCVM---------NRTLPHFILVE-----CCKIK-KMYEQ------------------------------------------------------------------------------------------EMIAIEAAINRNSS----------------NLPLPLPPKK-------------------------------------------------------------------------TRVISHIWDNNNP--FQITLVKGNKLNT-------EETVKVHVRAGLFHGTELLCKTVVS---SEIS-GKND----------------H-IWN-EQLEF-DINICDLPRMARLCFAVYAVLDKVKTKKSTKTINPSKY-QTIRKAGKVHYPVAWVNTMVFDFKGQLRSGDVILHSWSSFPDEL---------------EEMLNPMG----------TVQTNPY------------------------AENA----TALHITFPENKKQP----------------------------------CYYPPFDKII-------------------------------EKAAELA----------------------SGDSANV-S--SRGGKKFLAVLKEIL-----DRDPLS--QLCENEMDLIWTLRQDCRENFP-QSLPKLLL--SIKWNKLEDVA---QLQALLQI---W--PKLPPREALELLDFNYPDQYVREYAVGCL-RQMSDEELSQYLLQLVQVLKYEPF-------------------------------------------------------------------------------------------------------------------------------------LDCALSRFLLERALDNRRIGQFLFWHLR-SEVH-----------------TP--------AVSVQFGVILEAYCRGSVG-------HMKVLSKQVEALNKLKTLNSLIKLNA--VKLSRAK---------GKEAMHTCLKQ-SAY-----REALSDLQSPLNPCVILSELYV------------------------------------EKCKYMDSKMKPLWLVYSSRAFG-------EDS----VGVIFKNGDDLRQDMLTLQMLRLMDLLWKEAGLDL-----------RMLPYGCLATGDRSGLIEVVSTSETIADIQLNSSNV-AATAAFNKDALLNWLKEYNS--GDD-------------------------LDRAIEEFTLSCAGYCVASYVLGIGDRHSDNIMVKK----TGQLFHIDFGHILGNFKSKFGIKRERVPFILTYDFIHVIQQ-GK--TGNTEKFGRFRQCCEDAYLILRRHGNLFITLFALMLTAGLPELTSVKD--IQYLKDSLALG-KSEEEALKQFKQKFDEALRESWTTKVNWMAHTVRKDY-----------------------------------------------------------------------------------------------------------------------------------------------------------------------------------------------------------------------------------------------------RS-----------------------------------------------------------------------------------------Mmus_PIK3CG/1-1102                            -------ME---------------------------------------------------------------------------------------------------------------------------------LENYEQP------------------------------------------------VVLREDNLRR--------RRRMKPRSA--------------------------------------------AGSLSSMELIPIEFV-----------------------------------------------------LPTSQRIS-----------------------------------------------------------------------------KTPETALL---------------------HVA----------------------------------------------------------------------------------------------------------------------------------------GHG---------NVEQMKAQVWLRALETS-----VAAE---------------------------------------FYHRL---------------------------------------------------------------------------GPDQFLLLYQKKG-----------------------------QWYEIYDRYQVVQ----------TLDCLHYWK------LMHKSPGQI----------------HV-----VQR----HVPSEE------------TLAFQKQLTSLIGY-------DVT----DISNVHDDELEFTRRRLVTPRMAEVAGRD------AKLYAMHPWVTSK-PL--PDYLSKKIAN---------NCIFIVI-------H-------------------RGTTSQT-IKVSADDTPGTILQSFFTKMA-KKKSLMNISE-------SQSEQDFVLRVCGRDEYLVG-------------ETPLKNFQ---------WVRQCLK---------NGDEIHLVLDTPPDPALDEVR-----------------------------------KEEWPLVD-----------------------------------------------------DCTGVTGYHEQLTIH---------------------------------------------------------------------------------------------GKDHESVFTVSLWDCDRK--FRVKIRGIDIPVLPR-----NTDLTVFVEANIQHGQQVLCQRRTS---------PKPFAEE------------V-LWN-VWLEF-GIKIKDLPKGALLNLQIYCCKTPSLSSKAS-------AETPGSESKGKAQLLYYVNLLLIDHRFLLRHGDYVLHMWQISGKAEE--------------QGSFNADKL---------TSATNPD------------------------KENS----MSISILLDNYC-HP----------------------------------IALPKHRPTP-------------------------------DPEGDRVR--------------------------------AEMPNQLRKQLEAII-----ATDPLN--PLTAEDKELLWHFRYESLK-HP-KAYPKLFS--SVKWGQQEIVA---KTYQLLARREIWDQSALDVGLTMQLLDCNFSDENVRAIAVQKL-ESLEDDDVLHYLLQLVQAVKFEPY-------------------------------------------------------------------------------------------------------------------------------------HDSALARFLLKRGLRNKRIGHFLFWFLR-SEIA-----------------QS-------RHYQQRFAVILEAYLRGCGT------AMLQDFTQQVHVIEMLQKVTIDIKSLS--AEKYDVSS-------QVISQLKQKLES-LQN-----SNLPESFRVPYDPGLKAGTLVI------------------------------------EKCKVMASKKKPLWLEFKCADPTVL----SNET----IGIIFKHGDDLRQDMLILQILRIMESIWETESLDL-----------CLLPYGCISTGDKIGMIEIVKDATTIAQIQQSTVG---NTGAFKDEVLNHWLKEKCP-IEEK-------------------------FQAAVERFVYSCAGYCVATFVLGIGDRHNDNIMISE----TGNLFHIDFGHILGNYKSFLGINKERVPFVLTPDFLFVMGSSGK---KTSPHFQKFQDVCVRAYLALRHHTNLLIILFSMMLMTGMPQLTSKED--IEYIRDALTVG-KSEEDAKKYFLDQIEVCRDKGWTVQFNWFLHLVLGIKQGE--------------------------------------------------------------------------------------------------------------------------------------------------------------------------------------------------------------------------------------------------KHSA---------------------------------------------------------------------------------------Mmus_PIK3CD/1-1047                            -----------------------------------------------------------------------------------------------------------------------------------------------MP-----------------------------------------------------------------------PGVD-CPMEFWTK---------------------------------------EESQSVVVDFL-----------------------------------------------------LPTG------------------------------------------------------------------------------------VYLNF---------------------PVS----------------------------------------------------------------------------------------------------------------------------------------RNA---------NLSTIKQVLWHRAQYE--------P----------------------------------------LFHMLSDP-------------------------------------------------------------------------EAYVFTCVNQTA-----------------------------EQQELEDEQRRLC----------DIQPF---------------LPVL----------------RL-----VAR-----EGDRV------------KKLINSQISLLIGK-------GLH----EFDSLRDPEVNDFRTKMRQFCEEAAAHRQQLGWVEWLQYSFPLQLEPSARGWRAGLLRVS-----------NRALLVNV-----KFE-------------------GSEESFT-FQVSTKDMPLALMACALR----KKATVFRQPL-------VEQPEEYALQVNGRHEYLYG-------------NYPLCHFQ---------YICSCLH---------SGLTPHLTMVH-----SSSIL-AMRDE------------------------------------------------------------------------------------------QSNPAPQVQKPR------------------AKPPPIPAKK--------------------------------------------------------------------------PSSVSLWSLEQP--FSIELIEGRKVNA-------DERMKLVVQAGLFHGNEMLCKTVSS---SEVN-VCSE----------------P-VWK-QRLEF-DISVCDLPRMARLCFALYAVVEKAKKARSTK-----------KKSKKADCPIAWANLMLFDYKDQLKTGERCLYMWPSVPDEK---------------GELLNPAG----------TVRGNPN------------------------TESA----AALVIYLPEVAPHP----------------------------------VYFPALEKIL-------------------------------ELGRHGER--------------------------------GRITEEEQLQLREIL-----ERRGSG--ELYEHEKDLVWKMRHEVQEHFP-EALARLLL--VTKWNKHEDVAQLSQMLYLLCS---W--PELPVLSALELLDFSFPDCYVGSFAIKSL-RKLTDDELFQYLLQLVQVLKYESY-------------------------------------------------------------------------------------------------------------------------------------LDCELTKFLLGRALANRKIGHFLFWHLR-SEMH-----------------VP--------SVALRFGLIMEAYCRGSTH-------HMKVLMKQGEALSKLKALNDFVKVSS--QKTTKPQ---------TKEMMHMCMRQ-ETY-----MEALSHLQSPLDPSTLLEEVCV------------------------------------EQCTFMDSKMKPLWIMYSSEEAG------SAGN----VGIIFKNGDDLRQDMLTLQMIQLMDVLWKQEGLDL-----------RMTPYGCLPTGDRTGLIEVVLHSDTIANIQLNKSNM-AATAAFNKDALLNWLKSKNP--GEA-------------------------LDRAIEEFTLSCAGYCVATYVLGIGDRHSDNIMIRE----SGQLFHIDFGHFLGNFKTKFGINRERVPFILTYDFVHVIQQ-GK--TNNSEKFERFRGYCERAYTILRRHGLLFLHLFALMRAAGLPELSCSKD--IQYLKDSLALG-KTEEEALKHFRVKFNEALRESWKTKVNWLAHNVSKDN-----------------------------------------------------------------------------------------------------------------------------------------------------------------------------------------------------------------------------------------------------RQ-----------------------------------------------------------------------------------------Mmus_PIK3C2A/1-1686                           -------MAQISN------------NSEFKQCSSSHPEPIRTKD---------------------------------------------------VNKAEALQMEAEALAKLQKDRQMT---------------------------------------DSPRG--FELSSSTRQRTQGFNKQDYDLMV-------FPELD-----------SQK-----RAVDIDVEKLTQAELEKIL---LDDNF----------ETRKPPALPVTPVLSPSFSTQLYLRPSGQRGQ-----------------WPPGLCGPSTYT---------------LP-------------------------------------------------STYPSA-YSKQ-ATFQN----GFSPRMPTFPS--TESVYLRLP--------------------GQSPYFSYPLTPATP-------FHPQGSLPVYRPL--------------VSPDMAKLFE---------------------------------------------------------------------KIASTSEFL--KNG------------KARTDLEIANSKASV---CNLQI-------------------SPKSEDINK-------FDWLDLDPLSKPKVDYVEVLE-------------------HEEEKK--DPVLLAEDPWDAVLLEERS-P----------------SCHLERKVNGKS-----------------------------LSGATVTRSQSLI------------------------------IRTA----------------QF------------TKAQGQ----------VSQKDPNGTSSLPTGS-------SLL----QEFEVQNDEVAAFCQSIMKL---------------KTKFPYTDHCTN------PGYLLSPVTVQRN-MCGENASVKVSI-----EIE-------------------GLQLPVT-FTCDVSSTVEIIIMQALC----WVHDDLNQ----------VDVGSYILKVCGQEEVLQN-------------NHCLGSHE---------HIQNCRK---------WDTEIKLQLLT-----LSAMC-QNLARTAEDDEAPVDLNKYLYQIEKPYK-EVMTRHPVEELLDSYHYQVELALQTE-----------------NQHRAVDQVIKAVRKICSALDGVETPSVTEAVKKLKRAVNLPRNKSADVTSL-SGSDTRKNSTK------------GSLNPENP-------VQVSMDHLTTAIYDL--LRLHANSSR-----------CSTGCPRGSRNIKEAWTATEQ--LQFTVYAAHGISSNWV----SNYEKYYLICSLSHNGKDLFKPIQS---KKVG-TYKNFFYL------------I-KWD-ELIIF-PIQISQLPLESVLHLTLFGVLNQSSGSSPD-----------SNKQRKGPEALGKVSLTLFDFKRFLTCGTKLLYLWTSSH---------------------TNSIPG---------AIPKKSY------------------------VMER----IVLQVDFPSPA-FD----------------------------------IIYTSPQIDR-------------------------------NIIQQ-------------------------DK-----L--ETLESDIKGKLLDII-----HRDSSF--GLSKEDKVFLWENRYYCLK-HP-NCLPKILA--SAPNWKWANLA---KTYSLLHQ---W--PPLCPLAALELLDAKFADQEVRSLAVSWM-EAISDDELADLLPQFVQALKYEIY-------------------------------------------------------------------------------------------------------------------------------------LNSSLVRFLLSRALGNIQIAHSLYWLLK-DALH-----------------DT--------HFGSRYEHVLGALLSVGGK------GLREELSKQMKLVQLLGGVAEKVRQAS--GSTRQVV-------------LQKSMER-VQS-----FFLRNKCRLPLKPSLVAKELNI------------------------------------KSCSFFSSNAMPLKVTMVNADPL-------GEE----INVMFKVGEDLRQDMLALQMIKIMDKIWLKEGLDL-----------RMVIFRCLSTGRDRGMVELVPASDTLRKIQVEYGV----TGSFKDKPLAEWLRKYNP-SEEE-------------------------YEKASENFIYSCAGCCVATYVLGICDRHNDNIMLRS----TGHMFHIDFGKFLGHAQMFGSFKRDRAPFVLTSDMAYVING-GE---KPTIRFQLFVDLCCQAYNLIRKQTNLFLNLLSLMIPSGLPELTSIQD--LKYVRDALQPQ-TTDAEATIFFTRLIESSLG-SIATKFNFFIHNLAQLRFSGL--PSNDEPILSFSPKTYS-FRQDGRIKEVSVFTYHKKYNPDKHYIYVVRILREGHLE--PSFVFRTFDEFQELHNKLSIIFPLWKLPGFPN--------RMVLGRTHIKDVAAKRKIELNSYLQSLMNASTDVAECDLVCTFF-----HPLLRDEKAEG----------IARSAG--AVPFSPTLGQIGGAVKLSVSYR--NGTLFIMVMHIKDLV--TEDGADPNPYVKTYLLPDTHKTSKRKTKISRKTRNPTFNEMLVYSGYSKETLRQRELQLSVLSAESL---RENFFLGGITLPLKDFNLSKETVK----WYQLTAATYL------Mmus_PIK3C2B/1-1632                           -------MSST---------------QGNGEHWKSLESVGISRK--------------------------------------------------ELAMAEALQMEYDALSRLRHHKEES--------------------------------------------------------------------------------------------RAKQNTEPSLISWDEPALDFYS-------------------KPAGRRTDLKLLRGLSGSDPTLNYNSI---------------------SPPEGLPN-STSQDP-----------------QPGPDP-----------------------------------------------WPKGSLS-------------------------GDYLYIFDG--------------------SEGRCSLSPGSGDTD-------GSCKKLSPPPLP-----------------PR-------------------------------------------------------------VSIWDAPPLPPR--KGSPSPS----KISQPNDINSFSSAEQPPDKLLVAQDPE------EGEL--PDGRG------------------------QGHTLGSVDYDGINDA------------------------------------ITRLNLKSTYDSEISSDATRG-----------WK---EGRGPLDFNKDT------------------------------SGKPVARSKTMP----------------------------PQVPPR----------------TY-TSRYANRKNATPGNNRR---------ISAAPVGSRPHTVANGH-------ELF----EVSEERDEEVAAFCHMLDIL---------------RTGSDIQDYSLT-------GCVWSTVTPSPE-HLGDEVNLKVTV-----LCD-------------------SLREPLT-FTCNCSSTVDLLIHQTLC----YTHDELRE----------VDVGDFVLKPCGLEEFLQN-------------KHALGSHE---------YIQYCRK---------FDISIRLQLME-----QKAIR-SDLARTVNDDQSPSTLNYLIHLQERPVK-QTISRQALSLLFDTYHNEVDAFLLADGD----------------FPLKADRVVQSVKAICNALAAVETPEITSALNQLPPCPSRMQPKI-------QKDPSVLSVR--------------------------ENREKVVEALTAAILDL--VELYCSTFNADFQT-------AVPGSRKHDLVQEACHFPGA--LAFTVYGTHRIPIIWA----TSYEDFYLSCSLSHGGKELCSPLQT---RRAH-FSKYLFHL------------I-IWD-QQICF-PVQVNRLPRETLLCATLYALPVPPPGGSSE-----------ANKQKRVPEALGWVTTPLFNFRQVLTCGRKLLGLWPATQ---------------------ENSGAR---------WSAPNFH------------------------QPDS----VILQIDFPSSA-FD----------------------------------IKFTSPPGDK-------------------------------FSPR--------------------------YE-----F--GSLREEDQRKLKDIT-----QKESLY--WLTDADKKQLWEKRYYCHT-EV-SSLPLVLA--SAPSWEWACLP---DIYALLQQ---W--THMNHQDALGLLHATFPDQEVRRMAVQWI-GSLSDAELLDYLPQLVQALKYECY-------------------------------------------------------------------------------------------------------------------------------------LDSPLVRFLLKRAISDLRVTHYFFWLLK-DSLK-----------------DS--------QFSIRYQYLLAALLCCCGK------GLREEFNRQCWLVNTLAKLAQQVREAT--PSARQGI-------------LRVGLEE-VKQF----FALNGSCRLPLSPSLLVKGIVP------------------------------------RDCSYFNSNAVPLKLAFQNVDPL-------GEN----IRVIFKCGDDLRQDMLTLQMIRIMSKIWVQEGLDM-----------RMVIFRCFSTGRGKGMVEMIPNAETLRKIQVEHGV----TGSFKDRPLADWLQKHNP-GEDE-------------------------YEKAVENFIYSCAGCCVATYILGICDRHNDNIMLKT----TGHMFHIDFGRFLGHAQMFGNIKRDRAPFVFTSDMAYVING-GD---KPSSRFHDFVDLCCQAYNLIRKHTHLFLNLLGLMLSCGIPELSDLED--LKYVYDALRPQ-DTEANATTYFTRLIESSLG-SVATKLNFFIHNLAQMKFTG----SDDRLTLSFAPRTHT-LKSSGRIRDVFLCRHEKIFHPSKGYVYVVKVMRENAHE--ATYIQRTFEEFQELHNKLRLLFPSSFLPSFPS--------RFVIGRSRGEAVAERRKEELNGYIWHLIHAAPEVAECDLVYTFF-----HPLPRDEKASGP--SP-----APKSSDG---TWARPVGKVGGEVKLSISYK--NNKLFIMVMHIRGLQP-LQDGNDPDPYVKIYLLPDPQKTTKRKTKVARKTCNPTYNEMLVYDGIPKGDLQQRELQLSVLSEQGF---WENLLLGEVHIRLRELDLAQEKTG----WFGLGSRGHGT-----Mmus_PIK3C2G/1-1506                           -------MAYSWQTEPN--------RTEPQEDGSDTQQFHHTNQ---------------------------------------------------HLSSSQVRLGFDQLVEEINNK---------------------------TP----------------------------------------------------------------L-SESEKEEDTYFVPDAPNLGSKW---------------------------PSIYETHPRYFSEFTSQSPDSSQLRFGK------------LSAIGFNPAV----------------------LPTHQLIHEGA--------------------------------------------------------------------SWRN----------------------------------------------------------------------------------PSGKYH---GI-EYPRF-------------------------------------------------DALPP-------------------------------SSTGQGECNPQGQS-----------------------------GTKHHNYCG-----------EHEGNLPHHH-----------------------------------------------------------------------SSYSIDSIPNREKR------------RSGDV-------------NLVEPSLEFS--------------------------KDSFLPRT----------------------------------------------SENVSVESTEPIGCPI-------EIV----EVPQGSNKNLASFCNKVKKI---------------RESYHASDINSN------SGKIWAITTAYPS-RLFADTKFRVKI-----SID-------------------NSAQLLL-LMPHANYLVKDLIAEILL----LCANE------------PLSPKEYLLSVCGSEEFLQM-------------DHSLGGHK---------IFQK------------NKSVIQLHLQK-----NRDTP-GKLSRKSEDDHSPFHLN---QLLEFTHI-WKISRQCLSTVMKKYNLHVEHLLKPQKDMEEKHLSSMVSGNQHTSQPHVNNVLEEVKNICSVLGCIETKQVSDAVKELNLILQR------------PSQNFHQNSET---------------SKKGF-------IERVTAELSRSIYQL--IDVYCSTFCTDFQP----------VHTPGGVSRVHAGLQSH--LSFTVCSLHNVPETWA----HSYKAFSFSCWLTYAGKKLCQVKSC---RPLP-VTKSFSLL------------V-NWN-EIINF-PLEIKSLPRESMLIIKLFGID----------------------SATHSTNLLAWTCLPLFPRQESVL-GSRLFSVTLQSEPP-------------------IEMIA----------PGVWDGS------------------------QPSP----LTLQIDFPDAG-WE----------------------------------YLKPESEENR-------------------------------------------------------------TD-----H--EEPPRECLKHIAKLS-----QKKSPL--LLSEEKRRYLWFYRLYCNN-EN-SSLPLVLG--SAPGWDEETVS---EMHAILRR---W--TFSHPWEALGLLTSRFPDQDIREVAVQQL-DTLLTDELLDCLPQLVQAVKFEWN-------------------------------------------------------------------------------------------------------------------------------------LESPLVELLLRRSLQSIRVAHRLYWLLR-DAQG-----------------EA--------YFKSWYQELLAALQFCAGE------ALNEELSKEQKLVKLLGDIGEKVKSAS--DPQRKDV-------------LKKEIGS-LEEF----FKDIKTCHLPLNPALCIKGIDR------------------------------------DACSYFTSNASPLKITFINANPM-------GKN----ISVIFKAGDDLRQDMLALQIIQVMDNAWLQEGLDM-----------QMITYGCLSTGRAQGFIEMVPDAVTLAKIHLHSGL----IGPLKENTIKKWFSQHNH-LKED-------------------------YEKALRNFFYSCAGWCVVTFILGVCDRHNDNIMLTK----SGHMFHIDFGKFLGHAQTFGGIKRDRAPFIFTSEMEYFITE-GG---KNTQHFQDFVELCCRAYNIVRKHSQLILSLLEMMLHAGLPELRGIED--LKYVHNNLRPQ-DTDLEATSHFTKKIKESLE-CFPVKLNNLIHTLAQMPALS----LAKPAPQTLLQESCI-LNKTRTIQRVTILGFSKTHS----NLYLMEVTCSDNR---RSLTKKSFEQFYRLHSQMQKQFSSLALPEFPH--------WWHLPFTDSD---HKRIRDLSHYVEQVLRGSYEVANSDCVLSFFLSEHIQPTLEDSPFVDP---------GENSLDK------------SPKVQLLMTYE--DSRLTILVKHLKNIH--LPDGSVPSAHVEIYLLPHPSEVRRKKTKCVPKCTDPTYNEIVVYD--EVLGLQGHVLMLIVKS--------KTVFVGAVNIQLCSVPLNEEK------WYPLGNSII-------Mmus_PIK3C3/1-887                             -------MG--------------------------------------------------------------------------------------------------------------------------------------------------------------------------------------------------------------------------------------------------------------------------------------------------------------------------------------------------------------------------------------------------------------------------------------------------------------------------------------------------------------------------------------------------------------------------------------------------------------------------------------------------------------------------------------------------------------------------------------------------------------------------------------------------------------------------------------------------------------------------------------------------------------------------------------EAE----------------------------KFHYIYSCD--------------------------LDINVQLKI----GSLE----------------GK-REQKSYK-----------------------------------------AVLEDPMLKFSG-------------------------LYQ--------------------------------------------------------------------------------------------------------------------------------------------------------------------------------------------------------------------------------------------------------------------------------------------------ETCSDLYVTCQVFAEGKPLALPVRT--------SYKAFSTR------------W-NWN-EWLKL-PVKYPDLPRNAQVALTIWDVY-----------------------GPGSAVPVGGTTVSLFGKYGMFRQGMHDLKVWPNVEADGSEPTRTPGRTSSTLSEDQMSRLAK---------LTKAHRQGHMVKVDWLDRLTFREIEMINESEKRSS--NFMYLMVEFRCVKCDDK-EYGIVYYEKDGDES---------SPILTSFELVKVPDPQMSM------------------------------ENLVESKHHKLARSLRSGPSD----------HD--------LKPNATTRDQLNIIV-----SYPPTK--QLTYEEQDLVWKFRYYLTN-QE-KALTKFLK--CVNWDLPQEAK---QALELLGK---W--KPMDVEDSLELLSSHYTNPTVRRYAVARL-RQADDEDLLMYLLQLVQALKYENFDDIKNGLEPTKKDS----------------QTSASESLSNS---GVSS-------------GDIDSS--QIIT----------------------------NPLPPV---------ASPPPASKAKEVSDGENLEQDLCTFLISRACKNSTLANYLYWYVI-VECE-----------------DQDTQQ-RDPKTHEMYLNVMRRFSQALLKGDKSVRVMRSLLAAQQTFVDRLVHLMKAVQRES--GNRKKKN-----------ERLQALLGD-NE---KMNLSDVELIPLPLEPQVKIRGIIP------------------------------------ETATLFKSALMPAQLFFKTED---------GGK----YPVIFKHGDDLRQDQLILQIISLMDKLLRKENLDL-----------KLTPYKVLATSTKHGFMQFI-QSVPVAEVLDTEGS------------IQNFFRKYAP-SETGP---------------------YGISAEVMDTYVKSCAGYCVITYILGVGDRHLDNLLLTK----TGKLFHIDFGYILGR-----DPKPLPPPMKLNKEMVEGMGG------TQSEQYQEFRKQCYTAFLHLRRYSNLILNLFSLMVDANIPDIALEPDKTVKKVQDKFRLD-LSDEEAVHYMQSLIDESVH-ALFAAVVEQIHKFAQ----------------------------------------------------------------------------------------------------------------------------------------------------Y-----------------------------------------------------------------------------------------------------------------------------------------------------------------------------WRK-------------Ggal_PIK3CA/1-1068                            -----------------------------------------------------------------------------------------------------------------------------------------------MPP---------------------------------------------------------------------RPSSG----ELWGIH--------------------------------------LMPPRILVECL-----------------------------------------------------LPNG------------------------------------------------------------------------------------MIVTL---------------------ECL----------------------------------------------------------------------------------------------------------------------------------------REA---------TLLTIKHELFKEARKY--------P----------------------------------------LYQLLQDE-------------------------------------------------------------------------SSYIFVSVTQEA-----------------------------EREEFFDETRRLC----------DLRLF---------------QPFL----------------KV-----IEP-----VGNRE------------EKILNREIGFAIGM-------PIC----EFDMVKDPEVQDFRRNILNVCKEAVDLRDANAPHSRALYVCPPNVESSPEL--PKHIYNKLDK---------GQIIVVIWVI-VSPN-------------------NDKQKYT-LKINHDCVPEQVIAEAIR----KKTRSMLLSSEQLKLCVLEYQGKYILKVCGCDEYLLE-------------KYPLSQYK---------YIRSCIM---------LGRMPNLMLMA-----KESLY-TQLPL------------------------------------------------------------------------------------------DTFTMPSYSRRI------------------STATPYMNGE--------------------------------------------------------------------------ATAKSLWTINSA--LRIRILCATYVNVNI-----RDIDKIYVRTGIYHGGEPLCDNVNT---QRVP--CSN----------------P-RWN-EWLSY-DMYIPDLPRAARLCLSICSVKGRKGAK-------------------EEHCPLAWGNINMFDYTDTLVSGKMALNLWAVPHGL----------------EDLLNPIG----------VTGSNPI------------------------R-ET----PCLELEFDWFS-NP----------------------------------VKFPDMTVIE-------------------------------EHANWTISRELGFNYS--YAGLSNRI----AR-----D--NELRESDKEQLRAIC-----TRDPLS--EITEQEKDFLWSHRHYCVN-TP-EILPKLLL--SVKWNSRDEVA---QMYCLVKD---W--PPIKPEQAMELLDCNYPDPMVRAFAVRCLEKYLTDDKLSQYLIQLVQVLKYEQY-------------------------------------------------------------------------------------------------------------------------------------LDNQLVRFLLKKALTNQRIGHFFFWHLK-SEMH-----------------NK--------TVSQRFGLLLESYCRACGM-------YLKHLSRQVEAMEKLINLTDILKQEK--KDETQK---------VQMKFLVEQMRR-PDF-----MDALQGFISPLNPAHQLGNLRL------------------------------------EECRIMSSAKRPLWLNWENPDIM-------SELLFQNNEIIFKNGDDLRQDMLTLQIIRIMENIWQNQGLDL-----------RMLPYGCLSIGDCVGLIEVVRSSHTIMQIQCKGGL--KGALQFNSHTLHQWLKDKNK--GEM-------------------------YDAAIDLFTRSCAGYCVATFILGIGDRHNSNIMVKD----DGQLFHIDFGHFLDHKKKKFGYKRERVPFVLTQDFLIVISK-GAQECTKTREFERFQEMCYKAYLAIRQHANLFINLFSMMLGSGMPELQSFDD--IAYIRKTLALD-KTEQEALEYFMKQMNDAHHGGWTTKMDWIFHTIKQHALN----------------------------------------------------------------------------------------------------------------------------------------------------------------------------------------------------------------------------------------------------------------------------------------------------------------------------------------------Ggal_PIK3CB/1-1066                            -----------------------------------------------------------------------------------------------------------------------------------------------MP-----------------------------------------------------------------------PAVT-DSLDIWAVDSQI-----------------------------------GADGSISVDFL-----------------------------------------------------LPTG------------------------------------------------------------------------------------IYINL---------------------DVP----------------------------------------------------------------------------------------------------------------------------------------RDA---------TISHIKQLLWKQAHTY--------P----------------------------------------LFHLLMEI-------------------------------------------------------------------------DSYMFSCVNQTA-----------------------------VHEELEDETRRLC----------DVRPF---------------LPVL----------------KL-----VTR-----NCDPG------------EK-LDSKIGVLIGK-------GLH----EFDALQDPEVNDFRAKMRRISEEKIQSLVGLSWMDWLKHTYPPEQEPVV----PENFQDKLY---------SGNLVVAI-----HFD-------------------NCQDVFS-FQVSPNMNPIKLNELAIR----KRLTIHGKEDE------EIDPADYVLQVSGRLEYVFG-------------DHPLIQFQ---------YIHNCVM---------NRTLPQLTLVE-----CCTIK-KMCEQ------------------------------------------------------------------------------------------EMIAIEVAINRKSS----------------NLPLPLPPKK-------------------------------------------------------------------------TRATTSVWDICSP--FKIVLLKGNKLNT-------EENAKVHVRAGIFHGTELLCKTIVS---TEIS-GRSD----------------H-AWN-EVLEF-EINVCDLPRMARLCFAVYAVMDKMKTKKSTKSMNPSKY-QTIRKAGKVHYPVAWVNTMVFDYKGQLKNGEFVLHSWSSFPDEL---------------EEMLNPMG----------TVQTNPY------------------------TENA----TALHIRFQEYSKQP----------------------------------INYPPFDKIL-------------------------------EKAAEIAR---------------------SSDNAAM-A--GRGGKKFYIVLKEIM-----ERDPLS--QLCENEMDLIWTLRYDCRENFP-QSLPKLLL--SLKWNKLEDVA---QLQALLQI---W--PKLLPREALELLDFNYPDQYVREYAVGCL-KQMSDEELSQYLLQLVQVLKYEPF-------------------------------------------------------------------------------------------------------------------------------------LDCALSRFLLERALANRRIGQMLFWHLR-SEVH-----------------IP--------AVSVQFGLILEAYCRGSVA-------HMKVLAKQVEALNKMKTLNSLIKLNA--MKQSKAK---------GKDAMHTCLKQ-NAY-----REALSDLQSPLNPSVILSELHV------------------------------------EKCKYMDSKMKPLWIVYNNKMFG-------GDL----VGIIFKNGDDLRQDMLTLQILRLMDVLWKEAGLDL-----------RILPYGCLATGDHSGLIEAVSSSETIADIQLNSSNV-AAAAAFNKDALLNWLKEYNL--GDD-------------------------LDRAIEEFTLSCAGYCVATYVLGIGDRHSDNIMVRK----NGQLFHIDFGHILGNFKSKFGIKRERVPFILTYDFIHVIQQ-GK--TGNTEKFGRFRQYCEEAYLILRKHGNLFITLFALMLTAGLPELTSVKD--IQYLKDSLALG-KSEEEALKQFKQKFDEALRESWTTKVNWMAHTVRKDY-----------------------------------------------------------------------------------------------------------------------------------------------------------------------------------------------------------------------------------------------------RS-----------------------------------------------------------------------------------------Ggal_PIK3CG/1-1106                            -------ME---------------------------------------------------------------------------------------------------------------------------------LGDYEQP------------------------------------------------VVMREENKRR--------RRRMKPHCT--------------------------------------------SSNLSSMELISIEFI-----------------------------------------------------LPTSNKHT-----------------------------------------------------------------------------KVPEMMLL---------------------EIA----------------------------------------------------------------------------------------------------------------------------------------GNC---------TVEQMKAQIWMRAIEMS-----QTTD---------------------------------------FYHTF---------------------------------------------------------------------------TPDQFVLQYQKKG-----------------------------QWYEIYDKHQLLQ----------TLDCILYWK------VLQKKVGKI----------------YV-----VQK----QKPSEE------------VQEFQRQLNDLIGY-------DVT----DVSNVHDDELEFTRRRLVTPRMIEVACRD------PKLYAMHPWTTSK-PL--PEYLFKKITN---------NNIFIII-------H-------------------RGTTSQK-IKVSIDDTPDMILHSFFTKMA-KKKSLMDIPE-------DHSELDFVLRICGRDEYITG-------------DTPIKDFH---------WIRQCLK---------NGEEIHLVLDNPPDPSEDEVQ-----------------------------------KEEWPLVD-----------------------------------------------------DCTGVTGYHEQLTID---------------------------------------------------------------------------------------------GKDHERVFTISLWDCNRK--FRVKIIGIDIPVLPR-----NTDLTVFVEANIQHGQQLLSQRRTS---------SKPFTEE------------V-LWN-IWLEF-DIKIKDLPKGALLNLQIYCGKAQGLSTKTNLQ----SHESPNSDSKCKTQLLYYVNLLLIDHRFLLRSGEYVLHMWKIPGKGEE--------------QGSINADKL---------TSATNPD------------------------KENS----MAISIVLDKYC-HP----------------------------------IALPKHRITS-------------------------------DSQGDRTR--------------------------------AEMPNQLRKQLEEII-----ATDPLN--PLSPEDKELLWHFRYESIK-HP-KAYPKLLS--SVKWGQQEIVA---KTYQLLAKKEVWDQSTLDVGLTMQLLDCNFSDENVRAMAVQKL-ESLEDDDVLHYLLQLVQAVKFEPY-------------------------------------------------------------------------------------------------------------------------------------HDSALARFLLKRGLRNKRIGHFLFWFLR-SEIA-----------------QS-------MHYQQRFAVILEAYLRGCGK------AMLHDFMKQVQVIELLHKVTMEIKSVS--AEKYDVTS-------QVIAQLRQKLEK-LQG-----SKLPESFRVPYDPGLRAGPLVI------------------------------------EKCKVMASKKKPLWLEFKCADPTAL----SNET----IGIIFKHGDDLRQDMLILQILRIMESIWEAESLDL-----------CLLPYGCISTGNKIGMIEIVKDATTIAKIQQSTVG---NTGAFKDEILNQWLKDRCV-IEEK-------------------------FQAAVERFVYSCAGYCVATFVLGIGDRHNDNIMITE----TGNLFHIDFGHILGNYKSFLGINKERVPFVLTPDFLFVMGTSGK---KTSLHFHKFQDVCVKAYLALRHHTNLLIILFSMMLMTGMPQLTSKED--IEYIRDALTVG-KSEEDAKKHFLDQIEVCRDKGWTVQFNWFLHLVLGIKQGVE-------------------------------------------------------------------------------------------------------------------------------------------------------------------------------------------------------------------------------------------------KHSA---------------------------------------------------------------------------------------Ggal_PIK3CD/1-1046                            -----------------------------------------------------------------------------------------------------------------------------------------------MP-----------------------------------------------------------------------PGIY-CPMEFWSK---------------------------------------GENQNIQVDFL-----------------------------------------------------LPTG------------------------------------------------------------------------------------IYLNL---------------------SVP----------------------------------------------------------------------------------------------------------------------------------------CNA---------SLDTIKQVVWKHAQYE--------P----------------------------------------LYHMLSDP-------------------------------------------------------------------------EAYVFTCINQTA-----------------------------EQQELEDEQRRLC----------DIQPF---------------LPVL----------------RL-----VAR-----EGDRV------------KKLINSQISLLIGK-------GLH----EFDSVQDPEVNDFRTKMCQFCEERAAKRQQLSWAAWMEYNFPLQLEPMAKGLGTGLLHTP-----------IKNIFVNV-----KFQ-------------------SGGESFT-FQISPNEFPITLMSYAVK----KQATVFRHET-------MENPEDYTLQVNGKYEYLYG-------------NYPLYQFQ---------YIRSCLH---------RGLTPHLTMVH-----SSTII-AMRDE------------------------------------------------------------------------------------------QVNSIANPPKMA------------------VKPPPLPKKK--------------------------------------------------------------------------PNYGSLWSLEQS--FYIELVQGSKVNA-------DERMKLVVQAGLFHGNEMLCKTVSS---SEVN-VCSE----------------P-VWK-QRLDF-DINICDLPRMARLCFALYAVIEKAKKARSTK-----------KKSKKADCPIAWVNVMLFDYKDQLKTGECCLHMWSSFPDEK---------------GELLNPMG----------TVQCNPN------------------------TESA----AALVICFPNVASHP----------------------------------VYYPSFEQLL-------------------------------ELGRNGEQPR---------------------------S--APEDPEEKLQLKEIL-----ERRNHT--ELYEHEKDLVWKMRYDIRDQYP-QALAKLLT--ITKWNKHEDVA---QMISLLQT---W--PELPVLNALELLDFSFPDRYVGSFAINSL-KKLTDHELFQYLLQLVQVLKYESY-------------------------------------------------------------------------------------------------------------------------------------LDCELTKFLLDRALSNRKIGHFLFWHLR-SEMH-----------------VP--------AVALRFGLILEAYCRGSTH-------HMKVLMKQGEALSKMKALNEFVKLSS--PKATKPQ---------AKEMMHVCMKQ-ETY-----LEALSHLQSPLNPSIILTEVCV------------------------------------DQCTFMDSKMKPLWIVFNNEETG-------GGG----VGIIFKNGDDLRQDMLTLQMIQLMDVLWKQEGLDL-----------RMTPYGCLSTGDKTGLIEVVMHSDTIANIQLNKSNM-AATAAFNKDALLNWLKSKNP--GDA-------------------------LEQAIEEFTLSCAGYCVATYVLGIGDRHSDNIMIRE----TGQLFHIDFGHFLGNFKTKFGINRERVPFILTYDFVHVIQQ-GK--TNNSEKFERFRGYCEKAYMILRRHGPLFLHLFALMKAAGLPELTCSKD--IQYLKDSLALG-KTDEEALKHFRLKFNEALRESWKTKVNWLAHNVSKDN-----------------------------------------------------------------------------------------------------------------------------------------------------------------------------------------------------------------------------------------------------RQ-----------------------------------------------------------------------------------------Ggal_PIK3C2A/1-1700                           MDICPRGMAQLSS------------SNGFKQRSSPSLATARSKD---------------------------------------------------VDKEEALQMEAEALAKMQKERGIA---------------------------------------GNKQT--SHSLSSTGHVAQTHSKQDHDLMV-------FPESD-----------AKR--MEDKLSTIDIEKLTHAELEKLL---LDDSF----------ESSKVPALPVTPILSPSLSSQFYIGPTGQRGQ-----------------WTPGIPAPVTCT---------------LP-------------------------------------------------PIYPSASYIKQTSVFQN----GFHPSMSSYRS--REPIYFGLP--------------------RQSQYISYPLAATTP-------FHPQGSLPIYPPV--------------LTPELAKVFD---------------------------------------------------------------------KIASTSEFL--RNG------------KSSTDLEMTAIKSAV---SSLPA-------------------SEKCRDISK-------FDWLDLDPLSKPKVDSVETLY-------------------KAEDRGEMASGMTAEDPWDAVLLKEKLLV----------------TCHLERKINGKS------------------------------SGATVTRSQSLN------------------------------MRTT----------------QL------------GKLQGQ----------TSQKD-NGTTGTLTEN-------VLL----QEMEGQNQELSAFSQAVTKL---------------RTKFPYTDQQSN------PGFVLSPIMLQRN-ITGESASIKVSI-----EIK-------------------GFQQPVT-FTCDVSSPVELIIMQALC----WVHDDLNE----------VDIGSYFLKVCGQEEVLQN-------------KHCVGSHE---------YIQNCRK---------WDTEIKLQLVT-----HTEIC-RGLARTEEDDSTPIDLTKHLYKVEKPFR-EPNIRSSIEELLDIYHKQIEIALKNE-----------------NQHKAVDHLIKTVRKICSTLDGVETPAITESVKKLRRAVNLPRIKDTEVSLK-CGDDASKSSS-------------GSLQPESP-------LKVSIDLLTRAVEAL--VRLHMNSCSM----------SSATVSPEDKTTKEAWTATEH--LQFTIFAAHGIFPDWV----SNFEKYYLICSLTHNGKDLFKPVQS---KKAG-AYKNFFYL------------I-KWD-ELIIF-PIQISQLPLESVLCLTLFGILNQSSGSSPD-----------SNKQRKGPEMLGQVSLPLFDFRRLLSCGTKLLQLWTSLH---------------------PSHASG---------TASKKEN-------------------------MGR----IVLQVDFPSHA-FD----------------------------------IEYKIPQAAK-------------------------------TTVH--------------------------HS-----I--EALEKPLRERLLAIL-----SRDNTI--GLSKEDKEFLWERRHYCHG-HT-NSLPKILA--SAPHWDWASLP---EIYSLLQQ---W--PPLSPLAALELLDSKFADQEVRNTAVNWI-ETLSDDELTDFLPQFVQALKYETY-------------------------------------------------------------------------------------------------------------------------------------LDSALVKFLLARALGNIRIAHYLYWLLK-DTLY-----------------DP--------KFGVRYEQILGAFLSVCGK------GLREELEKQTRLVLLFGMVAEKVKQTS--VSGRQTA-------------LQSGMER-VQS-----FFLKNKCRLPLNPSLVAKELNI------------------------------------KACSFFSSN
[truncated: 491,581 more chars]
